# Supplementary material for: Gold-catalyzed four-component multifunctionalization of alkynes
Source: Nat Commun. 2023 Jun 15;14:3551. doi: 10.1038/s41467-023-39243-5 (PMC10272212; doi:10.1038/s41467-023-39243-5)
Supplement: Supplementary file 1 — Supplementary Information [file 41467_2023_39243_MOESM1_ESM.pdf]

Supplementary Information

Gold-Catalyzed Four-Component Multifunctionalization of Alkynes

Shangwen Fang,[1] Jie Han,[1] Chengjian Zhu,[1,2] Weipeng Li[1] and Jin Xie[1,3]*

[1]State Key Laboratory of Coordination Chemistry, Jiangsu Key Laboratory of Advanced Organic Materials, Chemistry and Biomedicine Innovation Center (ChemBIC), School of Chemistry and Chemical Engineering, Nanjing University, Nanjing 210023, China

[2]Green Catalysis Center, and College of Chemistry, Zhengzhou University, Zhengzhou, Henan 450001, China

[3]State Key Laboratory of Chemistry and Utilization of Carbon Based Energy Resources, College of Chemistry, Xinjiang University, Urumqi 830017, China

Email: xie@nju.edu.cn

Table of Contents

1. General information 3

2. Optimization details 4

3. General procedure 9

4. Characterization data for the products 10

5. X-ray structure of products 47

6. Mechanistic study 50

7. Preparation of starting materials 56

8. NMR spectra 69

9. References 182

1. General information

All reactions were conducted in oven- or flame-dried glassware under an air atmosphere. Unless otherwise noted, all reagents were used as received and handled under air atmosphere. Chloroform-d_1 was purchased from J & K Scientific Ltd.

NMR spectra were recorded on a Bruker Ultra-shield 500 and 600MHz spectrometer. ^{1}H NMR, ^{13}C NMR and ^{19}F NMR are recorded on an NMR spectrometer with $CDCl_3$ as solvent. Chemical shifts of ^{1}H, ^{13}C, ^{19}F and ^{31}P NMR spectra are reported in parts per million (ppm). The ^{19}F NMR spectra is {1H} decoupled and the ^{13}C NMR spectra is {1H} decoupled. The residual solvent signals were used as standard, and the chemical shifts were converted to the corresponding scale ($CDCl_3$: δ H = 7.26 ppm, δ C = 77.00 ppm). All coupling constants (*J* values) were reported in hertz (Hz). Multiplicities are reported as follows: singlet (s), doublet (d), doublet of doublets (dd), triplet(t), quint (quintet), and multiplet (m). GC-MS analyses were performed on a GC-MS with an EI mode. HRMS (ESI) was determined on the Micromass Q-TOF instrument. The IR spectrum was recorded on a Brucker Alpha FT/IR instrument. Schlenk tubes (10 mL and 500 mL) were purchased from synthware. Toppette was purchased from DLAB Scientific Co., Ltd. The compound names were generated by the computer program ChemDraw according to the guidelines specified by the International Union of Pure and Applied Chemistry (IUPAC).

All reagents were purchased from commercial suppliers, Aladdin, Adamas-beta®, TCI (Shanghai) Development Co., Ltd, Energy Chemical, J & K scientific Ltd., Bide Pharmatech Ltd, Alfa-Aesar and Sigma-Aldrich unless otherwise noted.

2. Optimization details

2.1 Initial screening for directing groups

Supplementary Table 1 Investigation of directing groups[a]

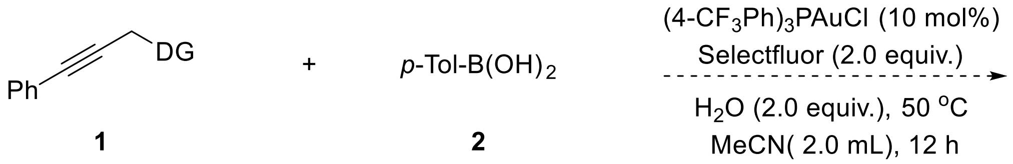

1 2

directing group	results	yield (%)[b]
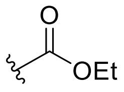	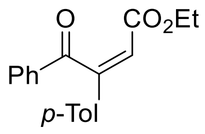	38
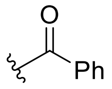	no alkyne multifunctionalization product	-
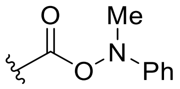	no alkyne multifunctionalization product	-
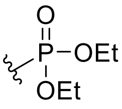	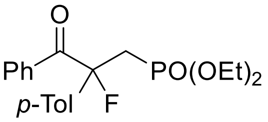	43
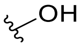	no alkyne multifunctionalization product	-
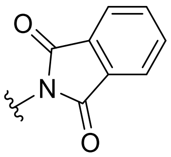	no alkyne multifunctionalization product	-
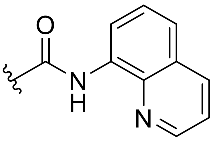	no alkyne multifunctionalization product	-

[a]Standard reaction conditions: (4-$CF_3Ph)_3PAuCl$ (10 mol%), **1** (0.1 mmol), **2** (0.3 mmol), Selectflour (0.2 mmol), H_2O (0.2 mmol), MeCN (2.0 mL), the reactions are carried out at 50 °C under air atmosphere for 12 h; [b]Isolated yields.

2.1 Optimization of oxidative oxo-arylfluorination of alkynes

Supplementary Table 2 Screening of gold catalysts and solvent[a]

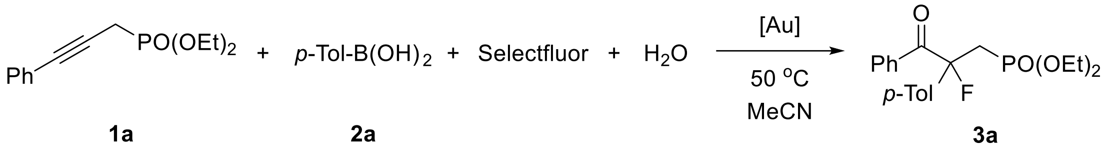

entry	gold catalyst	solvent	yield (%)[b]
1	$(4\text{-}CF_3Ph)_3PAuCl$ (10 mol%)	MeCN	70
2	DMSAuCl (10 mol%)	MeCN	55
3	$PNP(AuCl)_2$ (5 mol%)	MeCN	32
4	Me-DelphosAuCl (10 mol%)	MeCN	36
5	Au-I (10 mol%)	MeCN	64
6	Au-II (10 mol%)	MeCN	60
7	Au-III (5 mol%)	MeCN	60
8	Au-IV (5 mol%)	MeCN	53
9	Au-V (5 mol%)	MeCN	< 5
10	Au-VI (10 mol%)	MeCN	64
11	-	MeCN	0
12	$(4\text{-}CF_3Ph)_3PAuCl$ (10 mol%)	toluene	0
13	$(4\text{-}CF_3Ph)_3PAuCl$ (10 mol%)	DMF	< 5

[a]Standard reaction conditions: gold catalyst, **1a** (0.1 mmol), **2a** (0.3 mmol), Selectflour (0.4 mmol), H_2O (0.2 mmol), solvent (2.0 mL), the reactions are carried out at 50 °C under air atmosphere for 12 h; [b]Isolated yields.

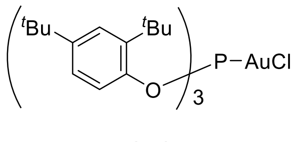

Au-I

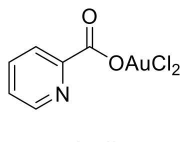

Au-II

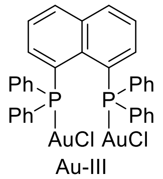

Au-III

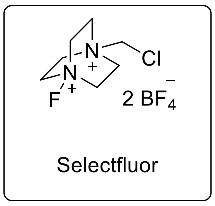

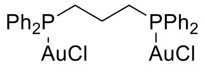

Au-IV

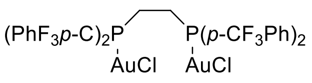

Au-V

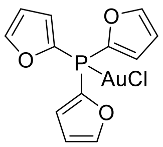

Au-VI

Supplementary Table 3. Screening of reaction temperature[a]

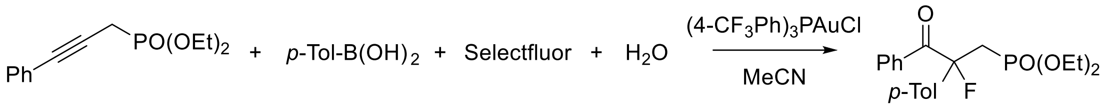

1a 2a 3a

entry	temperature (°C)	yield (%)[b]
1	40	65
2	50	70
3	60	61

[a]Standard reaction conditions: (4-CF_3Ph)$_3$PAuCl (10 mol%), **1a** (0.1 mmol), **2a** (0.3 mmol), Selectflour (0.4 mmol), H_2O (0.2 mmol), MeCN (2.0 mL), the reactions are carried out with heat under air atmosphere for 12 h; [b]Isolated yields.

Supplementary Table 4. Screening of [F]-reagent[a]

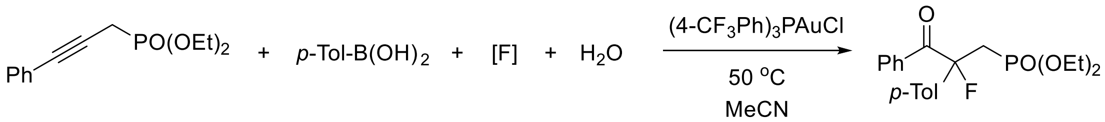

1a 2a 3a

entry	[F]-reagent	yield (%)[b]
1	[F]-I (4.0 equiv)	55
2	[F]-II (4.0 equiv)	0
3	NFSI (4.0 equiv)	0
4	Selectfluor (3.0 equiv)	56
5	Selectfluor (4.0 equiv)	70
6	Selectfluor (5.0 equiv)	64

[a]Standard reaction conditions: (4-CF_3Ph)$_3$PAuCl (10 mol%), **1a** (0.1 mmol), **2a** (0.3 mmol), [F]-reagent, H_2O (0.2 mmol), MeCN (2.0 mL), the reactions are carried out at 50 °C under air atmosphere for 12 h; [b]Isolated yields.

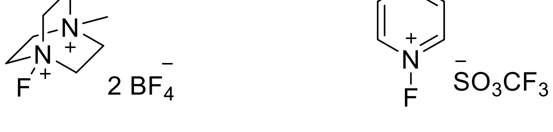

[F]-I [F]-II

Supplementary Table 5. Screening of the amount of boronic acid[a]

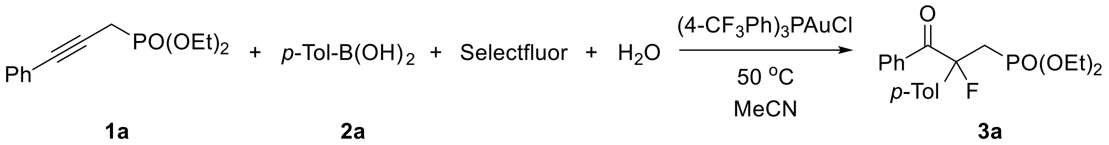

entry	boronic acid (equiv)	yield (%)[b]
1	2.0	47
2	3.0	70
3	4.0	66

[a]Standard reaction conditions: (4-CF_3Ph)$_3$PAuCl (10 mol%), **1a** (0.1 mmol), **2a**, Selectflour (0.4 mmol), H_2O (0.2 mmol), MeCN (2.0 mL), the reactions are carried out at 50 °C under air atmosphere for 12 h; [b]Isolated yields.

2.2 Optimization of oxo-arylalkenylation of alkynes

Supplementary Table 6. Screening of gold catalysts[a]

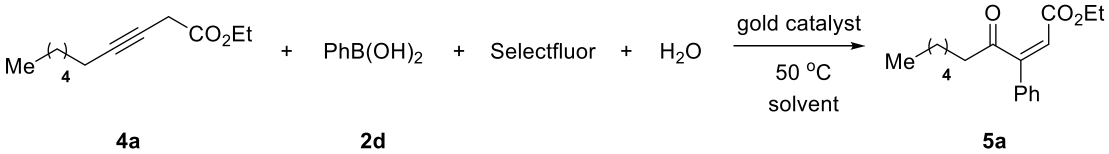

entry	gold catalyst	solvent	yield (%)[b]
1	(4-CF_3Ph)$_3$PAuCl (10 mol%)	MeCN	65
2	(*p*-Tol)$_3$PAuCl (10 mol%)	MeCN	52
3	Me-DelphosAuCl (10 mol%)	MeCN	7
4	PNP(AuCl)$_2$ (5 mol%)	MeCN	43
5	dppm(AuCl)$_2$ (5 mol%)	MeCN	48
6	-	MeCN	0
7	(4-CF_3Ph)$_3$PAuCl (10 mol%)	DMF	7
8	(4-CF_3Ph)$_3$PAuCl (10 mol%)	toluene	0

[a]Standard reaction conditions: gold catalyst, **4a** (0.1 mmol), **2d** (0.3 mmol), Selectflour (0.25 mmol), H_2O (0.2 mmol), solvent (2.0 mL), the reactions are carried out at 50 °C under air atmosphere for 12 h; [b]Isolated yields.

Supplementary Table 7. Screening of reaction temperature[a]

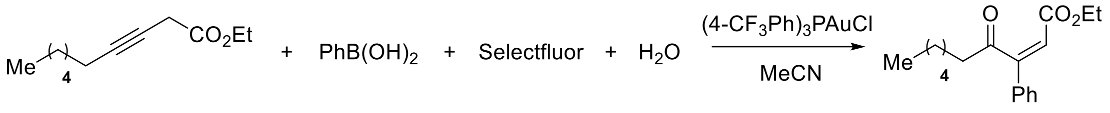

4a **2d** **5a**

entry	temperature (ºC)	yield (%)[b]
1.	40	48
2.	50	65
3.	60	50

[a]Standard reaction conditions: (4-$CF_3Ph)_3PAuCl$ (10 mol%), **4a** (0.1 mmol), **2d** (0.3 mmol), Selectflour (0.25 mmol), H_2O (0.2 mmol), MeCN (2.0 mL), the reactions are carried out with heat under air atmosphere for 12 h; [b]Isolated yields.

Supplementary Table 8. Screening of [F]- reagent[a]

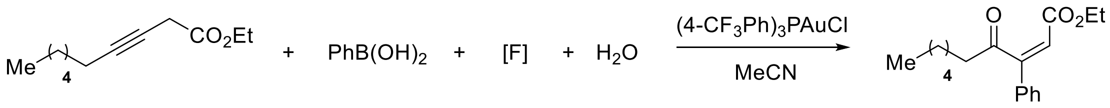

4a **2d** **5a**

entry	[F]- reagent	yield (%)[b]
1	-	0
2	[F]-I (2.5 equiv)	53
3	[F]-II (2.5 equiv)	< 5
4	NFSI (2.5 equiv)	< 5
5	Selectfluor (2.0 equiv)	47
6	Selectfluor (2.5 equiv)	65
7	Selectfluor (3.0 equiv)	60

[a]Standard reaction conditions: (4-$CF_3Ph)_3PAuCl$ (10 mol%), **4a** (0.1 mmol), **2d** (0.3 mmol), [F]-reagent, H_2O (0.2 mmol), MeCN (2.0 mL), the reactions are carried out at 50 ºC under air atmosphere for 12 h; [b]Isolated yields.

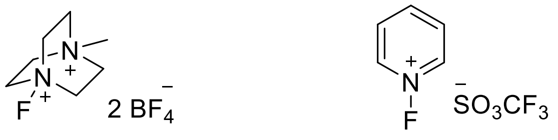

[F]-I [F]-II

Supplementary Table 9. Screening of the amount of boronic acid[a]

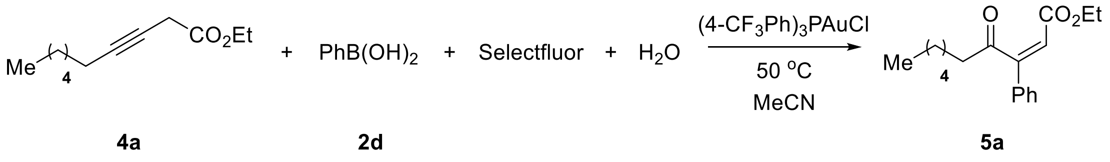

entry	boronic acid (equiv)	yield (%)[b]
1.	2.0	49
2.	3.0	65
3.	4.0	59

[a]Standard reaction conditions: $(4\text{-}CF_3Ph)_3PAuCl$ (10 mol%), **4a** (0.1 mmol), **2d**, Selectflour (0.25 mmol), H_2O (0.2 mmol), MeCN (2.0 mL), the reactions are carried out at 50 °C under air atmosphere for 12 h; [b]Isolated yields.

3. General procedure

GP1: General procedure for oxo-arylfluorination of alkynes

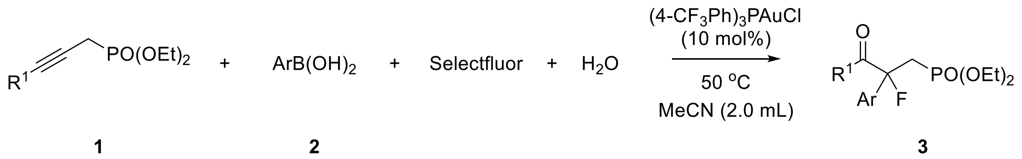

To a dried Schlenk tube, $(4\text{-}CF_3Ph)_3PAuCl$ (0.01mmol, 10 mol%), $ArB(OH)_2$ (0.3 mmol, 3.0 equiv.) and Selectfluor (0.4 mmol, 4.0 equiv.) are successively added under air atmosphere. Then MeCN (2.0 mL) is added into the tube under stirring conditions. After that, alkynes (0.1 mmol, 1.0 equiv.) and water (0.2 mmol, 2.0 euqiv) are added by microinjector under air atmosphere. The resulting reaction mixture is heated at 50 °C for 12 hours. When the reaction is finished (monitored by TLC), the reaction mixture is cooled to room temperature, and then concentrated in vacuo. The resulting residue is

purified by column chromatography on silica gel (eluent: petroleum ether/ethyl acetate) to give the final product **3**.

GP2: General procedure for oxo-arylalkenylation of alkynes

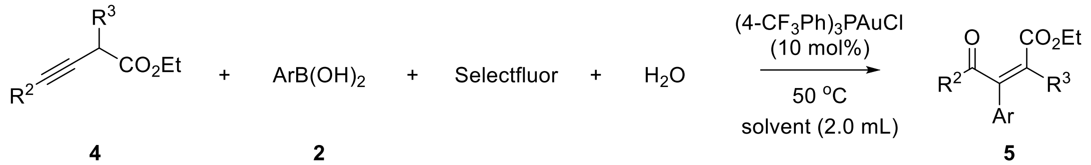

To a dried Schlenk tube, (4-$CF_3Ph)_3PAuCl$ (0.01mmol, 10 mol%), $ArB(OH)_2$ (0.3 mmol, 3.0 equiv.), Selectfluor (0.25 mmol, 2.5 equiv.) and MeCN (2 mL) are added successively under air atmosphere. After that, the corresponding alkynes (0.1 mmol, 1.0 equiv.) and water (0.2 mmol, 2.0 euqiv) are added by microinjector under air atmosphere. The resulting reaction mixture is heated at 50 °C for 12 hours and then cooled to room temperature and concentrated in vacuo. The resulting residue is purified by column chromatography on silica gel (eluent: petroleum ether/ethyl acetate) to give the final product **5**.

4. Characterization data for the products

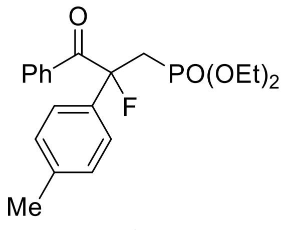

3a

diethyl (2-fluoro-3-oxo-3-phenyl-2-(*p*-tolyl)propyl)phosphonate

Compound **3a** was prepared according to GP1 in 0.1 mmol scale as a colorless oil (25.2 mg, 70%). The flash chromatography was performed with EA/PE (1:2~1:3, v/v) as the eluent.

^{1}H NMR (500 MHz, Chloroform-*d*) δ 7.90 (m, 2H), 7.50 – 7.45 (m, 1H), 7.40 (d, *J* = 8.4 Hz, 2H), 7.35 (m, 2H), 7.19 (d, *J* = 8.0 Hz, 2H), 4.09 – 3.99 (m, 4H), 3.37 – 3.23

(m, 1H), 2.68 – 2.59 (m, 1H), 2.33 (s, 3H), 1.23 (td, *J* = 7.0, 4.1 Hz, 6H);

^{13}C NMR (125 MHz, Chloroform-*d*) δ 196.9 (dd, *J* = 26.7, 4.5 Hz), 138.6, 135.5 (dd, *J* = 22.6, 11.6 Hz), 134.4 (d, *J* = 3.5 Hz), 132.9, 130.1 (d, *J* = 6.3 Hz), 129.6 (d, *J* = 1.7 Hz), 128.1, 123.9 (d, *J* = 8.7 Hz), 100.7 (dd, *J* = 192.6, 7.4 Hz), 61.8 (t, *J* = 5.9 Hz), 37.5 (dd, *J* = 139.6, 25.7 Hz), 21.1, 16.2 (dd, *J* = 6.2, 2.7 Hz);

^{19}F NMR (471 MHz, Chloroform-*d*) δ -157.61 (d, *J* = 4.8 Hz);

^{31}P NMR (202 MHz, Chloroform-*d*) δ 23.50 (d, *J* = 3.9 Hz);

IR (ATR): *v* = 1686, 1251, 1208, 1050, 1022, 959, 901, 830, 701, 688, 593;

HRMS (ESI) Calculated for $C_{20}H_{25}FO_4P$ $[M+H]^+$: 379.1469; found 379.1462.

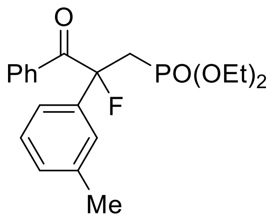

3b

diethyl (2-fluoro-3-oxo-3-phenyl-2-(*m*-tolyl)propyl)phosphonate

Compound **3b** was prepared according to GP1 in 0.1 mmol scale as a colorless oil (22.5 mg, 60%). The flash chromatography was performed with EA/PE (1:2~1:3, v/v) as the eluent.

^{1}H NMR (500 MHz, Chloroform-*d*) δ 7.91 – 7.89 (m, 2H), 7.47 (t, *J* = 7.4 Hz, 1H), 7.35 (t, *J* = 7.9 Hz, 3H), 7.32 – 7.24 (m, 2H), 7.13 (d, *J* = 7.6 Hz, 1H), 4.10 – 3.97 (m, 4H), 3.38 – 3.24 (m, 1H), 2.69 – 2.55 (m, 1H), 2.35 (s, 3H), 1.23 (td, *J* = 7.1, 4.9 Hz, 6H);

^{13}C NMR (125 MHz, Chloroform-*d*) δ 196.8 (dd, *J* = 26.8, 3.8 Hz), 138.7 (d, *J* = 1.7 Hz), 138.4 (dd, *J* = 22.5, 11.6 Hz), 134.4 (d, *J* = 3.4 Hz), 132.9, 130.1 (d, *J* = 6.2 Hz), 129.4, 128.8 (d, *J* = 1.8 Hz), 128.1, 124.5 (d, *J* = 8.6 Hz), 121.0 (d, *J* = 9.3 Hz), 100.6 (dd, *J* = 192.9, 7.0 Hz), 61.8 (t, *J* = 5.9 Hz), 37.7 (dd, *J* = 139.5, 25.6 Hz), 21.5, 16.2 (dd, *J* = 6.3, 3.2 Hz);

^{19}F NMR (471 MHz, Chloroform-*d*) δ -157.83 (d, *J* = 3.9 Hz);

^{31}P NMR (202 MHz, Chloroform-*d*) δ 23.43 (d, *J* = 3.9 Hz);

IR (ATR): *v* = 2925, 1687, 1447, 1251, 1208, 1162, 1097, 1052, 1025, 964, 909, 776, 697;

HRMS (ESI) Calculated for $C_{20}H_{25}FO_4P$ $[M+H]^+$: 379.1469; found 379.1466.

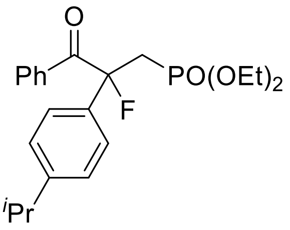

3c

diethyl (2-fluoro-2-(4-isopropylphenyl)-3-oxo-3-phenylpropyl)phosphonate

Compound **3c** was prepared according to GP1 in 0.1 mmol scale as a colorless oil (28.8 mg, 71%). The flash chromatography was performed with EA/PE (1:2~1:3, v/v) as the eluent.

^{1}H NMR (500 MHz, Chloroform-*d*) δ 7.92 – 7.91 (m, 2H), 7.50 – 7.40 (m, 3H), 7.34 (t, *J* = 7.8 Hz, 2H), 7.23 (d, *J* = 8.1 Hz, 2H), 4.09 – 3.94 (m, 4H), 3.37 – 3.23 (m, 1H), 2.90 – 2.85 (m, 1H), 2.69 – 2.60 (m, 1H), 1.25 – 1.17 (m, 12H);

^{13}C NMR (125 MHz, Chloroform-*d*) δ 196.9 (dd, *J* = 27.0, 4.4 Hz), 149.4, 135.7 (dd, *J* = 22.7, 11.7 Hz), 134.4 (d, *J* = 3.5 Hz), 132.8, 130.1 (d, *J* = 6.4 Hz), 128.0, 126.9, 124.0 (d, *J* = 8.9 Hz), 100.7 (dd, *J* = 192.6, 6.9 Hz), 61.7 (t, *J* = 7.0 Hz), 37.5 (dd, *J* = 139.4, 25.5 Hz), 33.7, 23.8 (d, *J* = 2.8 Hz), 16.2 (dd, *J* = 6.3, 2.9 Hz);

^{19}F NMR (471 MHz, Chloroform-*d*) δ -157.54 (d, *J* = 3.9 Hz);

^{31}P NMR (202 MHz, Chloroform-*d*) δ 23.51 (d, *J* = 3.8 Hz);

IR (ATR): *v* = 2960, 2926, 1686, 1510, 1251, 1050, 960, 901, 688;

HRMS (ESI) Calculated for $C_{22}H_{29}FO_4P$ [M+H]+: 407.1782; found 407.1775.

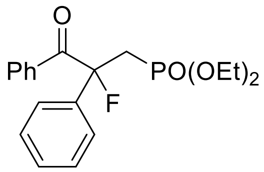

3d

diethyl (2-fluoro-3-oxo-2,3-diphenylpropyl)phosphonate

Compound **3d** was prepared according to GP1 in 0.1 mmol scale as a pale-yellow oil (22.3mg, 61%). The flash chromatography was performed with EA/PE (1:2~1:3, v/v) as the eluent.

^{1}H NMR (500 MHz, Chloroform-*d*) δ 7.91 – 7.89 (m, 2H), 7.55 – 7.52 (m, 2H), 7.49 – 7.46 (m, 1H), 7.41 – 7.30 (m, 5H), 4.09 – 3.98 (m, 4H), 3.38 – 3.25 (m, 1H), 2.71 – 2.62 (m, 1H), 1.23 (td, *J* = 7.1, 4.2 Hz, 6H);

^{13}C NMR (125 MHz, Chloroform-*d*) δ 196.8 (dd, *J* = 26.7, 4.6 Hz), 138.4 (dd, *J* = 22.5, 11.4 Hz), 134.3 (d, *J* = 3.6 Hz), 133.0 , 130.1 (d, *J* = 6.3 Hz), 128.9 (d, *J* = 1.9 Hz), 128.6, 128.1, 124.0 (d, *J* = 9.0 Hz), 100.6 (dd, *J* = 193.1, 7.1 Hz), 61.8 (t, *J* = 6.1 Hz), 37.5 (dd, *J* = 139.9, 25.5 Hz), 16.2 (dd, *J* = 6.3, 2.9 Hz);

^{19}F NMR (471 MHz, Chloroform-*d*) δ -158.10 (d, *J* = 4.6 Hz);

^{31}P NMR (202 MHz, Chloroform-*d*) δ 23.35 (d, *J* = 4.0 Hz);

IR (ATR): *v* = 1686, 1251, 1208, 1050, 1022, 959, 900, 830, 701, 688, 593;

HRMS (ESI) Calculated for $C_{19}H_{23}FO_4P$ $[M+H]^+$: 365.1313; found 365.1309.

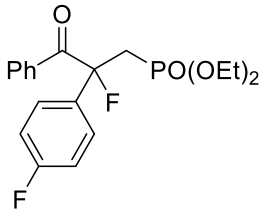

3e

diethyl (2-fluoro-2-(4-fluorophenyl)-3-oxo-3-phenylpropyl)phosphonate

Compound **3e** was prepared according to GP1 in 0.1 mmol scale as a pale-yellow oil (25.2 mg, 66%). The flash chromatography was performed with EA/PE (1:2~1:3, v/v) as the eluent.

^{1}H NMR (500 MHz, Chloroform-*d*) δ 7.89 – 7.87 (m, 2H), 7.53 – 7.46 (m, 3H), 7.37 – 7.34 (m, 2H), 7.10 – 7.05 (m, 2H), 4.09 – 3.98 (m, 4H), 3.33 – 3.20 (m, 1H), 2.70 – 2.61 (m, 1H), 1.22 (td, *J* = 7.1, 3.2 Hz, 6H);

^{13}C NMR (125 MHz, Chloroform-*d*) δ 196.7 (dd, *J* = 26.7, 4.9 Hz), 163.8, 161.8, 134.2 (d, *J* = 3.5 Hz), 133.1, 130.1 (d, *J* = 6.4 Hz), 128.2, 126.1 (t, *J* = 8.8 Hz), 115.9 (dd, *J* = 21.8, 1.3 Hz). 100.4 (dd, *J* = 193.5, 6.6 Hz), 61.9 (dd, *J* = 6.3, 2.8 Hz), 37.5 (dd, *J* =

140.3, 25.5 Hz), 16.2 (dd, *J* = 6.3, 2.4 Hz);

^{19}F NMR (471 MHz, Chloroform-*d*) δ -113.00, -157.08 (d, *J* = 4.2 Hz);

^{31}P NMR (202 MHz, Chloroform-*d*) δ 23.03 (d, *J* = 3.8 Hz);

IR (ATR): *ν* = 2926, 1685, 1531, 1441, 1349, 1243, 1040, 967, 782;

HRMS (ESI) Calculated for $C_{19}H_{22}F_2O_4P$ $[M+H]^+$: 383.1218; found 383.1217.

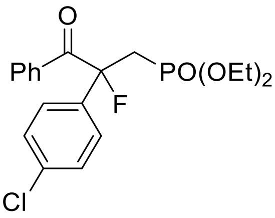

3f

diethyl (2-(4-chlorophenyl)-2-fluoro-3-oxo-3-phenylpropyl)phosphonate

Compound **3f** was prepared according to GP1 in 0.1 mmol scale as a pale-yellow oil (23.8 mg, 60%). The flash chromatography was performed with EA/PE (1:2~1:3, v/v) as the eluent.

^{1}H NMR (500 MHz, Chloroform-*d*) δ 7.89 – 7.86 (m, 2H), 7.51 – 7.45 (m, 3H), 7.39 – 7.34 (m, 4H), 4.08 – 3.98 (m, 4H), 3.32 – 3.19 (m, 1H), 2.69 – 2.60 (m, 1H), 1.22 (td, *J* = 7.1, 3.5 Hz, 6H);

^{13}C NMR (125 MHz, Chloroform-d) δ 196.5 (dd, *J* = 26.6, 5.1 Hz), 136.9 (dd, *J* = 23.0, 11.4 Hz), 134.9 , 134.1 (d, *J* = 3.5 Hz), 133.2 , 130.1 (d, *J* = 6.4 Hz), 129.1 (d, *J* = 1.7 Hz), 128.2 , 125.6 (d, *J* = 9.1 Hz), 100.3 (dd, *J* = 194.0, 6.7 Hz), 61.9 (dd, *J* = 6.3, 2.0 Hz), 37.4 (dd, *J* = 140.5, 25.5 Hz), 16.2 (dd, *J* = 6.2, 2.5 Hz);

^{19}F NMR (471 MHz, Chloroform-*d*) δ -157.89 (d, *J* = 4.2 Hz);

^{31}P NMR (202 MHz, Chloroform-*d*) δ 22.93 (d, *J* = 4.5 Hz);

IR (ATR): *ν* = 1686, 1247, 1094, 1049, 1016, 958, 899, 839, 781, 730, 699, 595, 538;

HRMS (ESI) Calculated for $C_{19}H_{22}ClFO_4P$ $[M+H]^+$: 399.0923; found 399.0921.

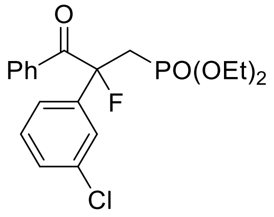

3g

diethyl (2-(3-chlorophenyl)-2-fluoro-3-oxo-3-phenylpropyl)phosphonate

Compound **3g** was prepared according to GP1 in 0.1 mmol scale as a colorless oil (25.0 mg, 62%). The flash chromatography was performed with EA/PE (1:2~1:3, v/v) as the eluent.

^{1}H NMR (500 MHz, Chloroform-*d*) δ 7.89 – 7.87 (m, 2H), 7.57 – 7.52 (m, 1H), 7.53 – 7.45 (m, 1H), 7.43 – 7.33 (m, 3H), 7.35 – 7.27 (m, 2H), 4.10 – 3.96 (m, 4H), 3.33 – 3.19 (m, 1H), 2.68 – 2.59 (m, 1H), 1.22 (td, *J* = 7.1, 5.1 Hz, 6H);

^{13}C NMR (125 MHz, Chloroform-*d*) δ 196.3 (dd, *J* = 26.6, 4.6 Hz), 140.3 (dd, *J* = 22.8, 11.5 Hz), 135.0 (d, *J* = 2.1 Hz), 134.1 (d, *J* = 3.6 Hz), 133.2 , 130.2 (d, *J* = 1.7 Hz), 130.0 (d, *J* = 6.5 Hz), 128.9 , 128.2 , 124.3 (d, *J* = 10.0 Hz), 122.2 (d, *J* = 8.9 Hz), 100.1 (dd, *J* = 194.8, 6.9 Hz), 61.9 (t, *J* = 5.6 Hz), 37.5 (dd, *J* = 140.4, 25.4 Hz), 16.2 (dd, *J* = 6.1, 3.5 Hz);

^{19}F NMR (471 MHz, Chloroform-*d*) δ -158.03 (d, *J* = 3.9 Hz);

^{31}P NMR (202 MHz, Chloroform-*d*) δ 22.78 (d, *J* = 5.0 Hz);

IR (ATR): *v* = 1686, 1248, 1211, 1050, 1023, 983, 963, 792, 691;

HRMS (ESI) Calculated for $C_{19}H_{22}ClFO_4P$ $[M+H]^+$: 399.0923; found 399.0923.

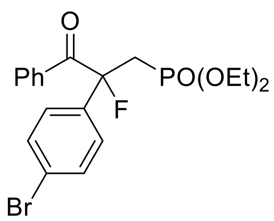

3h

diethyl (2-(4-bromophenyl)-2-fluoro-3-oxo-3-phenylpropyl)phosphonate

Compound **3h** was prepared according to GP1 in 0.1 mmol scale as a pale-yellow oil (32.5 mg, 74%). The flash chromatography was performed with EA/PE (1:2~1:3, v/v)

as the eluent.

^{1}H NMR (500 MHz, Chloroform-*d*) δ 7.88 – 7.86 (m, 2H), 7.54 – 7.47 (m, 3H), 7.43 – 7.39 (m, 2H), 7.38 – 7.34 (m, 2H), 4.10 – 3.97 (m, 4H), 3.32 – 3.18 (m, 1H), 2.69 – 2.60 (m, 1H), 1.22 (td, *J* = 7.1, 3.6 Hz, 6H);

^{13}C NMR (125 MHz, Chloroform-*d*) δ 196. 5 (dd, *J* = 26.5, 4.8 Hz), 137.4 (dd, *J* = 22.8, 11.3 Hz), 134.1 (d, *J* = 3.8 Hz), 133.2, 132.1 (d, *J* = 1.7 Hz), 130.1 (d, *J* = 6.4 Hz), 128.2, 125.8 (d, *J* = 9.1 Hz), 123.0, 100.4 (dd, *J* = 194.1, 6.9 Hz), 61.9 (dd, *J* = 6.3, 1.8 Hz), 37.3 (dd, *J* = 140.5, 25.4 Hz), 16.2 (dd, *J* = 6.2, 2.5 Hz);

^{19}F NMR (471 MHz, Chloroform-*d*) δ -158.10 (d, *J* = 3.9 Hz);

^{31}P NMR (202 MHz, Chloroform-*d*) δ 22.87 (d, *J* = 4.0 Hz);

IR (ATR): *ν* = 1685, 1487, 1395, 1246, 1210, 1048, 1020, 958, 899, 837, 781, 723, 698, 594, 528;

HRMS (ESI) Calculated for $C_{19}H_{22}BrFO_4P$ $[M+H]^+$: 443.0418; found 443.0418.

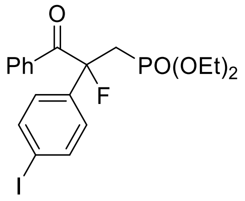

3i

diethyl (2-fluoro-2-(4-iodophenyl)-3-oxo-3-phenylpropyl)phosphonate

Compound **3i** was prepared according to GP1 in 0.1 mmol scale as a yellow oil (26.5 mg, 54%). The flash chromatography was performed with EA/PE (1:2~1:3, v/v) as the eluent.

^{1}H NMR (500 MHz, Chloroform-*d*) δ 7.88 (d, *J* = 8.4 Hz, 2H), 7.73 (d, *J* = 8.4 Hz, 2H), 7.55 – 7.46 (m, 1H), 7.38 – 7.35 (m, 2H), 7.30 – 7.24 (m, 2H), 4.09 – 3.98 (m, 4H), 3.31 – 3.17 (m, 1H), 2.69-2.59 (m, 1H), 1.23 (td, *J* = 7.1, 3.9 Hz, 6H);

^{13}C NMR (125 MHz, Chloroform-*d*) δ 196.5 (dd, *J* = 26.6, 4.9 Hz), 138.1 (dd, *J* = 22.8, 11.3 Hz), 138.0 (d, *J* = 1.9 Hz), 134.1 (d, *J* = 3.7 Hz), 133.2, 130.1 (d, *J* = 6.4 Hz), 128.3, 126.0 (d, *J* = 8.9 Hz), 100.4 (dd, *J* = 194.2, 6.9 Hz), 94.8, 61.9 (dd, *J* = 6.3, 1.8 Hz), 37.3 (dd, *J* = 140.4, 25.5 Hz), 16.3 (dd, *J* = 6.3, 2.7 Hz);

^{19}F NMR (471 MHz, Chloroform-*d*) δ -158.44 (d, *J* = 4.0 Hz);

31P NMR (202 MHz, Chloroform-*d*) δ 22.86 (d, *J* = 3.8 Hz);

IR (ATR): *v* = 2980, 2924, 2853, 1686, 1392, 1250, 1052, 1025, 970, 899, 720;

HRMS (ESI) Calculated for $C_{19}H_{22}FIO_4P$ $[M+H]^+$: 491.0279; found 491.0276.

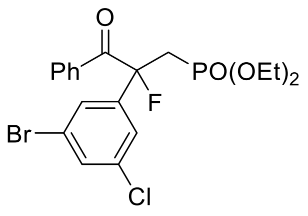

3j

diethyl (2-(3-bromo-5-chlorophenyl)-2-fluoro-3-oxo-3-phenylpropyl)phosphonate

Compound **3j** was prepared according to GP1 in 0.1 mmol scale as a colorless oil (41.9 mg, 88%). The flash chromatography was performed with EA/PE (1:2~1:3, v/v) as the eluent.

1H NMR (500 MHz, Chloroform-*d*) δ 7.90 – 7.86 (m, 2H), 7.58 (t, *J* = 1.7 Hz, 1H), 7.54 – 7.51 (m, 1H), 7.49 – 7.47 (m, 2H), 7.42 – 7.37 (m, 2H), 4.10 – 3.99 (m, 4H), 3.29 – 3.22 (m, 1H), 2.64 – 2.62 (m, 1H), 1.23 (q, *J* = 6.9 Hz, 6H).

13C NMR (125 MHz, Chloroform-*d*) δ 196.0 (dd, *J* = 26.6, 4.7 Hz), 141.9 (dd, *J* = 23.1, 11.5 Hz), 135.9 (d, *J* = 2.3 Hz), 133.9 (d, *J* = 3.8 Hz), 133.5, 131.8, 130.1 (d, *J* = 6.8 Hz), 128.4, 125.6 (d, *J* = 9.7 Hz), 123.4 (d, *J* = 1.9 Hz), 123.3 (d, *J* = 9.8 Hz), 99.6 (dd, *J* = 196.9, 6.6 Hz), 62.0 (dd, *J* = 6.4, 2.9 Hz), 37.6 (dd, *J* = 140.7, 25.2 Hz), 16.3 (dd, *J* = 6.3, 4.0 Hz);

19F NMR (471 MHz, Chloroform-*d*) δ -157.93 (d, *J* = 3.8 Hz);

31P NMR (202 MHz, Chloroform-*d*) δ 22.27 (d, *J* = 3.8 Hz);

IR (ATR): *v* = 1415, 1324, 1265, 1209, 1050, 1022, 973, 863, 760, 724, 701, 686;

HRMS (ESI) Calculated for $C_{19}H_{21}BrClFO_4P$ $[M+H]^+$: 477.0028; found 477.0026.

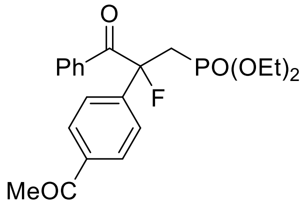

3k

diethyl (2-(4-acetylphenyl)-2-fluoro-3-oxo-3-phenylpropyl)phosphonate

Compound **3k** was prepared according to GP1 in 0.1 mmol scale as a white solid (23.6 mg, 58%). The flash chromatography was performed with EA/PE (1:1~1:2, v/v) as the eluent.

^{1}H NMR (500 MHz, Chloroform-*d*) δ 7.98 (d, *J* = 8.3 Hz, 2H), 7.89 – 7.87 (m, 2H), 7.64 (d, *J* = 8.6 Hz, 2H), 7.53 – 7.45 (m, 1H), 7.36 (t, *J* = 7.8 Hz, 2H), 4.08 – 4.00 (m, 4H), 3.36 – 3.22 (m, 1H), 2.72 – 2.63 (m, 1H), 2.58 (s, 3H), 1.22 (td, *J* = 7.1, 3.5 Hz, 6H);

^{13}C NMR (125 MHz, Chloroform-*d*) δ 197.3, 196.2 (dd, *J* = 26.7, 5.1 Hz), 143.1 (dd, *J* = 22.4, 11.0 Hz), 137.2, 134.1 (d, *J* = 3.5 Hz), 133.3, 130.1 (d, *J* = 6.5 Hz), 128.9 (d, *J* = 2.0 Hz), 128.3, 124.4 (d, *J* = 8.9 Hz), 100.6 (dd, *J* = 194.9, 6.8 Hz), 62.0 (d, *J* = 6.4 Hz), 37.3 (dd, *J* = 140.7, 25.2 Hz), 26.7, 16.3 (dd, *J* = 6.2, 2.6 Hz);

^{19}F NMR (471 MHz, Chloroform-*d*) δ -158.45 (d, *J* = 3.9 Hz);

^{31}P NMR (202 MHz, Chloroform-*d*) δ 22.77 (d, *J* = 3.8 Hz);

IR (ATR): *v* = 2982, 2929, 1606, 1447, 1406, 1360, 1263, 1101, 1018, 957;

HRMS (ESI) Calculated for $C_{21}H_{25}FO_5P$ $[M+H]^+$: 407.1418; found 407.1416.

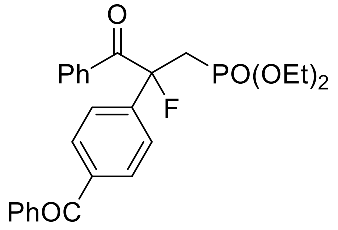

3l

diethyl (2-(4-benzoylphenyl)-2-fluoro-3-oxo-3-phenylpropyl)phosphonate

Compound **3l** was prepared according to GP1 in 0.1 mmol scale as a white solid (21.4 mg, 47%). The flash chromatography was performed with EA/PE (1:1~1:2, v/v) as the eluent.

^{1}H NMR (500 MHz, Chloroform-*d*) δ 7.93 – 7.90 (m, 2H), 7.83 (d, *J* = 8.3 Hz, 2H), 7.80 – 7.74 (m, 2H), 7.67 (d, *J* = 8.6 Hz, 2H), 7.63 – 7.55 (m, 1H), 7.54 – 7.43 (m, 3H), 7.38 (t, *J* = 7.8 Hz, 2H), 4.14 – 3.97 (m, 4H), 3.41 – 3.27 (m, 1H), 2.74 – 2.65 (m, 1H), 1.24 (td, *J* = 7.1, 3.3 Hz, 6H);

^{13}C NMR (125 MHz, Chloroform-*d*) δ 196.3 (dd, *J* = 26.6, 4.7 Hz), 195.8, 142. 5 (dd, *J* = 22.4, 11.4 Hz), 137.8, 137.1, 134.1 (d, *J* = 3.5 Hz), 133.3, 132.7, 130.5 (d, *J* = 2.0 Hz), 130.1 (d, *J* = 6.4 Hz), 130.0, 128.3 (d, *J* = 11.9 Hz), 124.1 (d, *J* = 9.1 Hz), 100.6 (dd, *J* = 194.8, 6.9 Hz), 61.9 (dd, *J* = 6.4, 2.4 Hz), 37.4 (dd, *J* = 140.4, 25.3 Hz), 16.2 (dd, *J* = 6.3, 2.5 Hz);

^{19}F NMR (471 MHz, Chloroform-*d*) δ -158.38 (d, *J* = 3.9 Hz);

^{31}P NMR (202 MHz, Chloroform-*d*) δ 22.79 (d, *J* = 3.8 Hz);

IR (ATR): *v* = 1687, 1659, 1316, 1276, 1051, 1025, 962, 939, 926, 701;

HRMS (ESI) Calculated for $C_{26}H_{27}FO_5P$ $[M+H]^+$: 469.1575; found 469.1573.

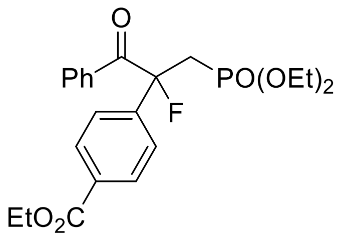

3m

ethyl 4-(3-(diethoxyphosphoryl)-2-fluoro-1-oxo-1-phenylpropan-2-yl)benzoate

Compound **3m** was prepared according to GP1 in 0.1 mmol scale as a pale-yellow oil (24.9 mg, 57%). The flash chromatography was performed with EA/PE (1:1~1:2, v/v) as the eluent.

^{1}H NMR (500 MHz, Chloroform-*d*) δ 8.06 (d, *J* = 8.3 Hz, 2H), 7.88 – 7.85 (m, 2H), 7.61 (d, *J* = 8.6 Hz, 2H), 7.47 (t, *J* = 7.4 Hz, 1H), 7.38 – 7.30 (m, 2H), 4.35 (q, *J* = 7.1 Hz, 2H), 4.09 – 3.97 (m, 4H), 3.36 – 3.22 (m, 1H), 2.71 – 2.61 (m, 1H), 1.36 (t, *J* = 7.1 Hz, 3H), 1.22 (td, *J* = 7.1, 4.8 Hz, 6H);

^{13}C NMR (125 MHz, Chloroform-*d*) δ 196.2 (dd, *J* = 26.6, 5.1 Hz), 165.8, 142.8 (dd, *J* = 22.4, 11.3 Hz), 134.0 (d, *J* = 3.6 Hz), 133.2, 130.8, 130.1 (d, *J* = 1.9 Hz), 130.0 (d, *J* = 6.3 Hz), 128.2, 124.1 (d, *J* = 9.1 Hz), 100.5 (dd, *J* = 194.5, 6.8 Hz), 61.9 (dd, *J* = 6.4, 2.7 Hz), 61.1, 37.3 (dd, *J* = 140.7, 25.2 Hz), 16.2 (dd, *J* = 6.3, 3.0 Hz), 14.2;

^{19}F NMR (471 MHz, Chloroform-*d*) δ -158.37 (d, *J* = 4.6 Hz);

^{31}P NMR (202 MHz, Chloroform-*d*) δ 22.83 (d, *J* = 3.8 Hz);

IR (ATR): *v* = 2349, 1716, 1271, 1104, 1049, 1019, 959, 719, 697;

HRMS (ESI) Calculated for $C_{22}H_{27}FO_6P$ $[M+H]^+$: 437.1524; found 437.1522.

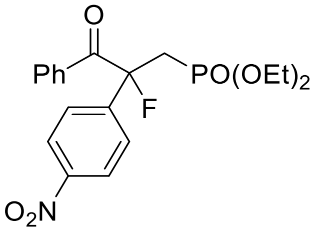

3n

diethyl (2-fluoro-2-(4-nitrophenyl)-3-oxo-3-phenylpropyl)phosphonate

Compound **3n** was prepared according to GP1 in 0.1 mmol scale as a pale-yellow solid (22.0 mg, 54%). Mp. = 79 – 81 °C. The flash chromatography was performed with EA/PE (1:1~1:2, v/v) as the eluent.

^{1}H NMR (500 MHz, Chloroform-*d*) δ 8.29 – 8.23 (m, 2H), 7.89 – 7.87 (m, 2H), 7.78 – 7.72 (m, 2H), 7.56 – 7.48 (m, 1H), 7.42 – 7.35 (m, 2H), 4.10 – 3.99 (m, 4H), 3.34 – 3.21 (m, 1H), 2.76 – 2.65 (m, 1H), 1.23 (td, *J* = 7.1, 1.4 Hz, 6H);

^{13}C NMR (125 MHz, Chloroform-*d*) δ 195.8 (dd, *J* = 26.2, 5.7 Hz), 148.0, 144.9 (dd, *J* = 22.6, 10.6 Hz), 133.7 (d, *J* = 3.6 Hz), 133.6, 130.0 (d, *J* = 6.7 Hz), 128.4, 125.3 (d, *J* = 9.3 Hz), 124.7, 124.0, 118.8, 100.4 (dd, *J* = 196.3, 6.6 Hz), 62.0 (dd, *J* = 6.3, 3.7 Hz), 37.2 (dd, *J* = 141.4, 25.1 Hz), 16.1 (dd, *J* = 6.2, 1.3 Hz);

^{19}F NMR (471 MHz, Chloroform-*d*) δ -158.21 (d, *J* = 3.8 Hz);

^{31}P NMR (202 MHz, Chloroform-d) δ 22.18 (d, *J* = 4.8 Hz);

IR (ATR): *v* = 2923, 2854, 1687, 1524, 1348, 1247, 1050, 1024, 962, 854, 719, 693;

HRMS (ESI) Calculated for $C_{19}H_{22}FNO_6P$ $[M+H]^+$: 410.1163; found 410.1161.

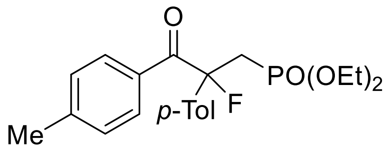

3o

diethyl (2-fluoro-3-oxo-2,3-di-*p*-tolylpropyl)phosphonate

Compound **3o** was prepared according to GP1 in 0.1 mmol scale as a pale-yellow oil (24.2 mg, 62%). The flash chromatography was performed with EA/PE (1:1~1:2, v/v) as the eluent.

¹H NMR (500 MHz, Chloroform-*d*) δ 7.84 – 7.82 (m, 2H), 7.39 (d, *J* = 8.3 Hz, 2H), 7.18 (d, *J* = 8.0 Hz, 2H), 7.14 (d, *J* = 8.0 Hz, 2H), 4.09 – 3.97 (m, 4H), 3.35 – 3.21 (m, 1H), 2.68 – 2.59 (m, 1H), 2.34 (s, 3H), 2.32 (s, 3H), 1.22 (td, *J* = 7.1, 4.8 Hz, 6H);

¹³C NMR (125 MHz, Chloroform-*d*) δ 196.2 (dd, *J* = 26.4, 4.7 Hz), 143.8, 138.4 , 135.7 (dd, *J* = 22.7, 11.3 Hz), 131.7 (d, *J* = 3.6 Hz), 130.3 (d, *J* = 6.4 Hz), 129.5, 128.8, 123.9 (d, *J* = 8.7 Hz), 101.6 (dd, *J* = 191.1, 6.8 Hz), 61.8 (dd, *J* = 6.3, 3.9 Hz), 37.4 (dd, *J* = 139.8, 25.9 Hz), 21.6, 21.1, 16.3 (dd, *J* = 6.1, 2.1 Hz);

¹⁹F NMR (471 MHz, Chloroform-*d*) δ -157.37 (d, *J* = 3.9 Hz);

³¹P NMR (202 MHz, Chloroform-*d*) δ 23.62;

IR (ATR): *v* = 2923, 2853, 1684, 1607, 1457, 1243, 1025, 965, 819;

HRMS (ESI) Calculated for $C_{21}H_{27}FO_4P$ $[M+H]^+$: 393.1626; found 393.1624.

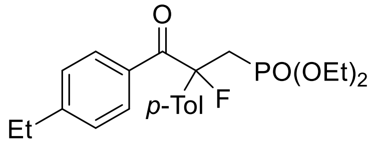

3p

diethyl (3-(4-ethylphenyl)-2-fluoro-3-oxo-2-(*p*-tolyl)propyl)phosphonate

Compound **3p** was prepared according to GP1 in 0.1 mmol scale as a pale-yellow oil (25.6 mg, 63%). The flash chromatography was performed with EA/PE (1:1~1:2, v/v) as the eluent.

¹H NMR (500 MHz, Chloroform-*d*) δ 7.86 – 7.84 (m, 2H), 7.44 – 7.35 (m, 2H), 7.19 – 7.16 (m, 4H), 4.07 – 3.97 (m, 4H), 3.35 – 3.21 (m, 1H), 2.76 – 2.47 (m, 3H), 2.32 (s, 3H), 1.25 – 1.17 (m, 9H);

¹³C NMR (125 MHz, Chloroform-*d*) δ 196.2 (d, *J* = 21.9 Hz), 150.0, 138.5, 135.7 (dd, *J* = 22.6, 11.2 Hz), 131.9 (d, *J* = 3.6 Hz), 130.5 (d, *J* = 6.3 Hz), 129.5, 127.7, 123.9 (d, *J* = 8.7 Hz), 100.8 (dd, *J* = 192.5, 7.1 Hz), 61.8 (t, *J* = 5.3 Hz), 37.5 (dd, *J* = 139.7, 25.8 Hz), 28.9, 21.1, 16.3 (dd, *J* = 6.2, 3.0 Hz), 15.1;

¹⁹F NMR (471 MHz, Chloroform-*d*) δ -157.39 (d, *J* = 3.9 Hz);

³¹P NMR (202 MHz, Chloroform-*d*) δ 23.63;

IR (ATR): *v* = 2924, 1683, 1607, 1255, 1054, 1025, 926, 827;

HRMS (ESI) Calculated for $C_{22}H_{29}FO_4P$ $[M+H]^+$: 407.1782; found 407.1781.

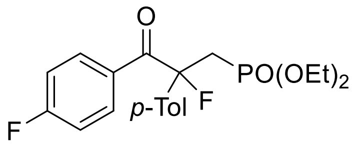

3q

diethyl (2-fluoro-3-(4-fluorophenyl)-3-oxo-2-(*p*-tolyl)propyl)phosphonate

Compound **3q** was prepared according to GP1 in 0.1 mmol scale as a pale-yellow oil (25.1 mg, 63%). The flash chromatography was performed with EA/PE (1:1~1:2, v/v) as the eluent.

^{1}H NMR (500 MHz, Chloroform-*d*) δ 7.98 – 7.94 (m, 2H), 7.37 (d, *J* = 8.4 Hz, 2H), 7.19 (d, *J* = 8.0 Hz, 2H), 7.02 (t, *J* = 8.7 Hz, 2H), 4.07 – 4.00 (m, 4H), 3.36 – 3.22 (m, 1H), 2.66 – 2.57 (m, 1H), 2.33 (s, 3H), 1.23 (t, *J* = 7.1 Hz, 6H);

^{13}C NMR (125 MHz, Chloroform-*d*) δ 195.3 (dd, *J* = 26.6, 4.3 Hz), 166.5, 164.5, 138.7, 135.3 (dd, *J* = 22.8, 11.8 Hz), 132.9 (dd, *J* = 9.3, 6.9 Hz), 130.7 (d, *J* = 3.4 Hz), 129.6, 123.8 (d, *J* = 8.8 Hz), 115.3 (d, *J* = 21.9 Hz), 100.8 (dd, *J* = 192.3, 7.1 Hz), 61.8 (t, *J* = 6.1 Hz), 37.5 (dd, *J* = 139.7, 25.7 Hz), 21.1, 16.3 (dd, *J* = 6.3, 1.4 Hz);

^{19}F NMR (471 MHz, Chloroform-*d*) δ -104.81, -157.16 (d, *J* = 4.7 Hz);

^{31}P NMR (202 MHz, Chloroform-*d*) δ 23.42;

IR (ATR): *v* = 1600, 1507, 1252, 1220, 1157, 1094, 1018, 962, 836, 774, 548, 531;

HRMS (ESI) Calculated for $C_{20}H_{24}F_2O_4P$ $[M+H]^+$: 397.1375; found 397.1375.

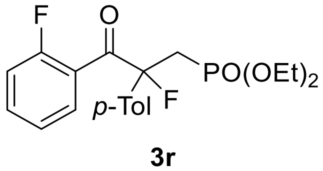

3r

diethyl (2-fluoro-3-(2-fluorophenyl)-3-oxo-2-(*p*-tolyl)propyl)phosphonate

Compound **3r** was prepared according to GP1 in 0.1 mmol scale as a pale-yellow oil (15.4 mg, 39%). The flash chromatography was performed with EA/PE (1:1~1:2, v/v) as the eluent.

^{1}H NMR (500 MHz, Chloroform-*d*) δ 7.66 – 7.62 (m, 1H), 7.46 – 7.35 (m, 3H), 7.20

(d, *J* = 8.0 Hz, 2H), 7.15 – 7.12 (m, 1H), 7.03 – 6.99 (m, 1H), 4.10 – 4.00 (m, 4H), 3.34 – 3.20 (m, 1H), 2.65 – 2.57 (m, 1H), 2.35 (s, 3H), 1.26 (td, *J* = 7.0, 4.3 Hz, 6H);

^{13}C NMR (125 MHz, Chloroform-*d*) δ 197.7 (dd, *J* = 31.0, 2.4 Hz), 161.2, 159.1, 138.5, 134.6 (dd, *J* = 23.1, 12.1 Hz), 133.5 (d, *J* = 8.4 Hz), 131.4 (dd, *J* = 5.2, 2.6 Hz), 129.4 (d, *J* = 1.8 Hz), 124.5 (dd, *J* = 14.2, 2.6 Hz), 124.2 (d, *J* = 9.1 Hz), 123.7 (d, *J* = 3.7 Hz), 116.1 (d, *J* = 22.0 Hz), 99.8 (dd, *J* = 193.1, 6.9 Hz), 61.9 (dd, *J* = 18.9, 6.2 Hz), 37.9 (dd, *J* = 138.9, 25.3 Hz), 21.1, 16.3 (dd, *J* = 6.2, 2.1 Hz);

^{19}F NMR (471 MHz, Chloroform-*d*) δ -109.44 (d, *J* = 15.2 Hz), -161.41 (dd, *J* = 15.2, 4.7 Hz);

^{31}P NMR (202 MHz, Chloroform-*d*) δ 23.10 (d, *J* = 3.6 Hz);

IR (ATR): *v* = 1697, 1610, 1450, 1250, 1206, 1098, 1050, 1021, 959, 904, 829, 761;

HRMS (ESI) Calculated for $C_{20}H_{24}F_2O_4P$ $[M+H]^+$: 397.1375; found 397.1374.

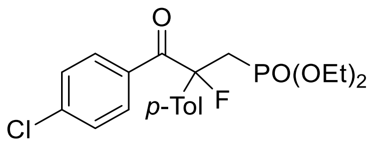

3s

diethyl (3-(4-chlorophenyl)-2-fluoro-3-oxo-2-(*p*-tolyl)propyl)phosphonate

Compound **3s** was prepared according to GP1 in 0.1 mmol scale as a pale-yellow oil (25.1 mg, 61%). The flash chromatography was performed with EA/PE (1:1~1:2, v/v) as the eluent.

^{1}H NMR (500 MHz, Chloroform-*d*) δ 7.86 – 7.84 (m, 2H), 7.36 (d, *J* = 8.3 Hz, 2H), 7.31 (d, *J* = 8.6 Hz, 2H), 7.18 (d, *J* = 8.0 Hz, 2H), 4.11 – 3.96 (m, 4H), 3.35 – 3.21 (m, 1H), 2.65 – 2.57 (m, 1H), 2.32 (s, 3H), 1.30 – 1.14 (m, 6H).

^{13}C NMR (125 MHz, Chloroform-*d*) δ 195.7 (dd, *J* = 26.7, 4.1 Hz), 139.4, 138.7, 135.1 (dd, *J* = 22.6, 11.9 Hz), 132.7 (d, *J* = 3.5 Hz), 131.5 (d, *J* = 6.8 Hz), 129.6, 128.4, 123.8 (d, *J* = 8.8 Hz), 100.7 (dd, *J* = 192.0, 7.0 Hz), 61.8 (t, *J* = 7.2 Hz) 37.5 (dd, *J* = 139.5, 25.6 Hz), 21.0, 16.2 (d, *J* = 6.3 Hz);

^{19}F NMR (471 MHz, Chloroform-*d*) δ -157.46 (d, *J* = 3.9 Hz);

^{31}P NMR (202 MHz, Chloroform-*d*) δ 23.37;

IR (ATR): *v* = 1687, 1588, 1250, 1092, 1050, 1022, 960, 903, 824, 790, 594;

HRMS (ESI) Calculated for $C_{20}H_{24}ClFO_4P$ $[M+H]^+$: 413.1079; found 413.1077.

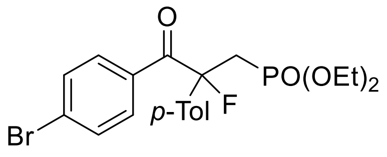

3t

diethyl (3-(4-bromophenyl)-2-fluoro-3-oxo-2-(*p*-tolyl)propyl)phosphonate

Compound **3t** was prepared according to GP1 in 0.1 mmol scale as a yellow oil (29.1 mg, 64%). The flash chromatography was performed with EA/PE (1:1~1:3, v/v) as the eluent.

^{1}H NMR (500 MHz, Chloroform-*d*) δ 7.78 – 7.76 (m, 2H), 7.49 (d, *J* = 8.6 Hz, 2H), 7.36 (d, *J* = 8.4 Hz, 2H), 7.19 (d, *J* = 8.0 Hz, 2H), 4.09 – 3.99 (m, 4H), 3.35 – 3.21 (m, 1H), 2.66 – 2.57 (m, 1H), 2.33 (s, 3H), 1.24 (td, *J* = 7.1, 1.7 Hz, 6H);

^{13}C NMR (125 MHz, Chloroform-*d*) δ 196.0 (dd, *J* = 26.8, 4.3 Hz), 138.7, 135.1 (dd, *J* = 22.7, 11.8 Hz), 133.1 (d, *J* = 3.6 Hz), 131.6 (d, *J* = 6.8 Hz), 131.4, 129.6, 128.2, 123.8 (d, *J* = 8.7 Hz), 100.7 (dd, *J* = 192.0, 7.1 Hz), 61.8 (dd, *J* = 8.6, 6.3 Hz), 37.5 (dd, *J* = 139.5, 25.6 Hz), 21.0, 16.2 (d, *J* = 6.3 Hz);

^{19}F NMR (471 MHz, Chloroform-*d*) δ -157.50 (d, *J* = 3.9 Hz);

^{31}P NMR (202 MHz, Chloroform-*d*) δ 23.33;

IR (ATR): *v* = 1690, 1584, 1395, 1251, 1209, 1052, 1024, 962, 902, 823;

HRMS (ESI) Calculated for $C_{20}H_{24}BrFO_4P$ $[M+H]^+$: 457.0574; found 457.0571.

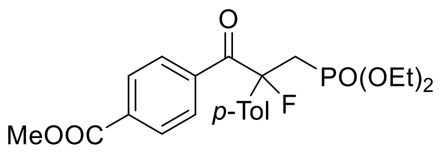

3u

methyl 4-(3-(diethoxyphosphoryl)-2-fluoro-2-(*p*-tolyl)propanoyl)benzoate

Compound **3u** was prepared according to GP1 in 0.1 mmol scale as a yellow oil (29.2 mg, 67%). The flash chromatography was performed with EA/PE (1:1~1:3, v/v) as the eluent.

¹H NMR (500 MHz, Chloroform-*d*) δ 8.00 (d, *J* = 8.5 Hz, 2H), 7.92 – 7.90 (m, 2H), 7.38 (d, *J* = 8.3 Hz, 2H), 7.20 (d, *J* = 8.0 Hz, 2H), 4.10 – 3.98 (m, 4H), 3.90 (s, 3H), 3.37 – 3.23 (m, 1H), 2.66 – 2.58 (m, 1H), 2.33 (s, 3H), 1.24 (td, *J* = 7.1, 2.1 Hz, 6H);

¹³C NMR (125 MHz, Chloroform-*d*) δ 197.0 (dd, *J* = 27.6, 4.0 Hz), 166.2, 138.8, 138.2 (d, *J* = 3.4 Hz), 135.0 (dd, *J* = 22.9, 11.9 Hz), 133.4, 129.8 (d, *J* = 6.1 Hz), 129.7 (d, *J* = 1.2 Hz), 129.2, 123.9 (d, *J* = 8.8 Hz), 100.7 (dd, *J* = 192.2, 7.1 Hz), 61.9 (dd, *J* = 9.5, 6.3 Hz), 52.4, 37.6 (dd, *J* = 139.3, 25.4 Hz), 21.0, 16.3 (d, *J* = 6.2 Hz);

¹⁹F NMR (471 MHz, Chloroform-*d*) δ -158.06 (d, *J* = 3.9 Hz);

³¹P NMR (202 MHz, Chloroform-*d*) δ 23.27;

IR (ATR): *v* = 1725, 1692, 1277, 1250, 1106, 1048, 1019, 959, 905, 829, 776, 595;

HRMS (ESI) Calculated for $C_{22}H_{27}FO_6P$ $[M+H]^+$: 437.1524; found 437.1516.

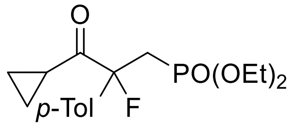

3v

diethyl (3-cyclopropyl-2-fluoro-3-oxo-2-(*p*-tolyl)propyl)phosphonate

Compound **3v** was prepared according to GP1 in 0.1 mmol scale as a pale-yellow oil (16.0 mg, 47%). The flash chromatography was performed with EA/PE (1:3~1:5, v/v) as the eluent.

¹H NMR (500 MHz, Chloroform-*d*) δ 7.30 (d, *J* = 8.4 Hz, 2H), 7.17 (d, *J* = 8.0 Hz, 2H), 4.13 – 3.97 (m, 4H), 3.17 – 3.03 (m, 1H), 2.60 – 2.51 (m, 1H), 2.48 – 2.42 (m, 1H), 2.33 (s, 3H), 1.27 (td, *J* = 7.0, 3.1 Hz, 6H), 1.19 – 1.13 (m, 1H), 1.06 – 0.99 (m, 1H), 0.93 – 0.87 (m, 1H), 0.86 – 0.80 (m, 1H);

¹³C NMR (125 MHz, Chloroform-*d*) δ 207.1 (dd, *J* = 28.3, 4.6 Hz), 138.5, 135.1 (dd, *J* = 23.2, 11.4 Hz), 129.3 (d, *J* = 1.7 Hz), 124.2 (d, *J* = 9.3 Hz), 99.6 (dd, *J* = 190.5, 7.1 Hz), 61.8 (dd, *J* = 15.0, 6.3 Hz), 35.3 (dd, *J* = 140.5, 24.4 Hz), 21.1, 16.3 (d, *J* = 6.2 Hz), 15.4 (d, *J* = 3.4 Hz), 12.9, 12.0;

¹⁹F NMR (471 MHz, Chloroform-*d*) δ -162.13 (d, *J* = 4.8 Hz);

³¹P NMR (202 MHz, Chloroform-*d*) δ 23.62;

IR (ATR): *v* = 1707, 1381, 1253, 1074, 1050, 1023, 988, 959, 937, 824, 542;

HRMS (ESI) Calculated for $C_{17}H_{25}FO_4P$ $[M+H]^+$: 343.1469; found 343.1462.

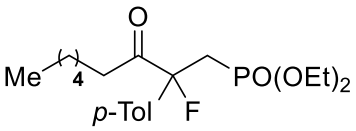

3w

diethyl (2-fluoro-3-oxo-2-(*p*-tolyl)nonyl)phosphonate

Compound **3w** was prepared according to GP1 in 0.1 mmol scale as a pale-yellow oil (25.4 mg, 66%). The flash chromatography was performed with EA/PE (1:3~1:5, v/v) as the eluent.

^{1}H NMR (500 MHz, Chloroform-*d*) δ 7.28 (d, *J* = 8.3 Hz, 2H), 7.15 (d, *J* = 8.0 Hz, 2H), 4.11 – 4.00 (m, 4H), 3.18 – 3.04 (m, 1H), 2.82 – 2.75 (m, 1H), 2.58 – 2.42 (m, 2H), 2.32 (s, 3H), 1.56 – 1.37 (m, 2H), 1.27 (td, *J* = 7.1, 4.7 Hz, 6H), 1.23 – 1.12 (m, 6H), 0.80 (t, *J* = 7.0 Hz, 3H);

^{13}C NMR (125 MHz, Chloroform-*d*) δ 207.9 (dd, *J* = 28.7, 3.8 Hz), 138.4, 134.8 (dd, *J* = 22.9, 12.5 Hz), 129.3 (d, *J* = 1.8 Hz), 123.9 (d, *J* = 9.8 Hz), 99.6 (dd, *J* = 190.3, 7.1 Hz), 77.0 (d, *J* = 31.9), 61.8 (dd, *J* = 35.3, 6.3 Hz), 36.5, 35.8 (dd, *J* = 139.8, 24.2 Hz), 31.4, 28.5, 22.7 (d, *J* = 2.1 Hz), 22.4, 21.0, 16.2 (dd, *J* = 6.3, 1.7 Hz), 13.9;

^{19}F NMR (471 MHz, Chloroform-*d*) δ -163.83 (d, *J* = 4.6 Hz);

^{31}P NMR (202 MHz, Chloroform-*d*) δ 23.78 (d, *J* = 5.0 Hz);

IR (ATR): *v* = 2956, 2928, 1727, 1393, 1253, 1052, 1024, 961, 826, 549;

HRMS (ESI) Calculated for $C_{20}H_{33}FO_4P$ $[M+H]^+$: 387.2095; found 387.2089.

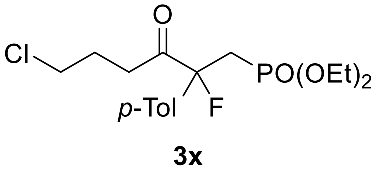

3x

diethyl (6-chloro-2-fluoro-3-oxo-2-(*p*-tolyl)hexyl)phosphonate

Compound **3x** was prepared according to GP1 in 0.1 mmol scale as a yellow oil (21.9 mg, 58%). The flash chromatography was performed with EA/PE (1:3~1:5, v/v) as the

eluent.

^{1}H NMR (500 MHz, Chloroform-*d*) δ 7.30 (d, *J* = 8.4 Hz, 2H), 7.18 (d, *J* = 8.0 Hz, 2H), 4.17 – 3.98 (m, 4H), 3.52 – 3.43 (m, 2H), 3.26 – 2.99 (m, 2H), 2.76 – 2.68 (m, 1H), 2.52 – 2.44 (m, 1H), 2.34 (s, 3H), 2.10 – 1.89 (m, 2H), 1.30 (td, *J* = 7.1, 1.5 Hz, 6H);

^{13}C NMR (125 MHz, Chloroform-*d*) δ 207.1 (dd, *J* = 29.1, 3.1 Hz), 138.7, 134.6 (dd, *J* = 22.9, 13.0 Hz), 129. 4 (d, *J* = 1.8 Hz), 124.0 (d, = 9.7 Hz), 99.7 (dd, *J* = 190.2, 6.9 Hz), 61.9 (dd, *J* = 42.4, 6.2 Hz), 44.1, 36.0 (dd, *J* = 139.7, 24.2 Hz), 33.8, 25.9 (d, *J* = 2.4 Hz), 21.0, 16.3 (dd, *J* = 6.0, 4.5 Hz);

^{19}F NMR (471 MHz, Chloroform-*d*) δ -163.28 (d, *J* = 5.4 Hz);

^{31}P NMR (202 MHz, Chloroform-*d*) δ 23.64 (d, *J* = 5.0 Hz);

IR (ATR): *v* = 2958, 2925, 2854, 1727, 1393, 1250, 1087, 1053, 1025, 962, 823;

HRMS (ESI) Calculated for $C_{17}H_{26}ClFO_4P$ $[M+H]^+$: 379.1236; found 379.1234.

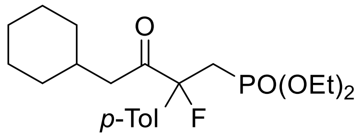

3y

diethyl (4-cyclohexyl-2-fluoro-3-oxo-2-(*p*-tolyl)butyl)phosphonate

Compound **3y** was prepared according to GP1 in 0.1 mmol scale as a yellow oil (22.5 mg, 56%). The flash chromatography was performed with EA/PE (1:3~1:5, v/v) as the eluent.

^{1}H NMR (500 MHz, Chloroform-*d*) δ 7.27 (d, *J* = 8.4 Hz, 2H), 7.15 (d, *J* = 8.0 Hz, 2H), 4.12 – 4.00 (m, 4H), 3.17-3.03 (m, 1H), 2.65 – 2.61 (m, 1H), 2.54 – 2.40 (m, 2H), 2.32 (s, 3H), 1.83 – 1.71 (m, 1H), 1.70 – 1.49 (m, 4H), 1.45 – 1.35 (m, 1H), 1.27 (td, *J* = 7.1, 5.2 Hz, 6H), 1.21 – 1.11 (m, 2H), 1.10 – 1.00 (m, 1H), 0.94 – 0.80 (m, 1H), 0.67 – 0.51 (m, 1H);

^{13}C NMR (125 MHz, Chloroform-*d*) δ 207.0 (dd, *J* = 28.4, 4.0 Hz), 138.4, 134.6 (dd, *J* = 22.9, 12.4 Hz), 129.3 (d, *J* = 1.7 Hz), 124.0 (d, *J* = 9.8 Hz), 99.6 (dd, *J* = 190.7, 6.9 Hz), 61.8 (dd, *J* = 31.1, 6.2 Hz), 43.9, 35.6 (dd, *J* = 140.1, 24.3 Hz), 32.9 (d, *J* = 53.7 Hz), 32.3 (d, *J* = 1.7 Hz), 26.2, 26.0 (d, *J* = 14.7 Hz), 21.0, 16.3 (d, *J* = 6.2 Hz);

^{19}**F NMR** (471 MHz, Chloroform-*d*) δ -163.94 (d, *J* = 4.8 Hz);

^{31}**P NMR** (202 MHz, Chloroform-*d*) δ 23.83;

IR (ATR): *v* = 2923, 2852, 1726, 1254, 1100, 1049, 1024, 995, 960, 822, 549;

HRMS (ESI) Calculated for $C_{21}H_{33}FO_4P$ $[M+H]^+$: 399.2095; found 399.2092.

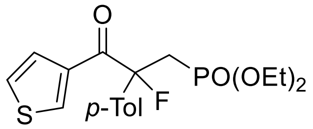

3z

diethyl (2-fluoro-3-oxo-3-(thiophen-3-yl)-2-(*p*-tolyl)propyl)phosphonate

Compound **3z** was prepared according to GP1 n 0.1 mmol scale as a yellow oil (14.5 mg, 41%). The flash chromatography was performed with EA/PE (1:3~1:5, v/v) as the eluent.

^{1}**H NMR** (500 MHz, Chloroform-*d*) δ 8.29 – 8.28 (m, 1H), 7.60 – 7.58 (m, 1H), 7.37 (d, *J* = 8.4 Hz, 2H), 7.21 – 7.15 (m, 1H), 7.16 (d, *J* = 8.0 Hz, 2H), 4.06 – 3.98 (m, 4H), 3.35 – 3.21 (m, 1H), 2.70 – 2.61 (m, 1H), 2.30 (s, 3H), 1.20 (td, *J* = 7.3, 1.1 Hz, 6H);

^{13}**C NMR** (125 MHz, Chloroform-*d*) δ 190.2 (dd, *J* = 26.8, 4.8 Hz), 138.5, 137.1 (d, *J* = 4.0 Hz), 135.31 (d, *J* = 14.0 Hz), 135.30 (dd, *J* = 23.0, 11.4 Hz), 129.4, 128.7 (d, *J* = 3.8 Hz), 125.1, 124.0 (d, *J* = 9.2 Hz), 100.5 (dd, *J* = 192.0, 7.0 Hz), 61.8 (t, *J* = 6.7 Hz), 36.6 (dd, *J* = 140.1, 25.0 Hz), 21.0, 16.1 (d, *J* = 6.4 Hz);

^{19}**F NMR** (471 MHz, Chloroform-*d*) δ -158.60 (d, *J* = 4.7 Hz);

^{31}**P NMR** (202 MHz, Chloroform-*d*) δ 23.43;

IR (ATR): *v* = 1673, 1510, 1244, 1049, 1021, 978, 960, 857, 808, 785, 698, 559;

HRMS (ESI) Calculated for $C_{18}H_{23}FO_4PS$ $[M+H]^+$: 385.1033; found 385.1027.

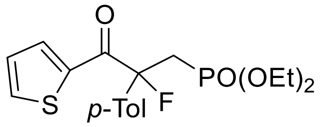

3aa

diethyl (2-fluoro-3-oxo-3-(thiophen-2-yl)-2-(*p*-tolyl)propyl)phosphonate

Compound **3aa** was prepared according to GP1 in 0.1 mmol scale as a yellow oil (14.0

mg, 36%). The flash chromatography was performed with EA/PE (1:3~1:5, v/v) as the eluent.

^{1}H NMR (500 MHz, Chloroform-*d*) δ 7.99 – 7.98 (m, 1H), 7.64 (d, *J* = 4.9 Hz, 1H), 7.40 (d, *J* = 8.1 Hz, 2H), 7.17 (d, *J* = 8.1 Hz, 2H), 7.07 (t, *J* = 4.4 Hz, 1H), 4.11 – 3.94 (m, 4H), 3.38 – 3.24 (m, 1H), 2.72 – 2.63 (m, 1H), 2.31 (s, 3H), 1.21 (t, *J* = 7.0 Hz, 6H);

^{13}C NMR (125 MHz, Chloroform-*d*) δ 189.0 (dd, *J* = 27.4, 4.8 Hz), 139.1 (d, *J* = 5.3 Hz), 138.6, 135.3 (d, *J* = 11.5 Hz), 135.1 (d, *J* = 11.7 Hz), 135.0 (d, *J* = 3.6 Hz), 129.4 , 128.1 (d, *J* = 2.6 Hz), 124.1 (d, *J* = 9.3 Hz), 100.3 (dd, *J* = 192.6, 7.2 Hz), 61.9 (dd, *J* = 11.8, 6.4 Hz), 36.4 (dd, *J* = 140.3, 24.7 Hz), 21.0, 16.2 (dd, *J* = 6.3, 2.0 Hz);

^{19}F NMR (471 MHz, Chloroform-*d*) δ -158.10 (d, *J* = 4.6 Hz);

^{31}P NMR (202 MHz, Chloroform-*d*) δ 23.14 (d, *J* = 4.3 Hz);

IR (ATR): *v* = 1658, 1409, 1239, 1049, 1020, 963, 848, 828, 729, 557;

HRMS (ESI) Calculated for $C_{18}H_{23}FO_4PS$ $[M+H]^+$: 385.1033; found 385.1028.

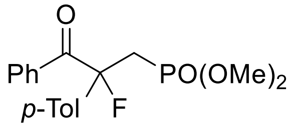

3bb

dimethyl (2-fluoro-3-oxo-3-phenyl-2-(*p*-tolyl)propyl)phosphonate

Compound **3bb** was prepared according to GP1 in 0.1 mmol scale as a yellow oil (11.0 mg, 31%). The flash chromatography was performed with EA/PE (1:3~1:5, v/v) as the eluent.

^{1}H NMR (500 MHz, Chloroform-*d*) δ 7.89 – 7.87 (m, 2H), 7.49 – 7.44 (m, 1H), 7.42 – 7.38 (m, 2H), 7.34 (t, *J* = 7.8 Hz, 2H), 7.20 (d, *J* = 8.0 Hz, 2H), 3.67 (d, *J* = 1.6 Hz, 3H), 3.65 (d, *J* = 1.6 Hz, 3H), 3.38 – 3.24 (m, 1H), 2.67 – 2.59 (m, 1H), 2.32 (s, 3H);

^{13}C NMR (125 MHz, Chloroform-*d*) δ 196.9 (dd, *J* = 26.7, 4.1 Hz), 138.6, 135.3 (dd, *J* = 22.6, 12.1 Hz), 134.3 (d, *J* = 3.5 Hz), 132.9, 130.0 (d, *J* = 6.2 Hz), 129.6 (d, *J* = 1.7 Hz), 128.1, 123.8 (d, *J* = 8.8 Hz), 100.6 (dd, *J* = 192.3, 7.0 Hz), 52.4 (dd, *J* = 9.9, 6.3 Hz), 36.8 (dd, *J* = 139.6, 25.8 Hz), 21.0;

^{19}F NMR (471 MHz, Chloroform-*d*) δ -157.18 (d, *J* = 3.3 Hz);

^{31}P NMR (202 MHz, Chloroform-*d*) δ 26.34 (d, *J* = 3.7 Hz);

IR (ATR): *v* = 1686, 1255, 1208, 1184, 1026, 967, 903, 830, 786, 701, 593;

HRMS (ESI) Calculated for $C_{18}H_{21}FO_4P$ $[M+H]^+$: 351.1156; found 351.1152.

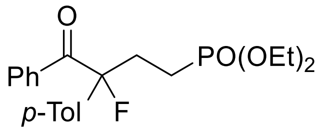

3cc

diethyl (3-fluoro-4-oxo-4-phenyl-3-(*p*-tolyl)butyl)phosphonate

Compound **3cc** was prepared according to GP1 in 0.1 mmol scale as a yellow oil (11.8 mg, 30%). The flash chromatography was performed with EA/PE (1:3~1:5, v/v) as the eluent.

^{1}H NMR (500 MHz, Chloroform-*d*) δ 7.88 – 7.86 (m, *J* = 8.5, 1.6 Hz, 2H), 7.50 – 7.45 (m, 1H), 7.37 – 7.31 (m, 4H), 7.20 (d, *J* = 8.0 Hz, 2H), 4.10 – 4.01 (m, 4H), 2.71 – 2.55 (m, 1H), 2.52 – 2.36 (m, 1H), 2.33 (s, 3H), 1.88 – 1.80 (m, 1H), 1.72 – 1.68 (m, 1H), 1.30 (q, *J* = 6.9 Hz, 6H);

^{13}C NMR (125 MHz, Chloroform-*d*) δ 197.3 (d, *J* = 27.7 Hz), 138.5, 134.7 (d, *J* = 22.1 Hz), 134.5 (d, *J* = 3.7 Hz), 133.1, 130.1 (d, *J* = 5.9 Hz), 129.6 (d, *J* = 1.5 Hz), 128.2, 124.0 (d, *J* = 8.9 Hz), 102.6 (dd, *J* = 190.3, 18.4 Hz), 61.7 (dd, *J* = 6.4, 2.7 Hz), 32.8 (dd, *J* = 24.0, 3.3 Hz), 32.6 (d, *J* = 3.3 Hz), 21.1, 20.3 (d, *J* = 3.8 Hz), 19.2 (d, *J* = 3.6 Hz), 16.4 (d, *J* = 6.2 Hz);

^{19}F NMR (471 MHz, Chloroform-*d*) δ -163.60;

^{31}P NMR (202 MHz, Chloroform-*d*) δ 31.28;

IR (ATR): *v* = 2980.72, 2925.75, 1737.86, 1682.83, 1447.43, 1243.79, 1163.06, 958.03;

HRMS (ESI) Calculated for $C_{21}H_{27}FO_4P$ $[M+H]^+$: 393.1626; found 393.1620.

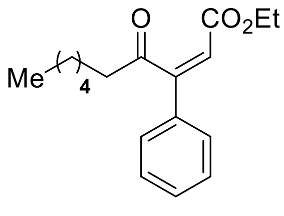

5a

ethyl (*Z*)-4-oxo-3-phenyldec-2-enoate

Compound **5a** was prepared according to GP2 in 0.1 mmol scale as a colorless oil (18.6 mg, 65%). The flash chromatography was performed with EA/PE (1:15~1:20, v/v) as the eluent.

^{1}H NMR (500 MHz, Chloroform-*d*) δ 7.45 – 7.37 (m, 5H), 6.14 (s, 1H), 4.21 (q, J = 7.0 Hz, 2H), 2.70 – 2.62 (m, 2H), 1.72 – 1.66 (m, 2H), 1.34 – 1.23 (m, 9H), 0.86 (t, J = 7.0 Hz, 3H);

^{13}C NMR (125 MHz, Chloroform-*d*) δ 206.6, 165.4, 158.3, 133.3, 130.3, 129.1, 126. 7, 115.8, 60.9, 42.8, 31.6, 28.7, 23.0, 22.5, 14.1, 14.0;

IR (ATR): v = 2929, 1708, 1615, 1369, 1337, 1276, 1262, 1177, 1127, 1077, 1028, 768, 692;

HRMS (ESI) Calculated for $C_{18}H_{25}O_3$ $[M+H]^+$: 289.1798; found 289.1793.

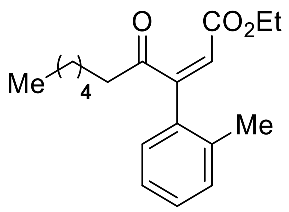

5b

ethyl (*Z*)-4-oxo-3-(*o*-tolyl)dec-2-enoate

Compound **5b** was prepared according to GP2 in 0.1 mmol scale as a colorless oil (16.5 mg, 54%). The flash chromatography was performed with EA/PE (1:15~1:20, v/v) as the eluent.

^{1}H NMR (500 MHz, Chloroform-*d*) δ 7.29 – 7.22 (m, 3H), 7.21 – 7.16 (m, 1H), 5.80 (s, 1H), 4.22 (q, J = 7.0 Hz, 2H), 2.57 (t, J = 7.5 Hz, 2H), 2.40 (s, 3H), 1.62 – 1.57 (m, 2H), 1.36 – 1.16 (m, 9H), 0.84 (t, J = 7.0 Hz, 3H);

^{13}C NMR (125 MHz, Chloroform-*d*) δ 205.2, 165.2, 158.7, 135.7, 134.4, 131.0, 129.0, 128.1, 126.0, 120.6, 61.0, 41.6, 31.5, 28.6, 23.0, 22.4, 20.3, 14.1, 14.0;

IR (ATR): v = 2956, 2926, 1717, 1369, 1328, 1259, 1199, 1180, 1126, 1075, 1029, 763;

HRMS (ESI) Calculated for $C_{19}H_{27}O_3$ $[M+H]^+$: 303.1955; found 303.1952.

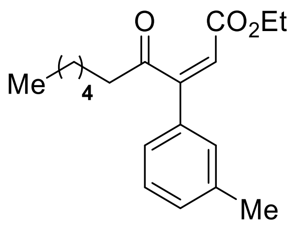

5c

ethyl (*Z*)-4-oxo-3-(*m*-tolyl)dec-2-enoate

Compound **5c** was prepared according to GP2 in 0.1 mmol scale as a colorless oil (17.0 mg, 56%). The flash chromatography was performed with EA/PE (1:15~1:20, v/v) as the eluent.

^{1}H NMR (500 MHz, Chloroform-*d*) δ 7.31 – 7.26 (m, 1H), 7.24 – 7.19 (m, 3H), 6.13 (s, 1H), 4.21 (q, *J* = 7.0 Hz, 2H), 2.65 (d, *J* = 7.6 Hz, 2H), 2.35 (s, 3H), 1.72-1.66 (m, 2H), 1.42 – 1.13 (m, 9H), 0.86 (t, *J* = 7.0 Hz, 3H);

^{13}C NMR (125 MHz, Chloroform-*d*) δ 206.8, 165.4, 158.6, 138.8, 133.2, 131.1, 128.9, 127.3, 123.8, 115.5, 60.8, 42.7, 31.6, 28.7, 23.0, 22.5, 21.4, 14.1, 14.0;

IR (ATR): *v* = 2955. 2927, 1710, 1616, 1368, 1332, 1280, 1260, 1161, 1126, 1080, 1033, 872, 788;

HRMS (ESI) Calculated for $C_{19}H_{27}O_3$ $[M+H]^+$: 303.1955; found 303.1950.

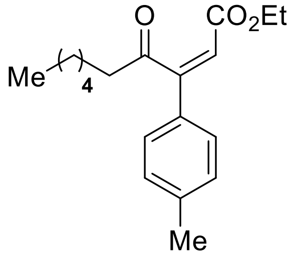

5d

ethyl (*Z*)-4-oxo-3-(*p*-tolyl)dec-2-enoate

Compound **5d** was prepared according to GP2 in 0.1 mmol scale as a colorless oil (16.2 mg, 54%). The flash chromatography was performed with EA/PE (1:15~1:20, v/v) as the eluent.

^{1}H NMR (500 MHz, Chloroform-*d*) δ 7.30 (d, *J* = 8.0 Hz, 2H), 7.19 (d, *J* = 8.0 Hz, 2H), 6.12 (s, 1H), 4.20 (q, *J* = 7.0 Hz, 2H), 2.65 (t, *J* = 7.6 Hz, 2H), 2.37 (s, 3H), 1.74 – 1.65 (m, 2H), 1.32 – 1.24 (m, 9H), 0.86 (t, *J* = 7.0 Hz, 3H);

^{13}C NMR (125 MHz, Chloroform-*d*) δ 206.9, 165.5, 158.3, 140.8, 130.4, 129.8, 126.6, 114.7, 60.8, 42.8, 31.6, 28.7, 23.1, 22.5, 21.3, 14.2, 14.0;

IR (ATR): *v* = 2956, 2925, 2857, 1711, 1605, 1369, 1275, 1176, 1077, 1035, 818, 750;

HRMS (ESI) Calculated for $C_{19}H_{27}O_3$ $[M+H]^+$: 303.1955; found 303.1951.

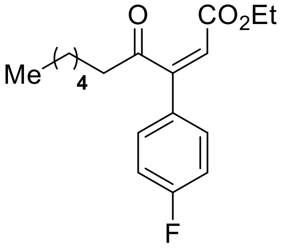

5e

ethyl (*Z*)-3-(4-fluorophenyl)-4-oxodec-2-enoate

Compound **5e** was prepared according to GP2 in 0.1 mmol scale as a colorless oil (18.4 mg, 60%). The flash chromatography was performed with EA/PE (1:15~1:20, v/v) as the eluent.

^{1}H NMR (500 MHz, Chloroform-*d*) δ 7.44 – 7.37 (m, 2H), 7.08 (t, *J* = 8.6 Hz, 2H), 6.09 (s, 1H), 4.21 (q, *J* = 7.0 Hz, 2H), 2.64 (t, *J* = 7.6 Hz, 2H), 1.70 – 1.64 (m, 2H), 1.34 – 1.23 (m, 9H), 0.86 (t, *J* = 7.0 Hz, 3H);

^{13}C NMR (125 MHz, Chloroform-*d*) δ 206.6, 165.2 (d, *J* = 34.3 Hz), 163.0, 157.2, 129.5 (d, *J* = 3.5 Hz), 128.8 (d, *J* = 8.4 Hz), 116.3 (d, *J* = 21.9 Hz), 115.8 (d, *J* = 1.7 Hz), 61.0, 42.8, 31.6, 28.7, 23.1, 22.5, 14.2, 14.0;

^{19}F NMR (471 MHz, Chloroform-*d*) δ -109.66;

IR (ATR): *v* = 2987, 1713, 1509, 1371, 1276, 1182, 1066, 839, 750;

HRMS (ESI) Calculated for $C_{18}H_{24}FO_3$ $[M+H]^+$: 307.1704; found 307.1703.

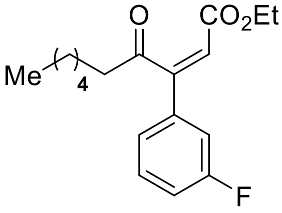

5f

ethyl (*Z*)-3-(3-fluorophenyl)-4-oxodec-2-enoate

Compound **5f** was prepared according to GP2 in 0.1 mmol scale as a colorless oil (20.2 mg, 66%). The flash chromatography was performed with EA/PE (1:15~1:20, v/v) as the eluent.

^{1}H NMR (500 MHz, Chloroform-*d*) δ 7.39 – 7.33 (m, 1H), 7.19 (m, 1H), 7.15 – 7.08 (m, 2H), 6.14 (s, 1H), 4.21 (q, *J* = 7.0 Hz, 2H), 2.74 – 2.55 (m, 2H), 1.76 – 1.60 (m, 2H), 1.36 – 1.20 (m, 9H), 0.85 (t, *J* = 7.0 Hz, 3H);

^{13}C NMR (125 MHz, Chloroform-*d*) δ 206.1, 165.1, 163.9, 161.9, 157.0 (d, *J* = 2.3 Hz), 135.5 (d, *J* = 7.7 Hz), 130.7 (d, *J* = 8.4 Hz), 122.5 (d, *J* = 3.0 Hz), 117.3 (d, *J* = 21.1 Hz), 117.0, 113.6 (d, *J* = 23.0 Hz), 61.1, 42.8, 31.6, 28.7, 23.0, 22.5, 14.1, 14.0;

^{19}F NMR (471 MHz, Chloroform-*d*) δ -111.34;

IR (ATR): *v* = 2929, 1711, 1582, 1370, 1335, 1268, 1202, 1159, 1082, 867, 788;

HRMS (ESI) Calculated for $C_{18}H_{24}FO_3$ $[M+H]^+$: 307.1704; found 307.1703.

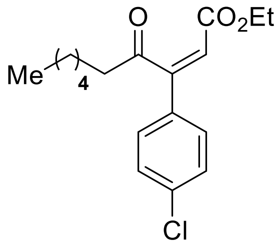

5g

ethyl (*Z*)-3-(4-chlorophenyl)-4-oxodec-2-enoate

Compound **5g** was prepared according to GP2 in 0.1 mmol scale as a colorless oil (17.0 mg, 53%). The flash chromatography was performed with EA/PE (1:15~1:20, v/v) as the eluent.

^{1}H NMR (500 MHz, Chloroform-*d*) δ 7.39 – 7.33 (m, 4H), 6.12 (s, 1H), 4.21 (q, *J* = 7.0 Hz, 2H), 2.68 – 2.57 (m, 2H), 1.70 – 1.64 (m, 2H), 1.33 – 1.21 (m, 9H), 0.86 (t, *J* = 7.0 Hz, 3H);

^{13}C NMR (125 MHz, Chloroform-*d*) δ 206.4, 165.2, 157.0, 136.6, 131.7, 129.4, 128.0, 116.3, 61.0, 42.8, 31.6, 28.7, 23.0, 22.5, 14.1, 14.0;

IR (ATR): *v* = 2956, 2928, 1710, 1492, 1369, 1336, 1268, 1179, 1093, 1031, 1013, 829;

HRMS (ESI) Calculated for $C_{18}H_{24}ClO_3$ $[M+H]^+$: 323.1408; found 323.1402.

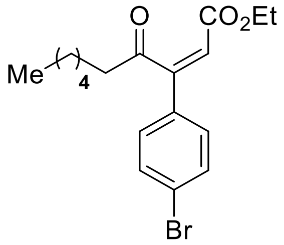

5h

ethyl (*Z*)-3-(4-bromophenyl)-4-oxodec-2-enoate

Compound **5h** was prepared according to GP2 in 0.1 mmol scale as a colorless oil (20.5 mg, 56%). The flash chromatography was performed with EA/PE (1:15~1:20, v/v) as the eluent.

^{1}H NMR (500 MHz, Chloroform-*d*) δ 7.52 (d, *J* = 8.5 Hz, 2H), 7.28 (d, *J* = 8.5 Hz, 2H), 6.12 (s, 1H), 4.21 (q, *J* = 7.0 Hz, 2H), 2.66 – 2.59 (m, 2H), 1.69 – 1.62 (m, 2H), 1.35 – 1.31 – 1.24 (m, 9H), 0.85 (t, *J* = 7.0 Hz, 3H);

^{13}C NMR (125 MHz, Chloroform-*d*) δ 206.2, 165.2, 157.1, 132.3, 132.2, 128.2, 124.9, 116.3, 61.0, 42.8, 31.6, 28.7, 23.0, 22.5, 14.1, 14.0;

IR (ATR): *v* = 2955, 2927, 1711, 1488, 1401, 1181, 1074, 826;

HRMS (ESI) Calculated for $C_{18}H_{24}BrO_3$ $[M+H]^+$: 367.0903; found 367.0902.

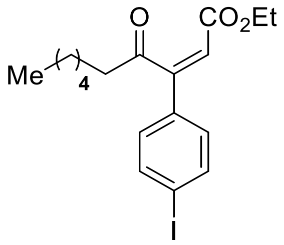

5i

ethyl (*Z*)-3-(4-iodophenyl)-4-oxodec-2-enoate

Compound **5i** was prepared according to GP2 in 0.1 mmol scale as a colorless oil (18.4 mg, 44%). The flash chromatography was performed with EA/PE (1:15~1:20, v/v) as the eluent.

^{1}H NMR (500 MHz, Chloroform-*d*) δ 7.39 – 7.33 (m, 4H), 6.12 (s, 1H), 4.21 (q, *J* = 7.0 Hz, 2H), 2.68 – 2.57 (m, 2H), 1.70 – 1.64 (m, 2H), 1.34 – 1.15 (m, 9H), 0.86 (t, *J* =

7.0 Hz, 3H);

^{13}C NMR (125 MHz, Chloroform-*d*) δ 206.4, 165.2, 157.0, 136.6, 131.7, 129.4, 128.0, 116.3, 61.0, 42.8, 31.6, 28.7, 23.0, 22.5, 14.1, 14.0;

IR (ATR): *v* = 2955, 2927, 1712, 1616, 1484, 1369, 1266, 1181, 1080, 1005, 822;

HRMS (ESI) Calculated for $C_{18}H_{24}IO_3$ $[M+H]^+$: 415.0765; found 415.0760.

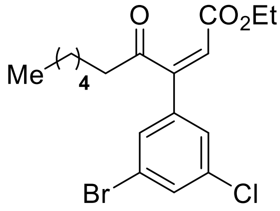

5j

ethyl (*Z*)-3-(3-bromo-5-chlorophenyl)-4-oxodec-2-enoate

Compound **5j** was prepared according to GP2 in 0.1 mmol scale as a colorless oil (22.0 mg, 55%). The flash chromatography was performed with EA/PE (1:15~1:20, v/v) as the eluent.

^{1}H NMR (500 MHz, Chloroform-*d*) δ 7.55 (t, *J* = 1.8 Hz, 1H), 7.45 (t, *J* = 1.8 Hz, 1H), 7.33 (t, *J* = 1.8 Hz, 1H), 6.12 (s, 1H), 4.22 (q, *J* = 7.0 Hz, 2H), 2.62 (t, *J* = 7.5 Hz, 2H), 1.70 – 1.64 (m, 2H), 1.38 – 1.18 (m, 9H), 0.86 (t, J = 7.0 Hz, 3H);

^{13}C NMR (125 MHz, Chloroform-*d*) δ 205.4, 164.7, 155.4, 136.6, 135.9, 132.8, 127.9, 125.5, 123.5, 118.4, 61.3, 42.8, 31.5, 28.6, 23.0, 22.5, 14.1, 14.0;

IR (ATR): *v* = 2956, 2926, 1714, 1554, 1259, 1240, 1184, 1080, 1028, 859;

HRMS (ESI) Calculated for $C_{18}H_{23}BrClO_3$ $[M+H]^+$: 401.0514; found 401.0512.

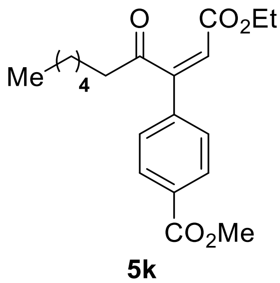

5k

methyl (*Z*)-4-(1-ethoxy-1,4-dioxodec-2-en-3-yl)benzoate

Compound **5k** was prepared according to GP2 in 0.1 mmol scale as a colorless oil (25.9

mg, 72%). The flash chromatography was performed with EA/PE (1:15~1:20, v/v) as the eluent.

^{1}H NMR (500 MHz, Chloroform-*d*) δ 8.05 (d, *J* = 8.5 Hz, 2H), 7.49 (d, *J* = 8.5 Hz, 2H), 6.20 (s, 1H), 4.23 (q, *J* = 7.0 Hz, 2H), 3.93 (s, 3H), 2.65 (t, *J* = 7.5 Hz, 2H), 1.70 – 1.64 (m, 2H), 1.35 – 1.20 (m, 9H), 0.85 (t, *J* = 7.0 Hz, 3H);

^{13}C NMR (125 MHz, Chloroform-*d*) δ 206.1, 166.2, 165.1, 157.2, 137.6, 131.6, 130.2, 126.7, 117.8, 61.1, 52.4, 42.9, 31.6, 28.7, 23.1, 22.5, 14.1, 14.0;

IR (ATR): *v* = 2954, 2924, 1714, 1275, 1182, 1110, 1080, 1019, 775;

HRMS (ESI) Calculated for $C_{20}H_{27}O_5$ $[M+H]^+$: 347.1853; found 347.1852.

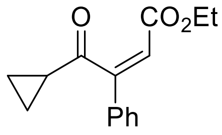

5l

ethyl (*Z*)-4-cyclopropyl-4-oxo-3-phenylbut-2-enoate

Compound **5l** was prepared according to GP2 in 0.1 mmol scale as a colorless oil (13.4 mg, 55%). The flash chromatography was performed with EA/PE (1:15~1:20, v/v) as the eluent.

^{1}H NMR (500 MHz, Chloroform-*d*) δ 7.46 – 7.44 (m, 2H), 7.43 – 7.36 (m, 3H), 6.21 (s, 1H), 4.22 (q, *J* = 7.2 Hz, 2H), 2.17 – 2.12 (m, 1H), 1.35 – 1.27 (m, 5H), 1.06 – 1.02 (m, 2H);

^{13}C NMR (125 MHz, Chloroform-*d*) δ 206.7, 165.2, 157.9, 133.5, 130.3, 129.0 , 127.0, 115.9, 60.9, 22.4, 14.1, 12.6;

IR (ATR): *v* = 2954, 2923, 2853, 1714, 1615, 1448, 1177, 1079, 873, 690;

HRMS (ESI) Calculated for $C_{15}H_{17}O_3$ $[M+H]^+$: 245.1172; found 245.1172.

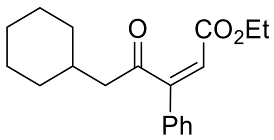

5m

ethyl (*Z*)-5-cyclohexyl-4-oxo-3-phenylpent-2-enoate

Compound **5m** was prepared according to GP2 in 0.1 mmol scale as a colorless oil (20.4 mg, 68%). The flash chromatography was performed with EA/PE (1:15~1:20, v/v) as the eluent.

^{1}H NMR (500 MHz, Chloroform-*d*) δ 7.46 – 7.36 (m, 5H), 6.11 (s, 1H), 4.21 (q, *J* = 7.0 Hz, 2H), 2.56 (d, *J* = 6.4 Hz, 2H), 2.02 – 1.94 (m, 1H), 1.85 – 1.76 (m, 2H), 1.67 – 160 (m, 3H), 1.33 – 1.24 (m, 5H), 1.16 – 1.04 (m, 1H), 0.93 – 0.85 (m, 2H);

^{13}C NMR (125 MHz, Chloroform-*d*) δ 205.5, 165.3, 158.2, 133.4, 130.2, 129.0, 126.7, 115.9, 60.8, 49.8, 33.2, 32.3, 26.3, 26.1, 14.2;

IR (ATR): *v* = 2922, 2851, 1712, 1617, 1447, 1280, 1180, 1028, 768, 693;

HRMS (ESI) Calculated for $C_{19}H_{25}O_3$ $[M+H]^+$: 301.1798; found 301.1796.

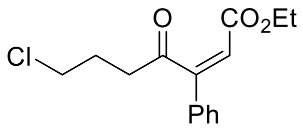

5n

ethyl (*Z*)-7-chloro-4-oxo-3-phenylhept-2-enoate

Compound **5n** was prepared according to GP2 in 0.1 mmol scale as a colorless oil (17.4 mg, 62%). The flash chromatography was performed with EA/PE (1:15~1:20, v/v) as the eluent.

^{1}H NMR (500 MHz, Chloroform-*d*) δ 7.43 – 7.39 (m, 5H), 6.18 (s, 1H), 4.22 (q, *J* = 7.0 Hz, 2H), 3.66 (t, *J* = 6.4 Hz, 2H), 2.84 (t, *J* = 7.0 Hz, 2H), 2.25 – 2.19 (m, 2H), 1.31 (t, *J* = 7.5 Hz, 3H);

^{13}C NMR (125 MHz, Chloroform-*d*) δ 205.3, 165.5, 157.9, 132.9, 130.6, 129.2, 126.7, 116.1, 61.1, 44.4, 39.7, 26.2, 14.2;

IR (ATR): *v* = 2923, 1708, 1370, 1337, 1265, 1180, 1094, 1026, 769, 693;

HRMS (ESI) Calculated for $C_{15}H_{18}ClO_3$ $[M+H]^+$: 281.0939; found 281.0937.

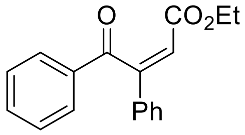

5o

ethyl (*Z*)-4-oxo-3,4-diphenylbut-2-enoate

Compound **5o** was prepared according to GP2 in 0.1 mmol scale as a white solid (14.4 mg, 51%). Mp. = 56 – 58 °C. The flash chromatography was performed with EA/PE (1:15~1:20, v/v) as the eluent.

^{1}H NMR (500 MHz, Chloroform-*d*) δ 7.96 (d, *J* = 7.0 Hz, 2H), 7.54 (d, *J* = 7.5 Hz, 1H), 7.50 – 7.48 (m, 2H), 7.45 – 7.42 (m, 2H), 7.39 – 7.35 (m, 3H), 6.50 (s, 1H), 4.07 (q, *J* = 7.1 Hz, 2H), 1.11 (t, *J* = 7.1 Hz, 3H);

^{13}C NMR (125 MHz, Chloroform-*d*) δ 196.4, 165.1, 155.4, 136.0, 134.2, 133.5, 130.4, 129.1, 128.9, 128.7, 126.9, 117.8, 60.9, 13.9;

IR (ATR): *ν* = 1711, 1675, 1448, 1369, 1275, 1216, 1171, 1046, 1019, 770, 688;

HRMS (ESI) Calculated for $C_{18}H_{17}O_3$ [M+H]+: 281.1172; found 281.1169.

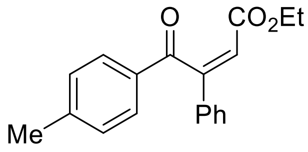

5p

ethyl (*Z*)-4-oxo-3-phenyl-4-(p-tolyl)but-2-enoate

Compound **5p** was prepared according to GP2 in 0.1 mmol scale as a colorless oil (12.0 mg, 41%). Mp. = 94 – 95 °C. The flash chromatography was performed with EA/PE (1:15~1:20, v/v) as the eluent.

^{1}H NMR (500 MHz, Chloroform-*d*) δ 7.86 (d, *J* = 8.2 Hz, 2H), 7.52 – 7.46 (m, 2H), 7.41 – 7.31 (m, 3H), 7.23 (d, *J* = 8.0 Hz, 2H), 6.49 (s, 1H), 4.07 (q, *J* = 7.1 Hz, 2H), 2.38 (s, 3H), 1.12 (t, *J* = 7.1 Hz, 3H);

^{13}C NMR (125 MHz, Chloroform-*d*) δ 196.0, 165.0, 155.6, 144.4, 134.4, 133.7, 130.3, 129.4, 129.0, 126.9, 117.6, 60.8, 21.7, 13.9;

IR (ATR): *ν* = 2922, 1714, 1672, 1607, 1369, 1345, 1278, 1172, 773;

HRMS (ESI) Calculated for $C_{19}H_{19}O_3$ [M+H]+: 295.1329; found 295.1328.

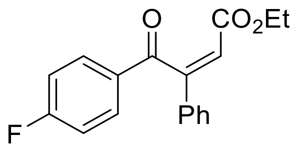

5q

ethyl (*Z*)-4-(4-fluorophenyl)-4-oxo-3-phenylbut-2-enoate

Compound **5q** was prepared according to GP2 in 0.1 mmol scale as a colorless oil (18.1 mg, 61%). Mp. = 90 – 93 °C. The flash chromatography was performed with EA/PE (1:15~1:20, v/v) as the eluent.

^{1}H NMR (500 MHz, Chloroform-*d*) δ 8.01 – 7.95 (m, 2H), 7.50 – 7.46 (m, 2H), 7.42 – 7.34 (m, 3H), 7.11 (t, *J* = 8.6 Hz, 2H), 6.50 (s, 1H), 4.09 (q, *J* = 7.1 Hz, 2H), 1.14 (t, *J* = 7.1 Hz, 3H);

^{13}C NMR (125 MHz, Chloroform-*d*) δ 194.9, 166.9, 165.0 (d, *J* = 14.5 Hz), 155.2, 134.0, 132.6 (d, *J* = 2.8 Hz), 131.6 (d, *J* = 9.3 Hz), 130.6, 129.2, 126.9, 117.9, 116.0 (d, *J* = 22.2 Hz), 61.0, 13.9;

^{19}F NMR (471 MHz, Chloroform-*d*) δ -104.23;

IR (ATR): *v* = 1712, 1675, 1596, 1344, 1277, 1215, 1179, 1151, 847, 774;

HRMS (ESI) Calculated for $C_{18}H_{16}FO_3$ $[M+H]^+$: 299.1078; found 299.1074.

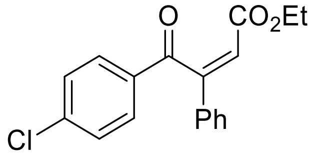

5r

ethyl (*Z*)-4-(4-chlorophenyl)-4-oxo-3-phenylbut-2-enoate

Compound **5r** was prepared according to GP2 in 0.1 mmol scale as a colorless oil (18.4 mg, 59%). Mp. = 113 – 115 °C. The flash chromatography was performed with EA/PE (1:15~1:20, v/v) as the eluent.

^{1}H NMR (500 MHz, Chloroform-*d*) δ 7.89 (d, *J* = 8.5 Hz, 2H), 7.49 – 7.45 (m, 2H), 7.43 – 7.34 (m, 5H), 6.50 (s, 1H), 4.09 (q, *J* = 7.1 Hz, 2H), 1.15 (t, *J* = 7.1 Hz, 3H);

^{13}C NMR (125 MHz, Chloroform-*d*) δ 195.2, 165.0, 155.0, 140.0, 134.5, 133.9, 130.6, 130.2, 129.2, 129.1, 126.9, 118.0, 61.0, 13.9;

IR (ATR): *ν* = 2923, 2852, 1714, 1678, 1589, 1344, 1279, 1216, 1183, 1092, 766;

HRMS (ESI) Calculated for $C_{18}H_{16}ClO_3$ $[M+H]^+$: 315.0782; found 315.0780.

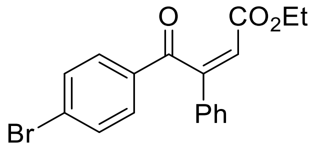

5s

ethyl (*Z*)-4-(4-bromophenyl)-4-oxo-3-phenylbut-2-enoate

Compound **5s** was prepared according to GP2 in 0.1 mmol scale as a colorless oil (15.5 mg, 43%). Mp. = 99 – 101 °C. The flash chromatography was performed with EA/PE (1:15~1:20, v/v) as the eluent.

^{1}H NMR (500 MHz, Chloroform-*d*) δ 7.81 (d, *J* = 8.5 Hz, 2H), 7.58 (d, *J* = 8.5 Hz, 2H), 7.49 – 7.43 (m, 2H), 7.42 – 7.34 (m, 3H), 6.50 (s, 1H), 4.09 (q, *J* = 7.1 Hz, 2H), 1.16 (t, *J* = 7.1 Hz, 3H);

^{13}C NMR (125 MHz, Chloroform-*d*) δ 195.4, 165.0, 155.0, 134.9, 133.9, 132.1, 130.6, 130.3, 129.2, 128.8, 126.9, 118.0, 61.0, 13.9;

IR (ATR): *ν* = 1713, 1677, 1585, 1397, 1369, 1279, 1213, 1182, 1069, 1010;

HRMS (ESI) Calculated for $C_{18}H_{16}BrO_3$ $[M+H]^+$: 359.0277; found 359.0277.

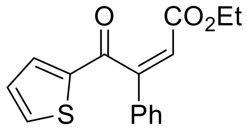

5t

ethyl (*Z*)-4-oxo-3-phenyl-4-(thiophen-2-yl)but-2-enoate

Compound **5t** was prepared according to GP2 in 0.1 mmol scale as a colorless oil (9.5 mg, 34%). Mp. = 69 – 71 °C. The flash chromatography was performed with EA/PE (1:15~1:20, v/v) as the eluent.

^{1}H NMR (500 MHz, Chloroform-*d*) δ 7.67 (dd, *J* = 4.9, 1.2 Hz, 1H), 7.57 – 7.51 (m, 3H), 7.43 – 7.34 (m, 3H), 7.06 (m, 1H), 6.46 (s, 1H), 4.11 (q, *J* = 7.1 Hz, 2H), 1.15 (t, *J* = 7.1 Hz, 3H);

^{13}C NMR (125 MHz, Chloroform-*d*) δ 188.4, 164.8, 154.4, 143.7, 134.5, 134.2, 133.9,

130.4, 129.1, 128.2, 126.9, 118.2, 61.0, 13.9;

IR (ATR): *v* = 2955, 2922, 2853, 1713, 1650, 1462, 1411, 1369, 1280, 1178;

HRMS (ESI) Calculated for $C_{16}H_{15}O_3S$ $[M+H]^+$: 287.0736; found 287.0735.

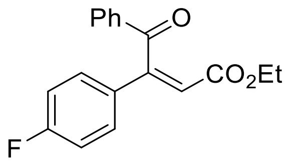

5u

ethyl (*Z*)-3-(4-fluorophenyl)-4-oxo-4-phenylbut-2-enoate

Compound **5u** was prepared according to GP2 in 0.1 mmol scale as a colorless solid (19.5 mg, 65%). Mp. = 42 – 44 °C. The flash chromatography was performed with EA/PE (1:15~1:20, v/v) as the eluent.

^{1}H NMR (500 MHz, Chloroform-*d*) δ 7.97 – 7.91 (m, 2H), 7.59 – 7.52 (m, 1H), 7.52 – 7.41 (m, 4H), 7.05 (t, *J* = 8.6 Hz, 2H), 6.45 (s, 1H), 4.06 (q, *J* = 7.1 Hz, 2H), 1.11 (t, *J* = 7.1 Hz, 3H);

^{13}C NMR (125 MHz, Chloroform-*d*) δ 196.2, 164.9 (d, *J* = 9.1 Hz), 163.0, 154.2, 135.8, 133.6, 130.4 (d, *J* = 3.4 Hz), 129.0 (d, *J* = 8.6 Hz), 128.8 (d, *J* = 11.6 Hz), 117.7 (d, J = 1.9 Hz), 116.4, 116.2, 61.0, 13.8;

^{19}F NMR (471 MHz, Chloroform-*d*) δ -109.40;

IR (ATR): *v* = 2983, 1710, 1674, 1598, 1508, 1450, 1369, 1278, 1216, 1163, 1046;

HRMS (ESI) Calculated for $C_{18}H_{16}FO_3$ $[M+H]^+$: 299.1078; found 299.1071.

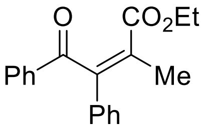

5v

ethyl (*Z*)-2-methyl-4-oxo-3,4-diphenylbut-2-enoate

Compound **5v** was prepared according to GP2 in 0.1 mmol scale as a colorless oil (13.0 mg, 45%). The flash chromatography was performed with EA/PE (1:15~1:20, v/v) as the eluent.

^{1}H NMR (500 MHz, Chloroform-*d*) δ 7.98 – 7.92 (m, 2H), 7.53 – 7.47 (m, 1H), 7.44 – 7.33 (m, 6H), 7.32 – 7.27 (m, 1H), 4.01 (q, *J* = 7.1 Hz, 2H), 2.06 (s, 3H), 1.00 (t, *J* = 7.1 Hz, 3H);

^{13}C NMR (125 MHz, Chloroform-*d*) δ 196.1, 167.1, 150.2, 135.9, 135.0, 133.0, 129.1, 128.62, 128.58, 128.52, 128.48, 61.3, 15.7, 13.5;

IR (ATR): *v* = 2925, 1711, 1667, 1448, 1261, 1134, 1021, 861, 762, 699;

HRMS (ESI) Calculated for $C_{19}H_{19}O_3$ $[M+H]^+$: 295.1329; found 295.1323.

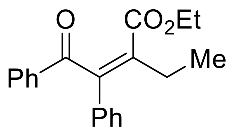

5w

ethyl (*Z*)-2-ethyl-4-oxo-3,4-diphenylbut-2-enoate

Compound **5w** was prepared according to GP2 in 0.1 mmol scale as a colorless oil (12.3 mg, 40%).

^{1}H NMR (500 MHz, Chloroform-*d*) δ 7.99 – 7.92 (m, 2H), 7.53 – 7.46 (m, 1H), 7.42 (dd, *J* = 8.4, 6.9 Hz, 2H), 7.39 – 7.27 (m, 5H), 4.01 (q, *J* = 7.1 Hz, 2H), 2.44 (q, *J* = 7.4 Hz, 2H), 1.15 (t, *J* = 7.4 Hz, 3H), 1.00 (t, *J* = 7.1 Hz, 3H);

^{13}C NMR (125 MHz, Chloroform-*d*) δ 196.0, 166.9, 149.3, 135.8, 135.0, 134.9, 132.9, 129.1, 128.6, 128.5, 128.5, 128.2, 61.1, 22.7, 13.9, 13.5;

IR (ATR): *v* = 2958, 2926, 2857, 1712, 1669, 1448, 1259, 1204, 1021, 761;

HRMS (ESI) Calculated for $C_{20}H_{21}O_3$ $[M+H]^+$: 309.1485; found 309.1479.

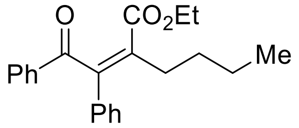

5x

ethyl (*Z*)-2-(2-oxo-1,2-diphenylethylidene)hexanoate

Compound **5x** was prepared according to GP2 in 0.1 mmol scale as a colorless oil (14.8 mg, 44%).

^{1}H NMR (500 MHz, Chloroform-*d*) δ 7.99 – 7.93 (m, 2H), 7.53 – 7.46 (m, 1H), 7.46 – 7.38 (m, 2H), 7.40 – 7.32 (m, 4H), 7.31 – 7.27 (m, 1H), 4.00 (q, *J* = 7.2 Hz, 2H), 2.46 – 2.38 (m, 2H), 1.56 – 1.50 (m, 2H), 1.31 (h, *J* = 7.4 Hz, 2H), 0.99 (t, *J* = 7.1 Hz, 3H), 0.86 (t, *J* = 7.4 Hz, 3H);

^{13}C NMR (125 MHz, Chloroform-*d*) δ 196.1, 167.1, 149.3, 135.9, 135.1, 133.9, 132.9, 129.1, 128.6, 128.5, 128.4, 128.2, 61.1, 31.5, 29.0, 22.6, 13.7, 13.4;

IR (ATR): *v* = 2958, 2926, 2857, 1712, 1669, 1448, 1259, 1204, 1140, 761, 698;

HRMS (ESI) Calculated for $C_{22}H_{25}O_3$ $[M+H]^+$: 337.1798; found 337.1796.

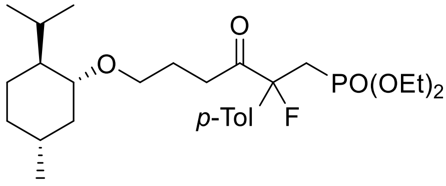

6a

diethyl (2-fluoro-6-(((*1R*,*2S*,*5R*)-2-isopropyl-5-methylcyclohexyl)oxy)-3-oxo-2-(*p*-tolyl)hexyl)phosphonate

Compound **6a** was prepared according to GP1 in 0.1 mmol scale as a colorless oil (20.4mg, 41%). The flash chromatography was performed with EA/PE (1: 3, v/v) as the eluent.

^{1}H NMR (500 MHz, Chloroform-*d*) δ 7.30 – 7.27 (m, 2H), 7.15 (d, *J* = 7.8 Hz, 2H), 4.10 – 4.00 (m, 4H), 3.53 – 3.48 (m, 1H), 3.21 – 3.02 (m, 2H), 2.96 – 2.81 (m, 2H), 2.75 – 2.59 (m, 1H), 2.58 – 2.44 (m, 1H), 2.32 (s, 3H), 2.11 – 2.02 (m, 1H), 2.01 – 1.96 (m, 1H), 1.84 – 1.67 (m, 3H), 1.62 – 1.53 (m, 2H), 1.32 – 1.24 (m, 6H), 1.14 – 1.08 (m, 1H), 0.93 – 0.58 (m, 12H);

^{13}C NMR (125 MHz, Chloroform-*d*) δ 207.7 (ddd, *J* = 28.9, 6.5, 3.7 Hz), 138.4, 134.8 (ddd, *J* = 23.0, 12.6, 2.9 Hz), 129.3 (d, *J* = 2.0 Hz), 124.0 (d, *J* = 9.9 Hz), 100.3 (dd, *J* = 6.9, 3.1 Hz), 98.8 (dd, *J* = 7.0, 3.1 Hz), 79.1, 78.9, 67.2 (d, *J* = 5.1 Hz), 62.0 (d, *J* = 6.3 Hz), 61.7 (d, *J* = 6.3 Hz), 48.2 (d, *J* = 10.1 Hz), 40.3 (d, *J* = 8.0 Hz), 36.3 (t, *J* = 23.4 Hz), 35.2 (t, 23.4 Hz), 34.5, 33.5 (d, *J* = 10.6 Hz), 31.5 (d, *J* = 3.7 Hz), 25.5 (d, *J* = 5.9 Hz), 23.5 (dd, *J* = 4.4, 2.2 Hz), 23.3 (d, *J* = 2.2 Hz), 22.3, 21.0, 20.9 (d, *J* = 2.3 Hz), 16.3 (dd, *J* = 6.2, 1.8 Hz), 16.2, 16.0;

^{19}F NMR (471 MHz, Chloroform-*d*) δ -163.63 (dd, *J* = 19.1, 4.8 Hz);

^{31}P NMR (202 MHz, Chloroform-*d*) δ 23.73 (dd, *J* = 10.4, 4.4 Hz);

IR (ATR): *v* = 2954, 2925, 2868, 1727, 1456, 1392, 1369, 1255, 1091, 1056, 1025, 962;

HRMS (ESI) Calculated for $C_{27}H_{45}FO_5P$ $[M+H]^+$: 499.2983; found 499.2976.

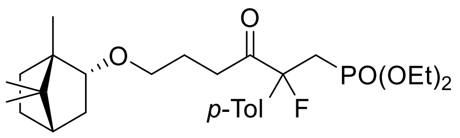

6b

diethyl (2-fluoro-3-oxo-2-(*p*-tolyl)-6-(((*1S*,*2R*,*4S*)-1,7,7-trimethylbicyclo[2.2.1]heptan-2-yl)oxy)hexyl)phosphonate

Compound **6b** was prepared according to GP1 in 0.1 mmol scale as a colorless oil (23.4mg, 47%). The flash chromatography was performed with EA/PE (1: 3, v/v) as the eluent.

^{1}H NMR (500 MHz, Chloroform-*d*) δ 7.30 – 7.26 (m, 2H), 7.15 (d, *J* = 8.0 Hz, 2H), 4.16 – 3.94 (m, 4H), 3.40 – 3.03 (m, 4H), 2.92 – 2.84 (m, 1H), 2.71 – 2.63 (m, 1H), 2.52 – 2.44 (m, 1H), 2.31 (s, 3H), 2.06 – 1.58 (m, 6H), 1.51 – 1.50 (m, 1H), 1.29 – 1.26 (m, 6H), 1.18 – 1.01 (m, 2H), 0.92 – 0.88 (dd, *J* = 13.0, 3.3 Hz, 0.5H), 0.79 – 0.77 (m, 7H), 0.71 (s, 1H), 0.70 – 0.66 (m, 0.5H);

^{13}C NMR (125 MHz, Chloroform-*d*) δ 207.8 (dt, *J* = 28.8, 3.5 Hz), 138.4, 134.8 (dd, *J* = 23.0, 12.4 Hz), 129.3 (d, *J* = 2.0 Hz), 124.0 (d, *J* = 9.8 Hz), 99.6 (ddd, *J* = 190.6, 7.0, 2.8 Hz), 84.4 (d, *J* = 2.8 Hz), 68.4 (d, *J* = 2.6 Hz), 62.0 (d, *J* = 6.3 Hz), 61.7 (d, *J* = 6.3 Hz), 49.0 (d, *J* = 2.3 Hz), 47.6 (d, *J* = 2.6 Hz), 44.9 (d, *J* = 3.5 Hz), 36.3 (d, *J* = 24.3 Hz), 36.1 (d, *J* = 6.9 Hz), 35.2 (d, *J* = 24.4 Hz), 33.3 (d, *J* = 13.0 Hz), 28.1 (d, *J* = 15.7 Hz), 26.5 (d, *J* = 2.8 Hz), 23.4 (dd, *J* = 6.3, 2.0 Hz), 21.0, 19.7, 18.7, 16.26 (dd, *J* = 6.3, 1.7 Hz), 13.9 (d, *J* = 10.9 Hz);

^{19}F NMR (471 MHz, Chloroform-*d*) δ -163.81 (dd, *J* = 118.3, 4.8 Hz);

^{31}P NMR (202 MHz, Chloroform-*d*) δ 23.71 (d, *J* = 4.9 Hz);

IR (ATR): *v* = 2981, 2948, 2873, 1727, 1453, 1389, 1254, 1164, 1095, 1054, 989;

HRMS (ESI) Calculated for $C_{27}H_{43}FO_5P$ $[M+H]^+$: 497.2827; found 497.2827.

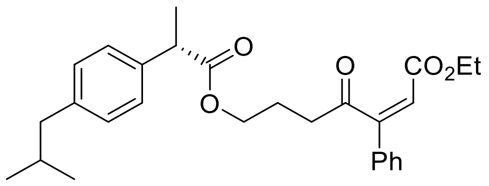

6c

ethyl (*S*, *Z*)-7-((2-(4-isobutylphenyl)propanoyl)oxy)-4-oxo-3-phenylhept-2-enoate

Compound **6c** was prepared according to GP2 in 0.1 mmol scale as a colorless oil (19.5mg, 43%). The flash chromatography was performed with EA/PE (1: 10, v/v) as the eluent.

^{1}H NMR (500 MHz, Chloroform-*d*) δ 7.45 – 7.36 (m, 5H), 7.12 (d, *J* = 8.1 Hz, 2H), 7.01 (d, *J* = 8.1 Hz, 2H), 6.16 (s, 1H), 4.20 (q, *J* = 7.2 Hz, 2H), 4.15 – 4.11 (m, 2H), 3.63 (q, *J* = 7.2 Hz, 1H), 2.64 – 2.60 (m, 2H), 2.41 (d, *J* = 7.2 Hz, 2H), 2.06 – 2.00 (m, *J* = 7.0 Hz, 2H), 1.84 – 1.79 (m, 1H), 1.44 (d, *J* = 7.2 Hz, 3H), 1.29 (t, *J* = 7.2 Hz, 3H), 0.89 (s, 3H), 0.87 (s, 3H).

^{13}C NMR (125 MHz, Chloroform-*d*) δ 205.4, 174.6, 165.4, 157.9, 140.4, 137.7, 132.9, 129.2, 129.1, 127.1, 126.6, 115.9, 63.6, 61.0, 45.1, 45.0, 38.8, 30.1, 22.4, 22.3, 18.3, 14.1;

IR (ATR): *v* = 2955, 2926, 1711, 1615, 1448, 1369, 1272, 1094, 1027, 770;

HRMS (ESI) Calculated for $C_{28}H_{35}O_5$ $[M+H]^+$: 451.2479; found 451.2477.

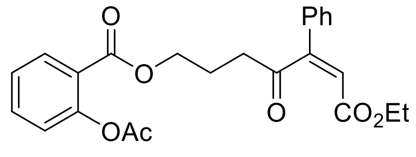

6d

(*Z*)-7-ethoxy-4,7-dioxo-5-phenylhept-5-en-1-yl 2-acetoxybenzoate

Compound **6d** was prepared according to GP2 in 0.1 mmol scale as a colorless oil (18.7mg, 44%). The flash chromatography was performed with EA/PE (1: 5, v/v) as the eluent.

^{1}H NMR (500 MHz, Chloroform-*d*) δ 7.94 – 7.92 (m, 1H), 7.56 – 7.50 (m, 1H), 7.43 – 7.34 (m, 5H), 7.29 – 7.23 (m, 1H), 7.09 – 7.07 (m, 1H), 6.18 (s, 1H), 4.35 (t, *J* = 6.6

Hz, 2H), 4.19 (q, J = 7.1 Hz, 2H), 2.80 (t, J = 7.2 Hz, 2H), 2.32 (s, 3H), 2.20 (t, J = 6.9 Hz, 2H), 1.28 (t, J = 7.1 Hz, 3H);

^{13}C NMR (125 MHz, Chloroform-*d*) δ 205.3, 169.6, 165.4, 164.3, 157.9, 150.6, 133.7, 132.9, 131.6, 130.5, 129.2, 126.6, 125.9, 123.7, 116.1, 64.2, 61.0, 38.9, 22.4, 21.0, 14.1;

IR (ATR): v = 2927, 1768, 1712, 1607, 1450, 1368, 1253.77, 1184.82, 1078.61, 1027.15, 752.06;

HRMS (ESI) Calculated for $C_{24}H_{25}O_7$ $[M+H]^+$: 425.1595; found 425.1591.

5. X-ray structure of products

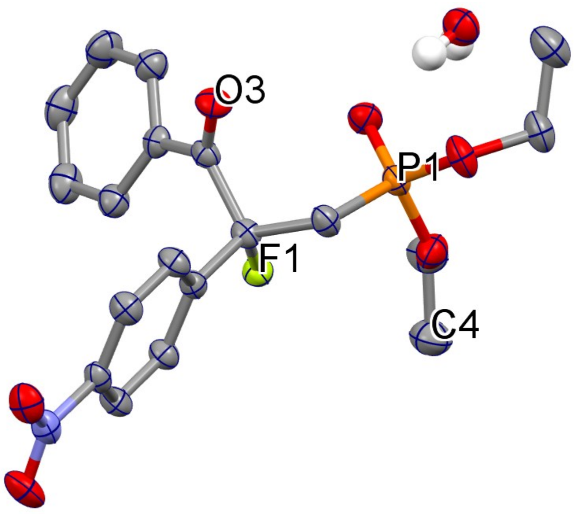

Supplementary Table 10. Crystal data and structure refinement for 3n.
CCDC: 2202498

Identification code	1_a
Empirical formula	$C_{19}H_{23}FNO_7P$
Formula weight	427.35
Temperature/K	296.15
Crystal system	monoclinic
Space group	C2/c
a/Å	39.028(4)
b/Å	10.8883(11)
c/Å	9.8254(10)
α/°	90
β/°	100.829(5)
γ/°	90
Volume/Å^3	4101.0(7)

Z	8
ρ_{calc}g/cm^3	1.384
μ/mm^{-1}	0.184
F(000)	1792.0
Crystal size/mm^3	? × ? × ?
Radiation	MoKα (λ = 0.71073)
2Θ range for data collection/°	4.25 to 56.572
Index ranges	$-43 \le h \le 51, -14 \le k \le 14, -12 \le l \le 13$
Reflections collected	19133
Independent reflections	5041 [R_{int} = 0.0691, R_{sigma} = 0.0597]
Data/restraints/parameters	5041/1/263
Goodness-of-fit on F^2	1.016
Final R indexes [I>=2σ (I)]	R_1 = 0.0523, wR_2 = 0.1370
Final R indexes [all data]	R_1 = 0.0620, wR_2 = 0.1463
Largest diff. peak/hole / e Å^{-3}	0.40/-0.50

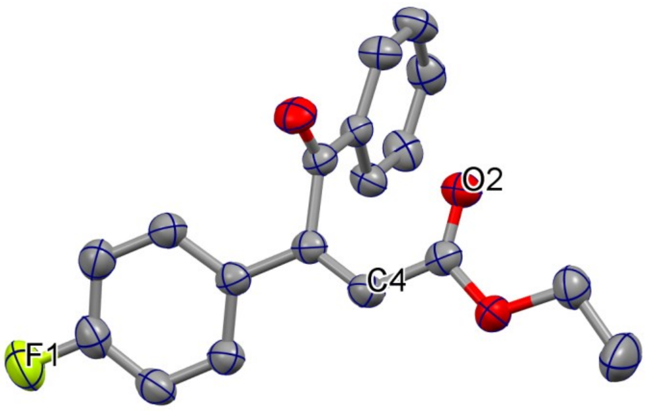

Supplementary Table 11. Crystal data and structure refinement for 5u.
CCDC: 2202499

Identification code	2_a
Empirical formula	$C_{18}H_{15}FO_3$
Formula weight	298.30
Temperature/K	296.15
Crystal system	orthorhombic
Space group	Pbca
a/Å	11.3707(3)
b/Å	13.3857(4)
c/Å	20.1113(6)
α/°	90
β/°	90
γ/°	90
Volume/Å^3	3061.04(15)

Z	8
ρ_{calc}g/cm^3	1.295
μ/mm^{-1}	0.095
F(000)	1248.0
Crystal size/mm^3	? × ? × ?
Radiation	MoKα (λ = 0.71073)
2Θ range for data collection/°	4.05 to 55.006
Index ranges	-14 ≤ h ≤ 14, -16 ≤ k ≤ 17, -25 ≤ l ≤ 26
Reflections collected	27377
Independent reflections	3511 [R_{int} = 0.0597, R_{sigma} = 0.0344]
Data/restraints/parameters	3511/0/200
Goodness-of-fit on F^2	1.065
Final R indexes [I>=2σ (I)]	R_1 = 0.0480, wR_2 = 0.0976
Final R indexes [all data]	R_1 = 0.0782, wR_2 = 0.1124
Largest diff. peak/hole / e Å^{-3}	0.17/-0.15

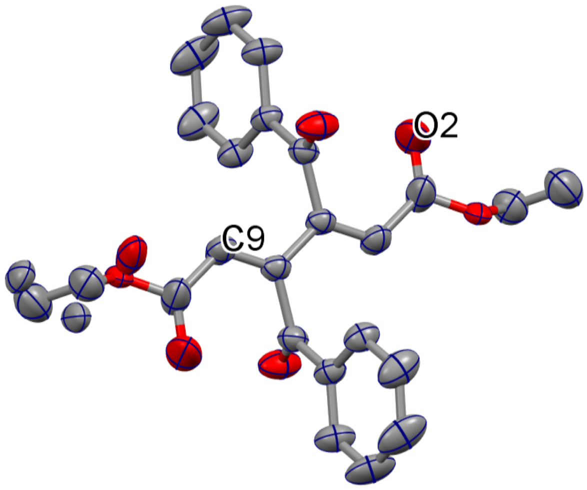

Supplementary Table 12. Crystal data and structure refinement for 8.
CCDC: 2202500

Identification code	data_220718li_xiejin_0m_a
Empirical formula	$C_{24}H_{22}O_6$
Formula weight	406.41
Temperature/K	193.0
Crystal system	monoclinic
Space group	$P2_1/c$
a/Å	9.0194(5)
b/Å	13.4473(7)
c/Å	9.6359(5)
α/°	90
β/°	116.234(3)

γ/°	90
Volume/Å^3	1048.32(10)
Z	2
ρ_{calc}g/cm^3	1.288
μ/mm^{-1}	0.489
F(000)	428.0
Crystal size/mm^3	0.16 × 0.12 × 0.11
Radiation	GaKα (λ = 1.34139)
2Θ range for data collection/°	9.51 to 107.914
Index ranges	-9 ≤ h ≤ 10, -12 ≤ k ≤ 16, -11 ≤ l ≤ 11
Reflections collected	6761
Independent reflections	1909 [R_{int} = 0.0339, R_{sigma} = 0.0313]
Data/restraints/parameters	1909/189/166
Goodness-of-fit on F^2	1.066
Final R indexes [I>=2σ (I)]	R_1 = 0.0540, wR_2 = 0.1296
Final R indexes [all data]	R_1 = 0.0704, wR_2 = 0.1412
Largest diff. peak/hole / e Å^{-3}	0.21/-0.25

6. Mechanistic study

6.1 ^{18}O labeling experiment

a. ESI-MS monitoring of oxo-arylfluorination reaction

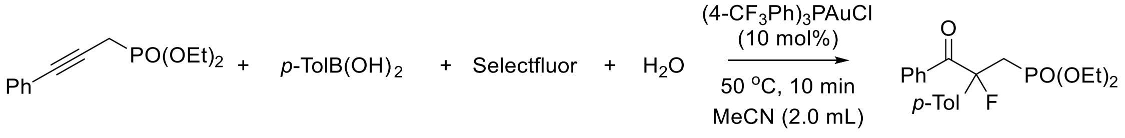

1a **2a** **3a**

To a dried Schlenk tube, (4-CF_3Ph)$_3$PAuCl (0.01mmol, 10 mol%), **2** (0.3 mmol, 3.0 equiv.) and Selectfluor (0.4 mmol, 4.0 equiv.) are successively added under air atmosphere. Then MeCN (2.0 mL) is added into the tube under stirring conditions. After that, alkynes **1** (0.1 mmol, 1.0 equiv.) and water (0.2 mmol, 2.0 euqiv) are added by microinjector under air atmosphere. The reaction mixture is heated at 50 °C for 10 minutes and stops heating. (footnote: prepare the corresponding ESI-MS sample and test in the shortest time).

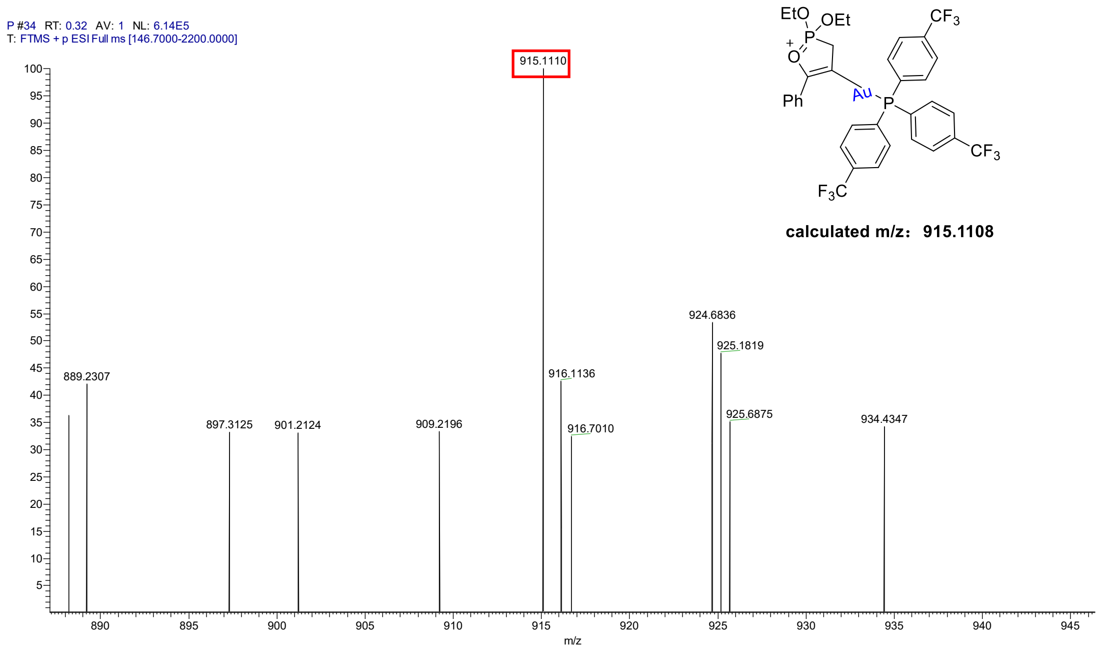

Supplementary Figure 1. HRMS investigation of oxo-arylfluorination reaction mixture at 10 minutes (reaction-intermediate, calculated 915.1108, found: 915.1110)

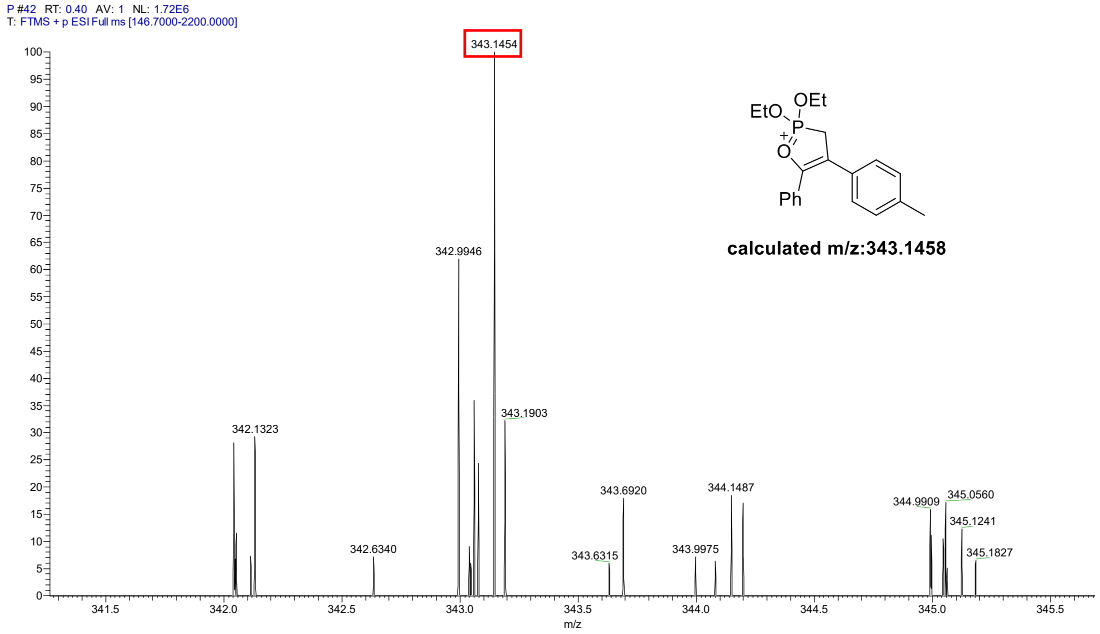

Supplementary Figure 2. HRMS investigation of oxo-arylfluorination reaction mixture at 10 minutes (reaction-intermediate, calculated 343.1458, found: 343.1454)

b. ESI-MS monitoring of oxo-arylalkenylation reaction

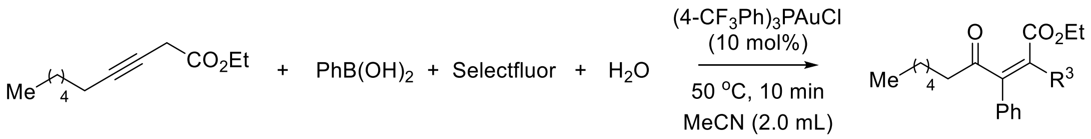

4a **3d** **5a**

To a dried Schlenk tube, $(4\text{-}CF_3Ph)_3PAuCl$ (0.01mmol, 10 mol%), **3d** (0.3 mmol, 3.0 equiv.) and Selectfluor (0.25 mmol, 2.5 equiv.) are successively added under air atmosphere. Then MeCN (2.0 mL) is added into the tube under stirring conditions. After that, alkynes **4a** (0.1 mmol, 1.0 equiv.) and water (0.2 mmol, 2.0 euqiv) are added by microinjector under air atmosphere. The reaction mixture is heated at 50 °C for 10 minutes and stops heating.

(footnote: prepare the corresponding ESI-MS sample and test in the shortest time).

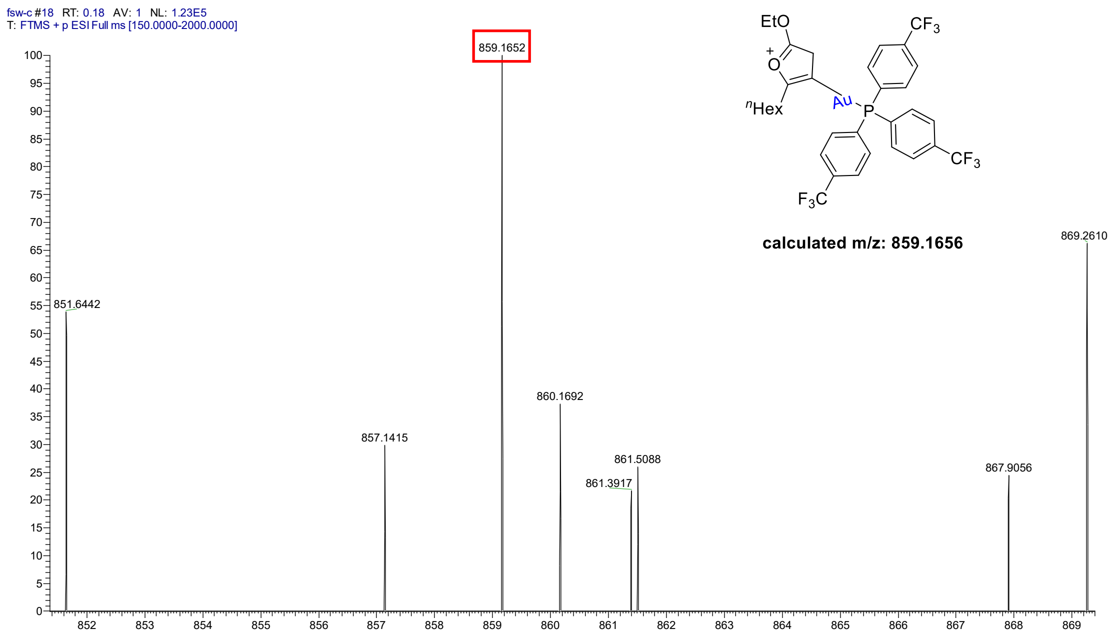

Supplementary Figure 3. HRMS investigation of oxo-arylalkenylation reaction mixture at 10 minutes (reaction-intermediate, calculated 859.1656, found: 859.1652)

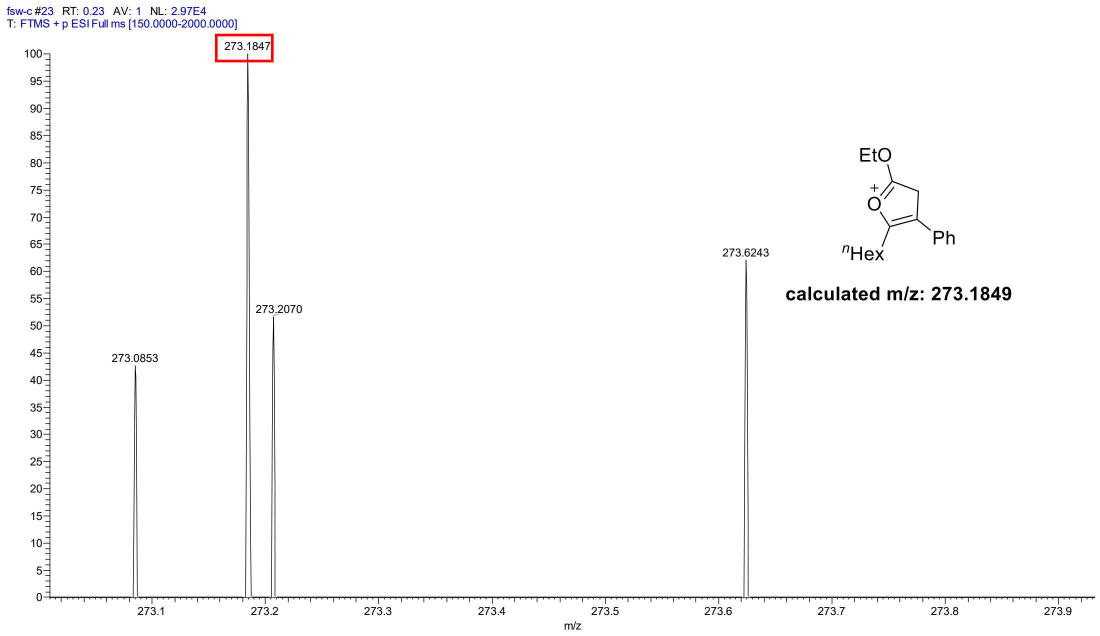

Supplementary Figure 4. HRMS investigation of oxo-arylalkenylation reaction mixture at 10 minutes (reaction-intermediate, calculated 273.1849, found: 273.1847)

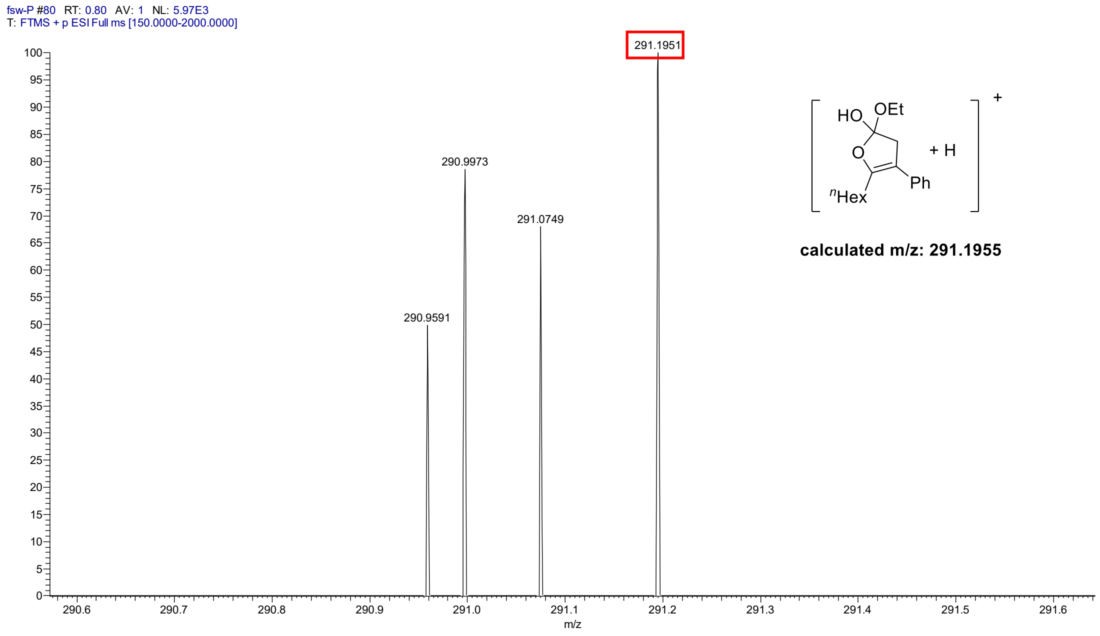

Supplementary Figure 5. HRMS investigation of oxo-arylalkenylation reaction mixture at 10 minutes (reaction-intermediate, calculated 291.1955, found: 291.1951)

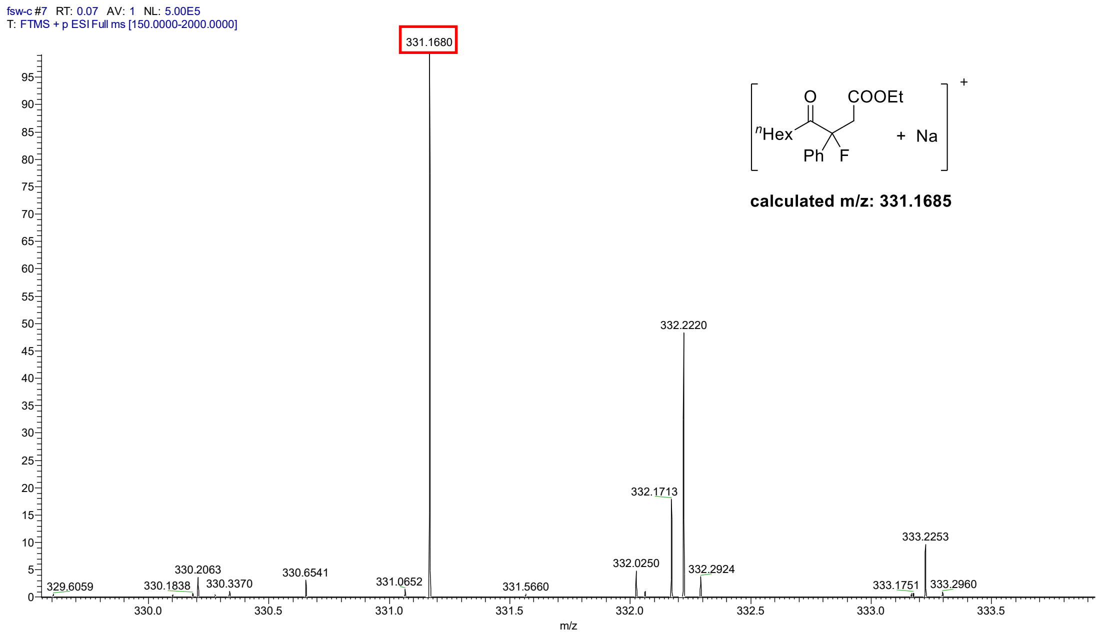

Supplementary Figure 6. HRMS investigation of oxo-arylalkenylation reaction mixture at 10 minutes (reaction-intermediate, calculated 331.1685, found: 331.1680)

6.2 ^{18}O labeling experiment

a. ^{31}P NMR for ^{18}O labeling experiment of oxidative oxo-arylfluorination of alkynes.

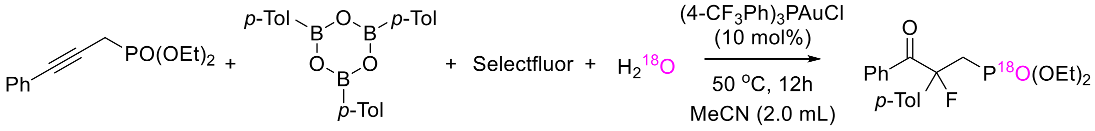

1a **6** **x equiv** **3a-^{18}O**

To a dried Schlenk tube, $(4\text{-}CF_3Ph)_3PAuCl$ (0.01mmol, 10 mol%), **6** (0.1 mmol, 1.0 equiv.) and Selectfluor (0.4 mmol, 4.0 equiv.) are successively added under air atmosphere. Then MeCN (2.0 mL) is added into the tube under stirring conditions. After that, alkynes **1a** (0.1 mmol, 1.0 equiv.) and $H_2{}^{18}O$ (x euqiv) are added by microinjector under air atmosphere. The resulting reaction mixture is heated at 50 °C. When the reaction is finished (monitored by TLC), the reaction mixture is cooled to room temperature. Prepare the corresponding ESI-MS sample and test. After ESI-MS test, recycle the example and combine with reaction mixture. Concentrated in vacuo,

the resulting residue is purified by column chromatography on silica gel (eluent: petroleum ether/ethyl acetate = 2:1) to give the final product **3a-^{18}O**. The ^{18}O labeling resulting are derived from ESI-MS data.

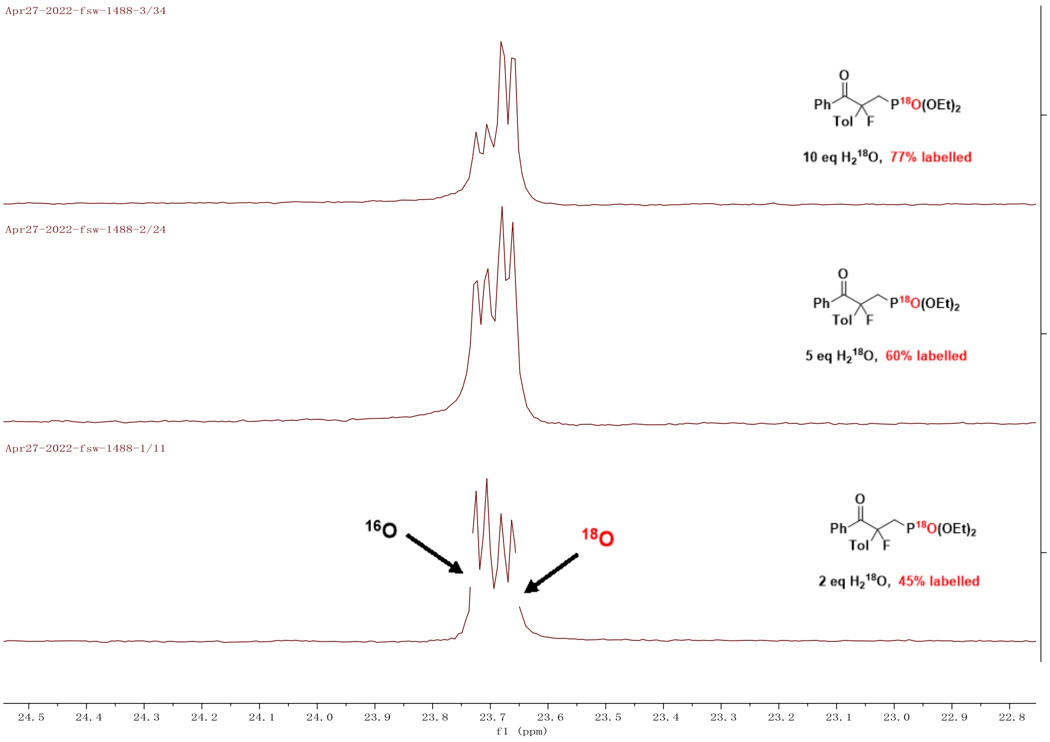

Supplementary Figure 7. ^{31}P NMR for ^{18}O labeling experiment of oxo-arylfluorination reaction

b. ^{13}C NMR for ^{18}O labeling experiment of oxo-arylalkenylation reaction

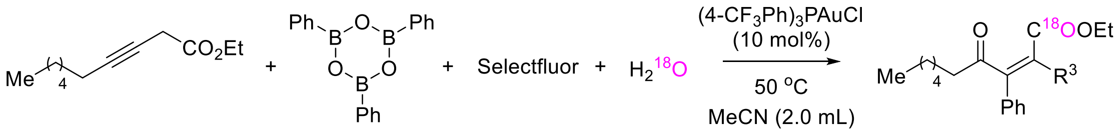

4a **7** **5a-^{18}O**

To a dried Schlenk tube, $(4\text{-}CF_3Ph)_3PAuCl$ (0.01mmol, 10 mol%), **7** (0.1 mmol, 1.0 equiv.) and Selectfluor (0.25 mmol, 2.5 equiv.) are successively added under air atmosphere. Then MeCN (2.0 mL) is added into the tube under stirring conditions. After that, alkynes **4a** (0.1 mmol, 1.0 equiv.) and $H_2{}^{18}O$ (x euqiv) are added by microinjector under air atmosphere. The resulting reaction mixture is heated at 50 °C. When the reaction is finished (monitored by TLC), the reaction mixture is cooled to room temperature. Prepare the corresponding ESI-MS sample and test. After ESI-MS test, recycle and combine the example with reaction mixture. Concentrated in vacuo,

the resulting residue is purified by column chromatography on silica gel (eluent: petroleum ether/ethyl acetate = 20:1) to give the final product **5a-^{18}O**. The ^{18}O labeling resulting are derived from ESI-MS data.

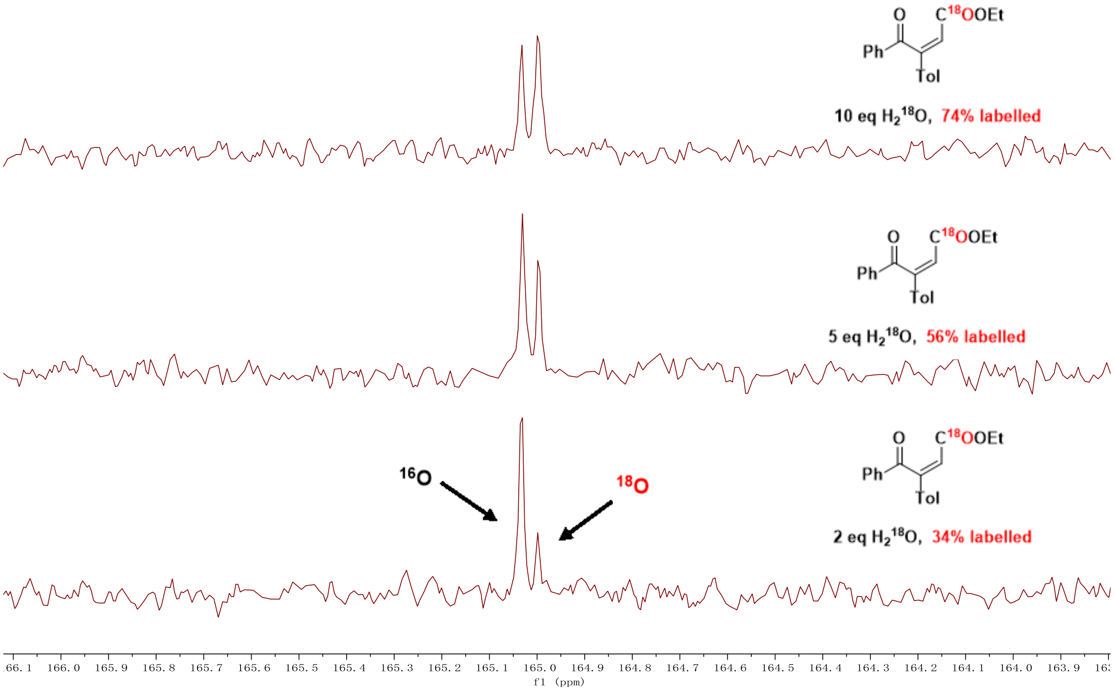

Supplementary Figure 8. ^{13}C NMR for ^{18}O labeling experiment of oxo-arylalkenylation reaction

7. Preparation of starting materials

Synthesis of alkynes 1a - 1aa [1]

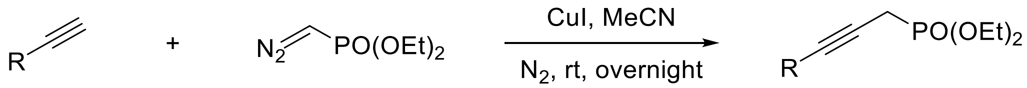

To an oven-dried flask (100 mL) with CuI (0.5 mmol, 5 mol%) and MeCN (30 mL) under N_2 atmosphere, alkynes (10 mol, 1.0 equiv..) are added. And then, diethyl diazomethylphosphonates (10 mol, 1.0 equiv..) are added dropwise into the mixture and stirred at room temperature overnight. After filtration and concentration under reduced pressure, the crude product is purified by column chromatography on silica gel to afford proparylphosphonates (eluent: EA/PE = 1:1 to 1:3, v/v).

Preparation of alkynes 1bb - 1cc [2]

Step 1

To an oven-dried flask (50 mL) are added $Pd(PPh_3)_2Cl_2$ (2 mol%), CuI (10 mol%) and equips with a magnetic stir bar. Then iodobenzene (10 mmol, 1.0 equiv..) and Et_3N (25 mL) are added through syringe under N_2 atmosphere at room temperature. Stirred at the same temperature for 15 min and 4-bromo-1-butyne (12 mmol, 1.2 equiv..) is added to the mixture. The reaction is conducted at room temperature overnight. Saturated aqueous NH_4Cl (20 mL) and EtOAc (30 mL) are added. Keep the organic phase and washed with brine, dried over Na_2SO_4. Concentrated in vacuo, and the residue is purified by column chromatography on silica gel to afford the desired product (eluent: EA/PE = 1:20, v/v).

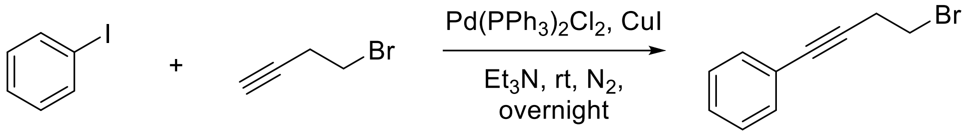

Step 2

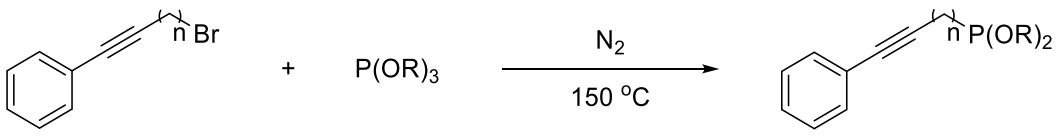

To an oven-dried flask (25 mL) are added the alkynyl bromides (10 mol, 2.0 equiv..) and $P(OR)_3$ (5 mol, 1 equiv..) under N_2 atmosphere. The mixture is stirred at 150°C overnight. After that, the mixture is cooled to room temperature and purified by column chromatography on silica gel to afford proparylphosphonates (eluent: EA/PE = 1:2 to 1:3, v/v).

Synthesis of 3-alkynoates [3]

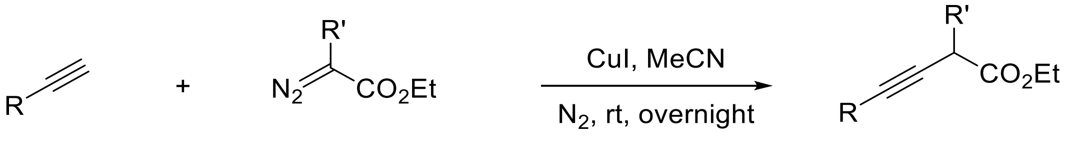

To a solution of alkynes (12 mmol, 1.2 equiv..) and CuI (0.5 mol, 5 mol%) in MeCN (30 mL) under N_2 atmosphere, diazocarbonyl compounds (10 mmol, 1.0 equiv..) are

added. The mixture is stirred at room temperature overnight. And then, the solvent is removed in vacuo and the residue is purified by column chromatography on silica gel to afford 3-alkynoates (eluent: EA/PE = 1:15 to 1:20, v/v).

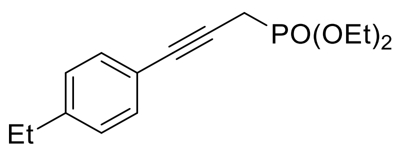

1p

diethyl (3-(4-ethylphenyl)prop-2-yn-1-yl)phosphonate

Prepared according to the general procedure 2.1. Flash column chromatography (eluent: EA/PE = 1:2, v/v) to afford **1p** as a light-yellow oil (496.7 mg, yield 50%, for the alkyne).

^{1}H NMR (500 MHz, Chloroform-*d*) δ 7.32 (d, *J* = 8.0 Hz, 2H), 7.11 (d, *J* = 8.0 Hz, 2H), 4.26 – 4.18 (m, 4H), 2.97 (d, *J* = 22.1 Hz, 2H)., 2.62 (q, *J* = 7.6 Hz, 2H), 1.36 (t, *J* = 7.1 Hz, 6H), 1.21 (t, *J* = 7.6 Hz, 3H);

^{13}C NMR (125 MHz, Chloroform-*d*) δ 144.5, 131.6 (d, *J* = 3.3 Hz), 127.7, 120.1 (d, *J* = 4.1 Hz), 82.8 (d, *J* = 10.4 Hz), 78.6 (d, *J* = 14.9 Hz), 63.0, 62.9, 28.7, 18.8 (d, *J* = 145.6 Hz), 16.4 (d, *J* = 5.9 Hz), 15.3;

^{31}P NMR (202 MHz, Chloroform-*d*) δ 21.61;

IR (ATR): *v* = 2968, 2932, 1737, 1510, 1443, 1392, 1256, 1163, 1049, 961;

HRMS (ESI) Calculated for $C_{15}H_{22}O_3P$ $[M+H]^+$: 281.1301; found 281.1297.

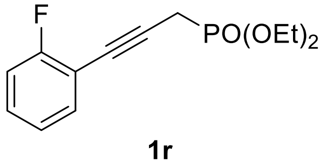

1r

diethyl (3-(2-fluorophenyl)prop-2-yn-1-yl)phosphonate

Prepared according to the general procedure 2.1. Flash column chromatography (eluent: EA/PE = 1:2, v/v) to afford **1r** as a light-yellow oil (167.5 mg, yield 62%, 6.0 mmol for the alkyne);

^{1}H NMR (500 MHz, Chloroform-*d*) δ 7.43 – 7.39 (m, 1H), 7.32 – 7.26 (m, 1H), 7.10 –

7.02 (m, 2H), 4.27 – 4.22 (m, 4H), 3.03 (d, *J* = 22.1 Hz, 2H), 1.38 (t, *J* = 7.1 Hz, 6H);

^{13}C NMR (125 MHz, Chloroform-*d*) δ 133.7, 129.9, 129.9, 123.9 (d, *J* = 3.8 Hz), 115.5, 115.4, 84.9 (d, *J* = 14.8 Hz), 76.3 (d, *J* = 10.5 Hz), 63.2, 63.1, 19.0 (d, *J* = 145.1 Hz)., 16.4 (d, *J* = 5.9 Hz);

^{19}F NMR (471 MHz, Chloroform-d) δ -110.47;

^{31}P NMR (202 MHz, Chloroform-*d*) δ 20.83;

IR (ATR): *v* = 2981, 2922, 2851, 1449, 1260, 1052, 1020, 969;

HRMS (ESI) Calculated for $C_{13}H_{17}FO_3P$ $[M+H]^+$: 271.0894; found 271.0889.

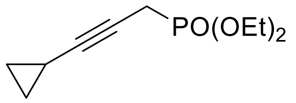

1v

diethyl (3-cyclopropylprop-2-yn-1-yl)phosphonate

Prepared according to the general procedure 2.1. Flash column chromatography (eluent: EA/PE = 1:4, v/v) to afford **1v** as a pale-yellow oil (395.7 mg, yield 61%, 3.0 mmol for the alkyne);

^{1}H NMR (500 MHz, Chloroform-*d*) δ 4.16 – 4.12 (m, 4H), 2.66 (dd, *J* = 21.8, 2.1 Hz, 2H), 1.32 (t, *J* = 7.1 Hz, 6H), 1.23 – 1.12 (m, 1H), 0.72 – 0.65 (m, 2H), 0.63 – 0.57 (m, 2H);

^{13}C NMR (125 MHz, Chloroform-*d*) δ 85.9 (d, *J* = 9.8 Hz), 64.6 (d, *J* = 14.9 Hz), 62.7 (d, *J* = 6.5 Hz), 18.0 (d, *J* = 146.2 Hz), 16.3 (d, *J* = 5.9 Hz), 7.8 (d, *J* = 2.7 Hz), -0.5 (d, *J* = 3.9 Hz);

^{31}P NMR (202 MHz, Chloroform-*d*) δ 22.58;

IR (ATR): *v* = 2983, 2906, 1392, 1258, 1017, 959, 775;

HRMS (ESI) Calculated for $C_{10}H_{18}O_3P$ $[M+H]^+$: 217.0988; found 217.0989.

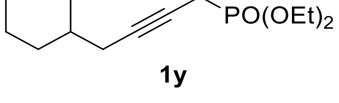

1y

diethyl (4-cyclohexylbut-2-yn-1-yl)phosphonate

Prepared according to the general procedure 2.1. Flash column chromatography (eluent: EA/PE = 1:4, v/v) to afford **1y** as a pale-yellow oil (702.6 mg, yield 43%, 6.0 mmol for the alkyne);

^{1}H NMR (500 MHz, Chloroform-*d*) δ 4.18 – 4.12 (m, 4H), 3.40 (d, *J* = 21.9 Hz, 2H), 2.62 – 2.60 (m, 2H), 1.80 – 1.69 (m, 5H), 1.67 – 1.60 (m, 1H), 1.33 (t, *J* = 7.1 Hz, 6H), 1.27 – 1.12 (m, 3H), 1.04 – 0.98 (m, 2H);

^{13}C NMR (125 MHz, Chloroform-*d*) δ 125.2 (d, *J* = 14.4 Hz), 110.0 (d, *J* = 14.5 Hz), 62.4, 62.3, 47.7 (d, *J* = 2.9 Hz), 39.8 (d, *J* = 142.2 Hz), 37.0 (d, *J* = 3.7 Hz), 32.3, 26.3, 26.1, 16.4 (d, *J* = 6.3 Hz);

^{31}P NMR (202 MHz, Chloroform-*d*) δ 22.18;

IR (ATR): *v* = 2981, 2922, 2851, 1448, 1260, 1052, 1020, 959, 841;

HRMS (ESI) Calculated for $C_{14}H_{26}O_3P$ $[M+H]^+$: 273.1614; found 273.1609.

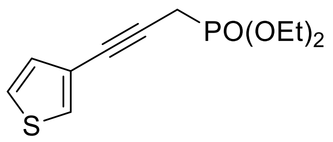

1z

diethyl (3-(thiophen-3-yl)prop-2-yn-1-yl)phosphonate

Prepared according to the general procedure 2.1. Flash column chromatography (eluent: EA/PE = 1:3, v/v) to afford **1z** as a yellow oil (1162.2 mg, yield 45%, 10.0 mmol for the alkyne);

^{1}H NMR (500 MHz, Chloroform-*d*) δ 7.42 – 7.38 (m, 1H), 7.25-7.26 (m, 1H), 7.09 – 7.07 (m, 1H), 4.25 – 4.19 (m, 4H), 2.96 (d, *J* = 22.0 Hz, 2H), 1.37 (t, *J* = 7.1 Hz, 6H);

^{13}C NMR (125 MHz, Chloroform-*d*) δ 129.9 (d, *J* = 2.8 Hz), 128.7 (d, *J* = 4.0 Hz), 125.2, 79.0 (d, *J* = 15.0 Hz), 77.9 (d, *J* = 10.2 Hz), 63.0 (d, *J* = 6.5 Hz), 18.8 (d, *J* = 145.8 Hz), 16.4 (d, *J* = 6.0 Hz);

^{31}P NMR (202 MHz, Chloroform-*d*) δ 21.46;

IR (ATR): *v* = 2981, 2905, 1391, 1254, 1215, 1163, 1049, 1018, 960, 859;

HRMS (ESI) Calculated for $C_{11}H_{16}O_3PS$ $[M+H]^+$: 259.0552; found 259.0549.

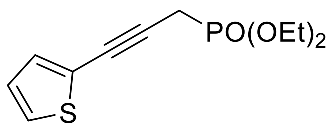

1aa

diethyl (3-(thiophen-2-yl)prop-2-yn-1-yl)phosphonate

Prepared according to the general procedure 2.1. Flash column chromatography (eluent: EA/PE = 1:3, v/v) to afford **1aa** as a yellow oil (363.0 mg, yield 35%, 4.0 mmol for the alkyne);

^{1}H NMR (500 MHz, Chloroform-*d*) δ 7.22 – 7.20 (m, 1H), 7.18 – 7.17 (m, 1H), 6.94 – 6.95 – 6.64 (m 1H), 4.26 – 4.18 (m, 4H), 2.99 (d, *J* = 22.0 Hz, 2H), 1.37 (t, *J* = 7.1 Hz, 6H);

^{13}C NMR (125 MHz, Chloroform-*d*) δ 131.9 (d, *J* = 3.4 Hz), 126.8, 126.7, 83.5 (d, *J* = 15.4 Hz), 76.1 (d, *J* = 10.4 Hz), 63.0 (d, *J* = 6.8 Hz), 19.1 (d, *J* = 145.6 Hz), 16.4 (d, *J* = 5.9 Hz)

^{31}P NMR (202 MHz, Chloroform-*d*) δ 20.91;

IR (ATR): *v* = 2982, 2930, 2165, 1626, 1412, 1391, 1248, 1014, 966, 848;

HRMS (ESI) Calculated for $C_{11}H_{16}O_3PS$ $[M+H]^+$: 259.0552; found 259.0548.

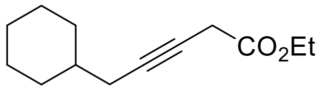

4m

ethyl 5-cyclohexylpent-3-ynoate

Prepared according to the general procedure 2.2. Flash column chromatography (eluent: EA/PE = 1:20 v/v) to afford **4m** as a pale-yellow oil (293.7 mg, yield 47%, 3.0 mmol for the alkyne).

^{1}H NMR (500 MHz, Chloroform-*d*) δ 4.18 (q, *J* = 7.1 Hz, 2H), 3.24 (t, *J* = 2.5 Hz, 2H), 2.09 – 2.07 (m, 2H), 1.82 – 1.78 (m, 2H), 1.73 – 1.69 (m, 2H), 1.66 – 1.62 (m, 1H), 1.50 – 1.41 (m, 1H), 1.28 (t, *J* = 7.1 Hz, 3H), 1.25 – 1.19 (m, 2H), 1.18 – 1.10 (m, 1H), 1.02 – 0.93 (m, 2H);

^{13}C NMR (125 MHz, Chloroform-*d*) δ 169.0, 82.7, 72.3, 61.4, 37.4, 32.6, 26.6, 26.3, 26.2, 26.1, 14.1;

IR (ATR): v = 2982, 2922, 2851, 1739, 1449, 1368, 1325, 1258, 1162, 1029;

HRMS (ESI) Calculated for $C_{13}H_{21}O_2$ $[M+H]^+$: 209.1536; found 209.1536.

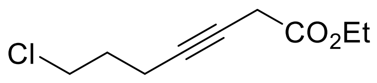

4n

ethyl 7-chlorohept-3-ynoate

Prepared according to the general procedure 2.2. Flash column chromatography (eluent: EA/PE = 1:20, v/v) to afford **4n** as a pale-yellow oil (556.4 mg, yield 59%, 5.0 mmol for the alkyne).

^{1}H NMR (500 MHz, Chloroform-*d*) δ 4.21 (q, J = 7.1 Hz, 2H), 3.78 (s, 2H), 3.59 (t, J = 6.6 Hz, 2H), 2.92 – 2.87 (m, 2H), 2.13 – 2.08 (m, 2H), 1.29 (t, J = 7.1 Hz, 3H);

^{13}C NMR (125 MHz, Chloroform-*d*) δ 168.1, 124.5, 113.0, 61.4, 46.9, 43.3, 38.1, 30.4, 14.1;

IR (ATR): v = 2982, 1739, 1444, 1404, 1369, 1259, 1178, 1096, 1028, 652;

HRMS (ESI) Calculated for $C_9H_{14}ClO_2$ $[M+H]^+$: 189.0677; found 189.0676.

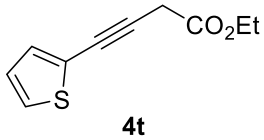

4t

ethyl 4-(thiophen-2-yl)but-3-ynoate

Prepared according to the general procedure 2.2. Flash column chromatography (eluent: EA/PE = 1:15, v/v) to afford **4t** as a yellow oil (951.8 mg, yield 49%, 10.0 mmol for the alkyne).

^{1}H NMR (500 MHz, Chloroform-*d*) δ 7.21 – 7.19 (m, 2H), 6.95 – 6.93 (m, 1H), 4.21 (q, J = 7.1 Hz, 2H), 3.51 (s, 2H), 1.29 (t, J = 7.1 Hz, 3H).

^{13}C NMR (125 MHz, Chloroform-*d*) δ 167.9, 132.0, 126.8, 85.2, 61.8, 27.0, 14.1.

IR (ATR): v = 2981, 1736, 1368, 1330, 1299, 1258, 1159, 1095, 1027, 847;

HRMS (ESI) Calculated for $C_{10}H_{11}O_2S$ $[M+H]^+$: 195.0474; found 195.0472.

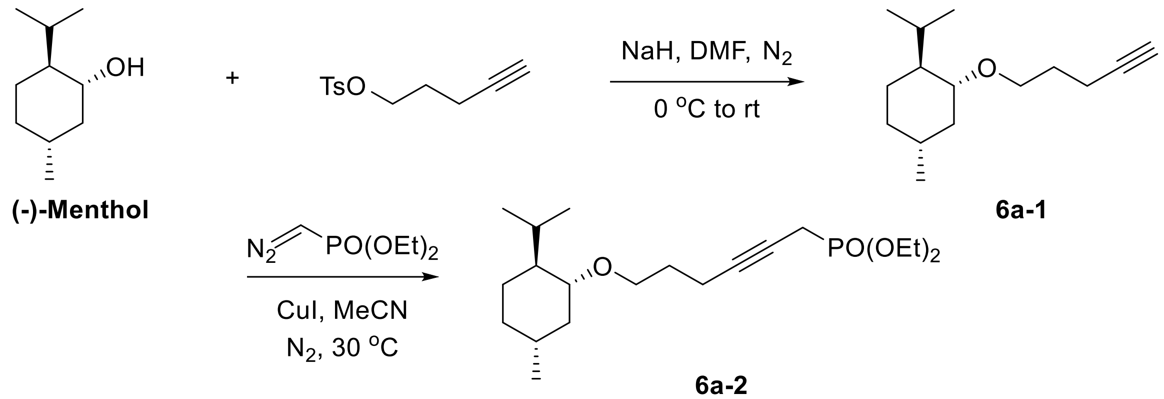

Under N_2 atmosphere, to a solution of (-)-Menthol (10.0 mmol, 1.0 eq) in DMF (20 mL) is added NaH (1.5 eq, 60% dispersion in mineral oil) at 0 °C. And then, the mixture is warmed to rt and stirred for 1 hours. Pent-4-yn-1-yl 4-methylbenzenesulfonate (10.0 mmol, 1.0 eq) in DMF (10 mL) is added dropwise in 10 min. The mixture is warmed up to 50 °C and stirred for 24 hours. The reaction mixture is quenched with NH_4Cl saturated solution, and the organic layer is extracted with EA, washed with brine and dried over anhydrous Na_2SO_4. The residue is purified by chromatography on silica gel with EA/PE (1:40, v/v) as the eluent to afford desired product **6a-1** as a colorless oil (75% yield).

^{1}H NMR (500 MHz, Chloroform-*d*) δ 3.72 – 3.68 (m, 1H), 3.39 – 3.35 (m, 1H), 3.04 – 2.99 (m, 1H), 2.32 – 2.28 (m, 2H), 2.23 – 2.17 (m, 1H), 2.12 – 2.07 (m, 1H), 1.93 (t, *J* = 2.7 Hz, 1H), 1.80 – 1.73 (m, 2H), 1.66 – 1.59 (m, 3H), 1.37 – 1.31 (m, 1H), 1.23 – 1.18 (m, 1H), 0.98 – 0.95 (m, 1H), 0.91 (d, *J* = 6.6 Hz, 3H), 0.85 – 0.82 (m, 3H), 0.85 – 0.81 (m, 1H), 0.77 (d, *J* = 7.0 Hz, 3H);

^{13}C NMR (125 MHz, Chloroform-*d*) δ 84.2, 79.3, 68.3, 66.6, 48.3, 40.5, 34.6, 31.6, 29.2, 25.6, 23.3, 22.4, 21.0, 16.2 15.4;

IR (ATR): *v* = 3312, 2953, 2920, 2868, 1456, 1370, 1239, 1111, 1093, 626;

HRMS (ESI) Calculated for $C_{15}H_{27}O$ $[M+H]^+$: 223.2056; found 223.2053.

To a solution of **6a-1** (2.0 mmol, 1.0 eq) and CuI (0.2 mol, 10 mol%) in MeCN (15 mL) are added diethyl (diazomethyl)phosphonate (3.0 mmol, 1.5 eq) under N_2 atmosphere. The reaction mixture is stirred at 30 °C for 12 hours. After filtration and concentration under reduced pressure, the crude product is purified by column chromatography on

silica gel with EA/PE (1:2, v/v) to afford **6a-2** as a pale-yellow oil (54% yield).

^{1}H NMR (500 MHz, Chloroform-*d*) δ 4.15 – 4.10 (m, 4H), 3.65 – 3.61 (m, 1H), 3.31 – 3.26 (m, 1H), 2.96 – 2.93 (m, 1H), 2.70 – 2.64 (m, 2H), 2.25 – 2.20 (m, 2H), 2.18 – 2.11 (m, 2H), 2.06 – 2.02 (m, 1H), 1.71 – 1.65 (m, 2H), 1.61 – 1.53 (m, 2H), 1.30 (t, *J* = 7.1 Hz, 6H), 1.18 – 1.12 (m, 1H), 0.93 – 0.75 (m, 9H), 0.72 (d, *J* = 7.0 Hz, 3H);

^{13}C NMR (125 MHz, Chloroform-*d*) δ 82.5 (d, *J* = 10.3 Hz), 79.2, 69.4 (d, *J* = 14.5 Hz), 66.8, 62.7, 62.6, 48.2, 40.4, 34.5, 31.4, 29.3 (d, *J* = 2.9 Hz), 25.5, 23.2, 22.2, 20.8, 18.5, 17.3, 16.3 (d, *J* = 5.9 Hz), 16.1, 15.7 (d, *J* = 3.1 Hz);

^{31}P NMR (202 MHz, Chloroform-*d*) δ 22.79;

IR (ATR): *v* = 2952, 2920, 2868, 1456, 1262, 1110, 1054, 1022, 962, 842;

HRMS (ESI) Calculated for $C_{20}H_{38}O_4P$ $[M+H]^+$: 373.2502; found 373.2495.

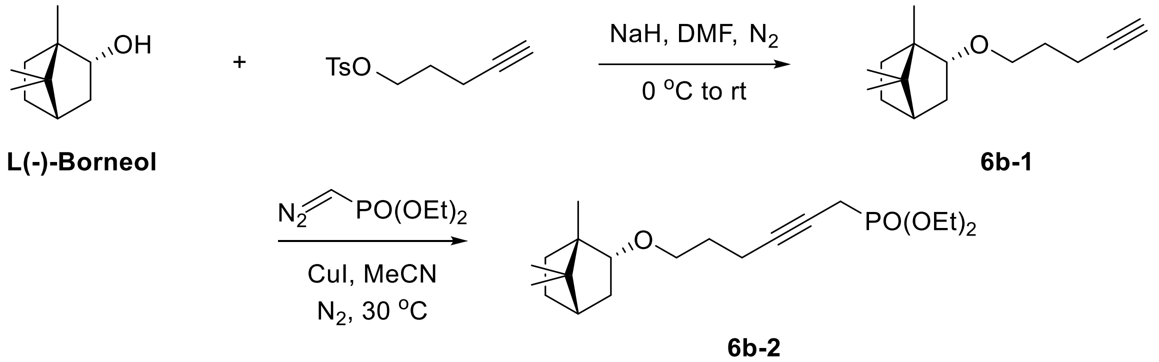

Under N_2 atmosphere, to a solution of L(-)-Borneol (10.0 mmol, 1.0 eq) in DMF (20 mL) is added NaH (1.5 eq, 60% dispersion in mineral oil) at 0 °C. And then, the mixture is warmed to rt and stirred for 1 hours. Pent-4-yn-1-yl 4-methylbenzenesulfonate (10.0 mmol, 1.0 eq) in DMF (10 mL) is added dropwise in 10 min. The mixture is warmed up to 50 °C and stirred for 24 hours. The reaction mixture is quenched with NH_4Cl saturated solution, and the organic layer is extracted with EA, washed with brine and dried over anhydrous Na_2SO_4. The residue is purified by chromatography on silica gel with EA/PE (1:40, v/v) as the eluent to afford desired product **6b-1** as a colorless oil (61% yield).

^{1}H NMR (500 MHz, Chloroform-*d*) δ 3.55 – 3.51 (m, 2H), 3.44 – 3.39 (m, 1H), 2.31 – 2.28 (m, 2H), 2.13 – 2.07 (m, 1H), 1.99 – 1.89 (m, 2H), 1.79 – 1.73 (m, 2H), 1.71 – 1.65 (m, 1H), 1.62 – 1.60 (m, 1H), 1.23 – 1.15 (m, 2H), 1.00 (dd, *J* = 13.0, 3.3 Hz, 1H),

0.86 (s, 3H), 0.83 (s, 6H);

^{13}C NMR (125 MHz, Chloroform-*d*) δ 84.7, 84.4, 68.2, 68.0, 49.2, 47.8, 45.0, 36.2, 29.1, 28.2, 26.6, 19.8, 18.8, 15.3, 14.0;

IR (ATR): *v* = 3312, 2949, 2873, 1454, 1388, 1362, 1140, 1118, 1099, 1077, 626;

HRMS (ESI) Calculated for $C_{15}H_{25}O$ $[M+H]^+$: 221.1900; found 221.1897.

To a solution of **6b-1** (2.0 mmol, 1.0 eq) and CuI (0.2 mol, 10 mol%) in MeCN (15 mL) is added diethyl (diazomethyl)phosphonate (3.0 mmol, 1.5 eq) under N_2 atmosphere. The reaction mixture is stirred at 30 °C for 12 hours. After filtration and concentration under reduced pressure, the crude product is purified by column chromatography on silica gel with EA/PE (1:2, v/v) to afford **6b-2** as a pale-yellow oil (50% yield).

^{1}H NMR (500 MHz, Chloroform-*d*) δ 4.15 – 4.12 (m, 4H), 3.52 – 3.45 (m, 2H), 3.39 – 3.35 (m, 1H), 2.72 – 2.67 (m, 2H), 2.27 – 2.23 (m, 2H), 2.10 – 2.04 (m, 1H), 1.96 – 1.90 (m, 1H), 1.74 – 1.63 (m, 3H), 1.59 (t, *J* = 4.6 Hz, 1H), 1.32 (t, *J* = 7.1 Hz, 6H), 1.18 – 1.13 (m, 2H), 0.98 – 0.94 (m, 1H), 0.82 (s, 3H), 0.81 (s, 6H);

^{13}C NMR (125 MHz, Chloroform-*d*) δ 84.6, 82.7 (d, *J* = 10.2 Hz), 69.3 (d, *J* = 14.5 Hz), 68.2, 62.73, 62.67, 49.1, 47.7, 45.0, 36.2, 29.2 (d, *J* = 2.9 Hz), 28.2, 26.6, 19.7, 18.8, 18.5, 17.4, 16.4 (d, *J* = 5.9 Hz), 15.7 (d, *J* = 3.0 Hz), 13.9;

^{31}P NMR (202 MHz, Chloroform-*d*) δ 22.83;

IR (ATR): *v* = 2981, 2948, 2873, 1388, 1262, 1118, 1097, 1053, 1021, 963;

HRMS (ESI) Calculated for $C_{20}H_{36}O_4P$ $[M+H]^+$: 371.2346; found 371.2338.

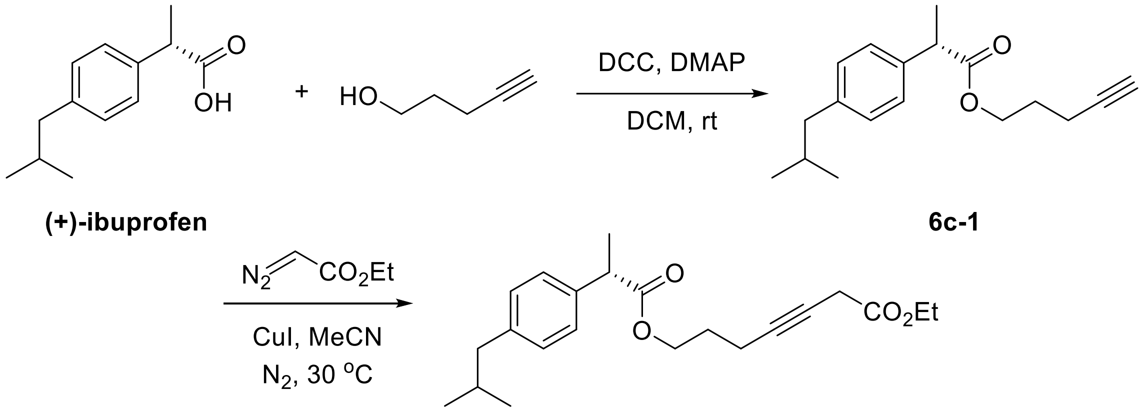

6c-2

To a solution of (+)-ibuprofen (10 mmol, 1.0 eq), DCC (15 mmol, 1.5 eq) and DMAP (2 mmol, 2 mol%) in DCM (30 mL), 4-pentyn-1-ol (15 mmol, 1.5 eq) is added at rt. The reaction mixture is stirred at the same temperature for overnight, before quenched with H_2O and extracted with DCM. The organic phase is dried over anhydrous Na_2SO_4. After filtration and concentration under reduced pressure, the crude product Is purified by column chromatography on silica gel with EA/PE (1:40, v/v) to afford **6c-1** as a colorless oil (87% yield).

^{1}H NMR (500 MHz, Chloroform-*d*) δ 7.20 (d, *J* = 8.1 Hz, 2H), 7.10 (d, *J* = 8.1 Hz, 2H), 4.17 (td, J = 6.1, 3.8 Hz, 2H), 3.69 (q, *J* = 7.2 Hz, 1H), 2.45 (d, *J* = 7.2 Hz, 2H), 2.16 – 2.13 (m, 2H), 1.93 (t, *J* = 2.7 Hz, 1H), 1.88 – 1.76 (m, 3H), 1.49 (d, *J* = 7.2 Hz, 3H), 0.90 (d, *J* = 6.7 Hz, 6H);

^{13}C NMR (125 MHz, Chloroform-*d*) δ 174.6, 140.5, 137.7, 129.3, 127.1, 82.9, 68.9, 63.0, 45.1, 45.0, 30.1, 27.5, 22.3, 18.3, 15.0;

IR (ATR): *ν* = 3294, 2930, 2854, 2117, 1733, 1450, 1240, 1071, 847;

HRMS (ESI) Calculated for $C_{18}H_{25}O_2$ $[M+H]^+$: 273.1849; found 273.1846.

To a solution of **6c-1** (4 mmol, 1.0 equiv..) and CuI (0.4 mol, 10 mol%) in MeCN (20 mL) under N_2 atmosphere, diazocarbonyl compounds (6 mmol, 1.5 equiv.) are added. The mixture is stirred at room temperature overnight. After filtration and concentration under reduced pressure, the crude product is purified by column chromatography on silica gel with EA/PE (1:20, v/v) to afford **6c-2** as a pale-yellow oil (43% yield).

^{1}H NMR (500 MHz, Chloroform-*d*) δ 7.18 (d, *J* = 8.2 Hz, 2H), 7.08 (d, *J* = 8.2 Hz, 2H),

4.22 – 4.11 (m, 4H), 3.68 (q, *J* = 7.2 Hz, 1H), 3.21 (t, *J* = 2.4 Hz, 2H), 2.44 (d, *J* = 7.2 Hz, 2H), 2.19 – 2.15 (m, 2H), 1.87 – 1.73 (m, 3H), 1.48 (d, *J* = 7.2 Hz, 3H), 1.26 (t, *J* = 7.1 Hz, 3H), 0.89 (d, *J* = 6.7 Hz, 6H).

^{13}C NMR (125 MHz, Chloroform-*d*) δ 174.6, 168.7, 140.4, 137.7, 129.3, 127.1, 82.2, 72.3, 63.3, 61.4, 45.10, 45.0, 30.1, 27.6, 26.0, 22.3, 18.4, 15.3, 14.1;

IR (ATR): *v* = 2956, 2870,1732, 1464, 1367, 1256, 1160, 1094, 1029;

HRMS (ESI) Calculated for $C_{22}H_{31}O_4$ $[M+H]^+$: 359.2217; found 359.2210.

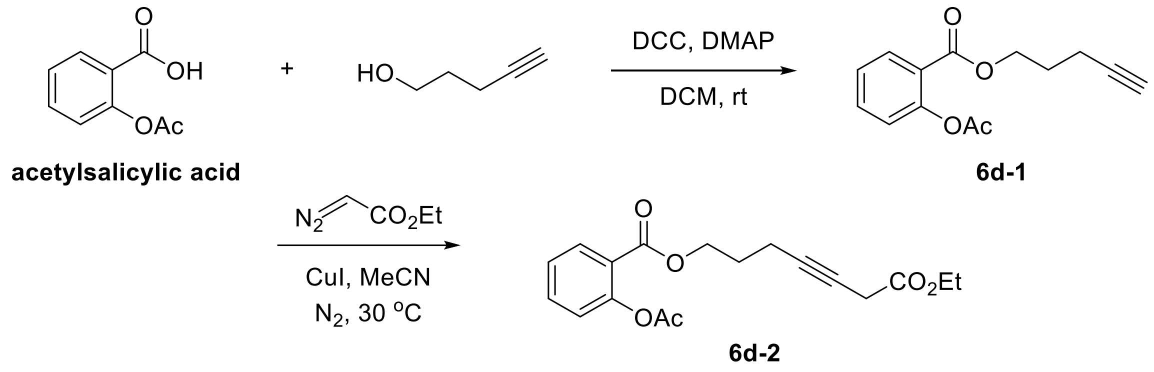

To a solution of acetylsalicylic acid (10 mmol, 1.0 eq), DCC (15 mmol, 1.5 eq) and DMAP (2 mmol, 2 mol%) in DCM (30 mL), 4-pentyn-1-ol (15 mmol, 1.5 eq) is added at rt. The reaction mixture is stirred at the same temperature for overnight, before quenched with H_2O and extracted with DCM. The organic phase is dried over anhydrous Na_2SO_4. After filtration and concentration under reduced pressure, the crude product is purified by column chromatography on silica gel with EA/PE (1:50, v/v) to afford **6d-1** as a colorless oil (71% yield).

^{1}H NMR (500 MHz, Chloroform-*d*) δ 8.01 (dd, *J* = 7.9, 1.7 Hz, 1H), 7.57 – 7.55 (m, 1H), 7.33 – 7.29 (m, 1H), 7.11 – 7.09 (m, 1H), 4.39 (t, *J* = 6.3 Hz, 2H), 2.37 – 2.34 (m, 5H), 2.00 – 1.93 (m, 3H);

^{13}C NMR (125 MHz, Chloroform-*d*) δ 169.6, 164.3, 150.7, 133.8, 131.7, 126.0, 123.8, 123.2, 82.9, 69.2, 63.6, 27.6, 21.0, 15.3;

IR (ATR): *v* = 2962, 1768, 1720, 1607, 1485, 1451, 1251, 1160, 915;

HRMS (ESI) Calculated for $C_{14}H_{15}O_4$ $[M+H]^+$: 247.0965; found 247.0965.

To a solution of **6d-1** (4 mmol, 1.0 equiv..) and CuI (0.4 mol, 10 mol%) in MeCN (20 mL) under N_2 atmosphere, diazocarbonyl compounds (6 mmol, 1.5 equiv..) are added. The mixture is stirred at room temperature overnight. After filtration and concentration under reduced pressure, the crude product is purified by column chromatography on silica gel with EA/PE (1:10, v/v) to afford **6d-2** as a pale-yellow oil (45% yield).

^{1}H NMR (500 MHz, Chloroform-*d*) δ 8.05 – 8.03 (m, 1H), 7.57 – 7.54 (m, 1H), 7.32 – 7.29 (m, 1H), 7.11 – 7.09 (m, 1H), 4.32 (t, *J* = 6.5 Hz, 2H), 4.20 (q, *J* = 7.1 Hz, 2H), 3.77 (s, 2H), 2.90 (t, *J* = 7.3 Hz, 2H), 2.35 (s, 3H), 2.11 – 2.04 (m, 2H), 1.28 (t, *J* = 7.1 Hz, 3H);

^{13}C NMR (125 MHz, Chloroform-*d*) δ 169.6, 168.1, 164.2, 150.78, 133.8, 131.7, 126.0, 124.7, 123.8, 123.2, 112.9, 63.4, 61.4, 46.9, 37.3, 26.6, 21.0, 14.1;

IR (ATR): *ν* = 2928, 1769, 1720, 1607, 1252, 1186, 1078, 1028;

HRMS (ESI) Calculated for $C_{18}H_{21}O_6$ $[M+H]^+$: 333.1333; found 333.1326.

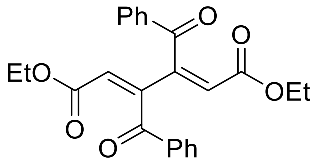

8

diethyl (2*Z*,4*Z*)-3,4-dibenzoylhexa-2,4-dienedioate

To a dried Schlenk tube, $(4\text{-}CF_3Ph)_3PAuCl$ (0.01mmol, 10 mol%), Selectfluor (0.25 mmol, 2.5 equiv.) and MeCN (2 mL) are added successively under air atmosphere. After that, **4a** (0.1 mmol, 1.0 equiv.) and water (0.2 mmol, 2.0 euqiv) are added by microinjector under air atmosphere. The resulting reaction mixture is heated at 50 °C for 12 hours and then cooled to room temperature and concentrated in vacuo. The resulting residue is purified by column chromatography on silica gel (eluent: petroleum ether/ethyl acetate = 10: 1) to give the final product **8** (19.3 mg, 95% yield, colorless oil).

^{1}H NMR (500 MHz, Chloroform-*d*) δ 8.03 – 7.98 (m, 4H), 7.65 – 7.62 (m, 2H), 7.52 (t, *J* = 7.8 Hz, 4H), 6.07 (s, 2H), 3.97 (q, *J* = 7.2 Hz, 4H), 1.03 (t, *J* = 7.2 Hz, 6H).

^{13}C NMR (125 MHz, Chloroform-*d*) δ 194.3, 163.9, 149.9, 135.5, 134.2, 129.0, 128.9,

125.3, 61.5, 13.7.

IR (ATR): *v* = 2982, 2927, 1713, 1672, 1596, 1308, 1234, 1041, 957, 868;

HRMS (ESI) Calculated for $C_{24}H_{23}O_6$ $[M+H]^+$: 407.1489; found 407.1483.

8. NMR spectra

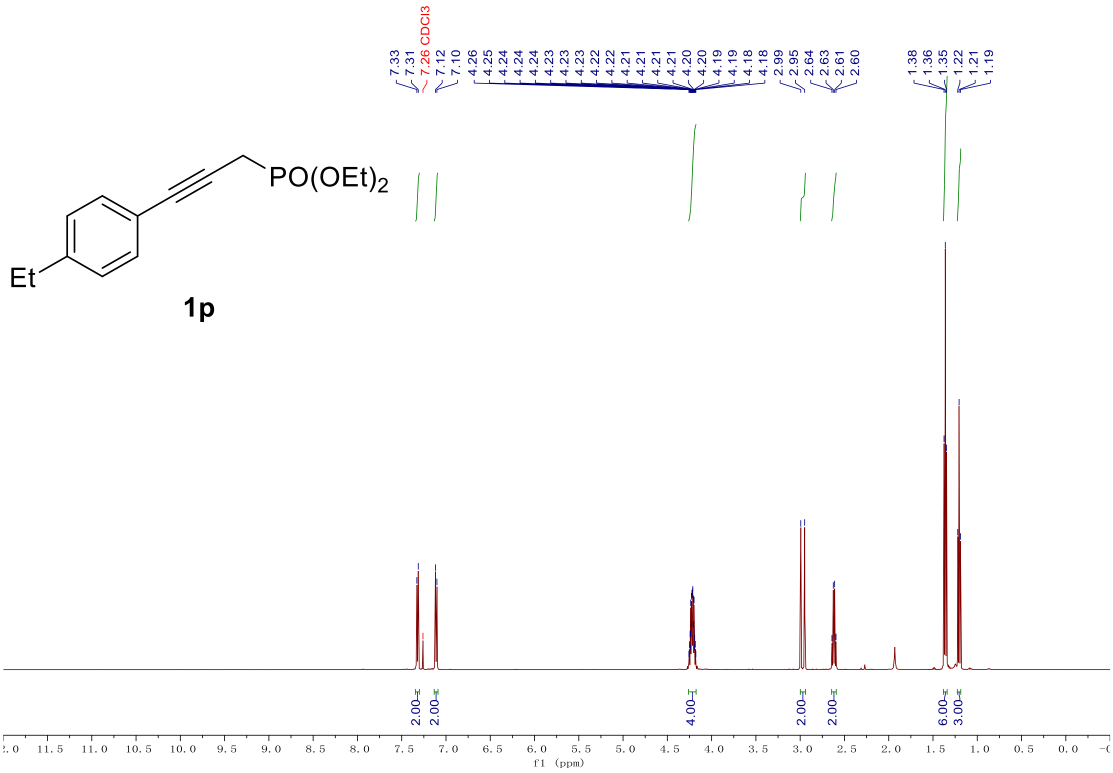

Supplementary Figure 9. ^{1}H NMR (500 MHz, $CDCl_3$) spectrum for compound **1p**

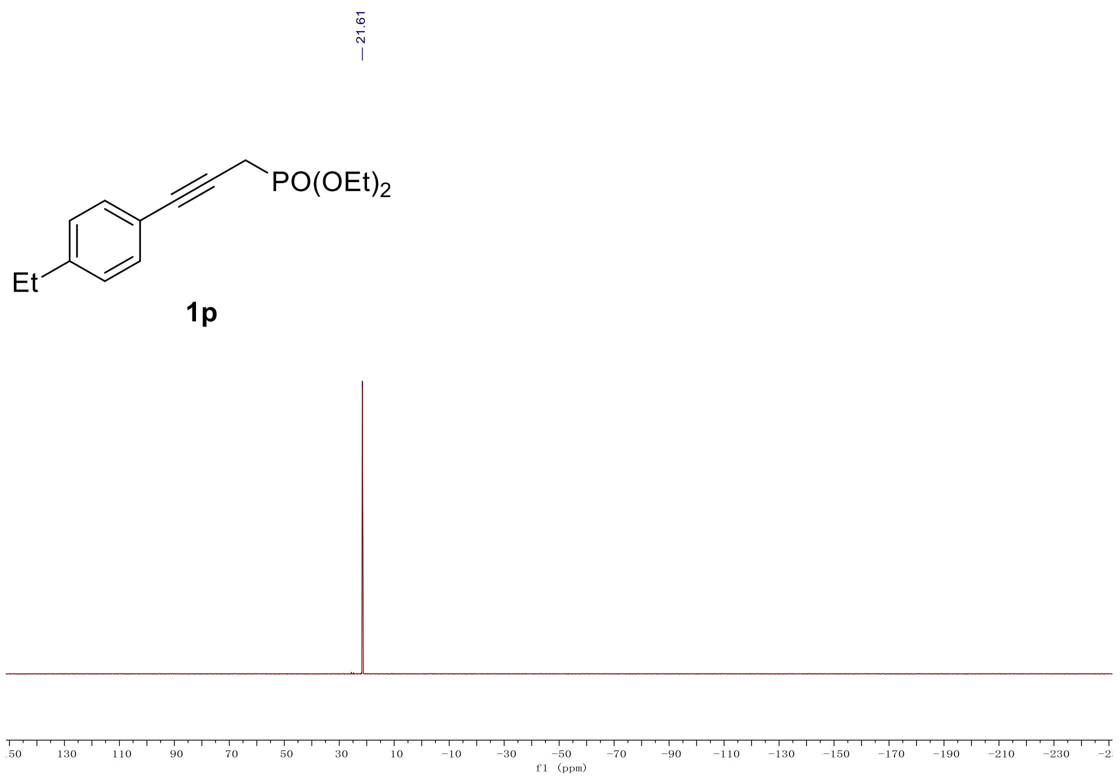

Supplementary Figure 10. ^{31}P NMR (202 MHz, $CDCl_3$) spectrum for compound **1p**

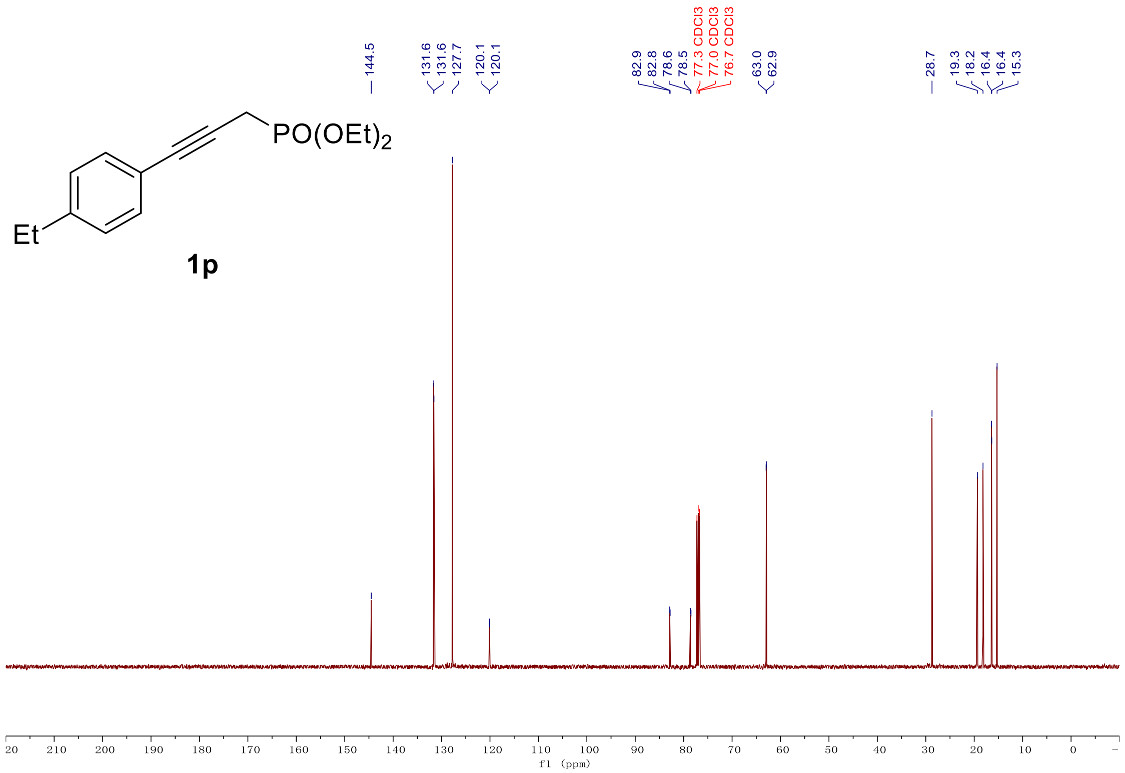

Supplementary Figure 11. ^{13}C NMR (125 MHz, $CDCl_3$) spectrum for compound **1p**

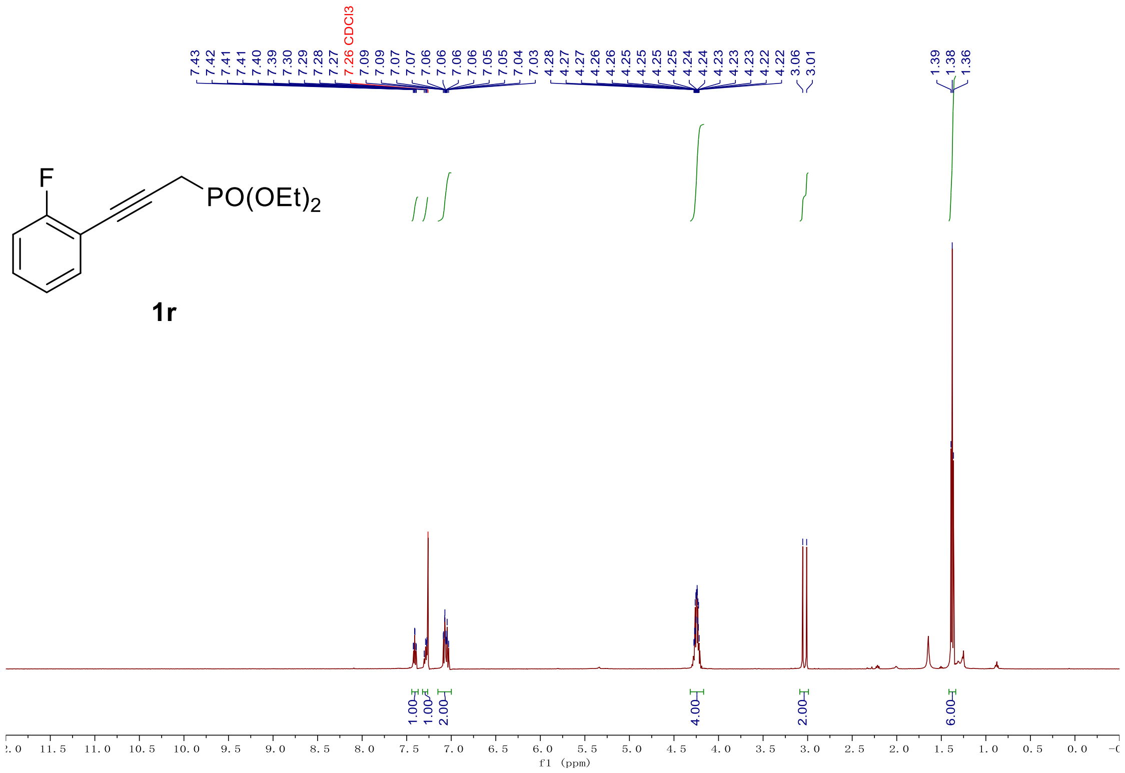

Supplementary Figure 12. ^{1}H NMR (500 MHz, $CDCl_3$) spectrum for compound **1r**

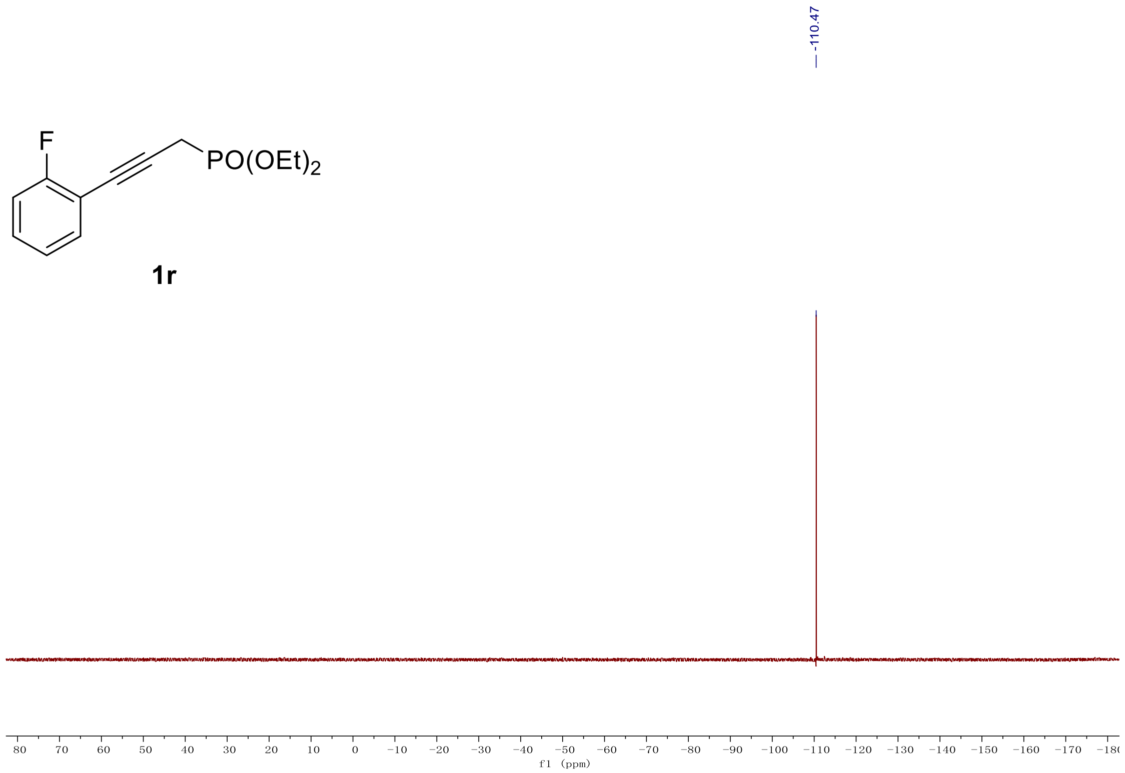

Supplementary Figure 13. ^{19}F NMR (471 MHz, $CDCl_3$) spectrum for compound **1r**

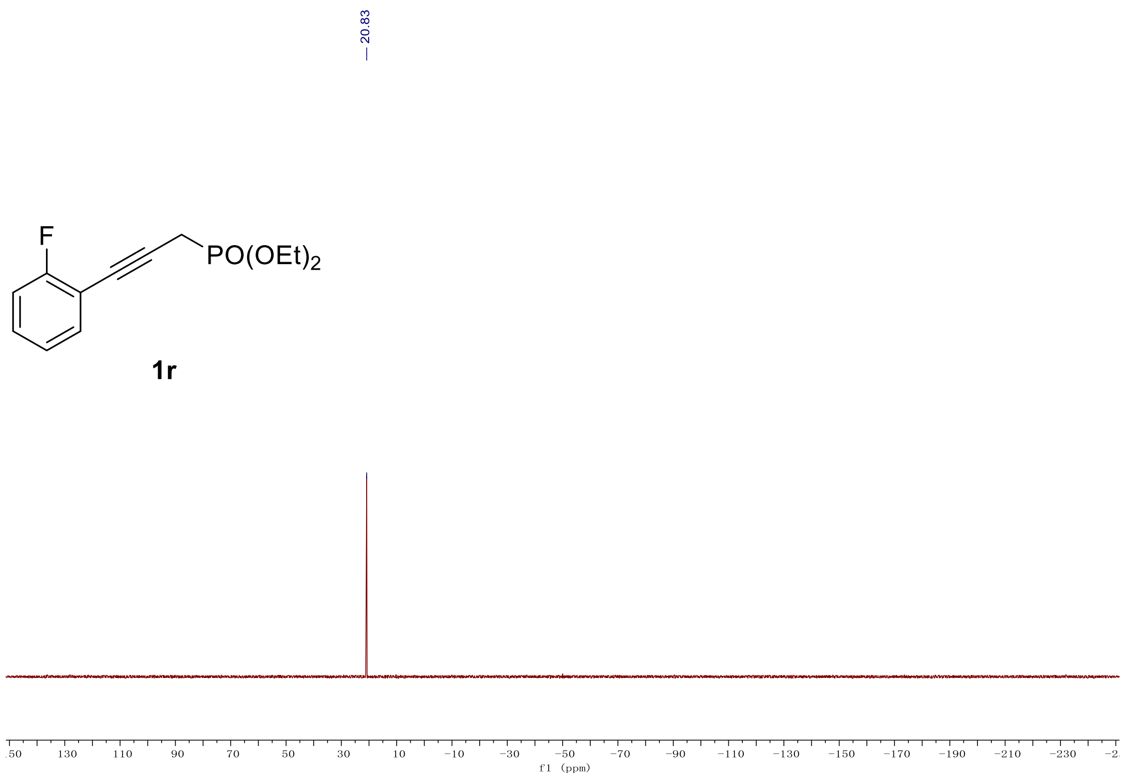

Supplementary Figure 14. ^{31}P NMR (202 MHz, CDCl3) spectrum for compound **1r**

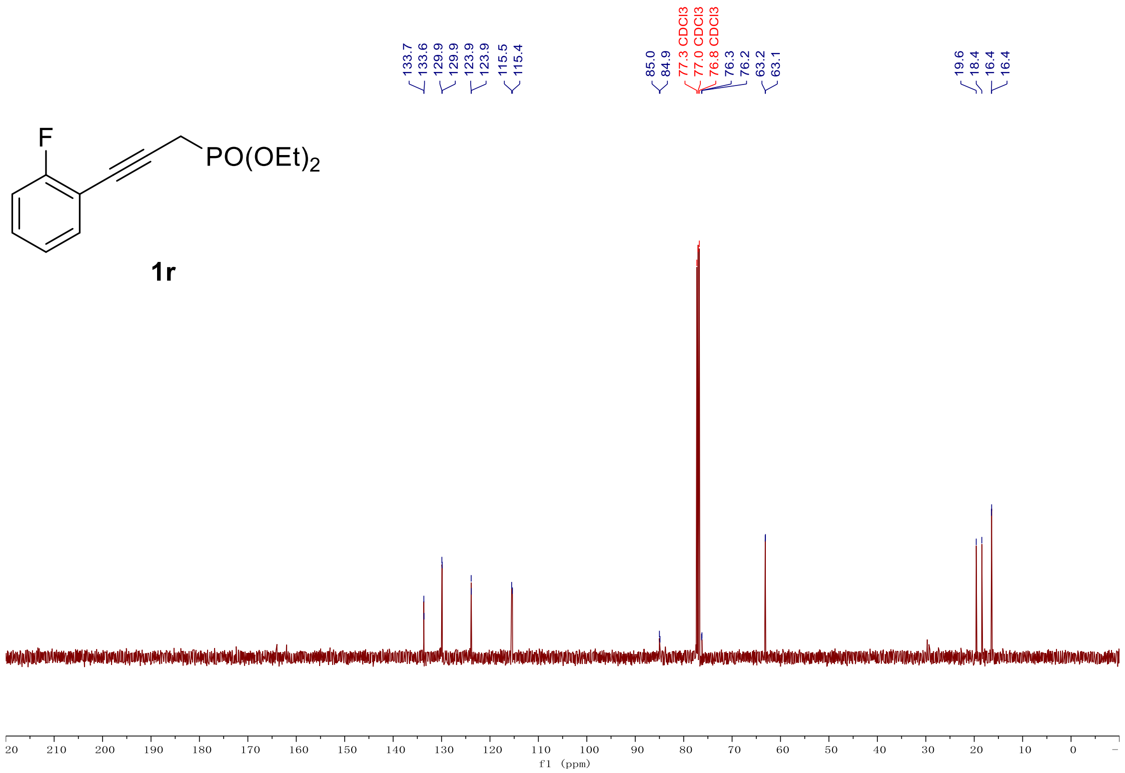

Supplementary Figure 15. ^{13}C NMR (125 MHz, CDCl3) spectrum for compound **1r**

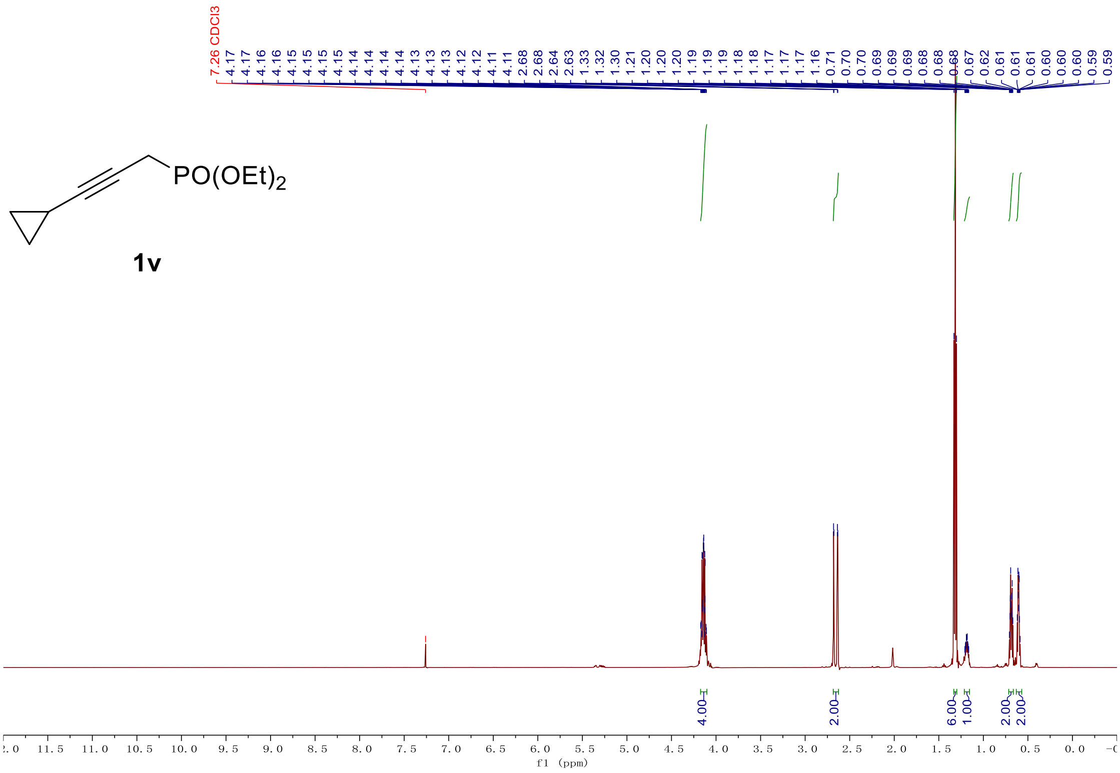

Supplementary Figure 16. ^{1}H NMR (500 MHz, CDCl3) spectrum for compound **1v**

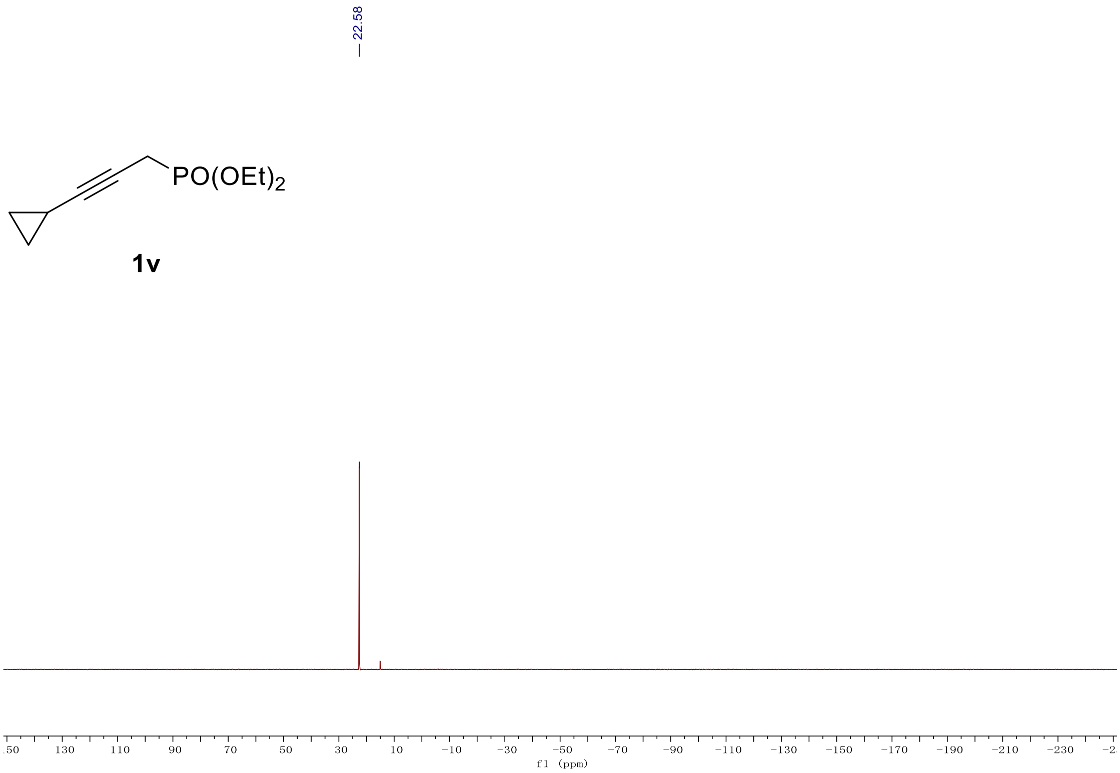

Supplementary Figure 17. ^{31}P NMR (202 MHz, CDCl3) spectrum for compound **1v**

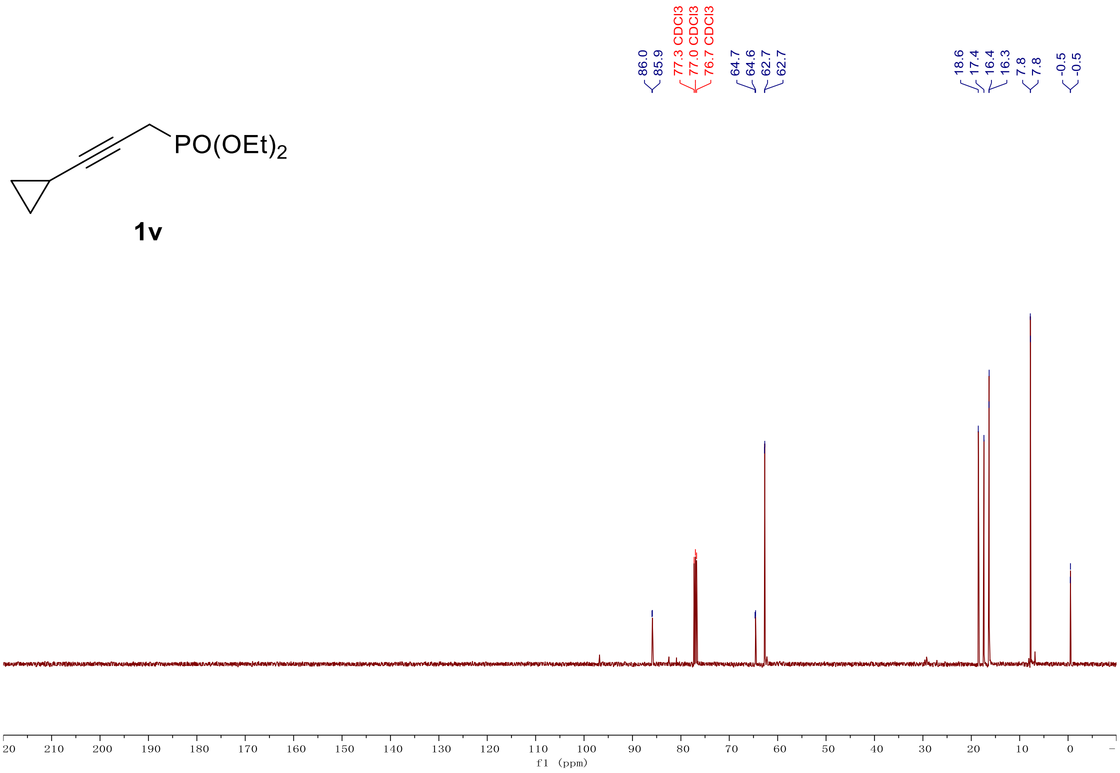

Supplementary Figure 18. ^{13}C NMR (125 MHz, CDCl$_3$) spectrum for compound **1v**

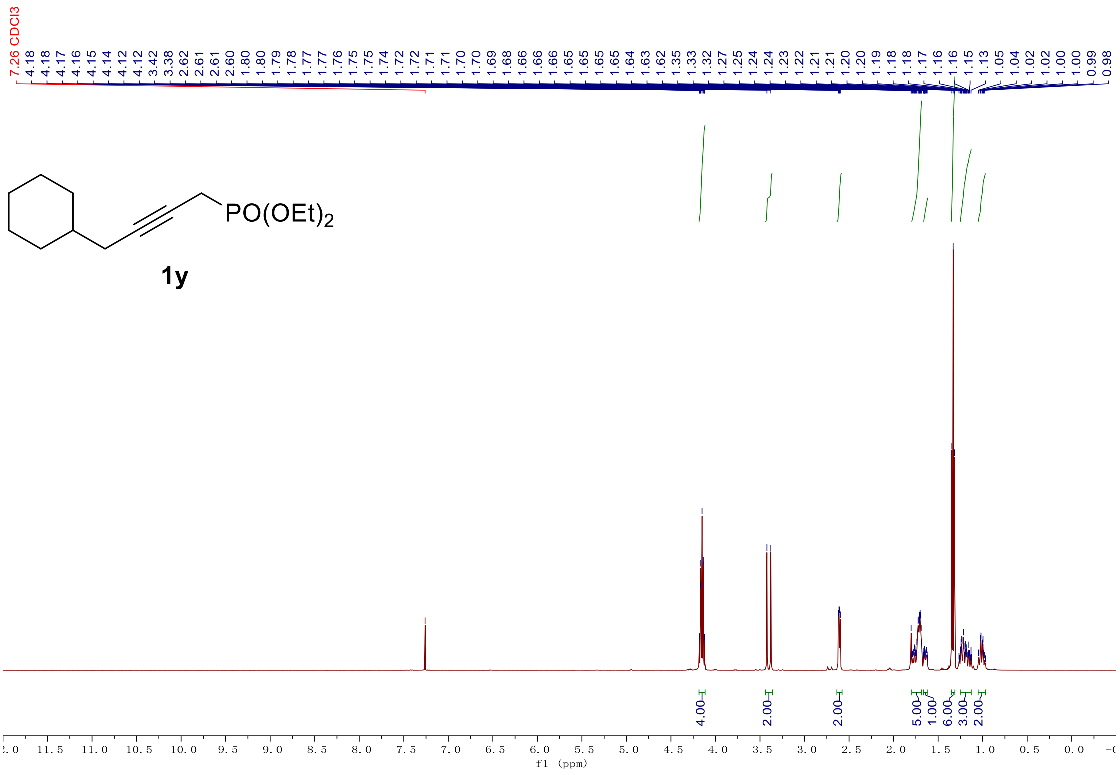

Supplementary Figure 19. ^{1}H NMR (500 MHz, CDCl$_3$) spectrum for compound **1y**

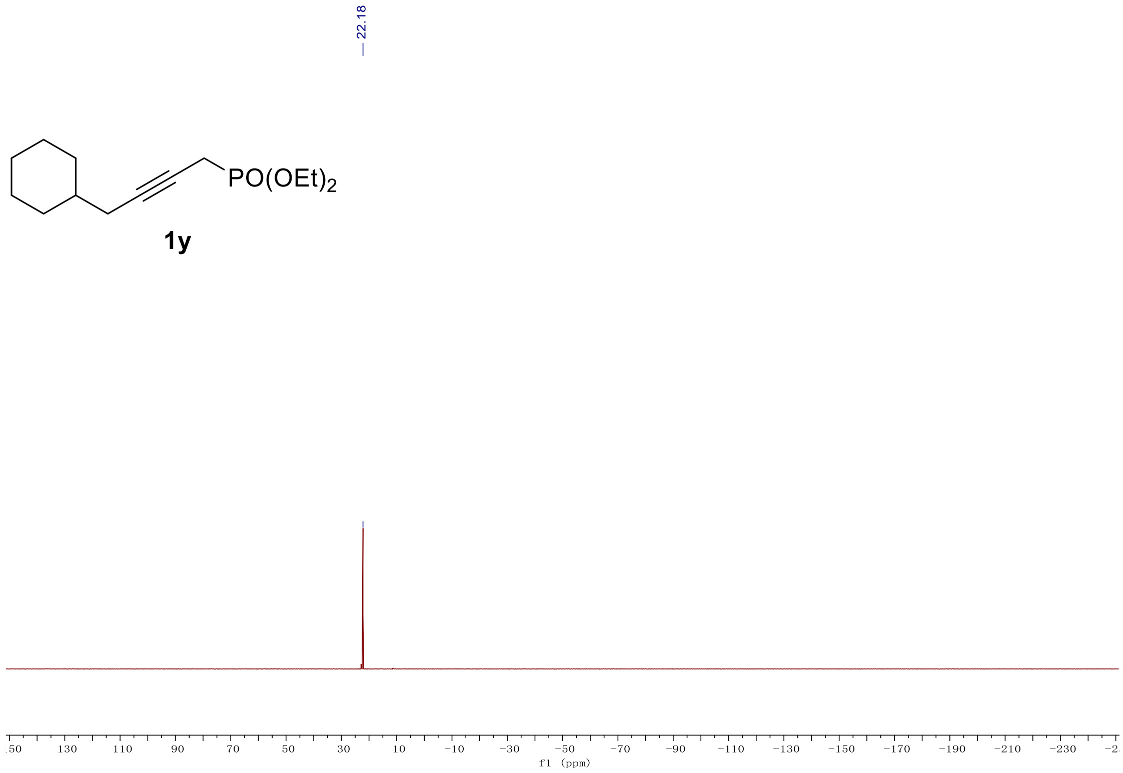

Supplementary Figure 20. ^{31}P NMR (202 MHz, $CDCl_3$) spectrum for compound **1y**

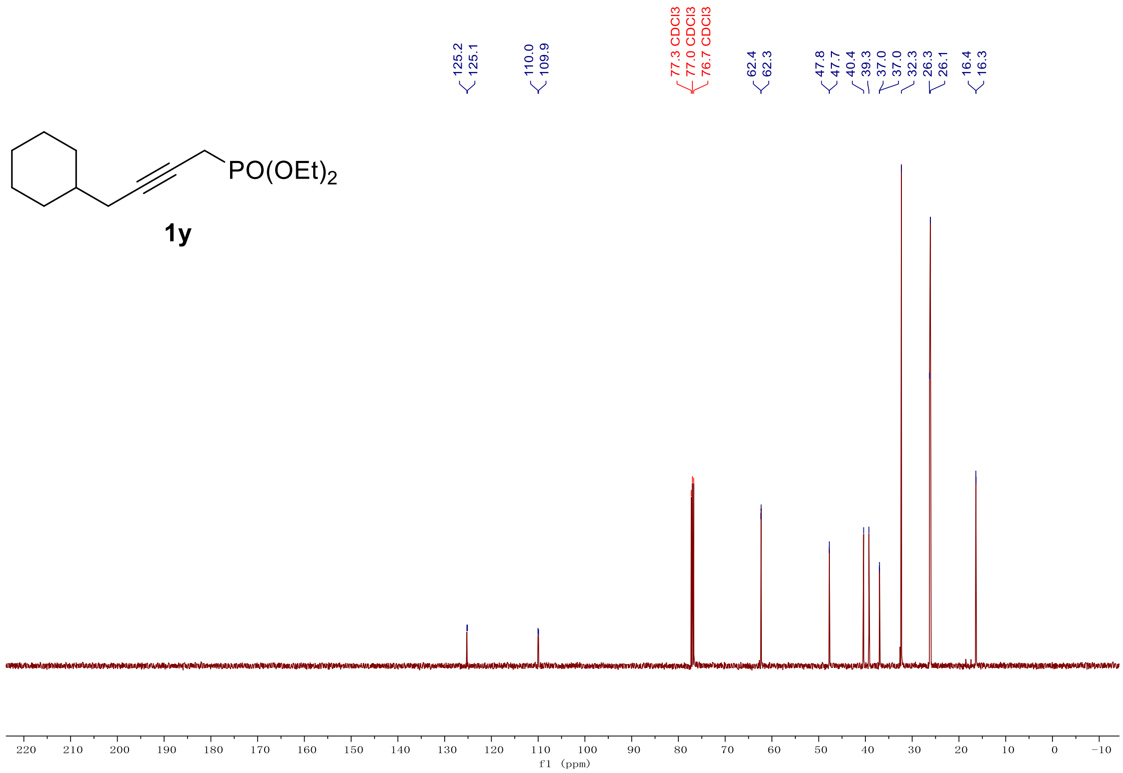

Supplementary Figure 21. ^{13}C NMR (125 MHz, $CDCl_3$) spectrum for compound **1y**

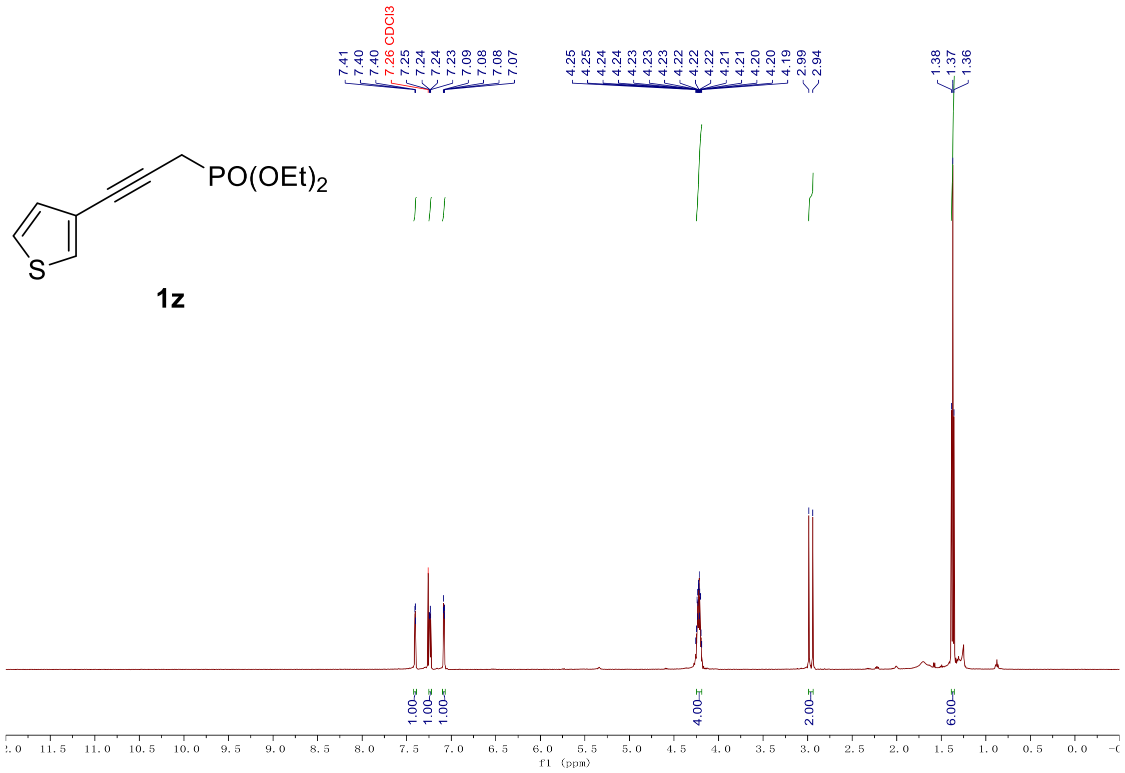

Supplementary Figure 22. ^{1}H NMR (500 MHz, $CDCl_3$) spectrum for compound **1z**

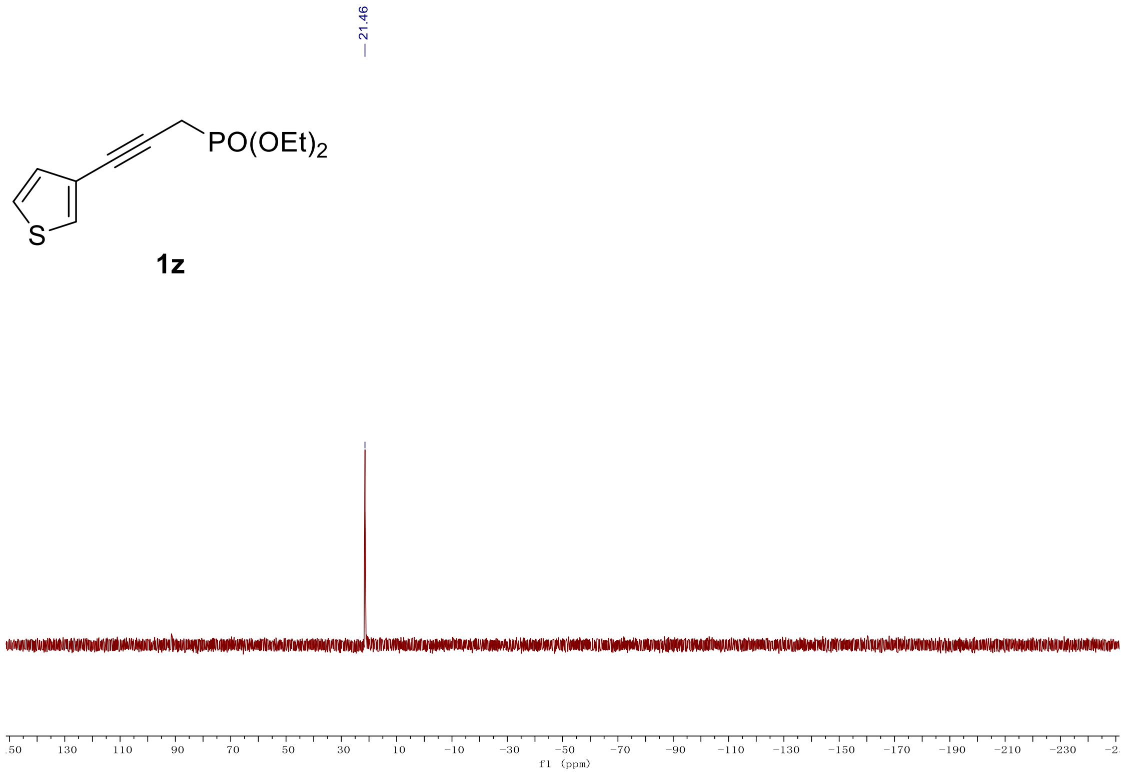

Supplementary Figure 23. ^{31}P NMR (202 MHz, $CDCl_3$) spectrum for compound **1z**

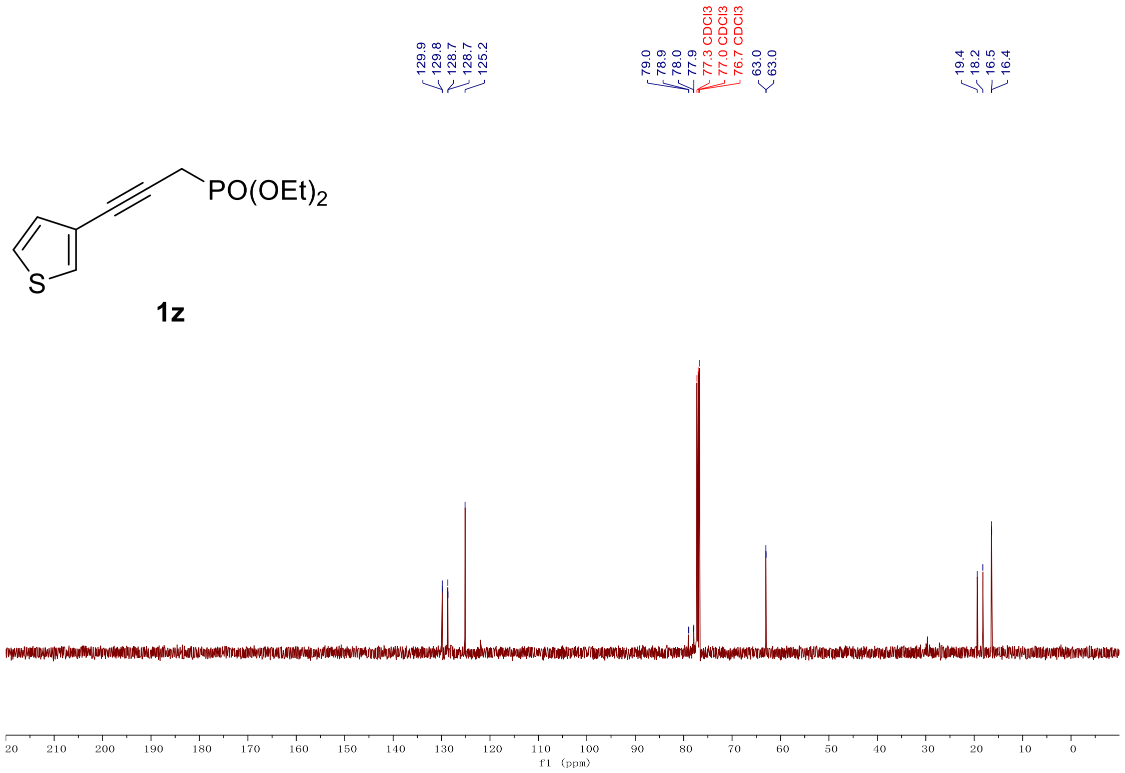

Supplementary Figure 24. ^{13}C NMR (125 MHz, $CDCl_3$) spectrum for compound **1z**

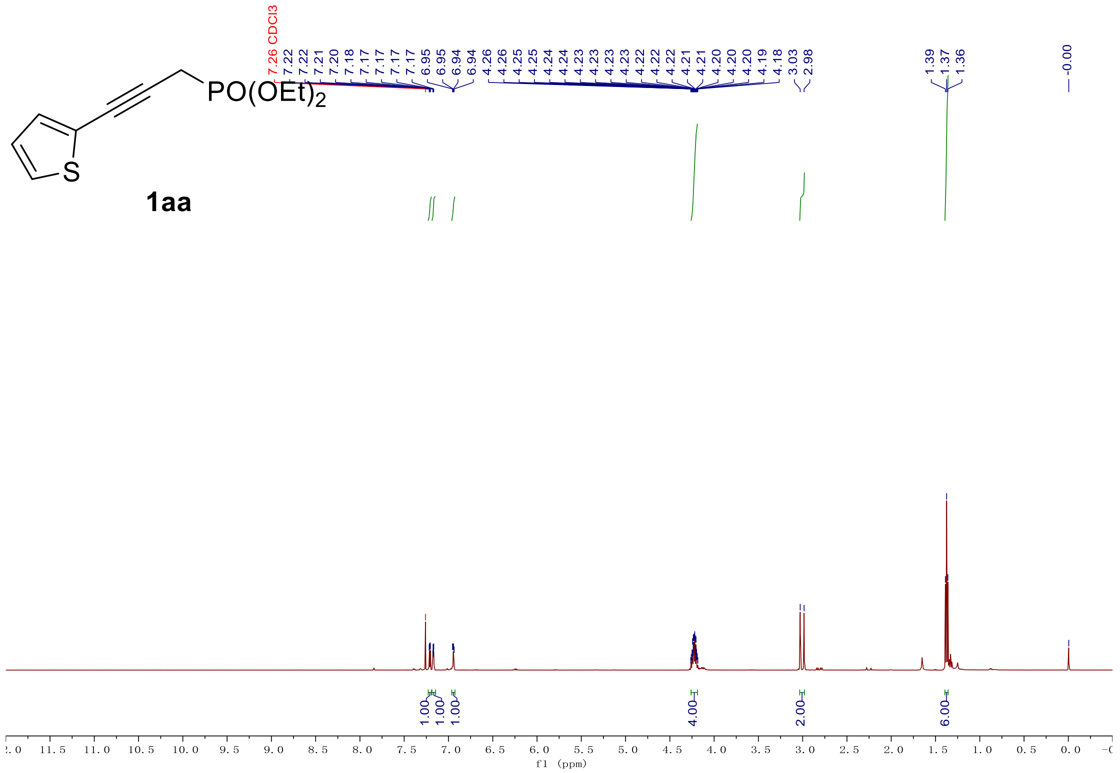

Supplementary Figure 25. ^{1}H NMR (500 MHz, $CDCl_3$) spectrum for compound **1aa**

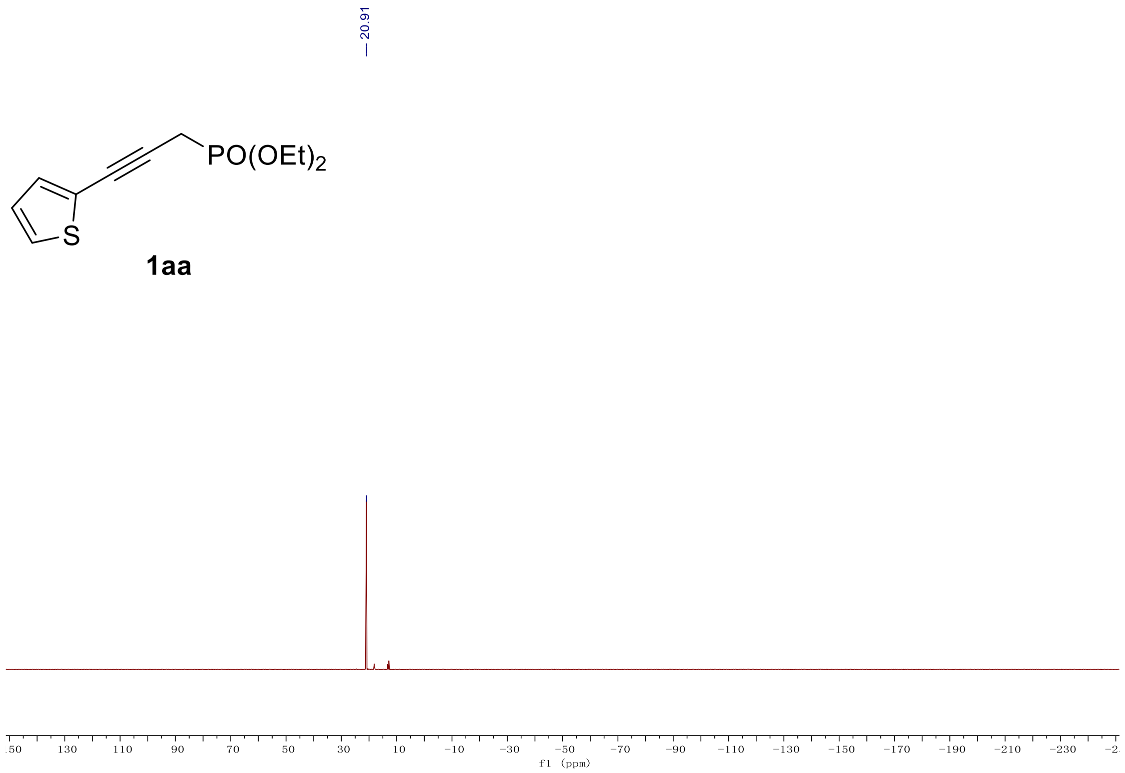

Supplementary Figure 26. ^{31}P NMR (202 MHz, $CDCl_3$) spectrum for compound **1aa**

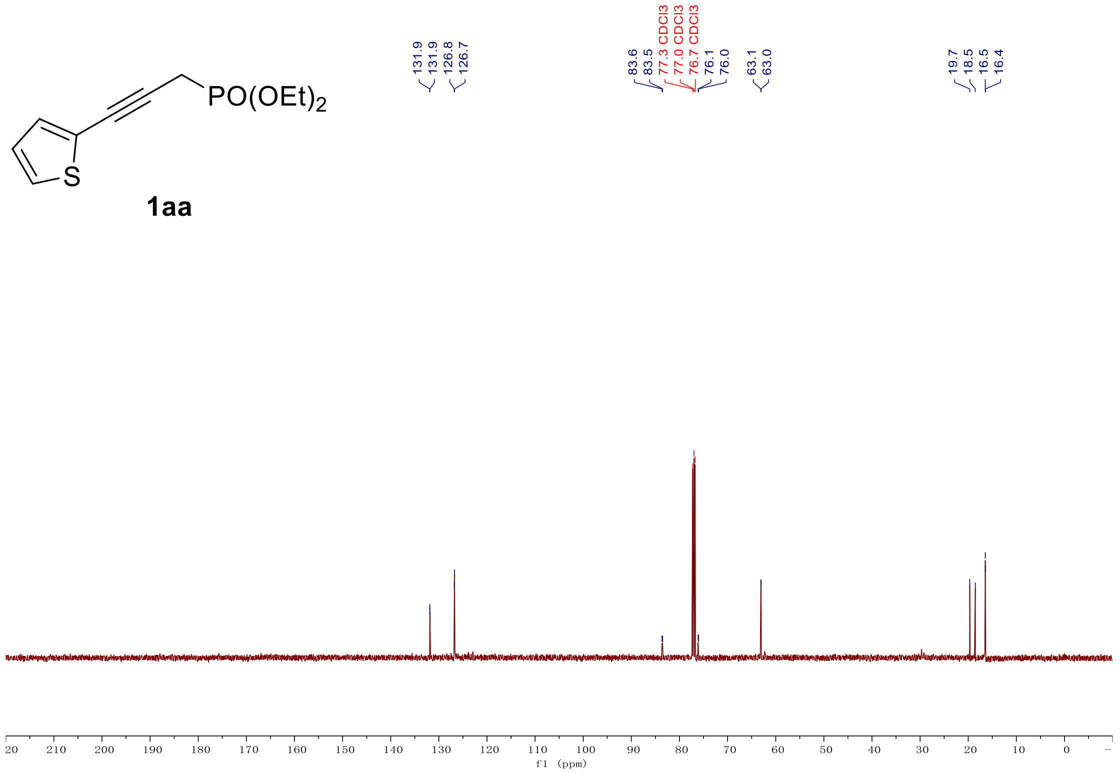

Supplementary Figure 27. ^{13}C NMR (125 MHz, $CDCl_3$) spectrum for compound **1aa**

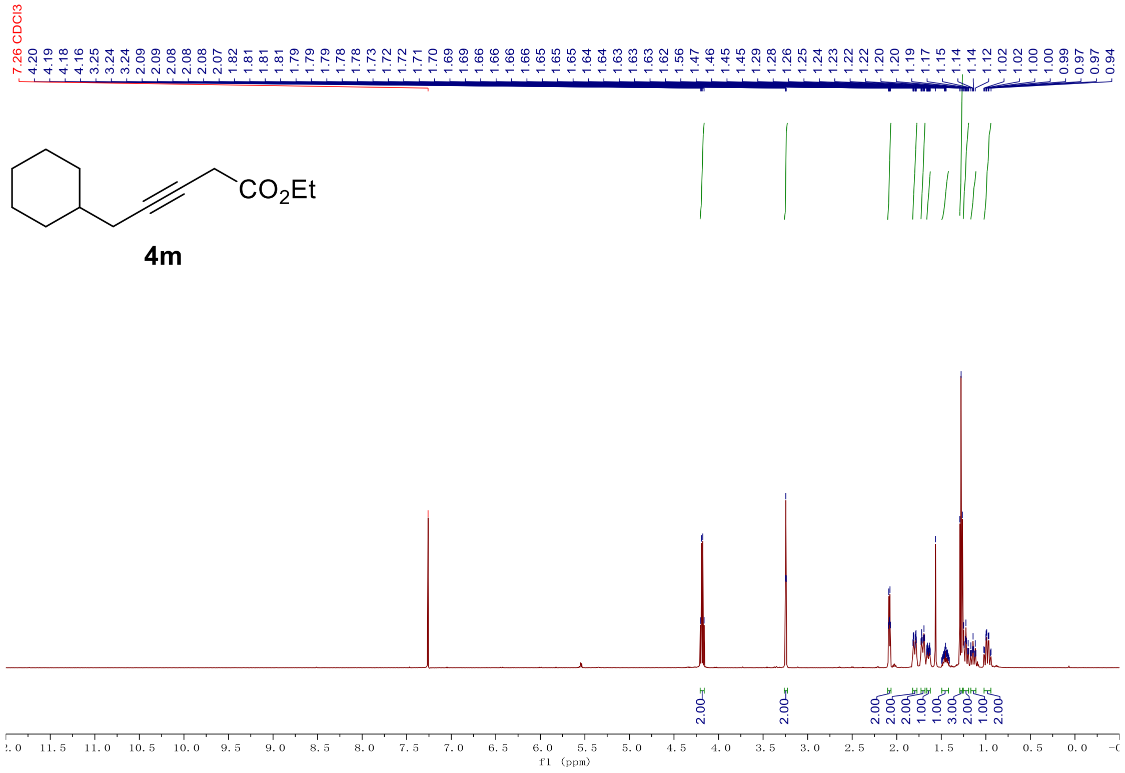

Supplementary Figure 28. ^{1}H NMR (500 MHz, CDCl3) spectrum for compound **4m**

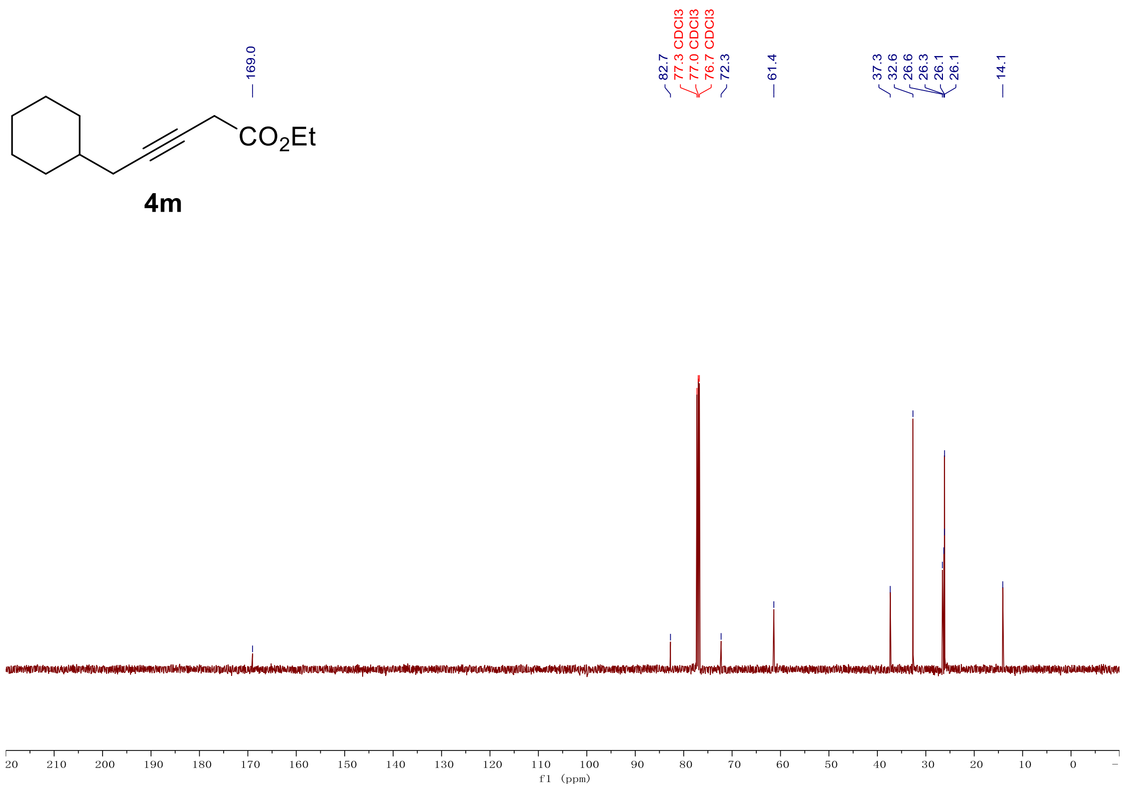

Supplementary Figure 29. ^{13}C NMR (125 MHz, CDCl3) spectrum for compound **4m**

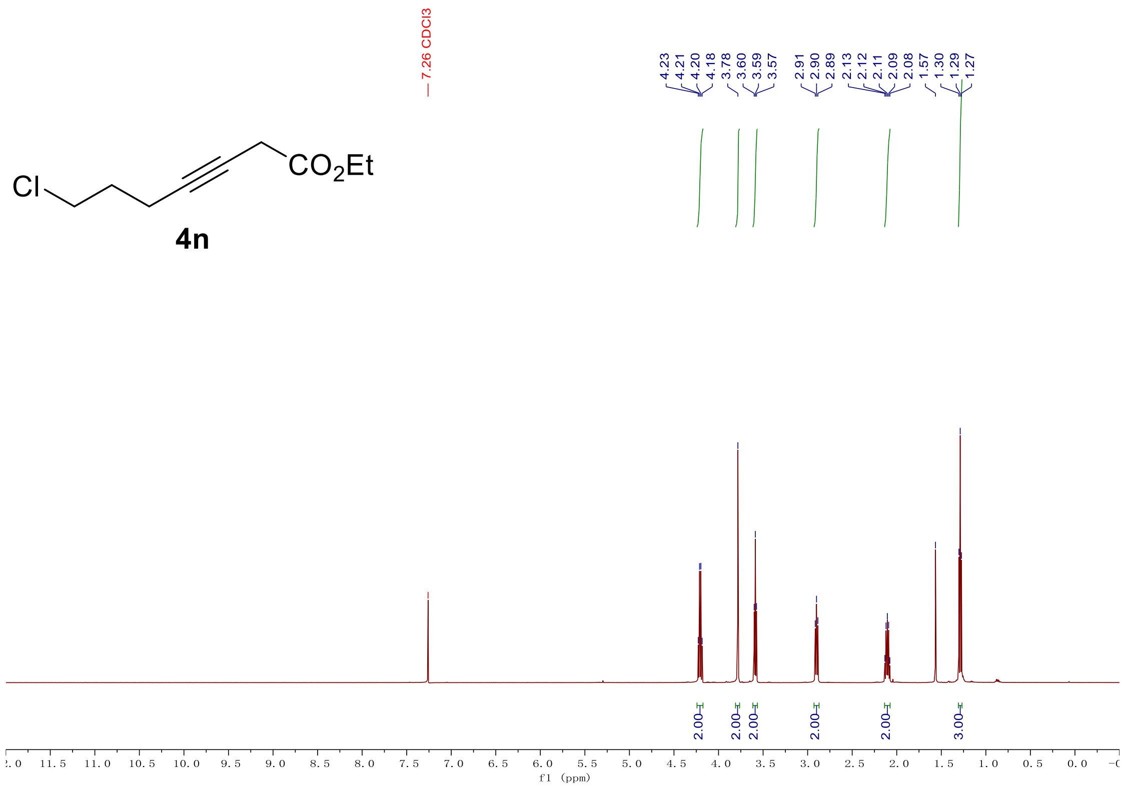

Supplementary Figure 30. ^{1}H NMR (500 MHz, $CDCl_3$) spectrum for compound **4n**

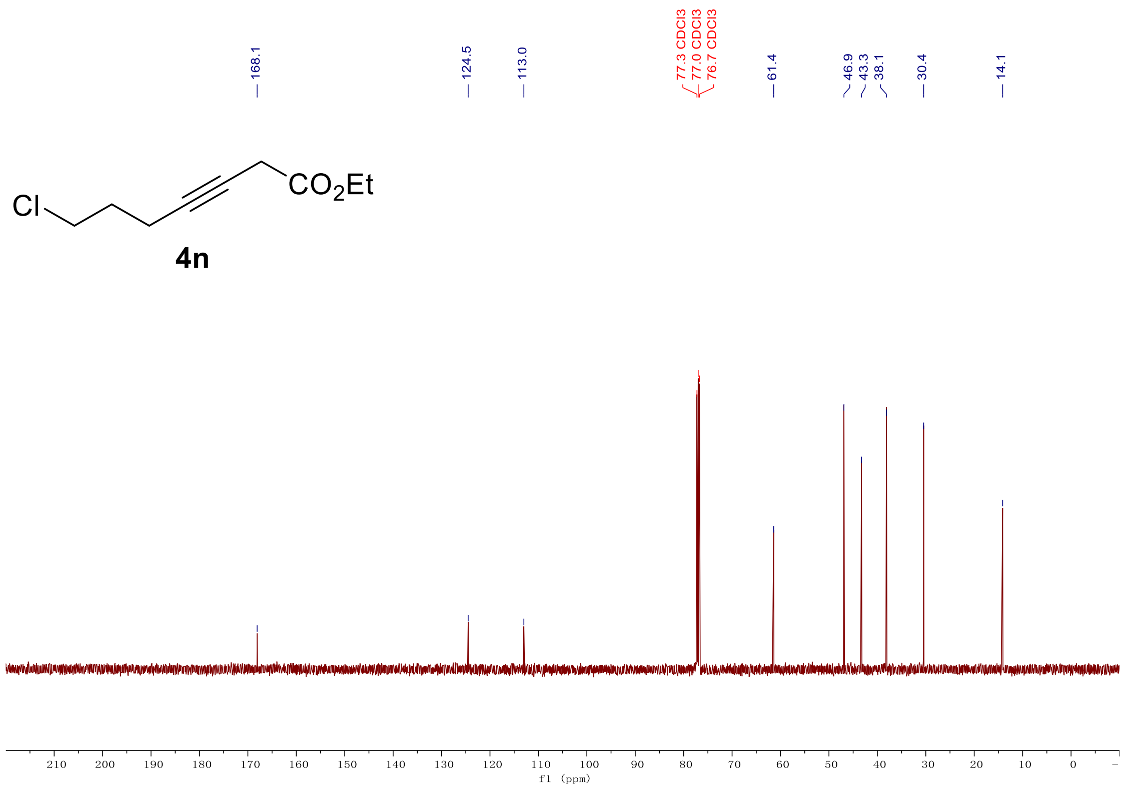

Supplementary Figure 31. ^{13}C NMR (125 MHz, $CDCl_3$) spectrum for compound **4n**

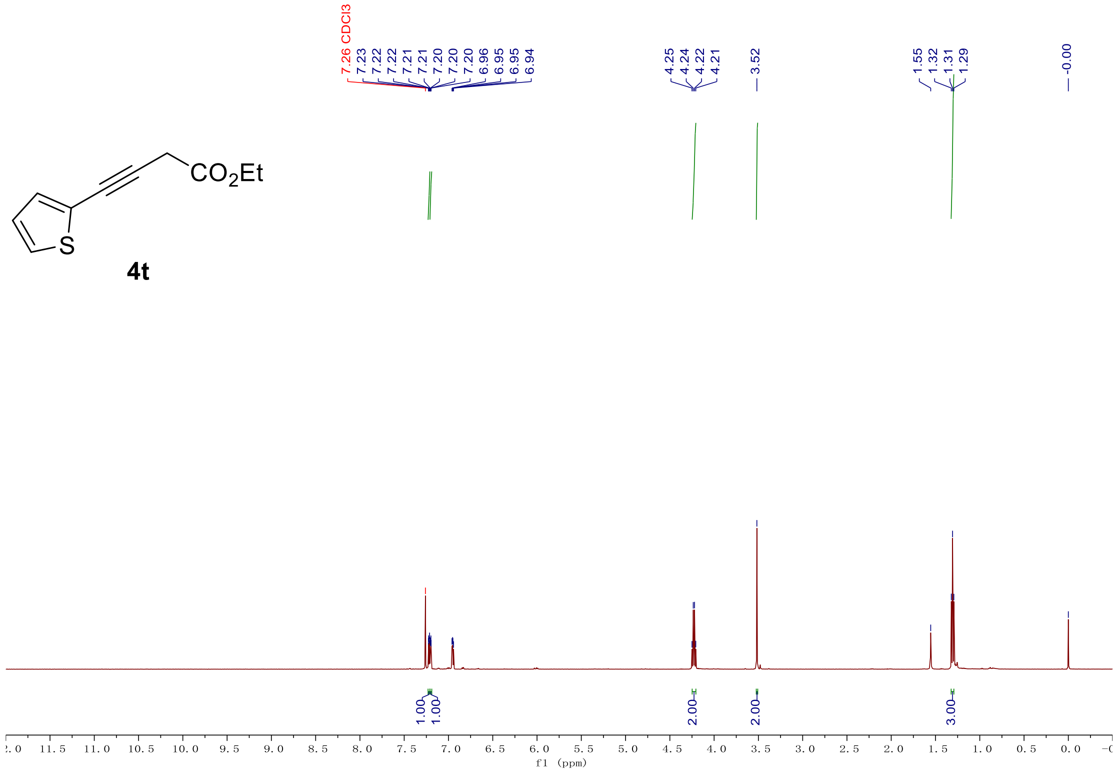

Supplementary Figure 32. ^{1}H NMR (500 MHz, $CDCl_3$) spectrum for compound **4t**

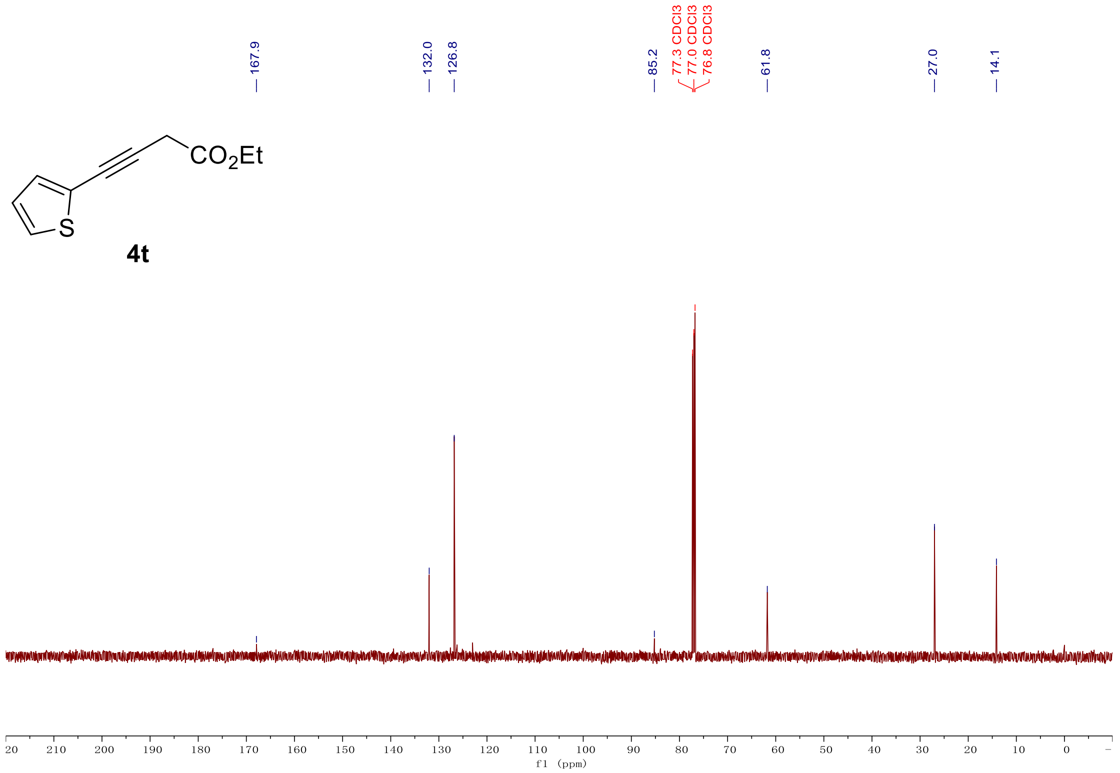

Supplementary Figure 33. ^{13}C NMR (125 MHz, $CDCl_3$) spectrum for compound **4t**

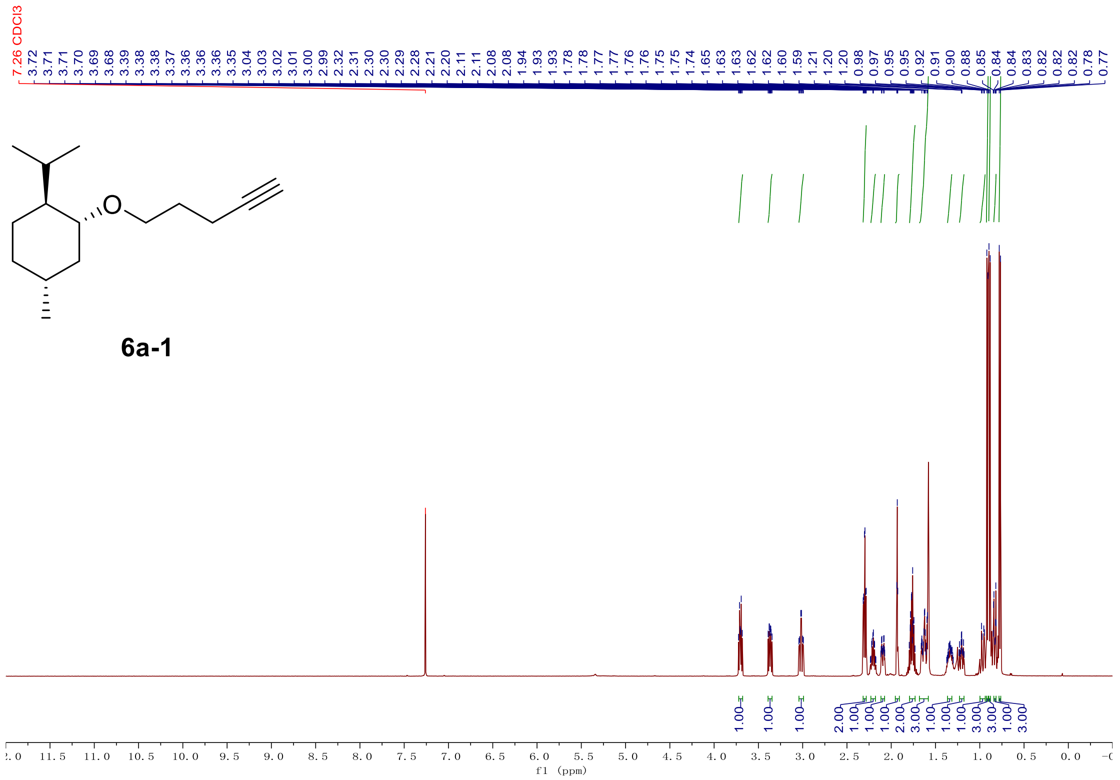

Supplementary Figure 34. ^{1}H NMR (500 MHz, $CDCl_3$) spectrum for compound **6a-1**

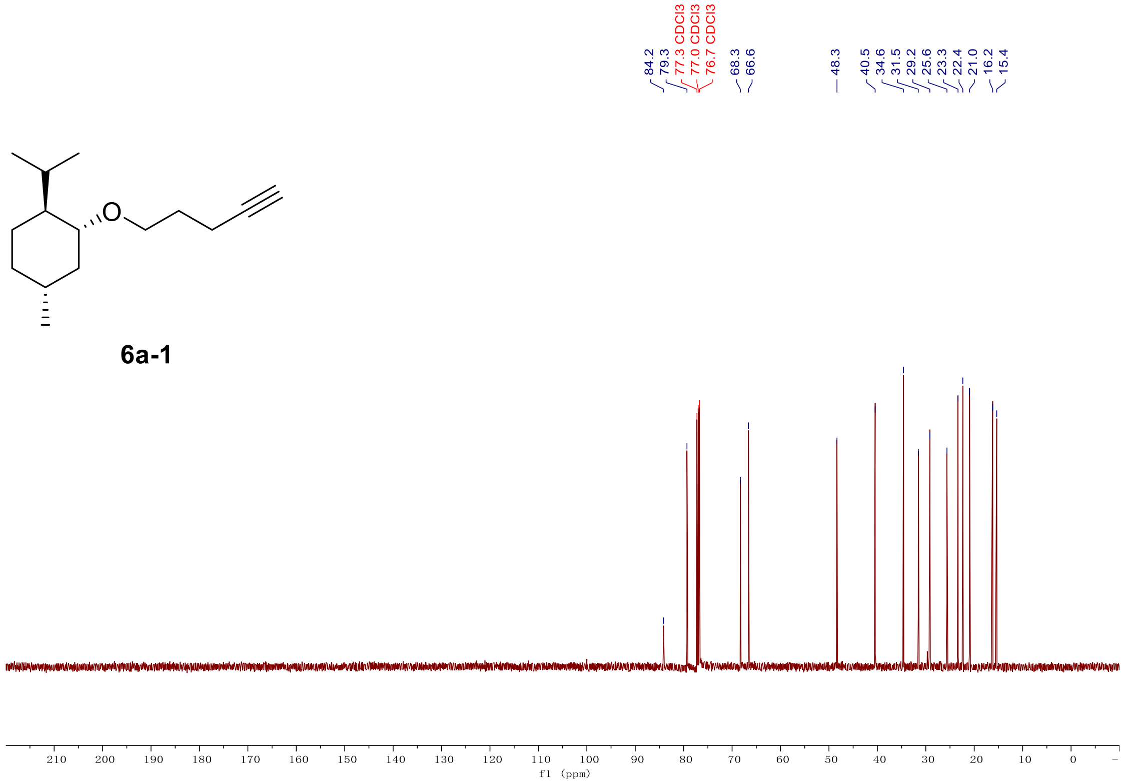

Supplementary Figure 35. ^{13}C NMR (125 MHz, $CDCl_3$) spectrum for compound **6a-1**

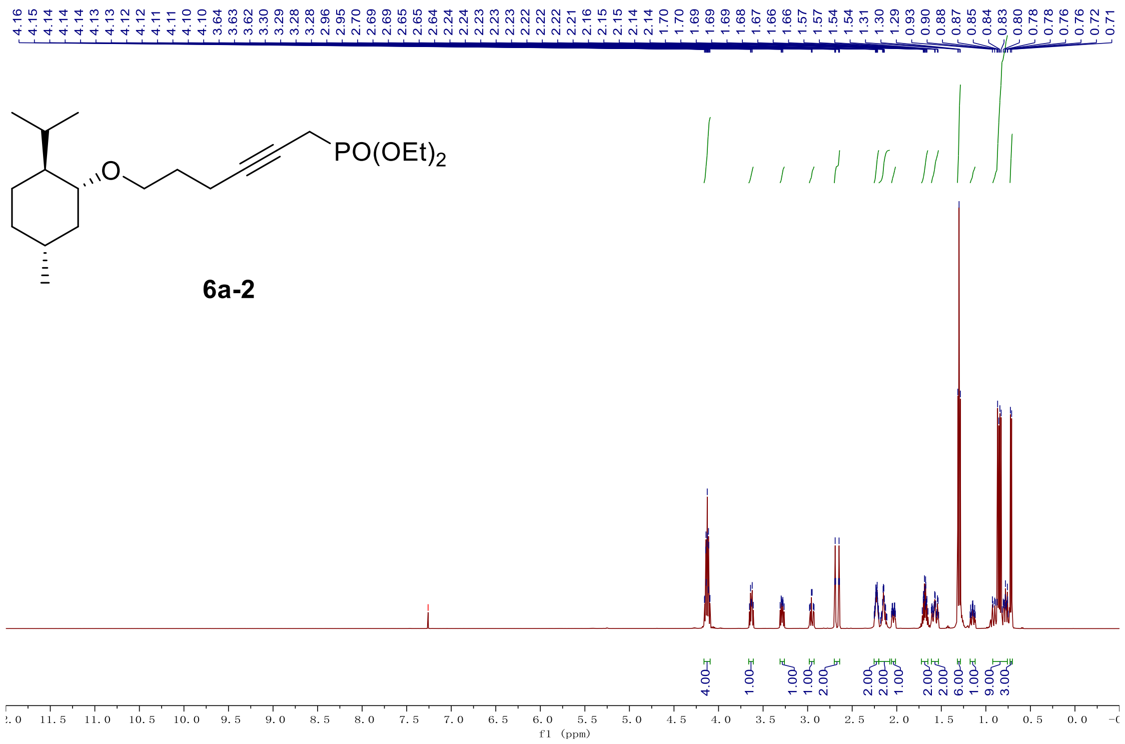

Supplementary Figure 36. ^{1}H NMR (500 MHz, $CDCl_3$) spectrum for compound **6a-2**

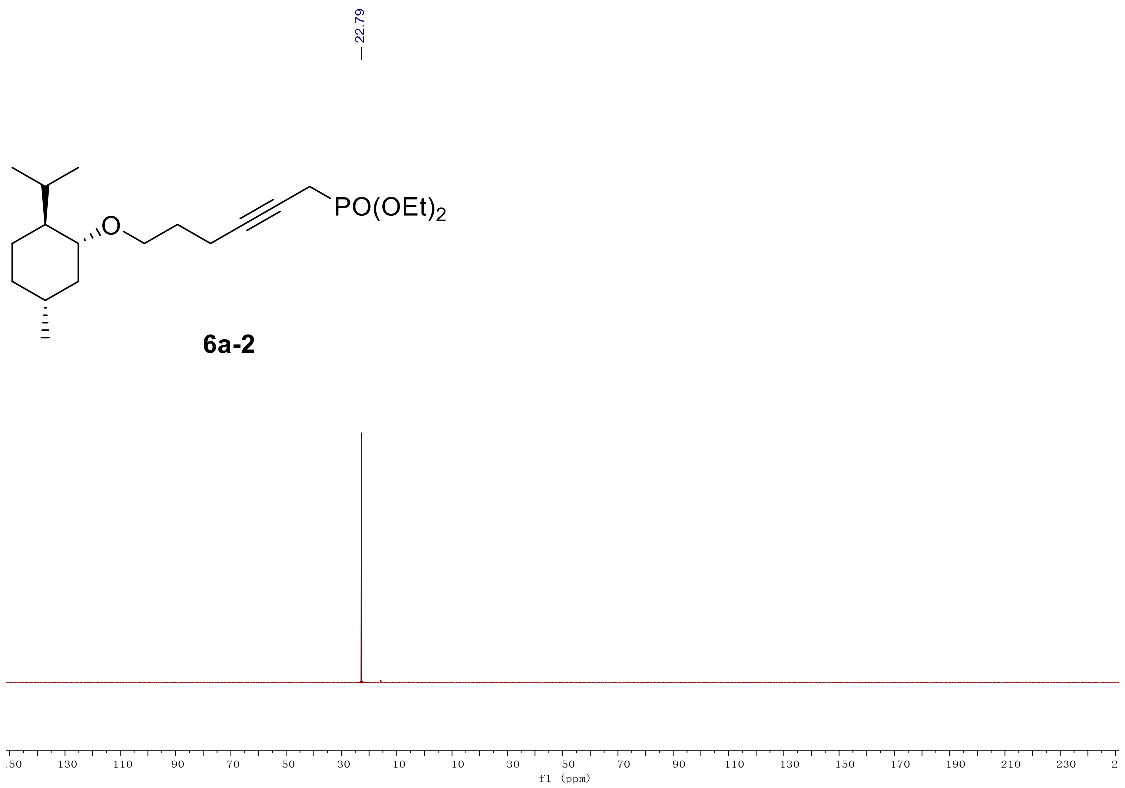

Supplementary Figure 37. ^{31}P NMR (202 MHz, $CDCl_3$) spectrum for compound **6a-2**

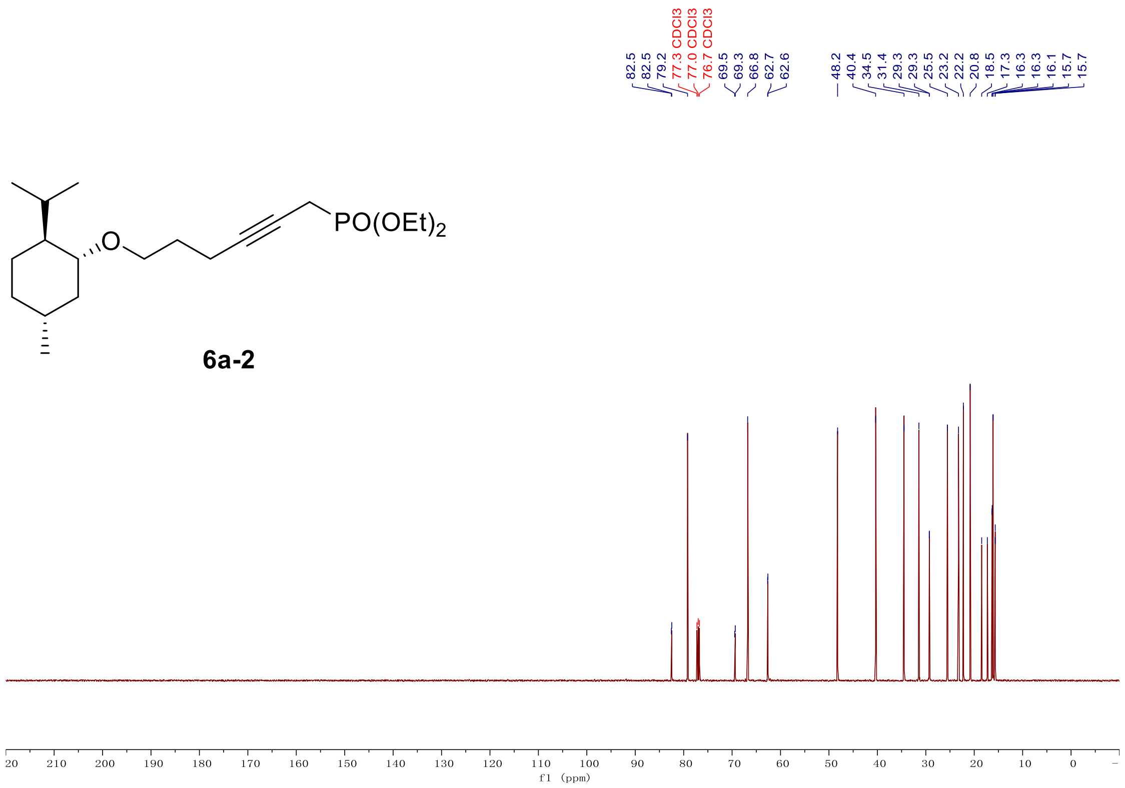

Supplementary Figure 38. ^{13}C NMR (125 MHz, $CDCl_3$) spectrum for compound **6a-2**

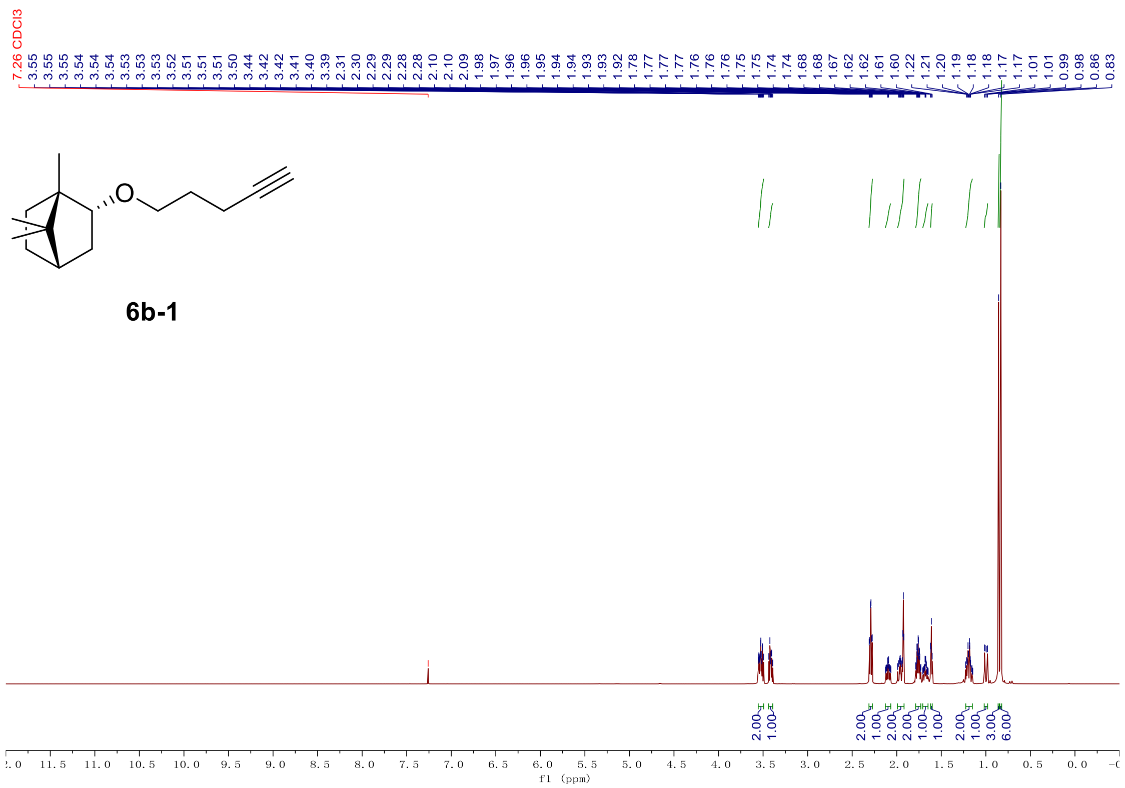

Supplementary Figure 39. ^{1}H NMR (500 MHz, $CDCl_3$) spectrum for compound **6b-1**

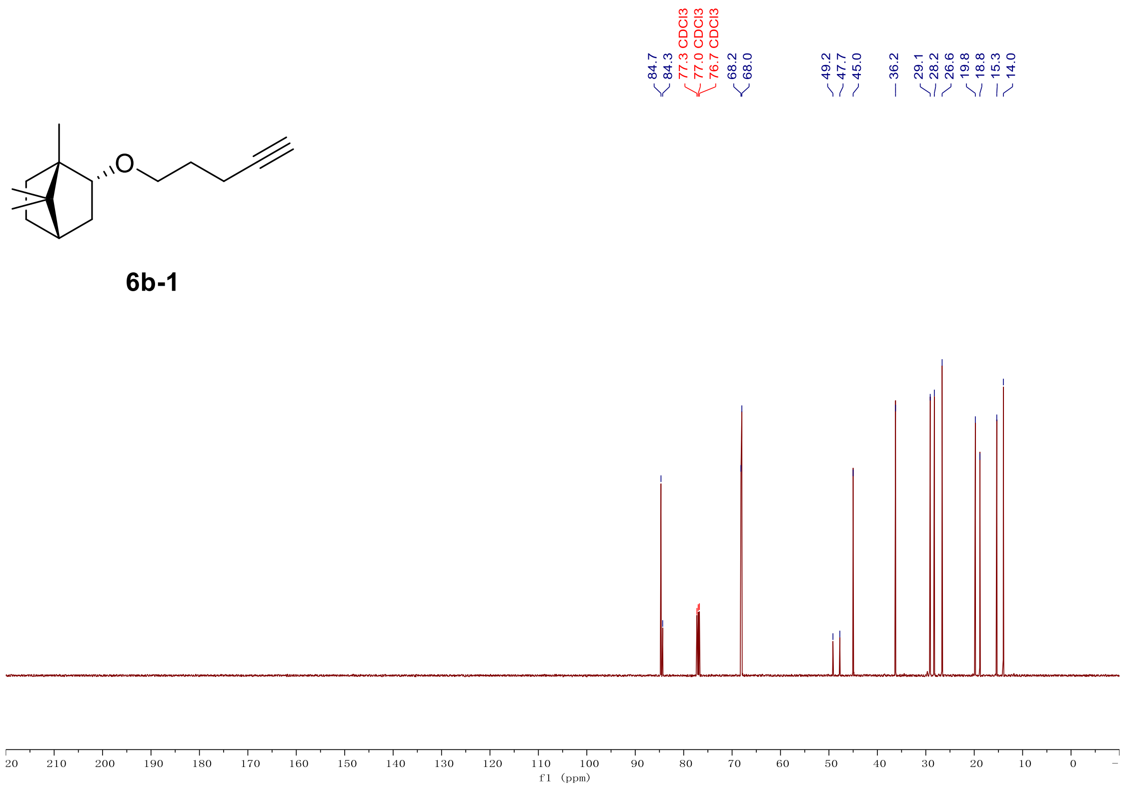

Supplementary Figure 40. ^{13}C NMR (125 MHz, $CDCl_3$) spectrum for compound **6b-1**

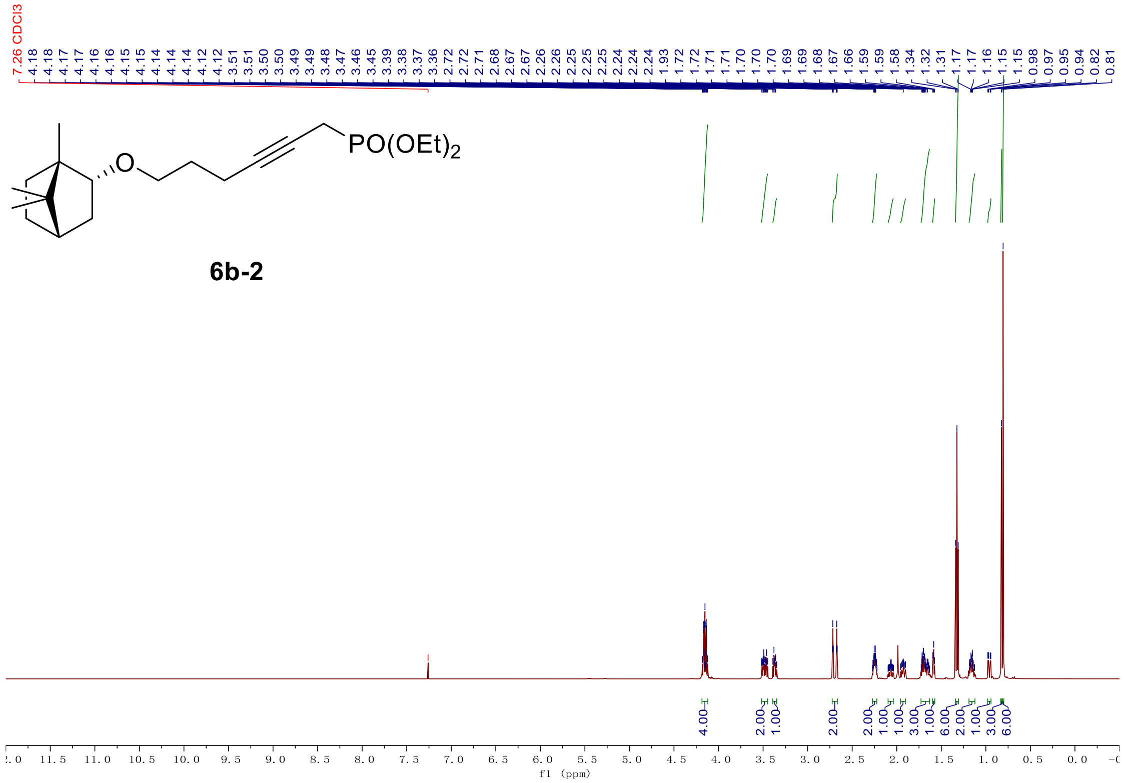

Supplementary Figure 41. ^{1}H NMR (500 MHz, $CDCl_3$) spectrum for compound **6b-2**

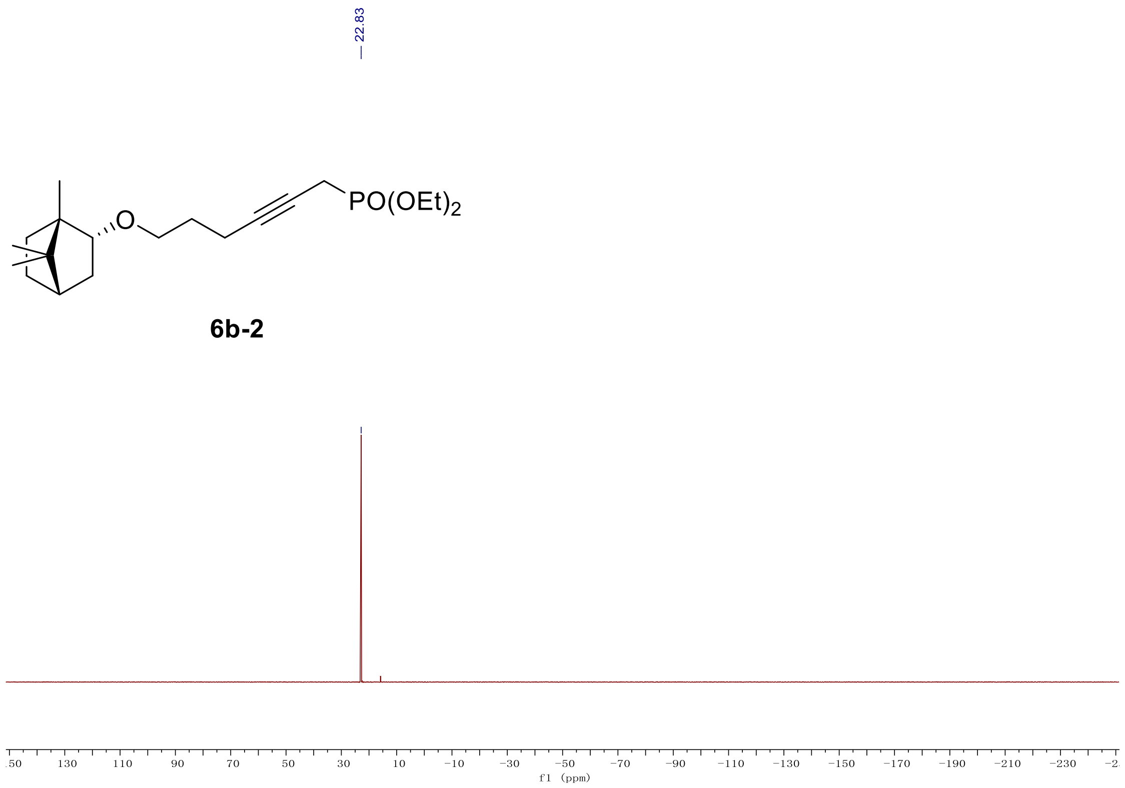

Supplementary Figure 42. ^{31}P NMR (202 MHz, $CDCl_3$) spectrum for compound **6b-2**

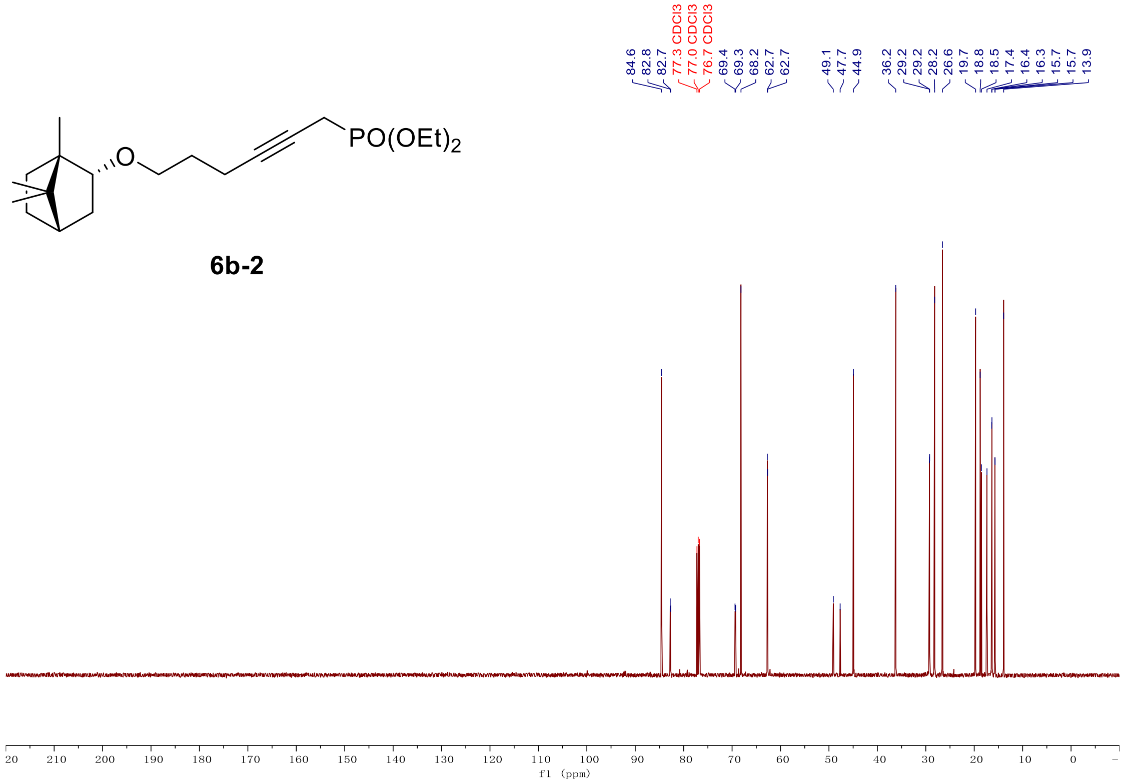

Supplementary Figure 43. ^{13}C NMR (125 MHz, $CDCl_3$) spectrum for compound **6b-2**

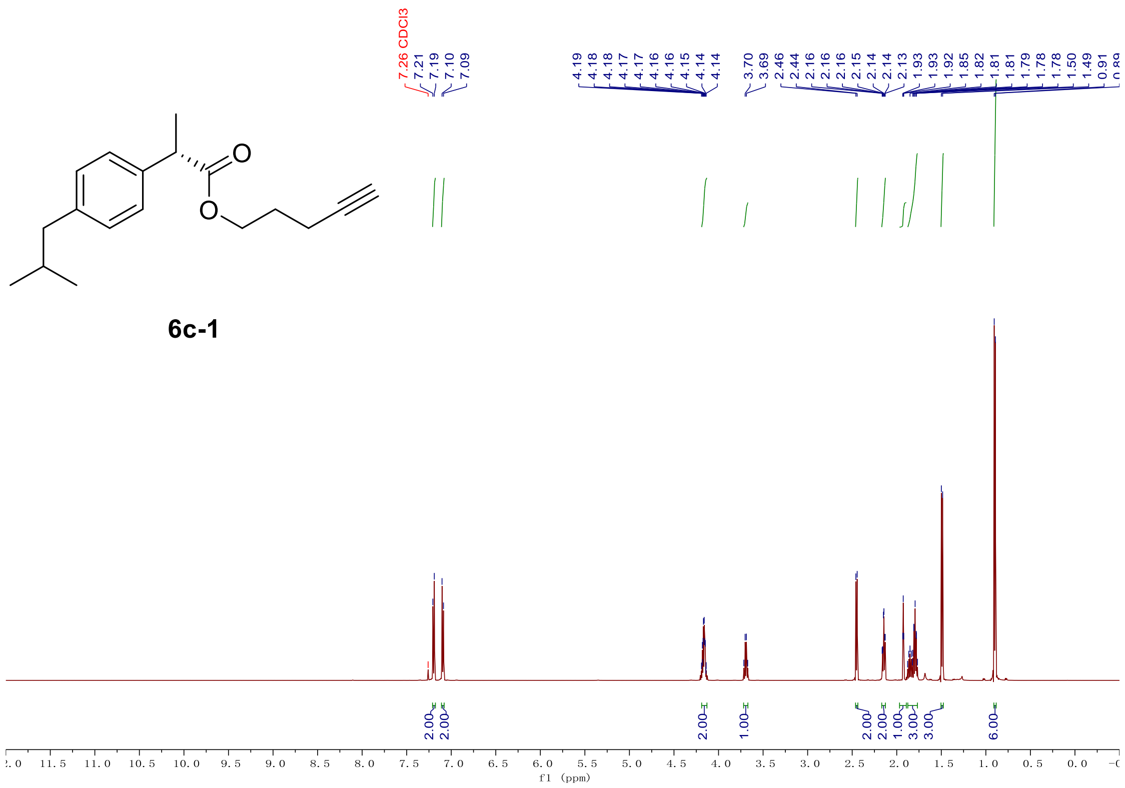

Supplementary Figure 44. ^{1}H NMR (500 MHz, $CDCl_3$) spectrum for compound **6c-1**

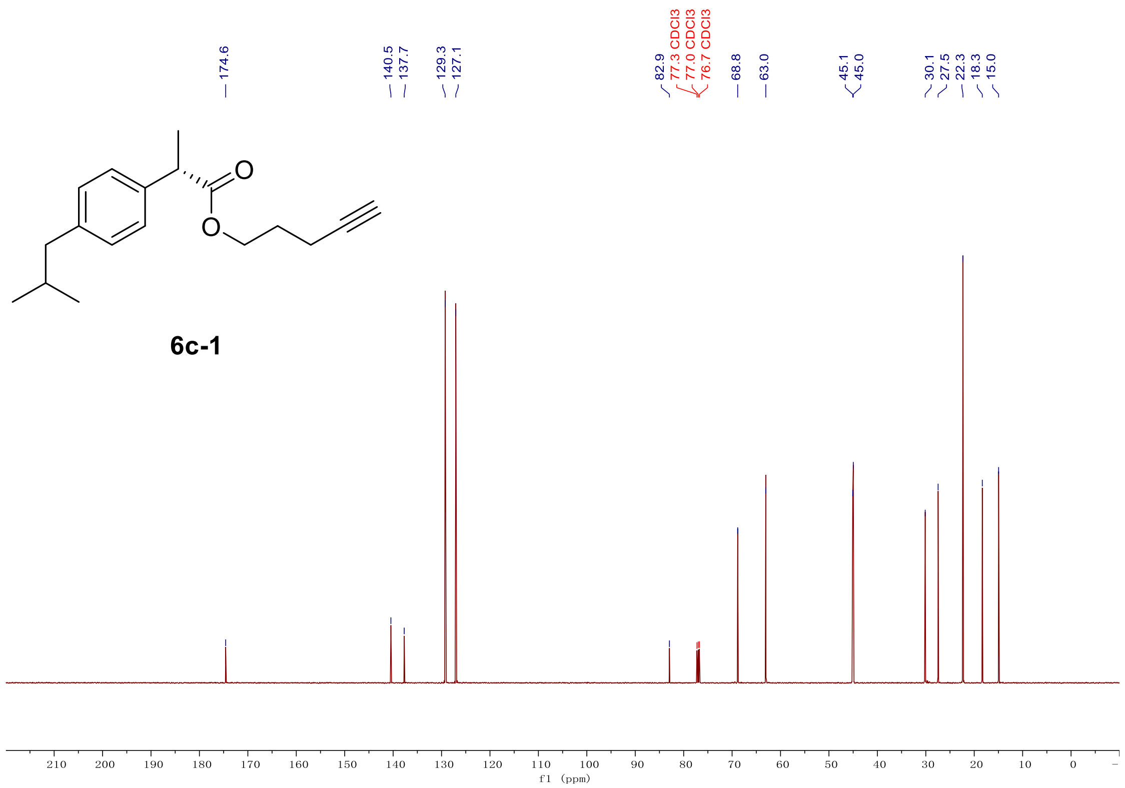

Supplementary Figure 45. ^{13}C NMR (125 MHz, $CDCl_3$) spectrum for compound **6c-1**

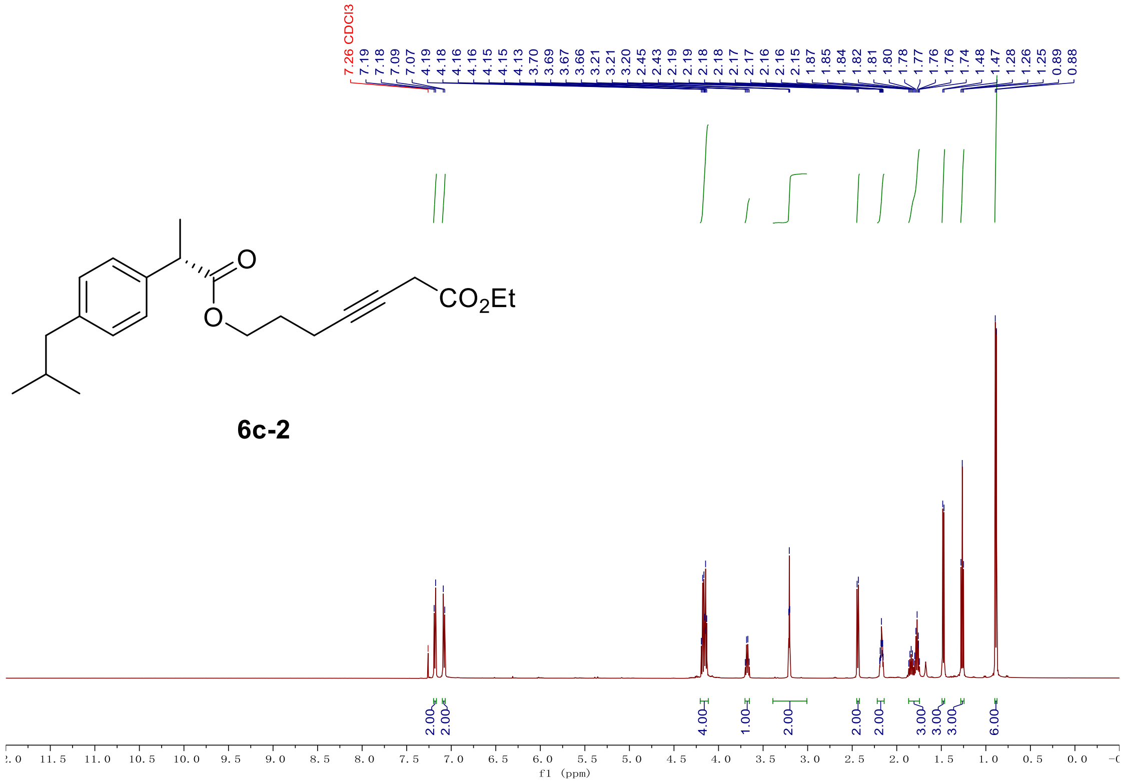

Supplementary Figure 46. ^{1}H NMR (500 MHz, $CDCl_3$) spectrum for compound **6c-2**

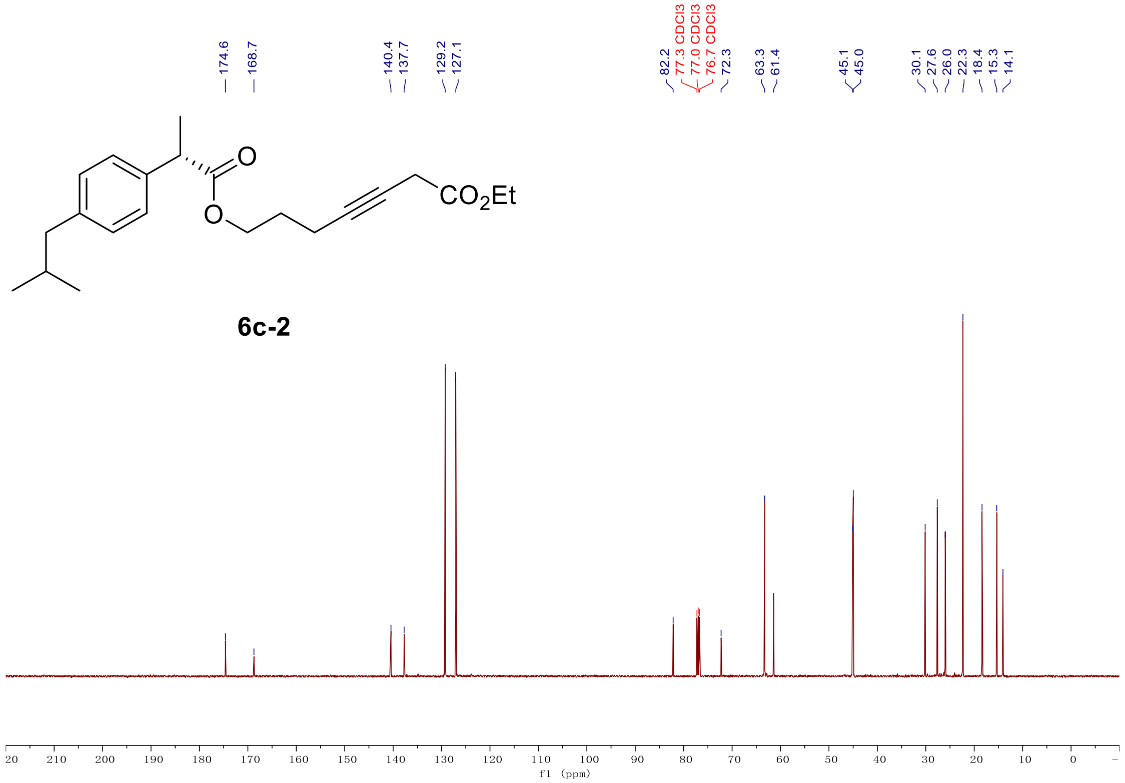

Supplementary Figure 47. ^{13}C NMR (125 MHz, $CDCl_3$) spectrum for compound **6c-2**

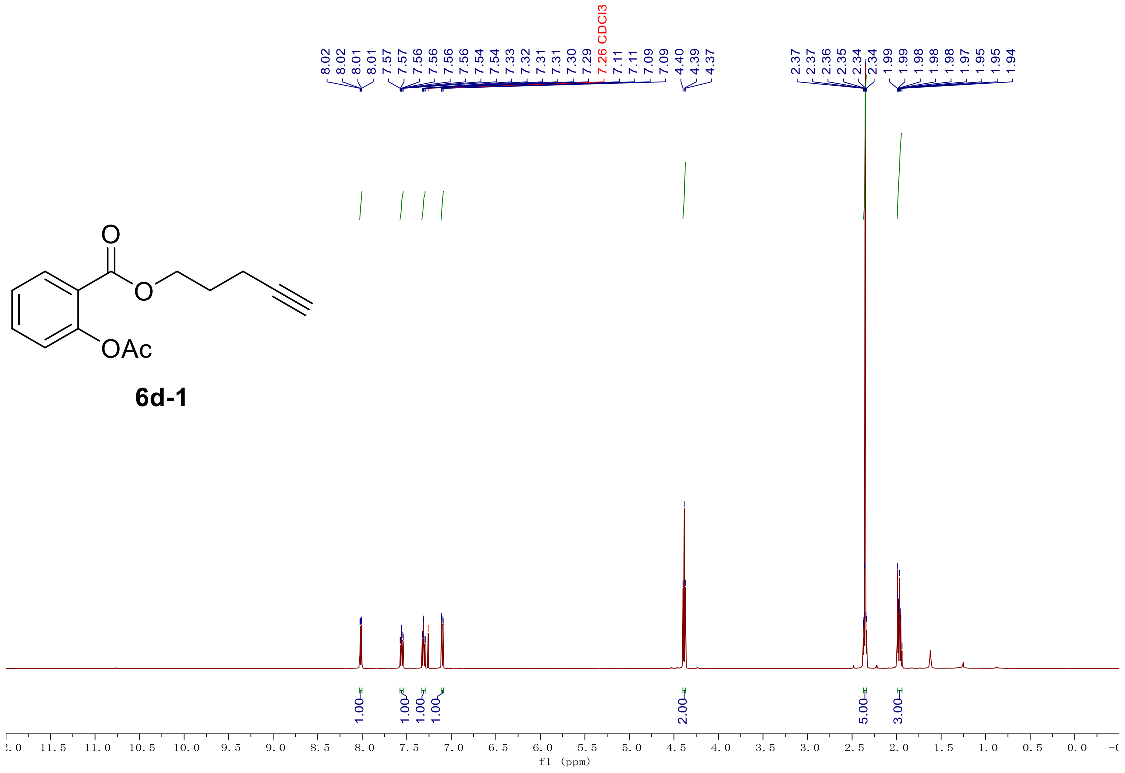

Supplementary Figure 48. ^{1}H NMR (500 MHz, $CDCl_3$) spectrum for compound **6d-1**

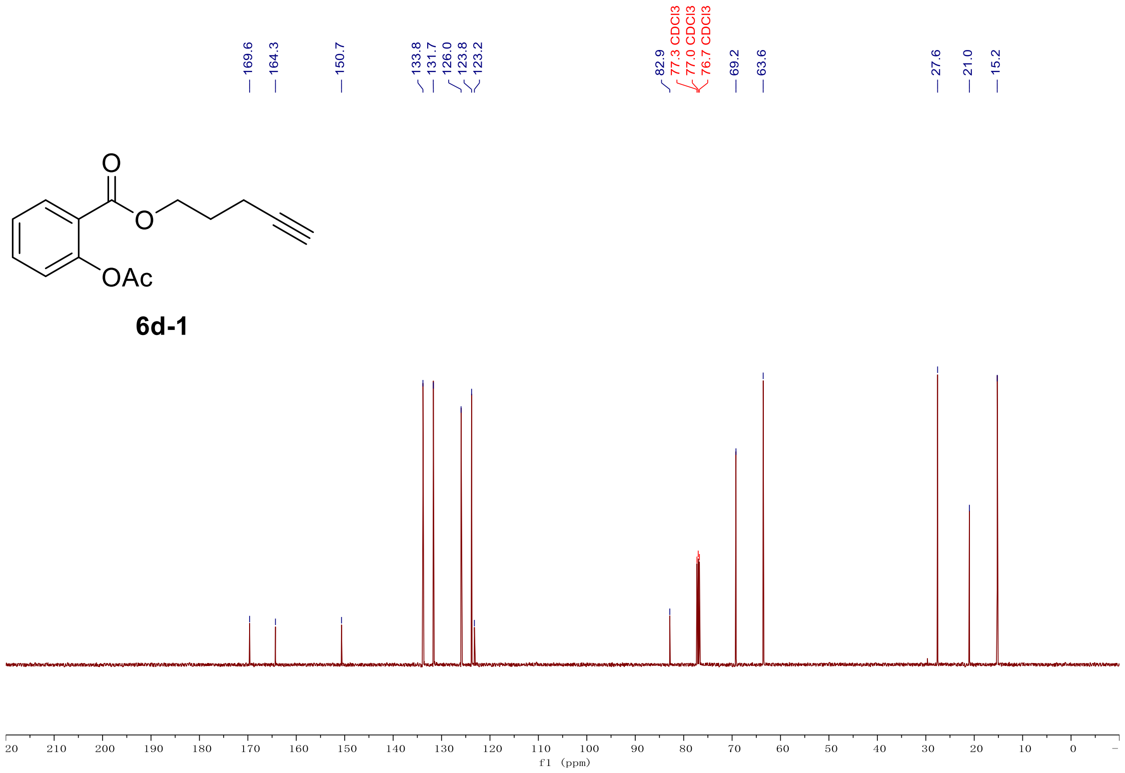

Supplementary Figure 49. ^{13}C NMR (125 MHz, $CDCl_3$) spectrum for compound **6d-1**

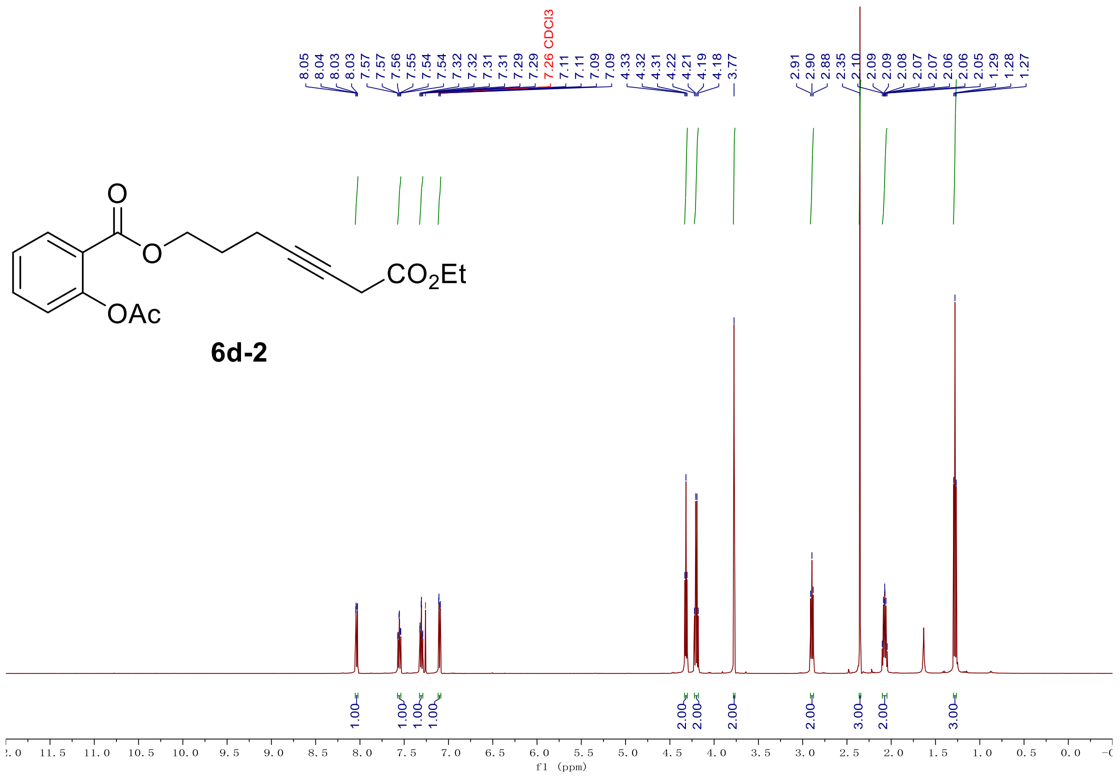

Supplementary Figure 50. ^{1}H NMR (500 MHz, $CDCl_3$) spectrum for compound **6d-2**

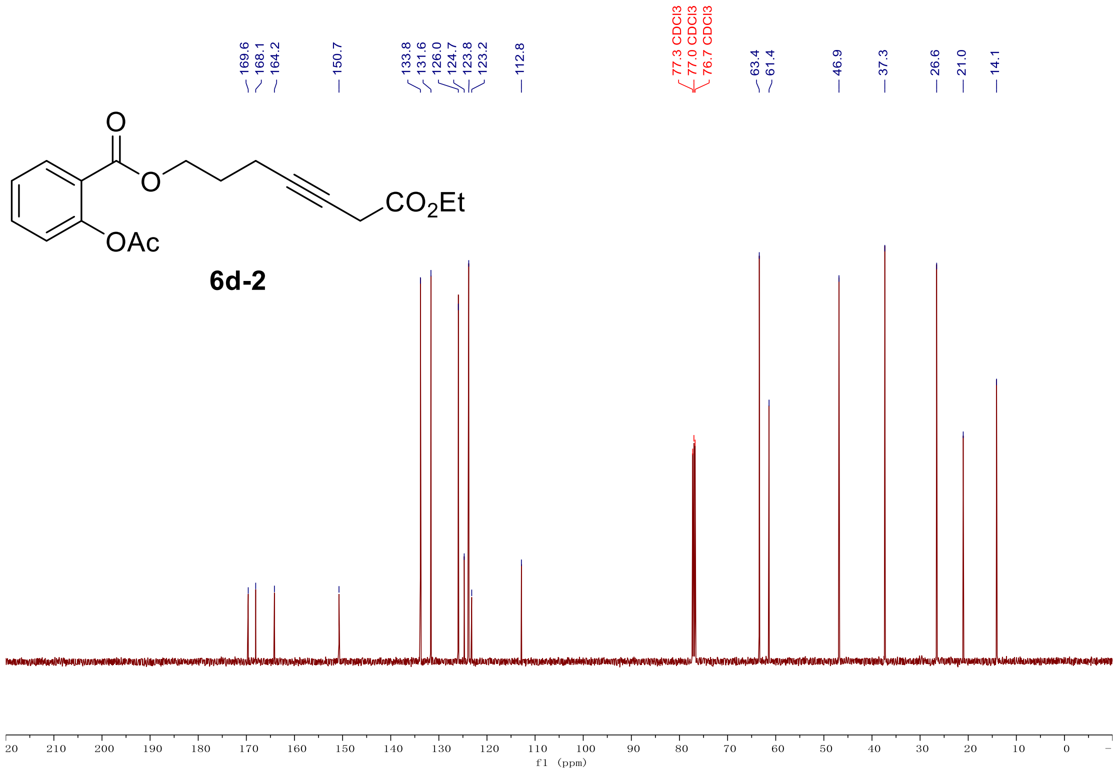

Supplementary Figure 51. ^{13}C NMR (125 MHz, $CDCl_3$) spectrum for compound **6d-2**

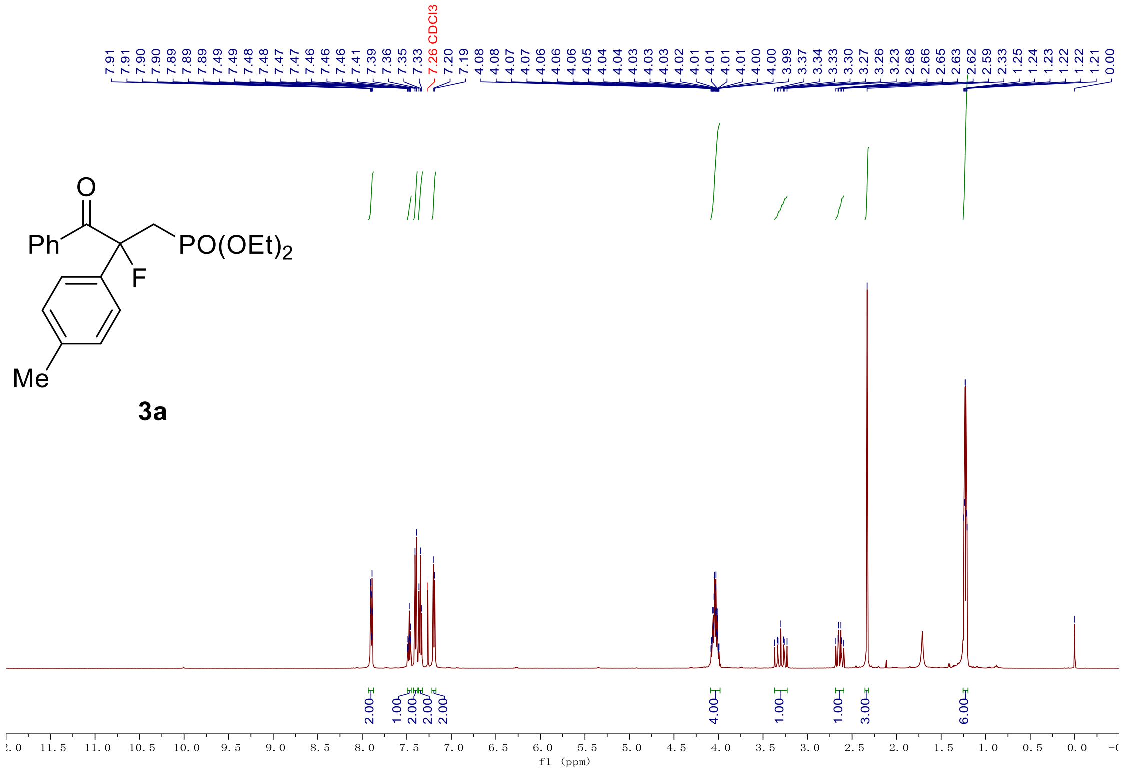

Supplementary Figure 52. ^{1}H NMR (500 MHz, $CDCl_3$) spectrum for compound **3a**

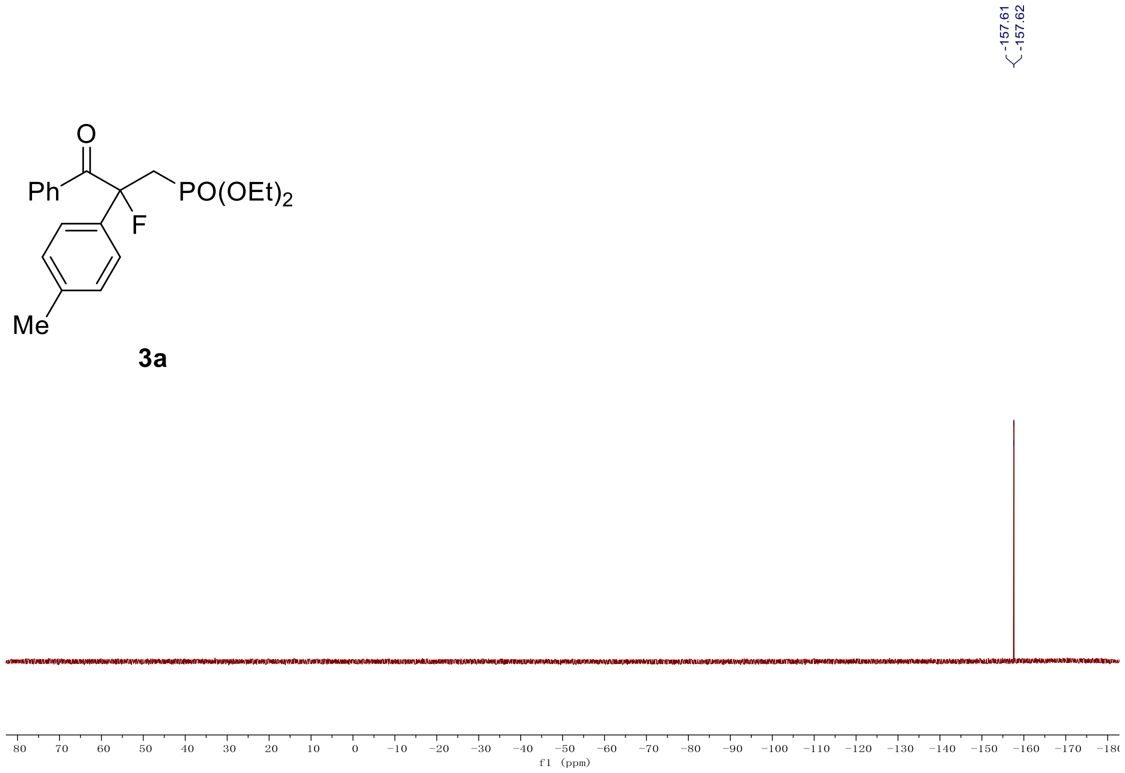

Supplementary Figure 53. ^{19}F NMR (471 MHz, $CDCl_3$) spectrum for compound **3a**

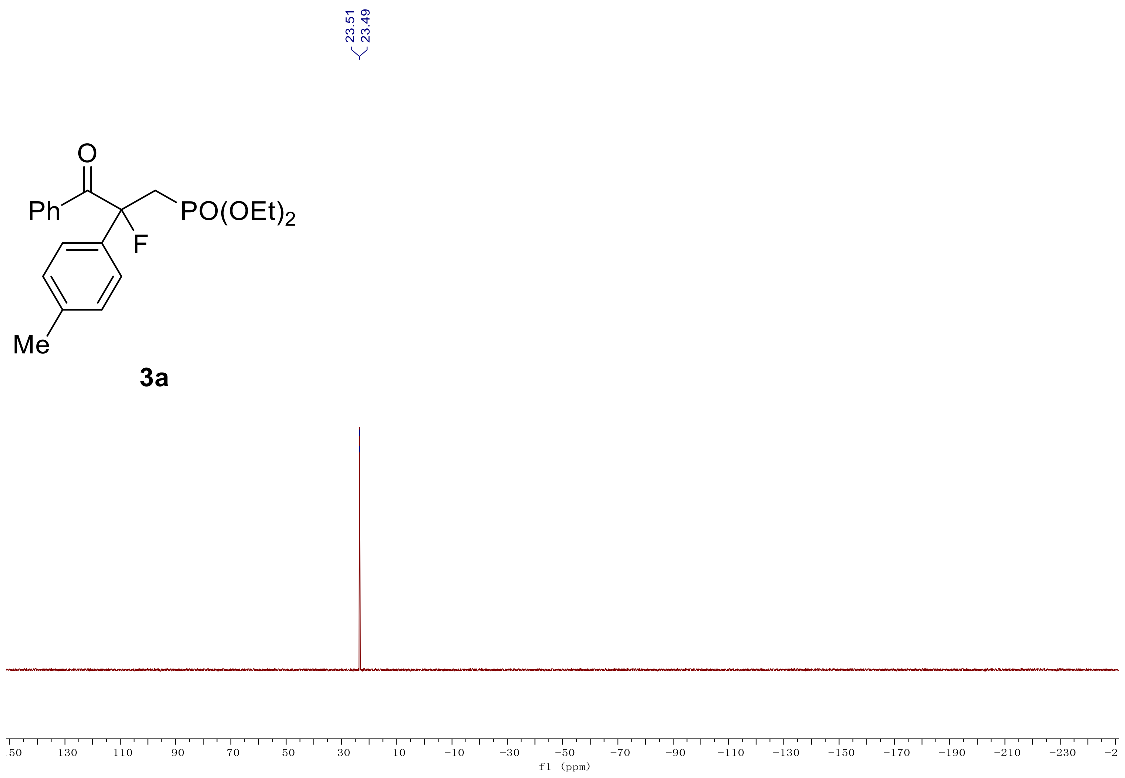

Supplementary Figure 54. ^{31}P NMR (202 MHz, CDCl3) spectrum for compound **3a**

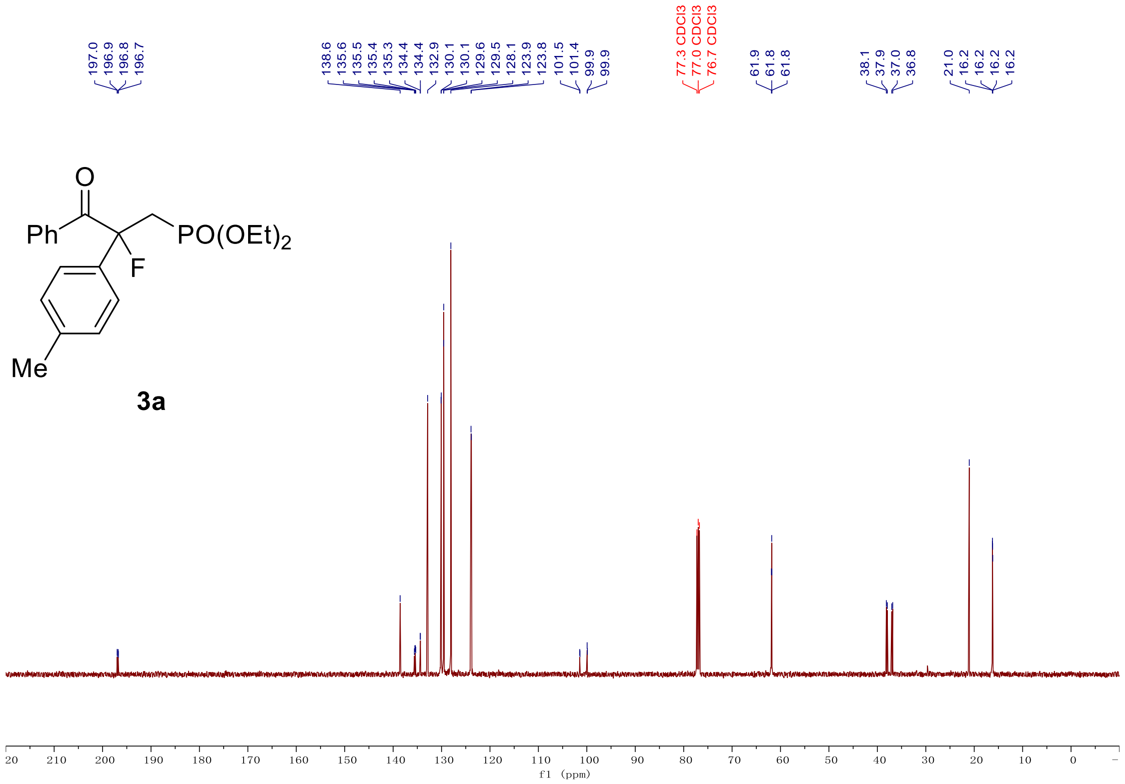

Supplementary Figure 55. ^{13}C NMR (125 MHz, CDCl3) spectrum for compound **3a**

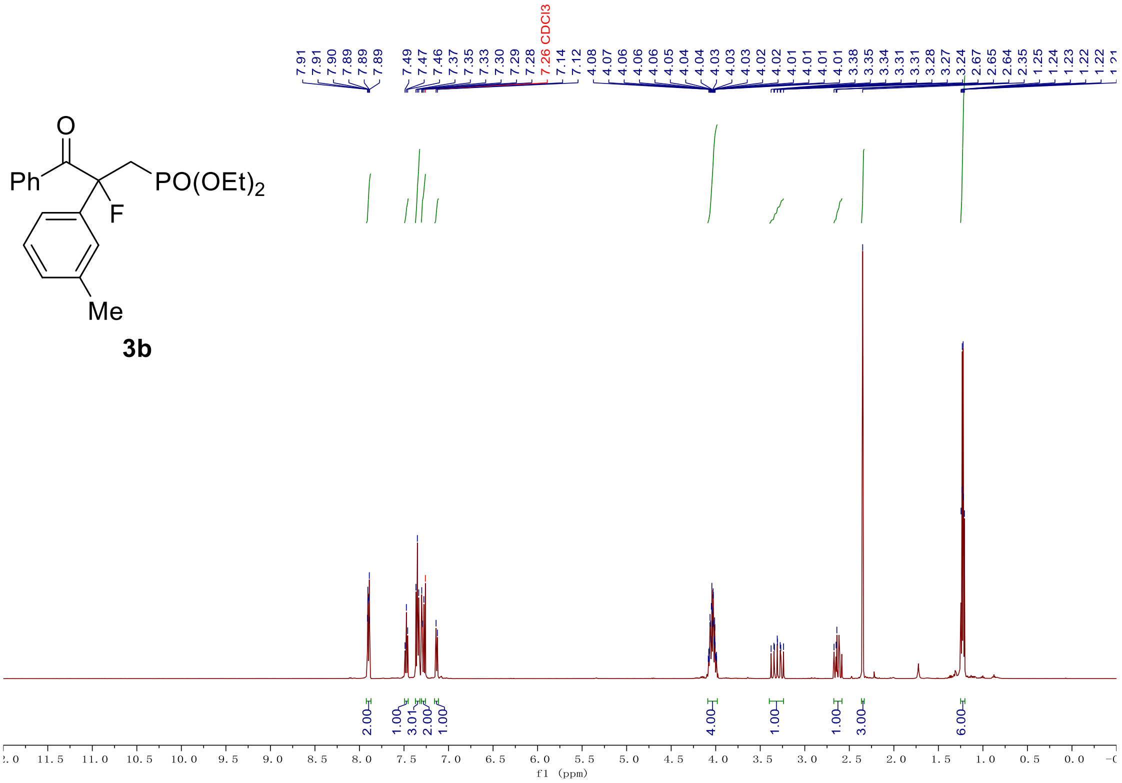

Supplementary Figure 56. ^{1}H NMR (500 MHz, $CDCl_3$) spectrum for compound **3b**

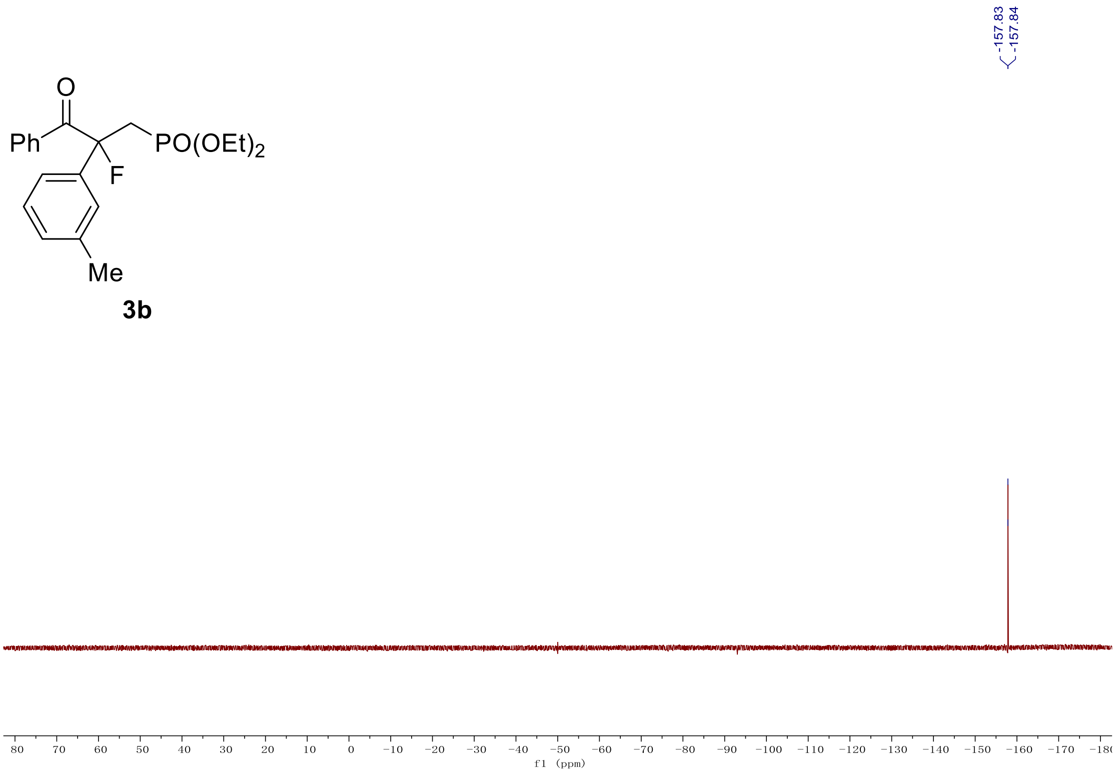

Supplementary Figure 57. ^{19}F NMR (471 MHz, $CDCl_3$) spectrum for compound **3b**

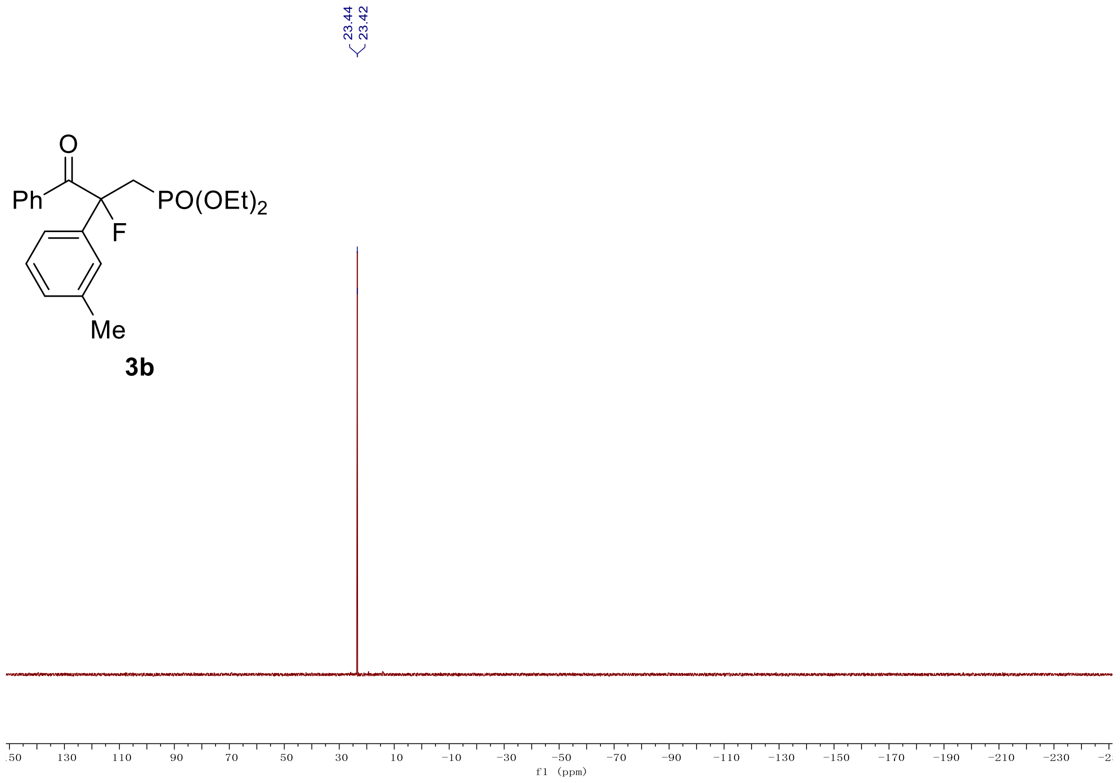

Supplementary Figure 58. ^{31}P NMR (202 MHz, CDCl$_3$) spectrum for compound **3b**

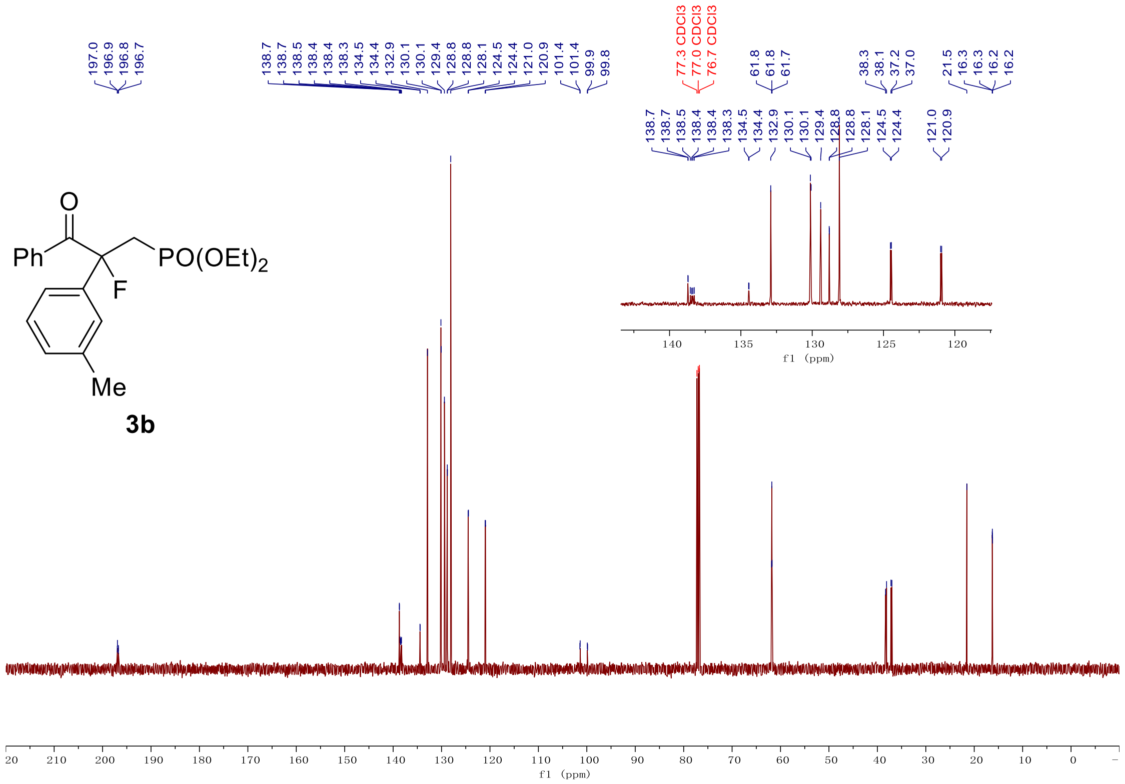

Supplementary Figure 59. ^{13}C NMR (125 MHz, CDCl$_3$) spectrum for compound **3b**

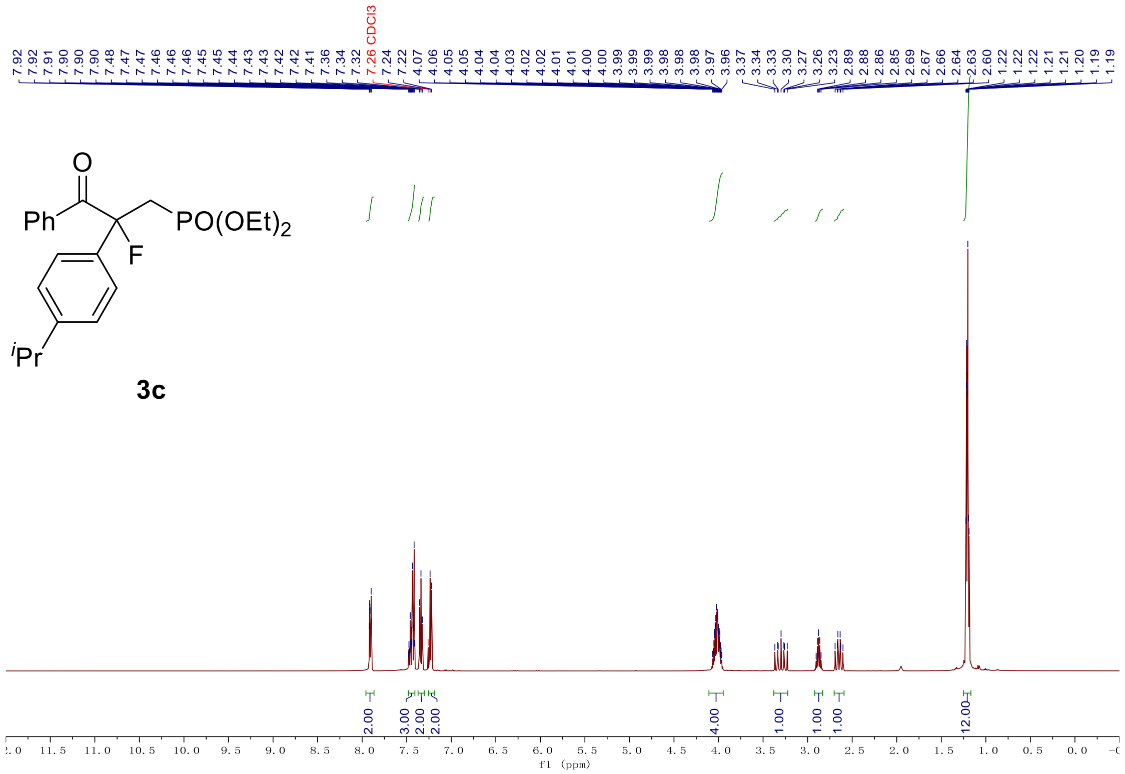

Supplementary Figure 60. ^{1}H NMR (500 MHz, $CDCl_3$) spectrum for compound **3c**

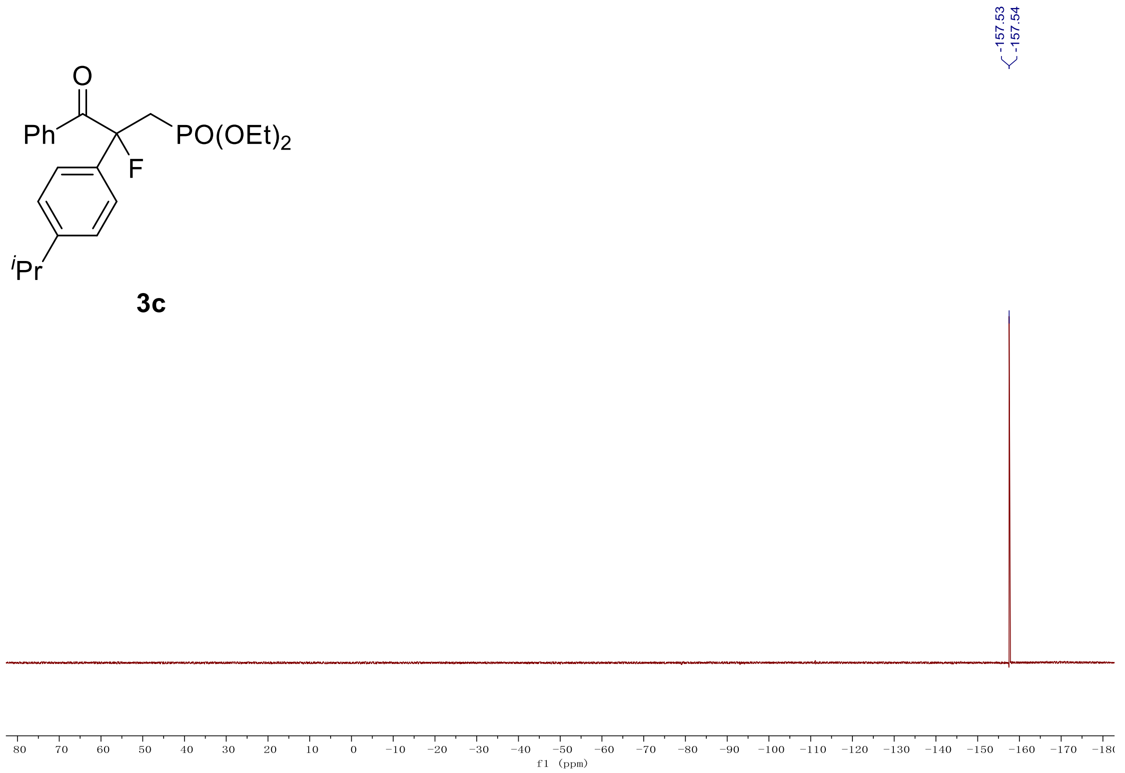

Supplementary Figure 61. ^{19}F NMR (471 MHz, $CDCl_3$) spectrum for compound **3c**

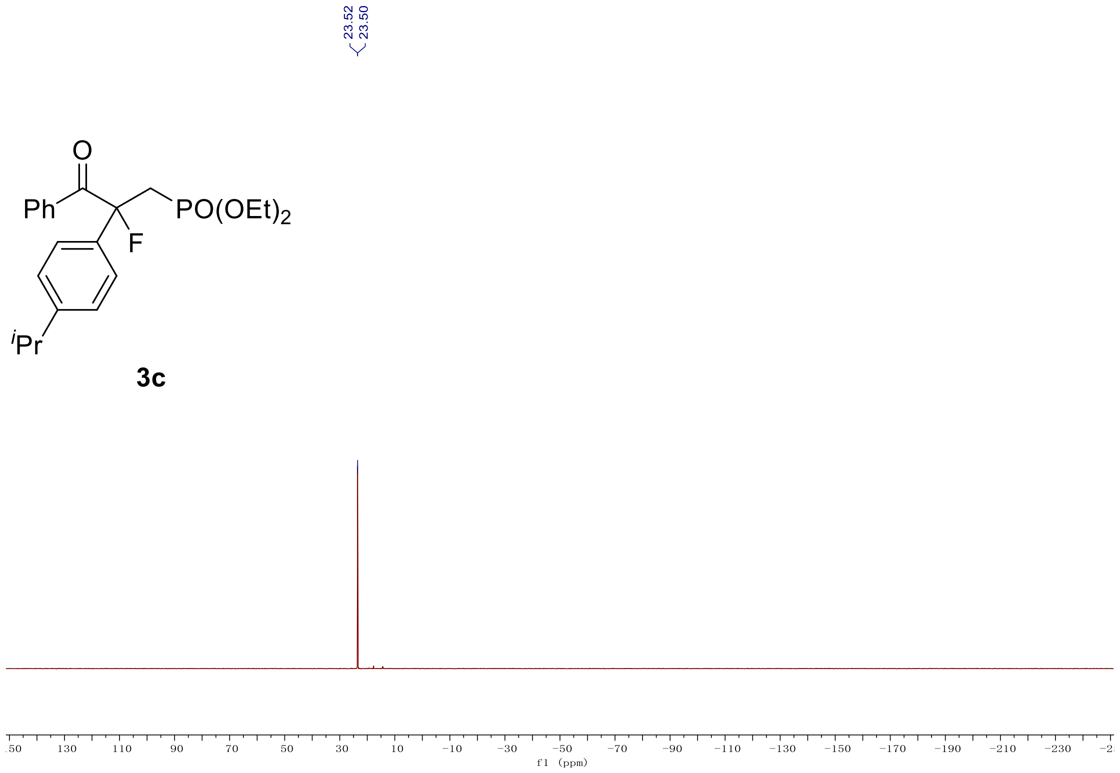

Supplementary Figure 62. ^{31}P NMR (202 MHz, $CDCl_3$) spectrum for compound **3c**

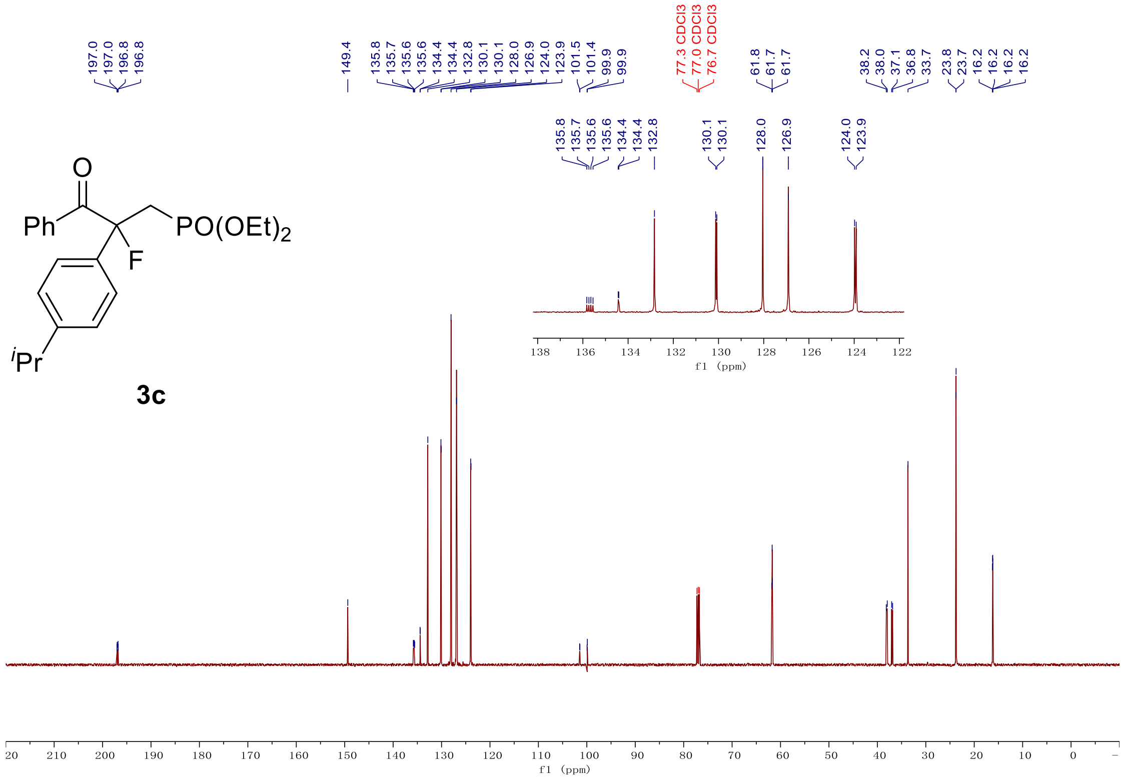

Supplementary Figure 63. ^{13}C NMR (125 MHz, $CDCl_3$) spectrum for compound **3c**

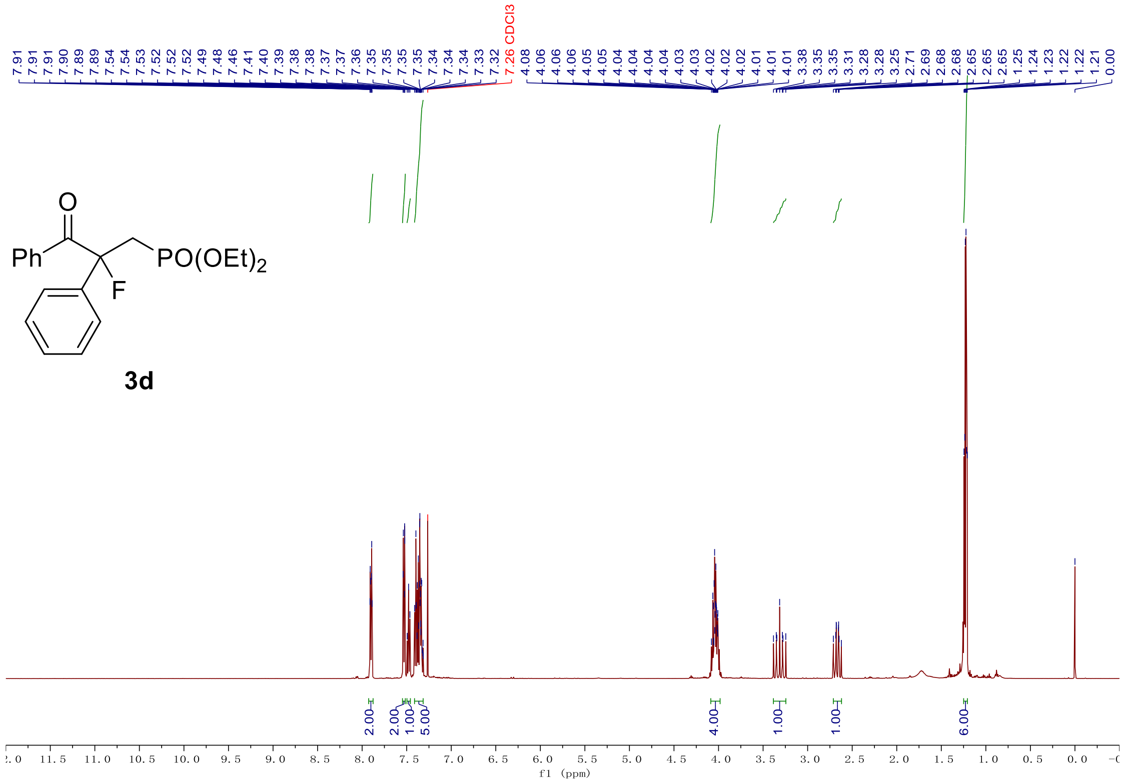

Supplementary Figure 64. ^{1}H NMR (500 MHz, $CDCl_3$) spectrum for compound **3d**

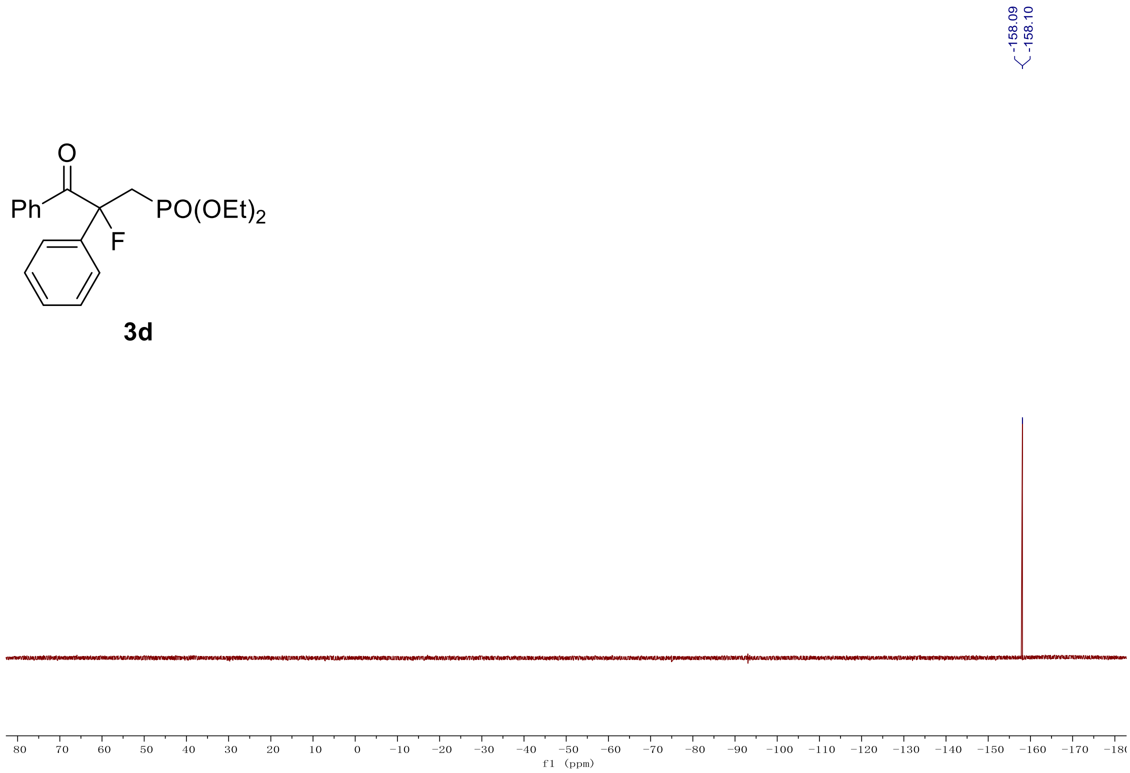

Supplementary Figure 65. ^{19}F NMR (471 MHz, $CDCl_3$) spectrum for compound **3d**

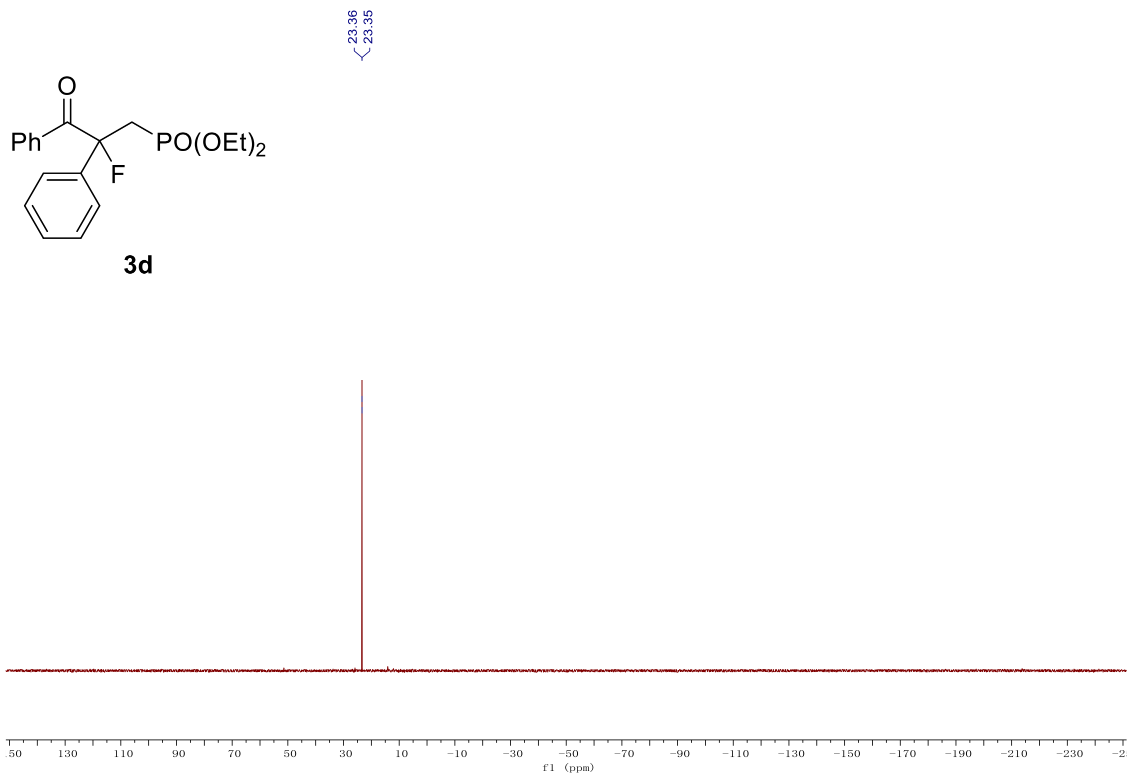

Supplementary Figure 66. ^{31}P NMR (202 MHz, $CDCl_3$) spectrum for compound **3d**

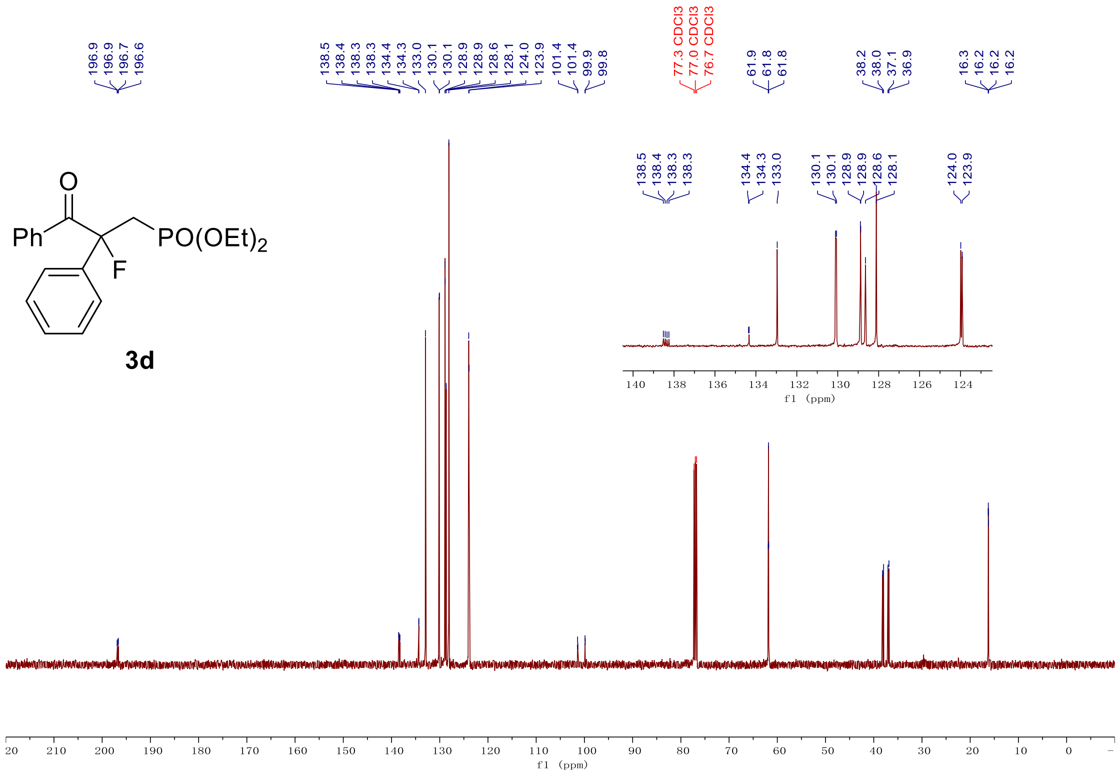

Supplementary Figure 67. ^{13}C NMR (125 MHz, $CDCl_3$) spectrum for compound **3d**

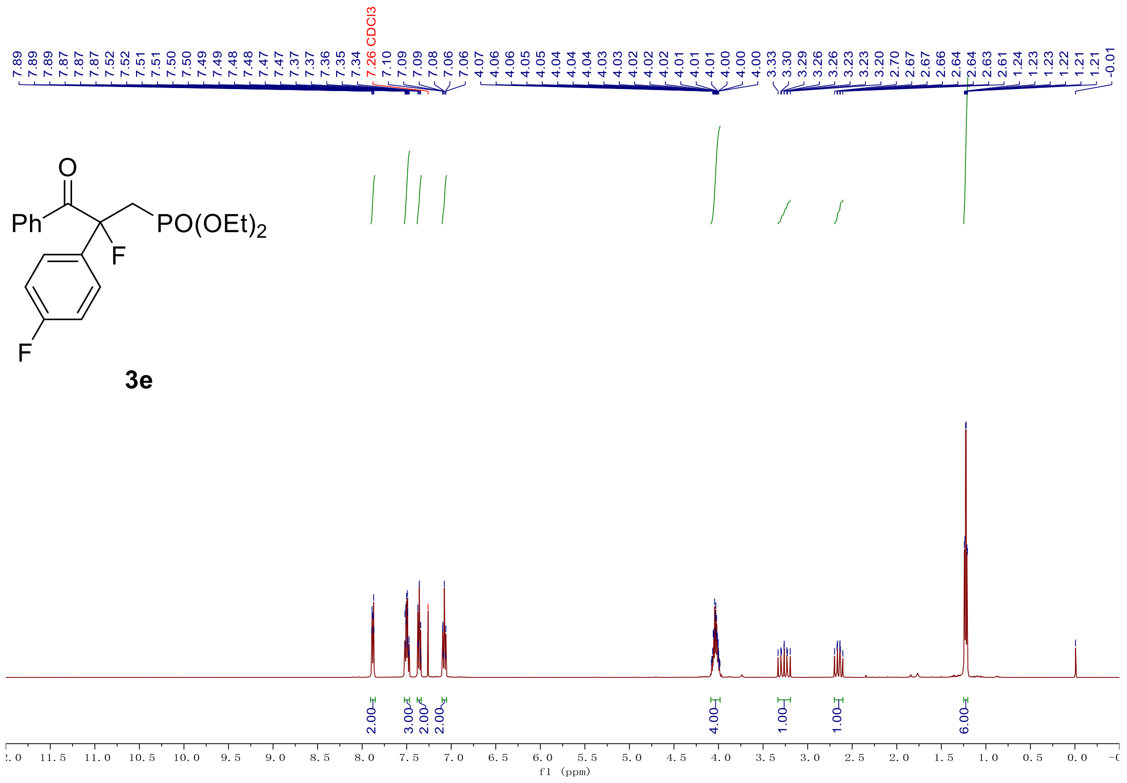

Supplementary Figure 68. ^{1}H NMR (500 MHz, $CDCl_3$) spectrum for compound **3e**

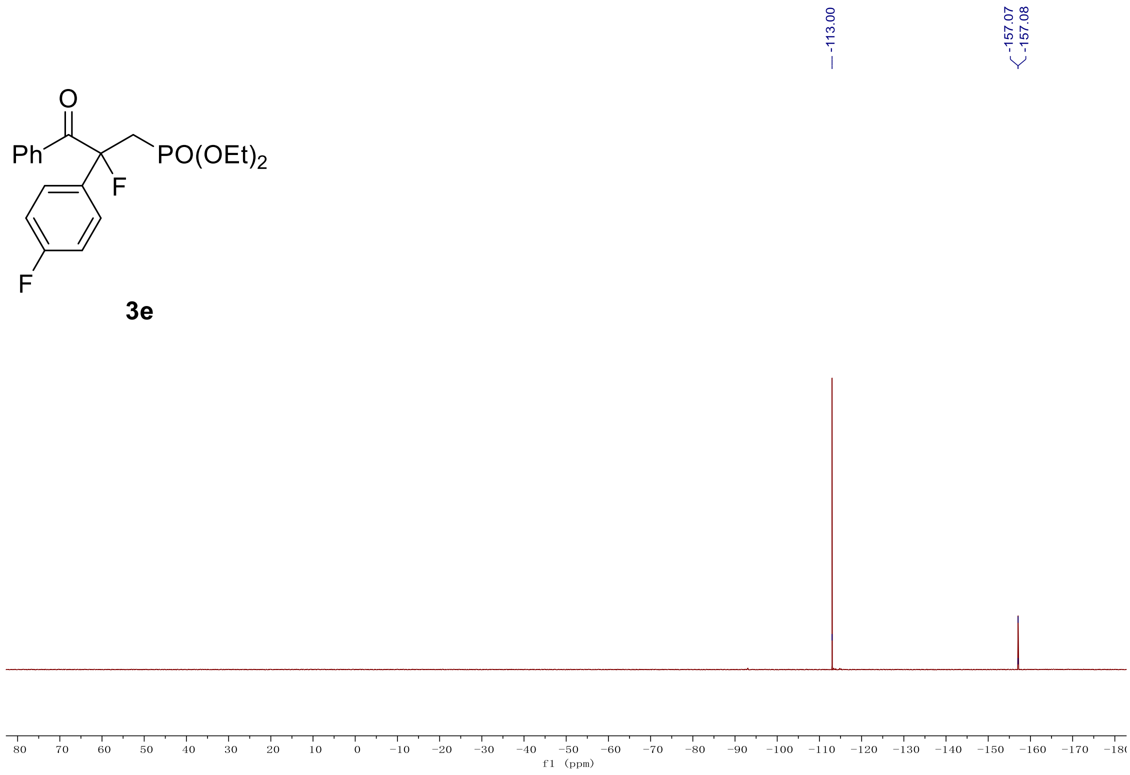

Supplementary Figure 69. ^{19}F NMR (471 MHz, $CDCl_3$) spectrum for compound **3e**

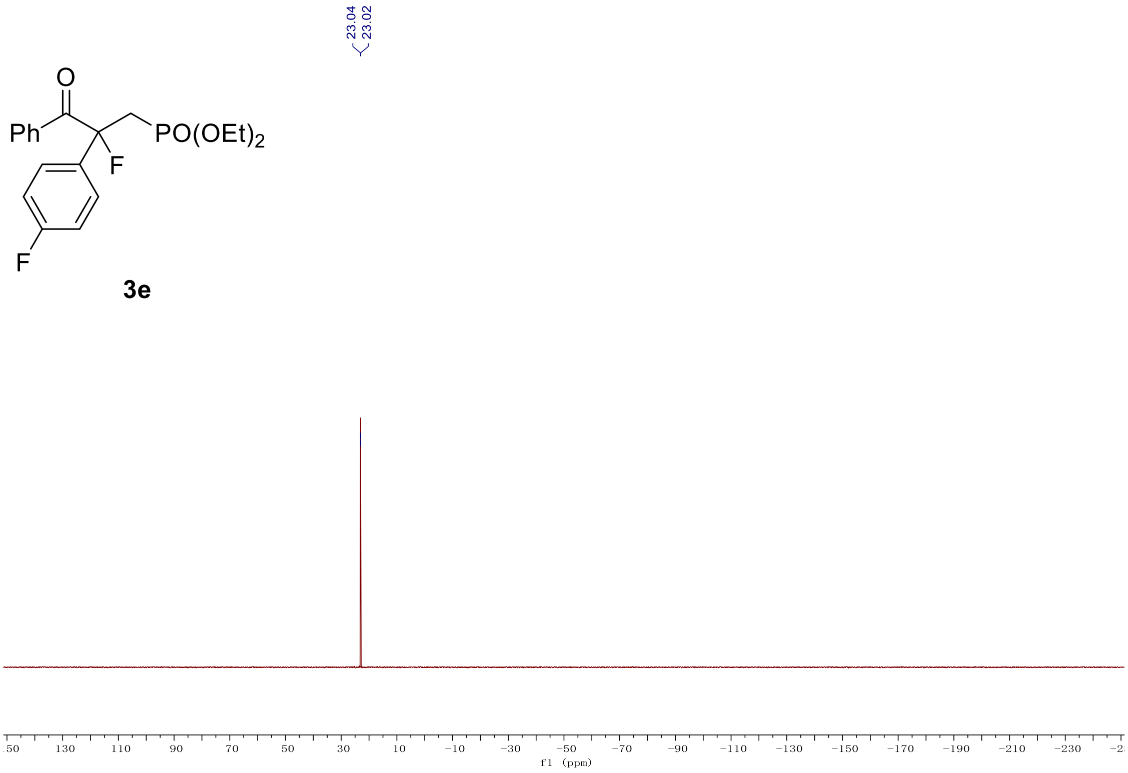

Supplementary Figure 70. ^{31}P NMR (202 MHz, CDCl$_3$) spectrum for compound **3e**

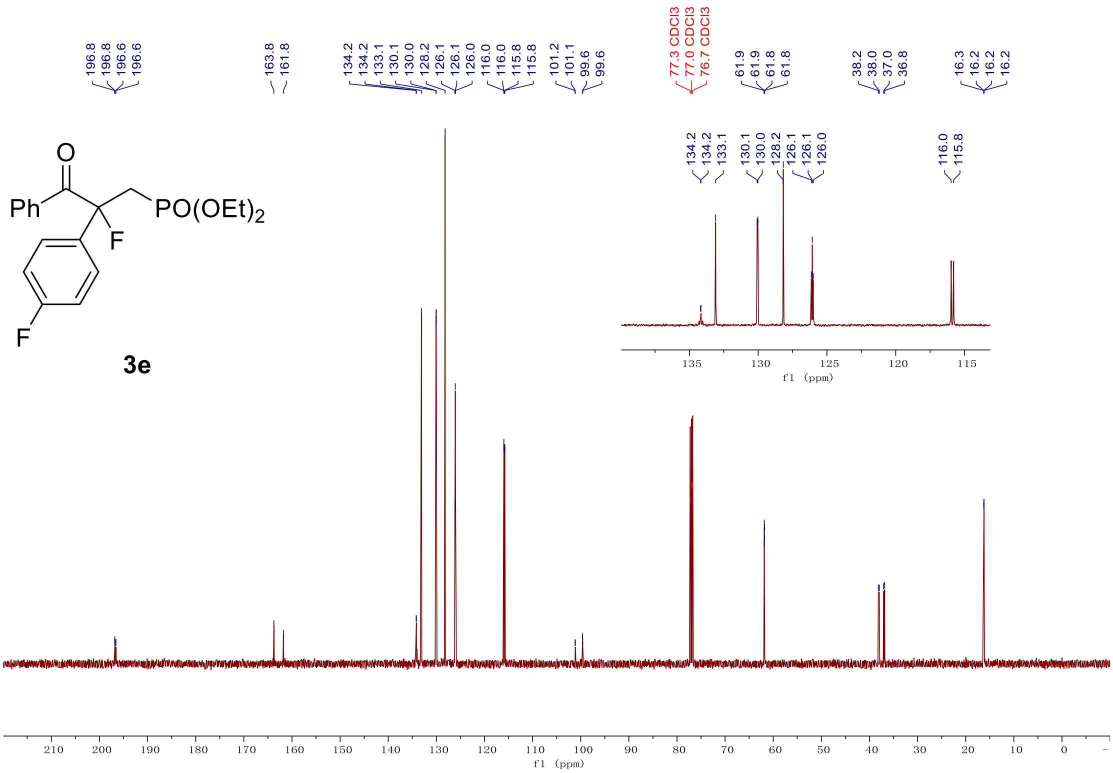

Supplementary Figure 71. ^{13}C NMR (125 MHz, CDCl$_3$) spectrum for compound **3e**

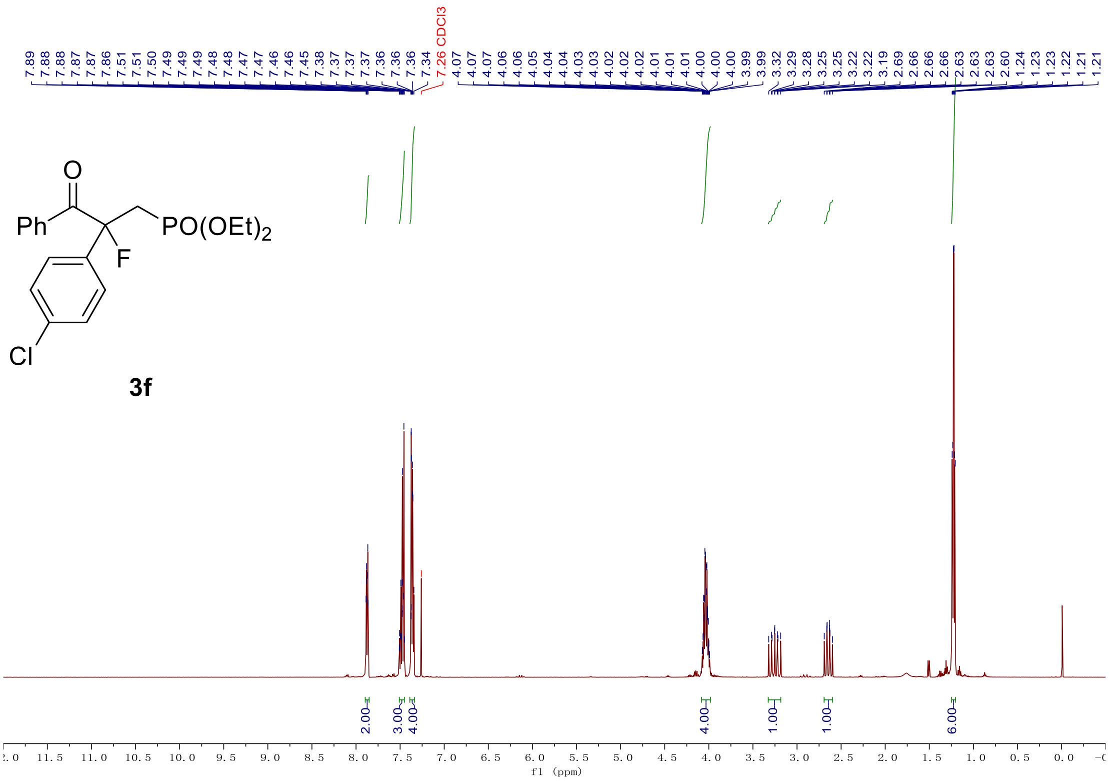

Supplementary Figure 72. ^{1}H NMR (500 MHz, $CDCl_3$) spectrum for compound **3f**

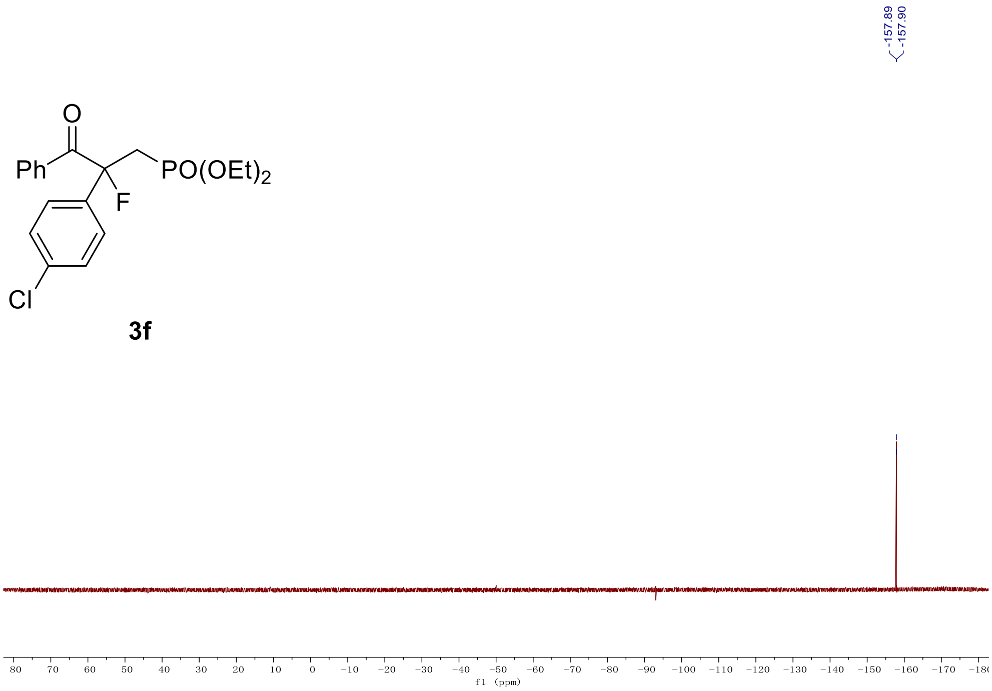

Supplementary Figure 73. ^{19}F NMR (471 MHz, $CDCl_3$) spectrum for compound **3f**

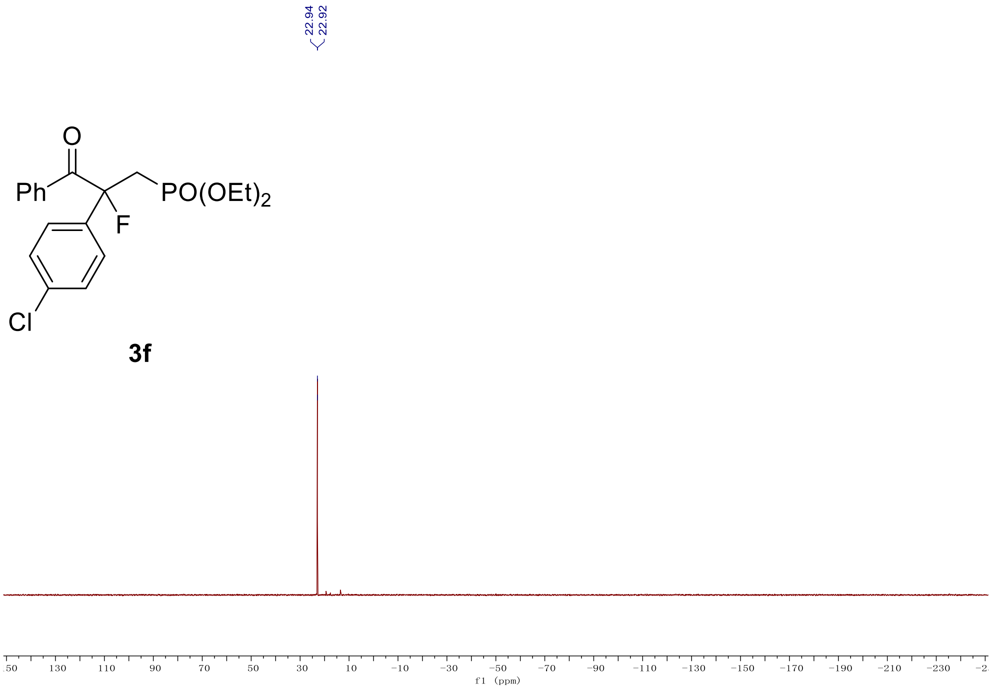

Supplementary Figure 74. ^{31}P NMR (202 MHz, $CDCl_3$) spectrum for compound **3f**

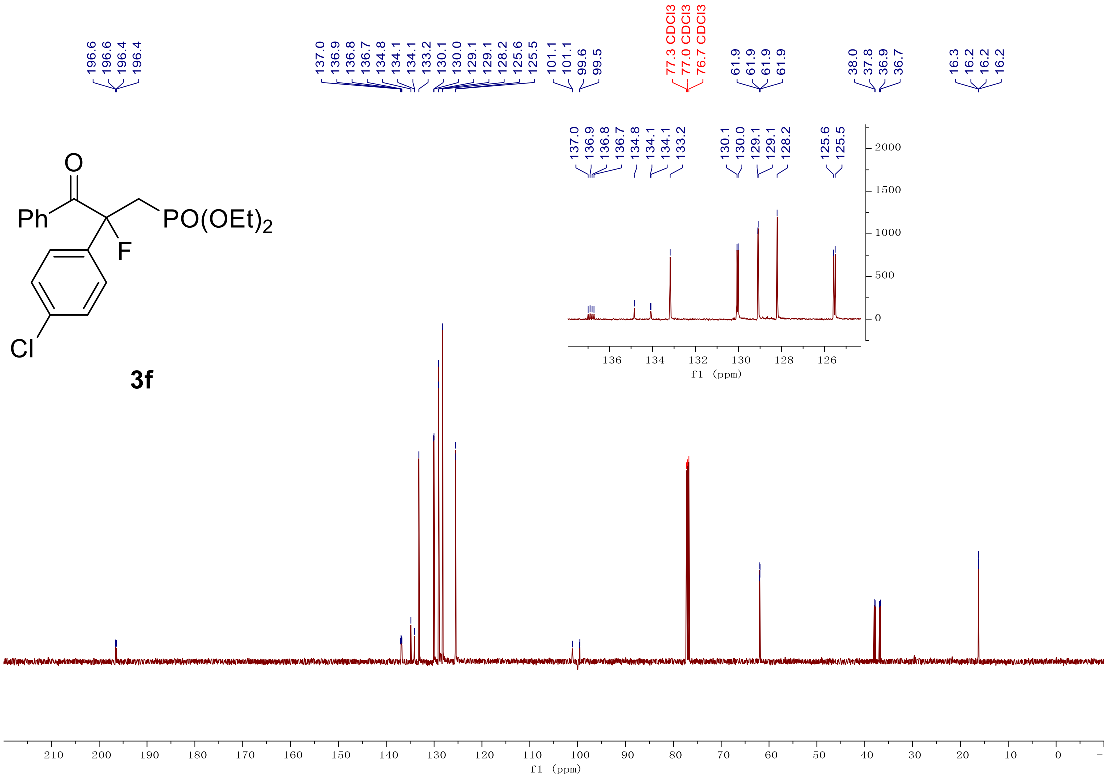

Supplementary Figure 75. ^{13}C NMR (125 MHz, $CDCl_3$) spectrum for compound **3f**

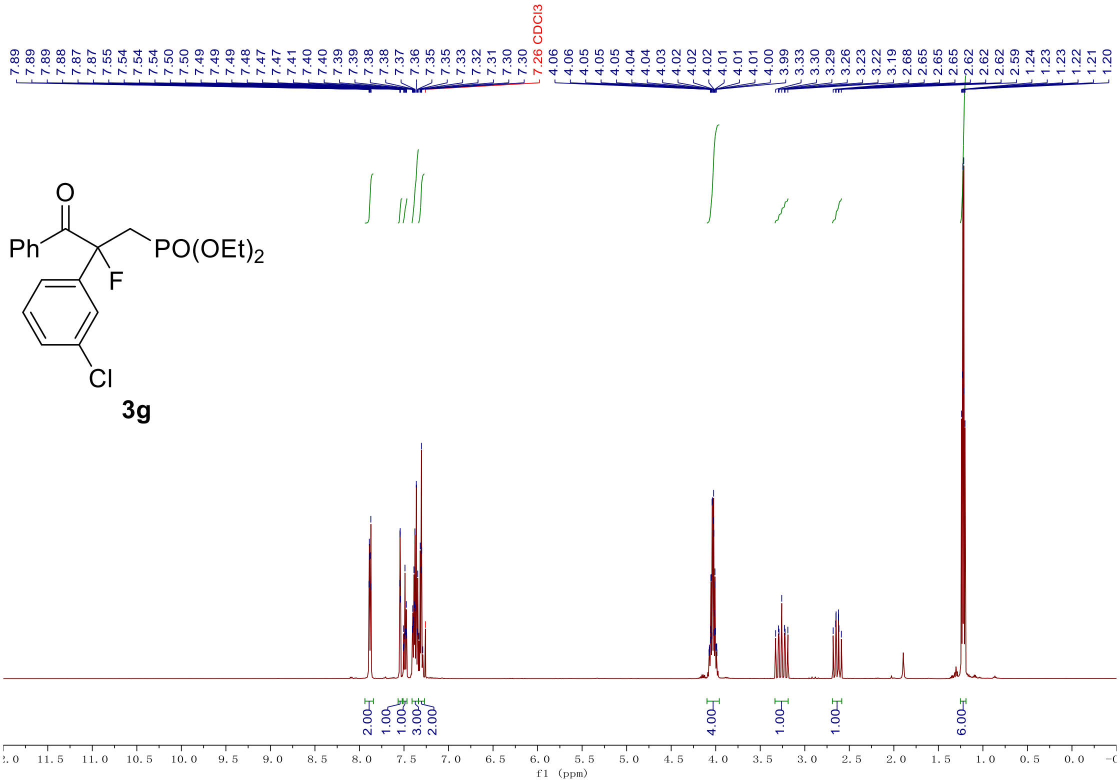

Supplementary Figure 76. ^{1}H NMR (500 MHz, $CDCl_3$) spectrum for compound **3g**

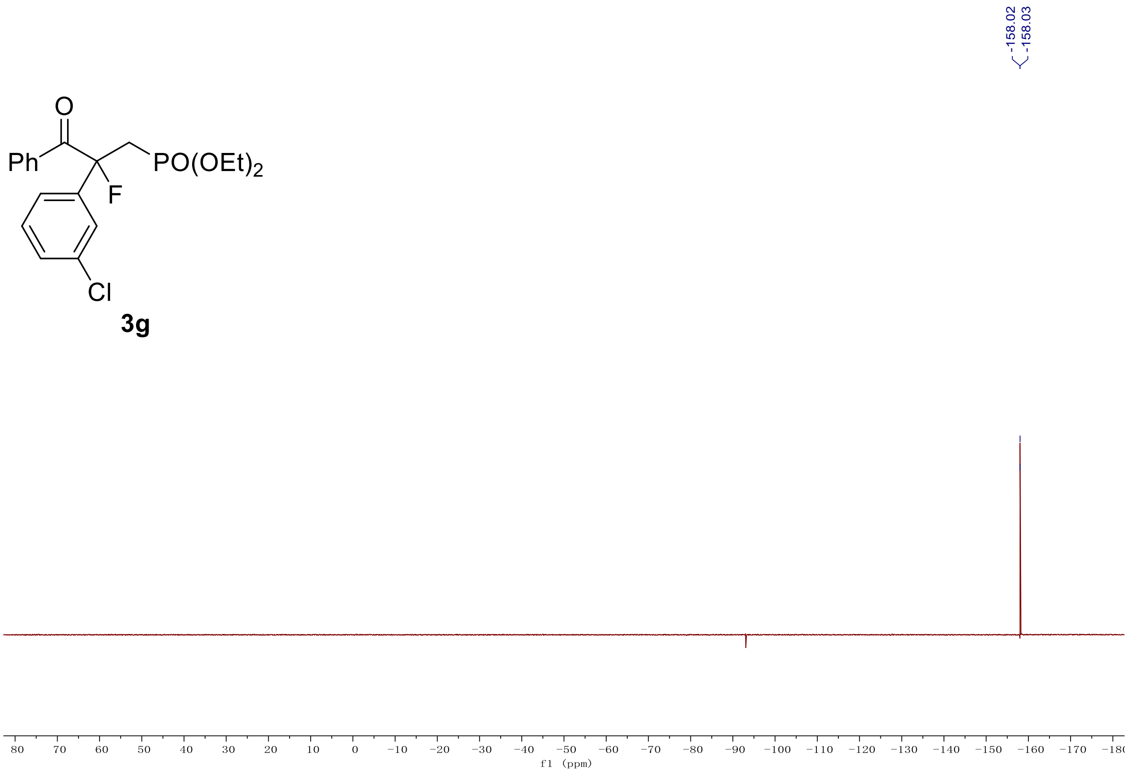

Supplementary Figure 77. ^{19}F NMR (471 MHz, $CDCl_3$) spectrum for compound **3g**

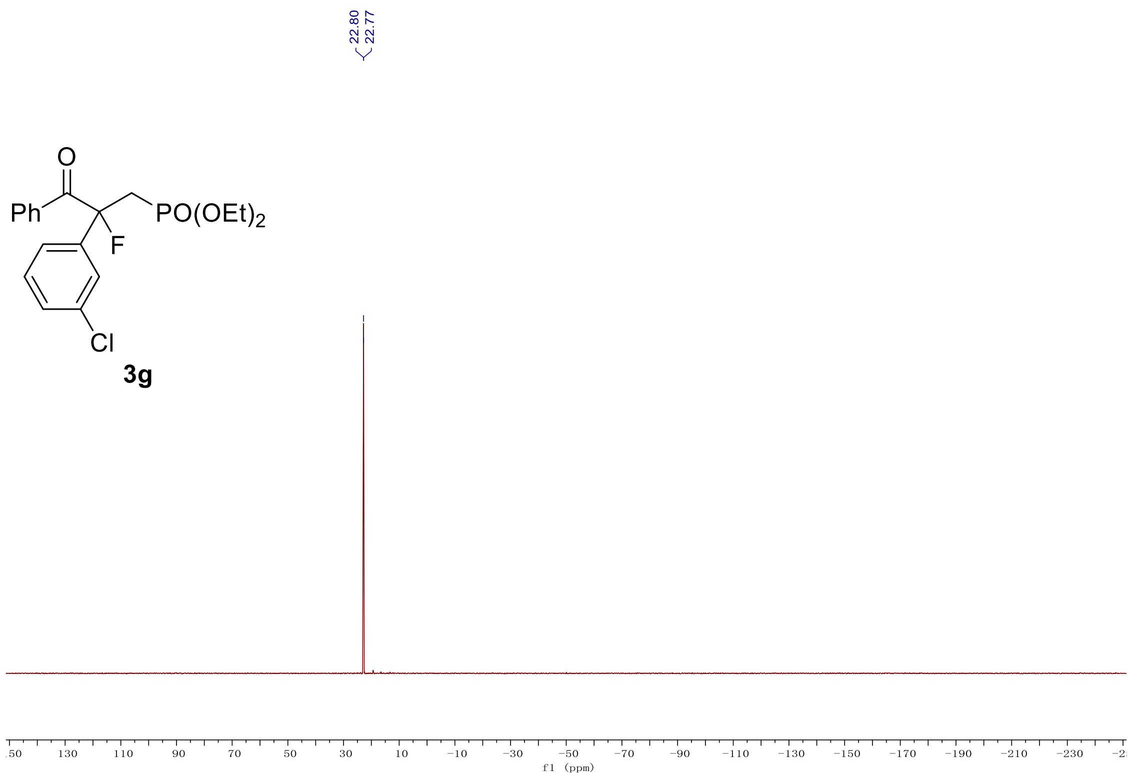

Supplementary Figure 78. ^{31}P NMR (202 MHz, $CDCl_3$) spectrum for compound **3g**

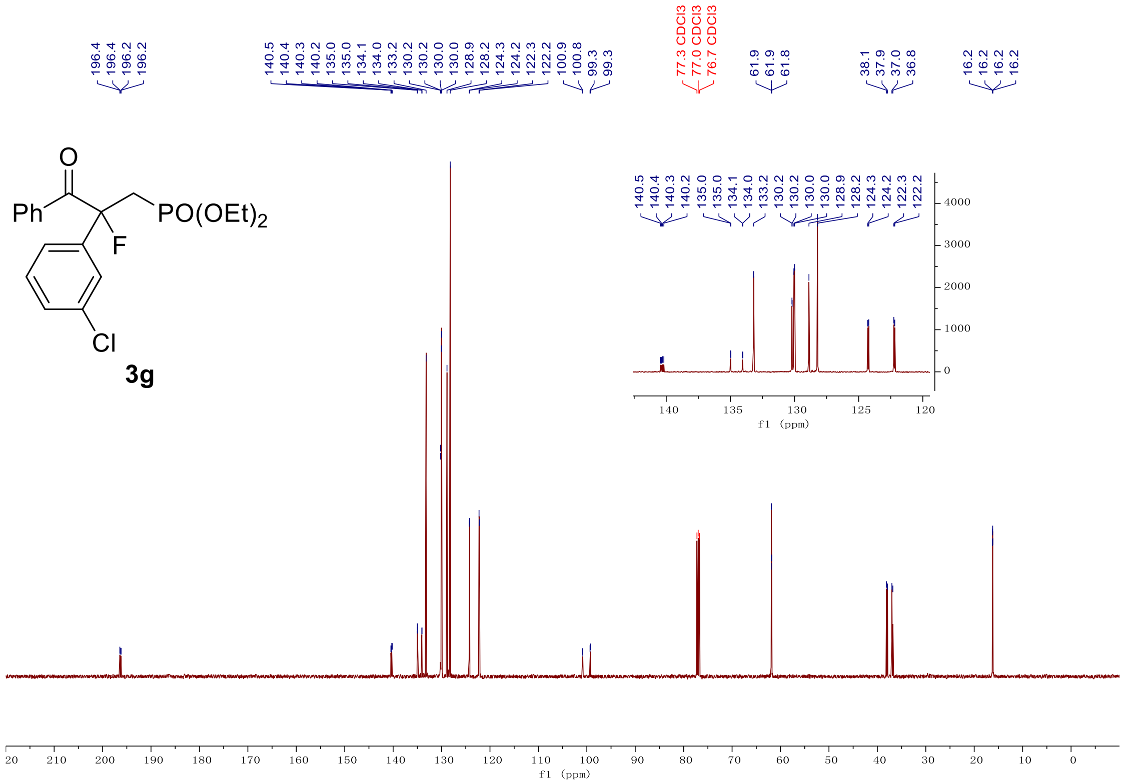

Supplementary Figure 79. ^{13}C NMR (125 MHz, $CDCl_3$) spectrum for compound **3g**

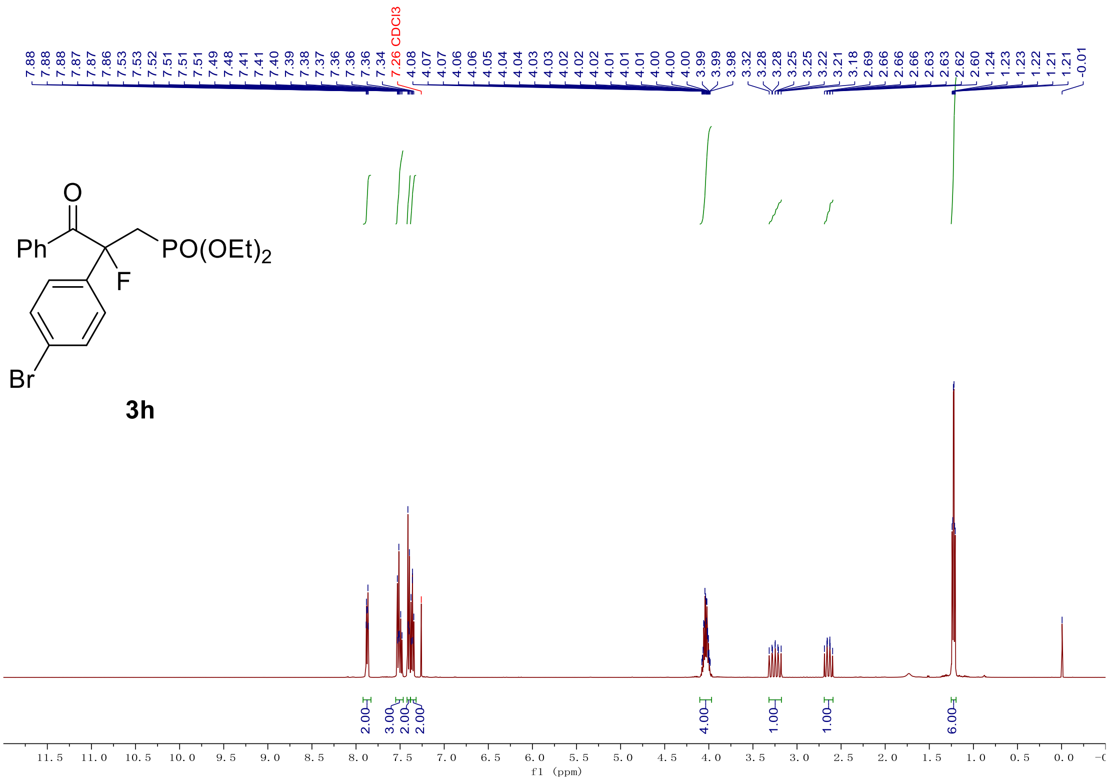

Supplementary Figure 80. ^{1}H NMR (500 MHz, $CDCl_3$) spectrum for compound **3h**

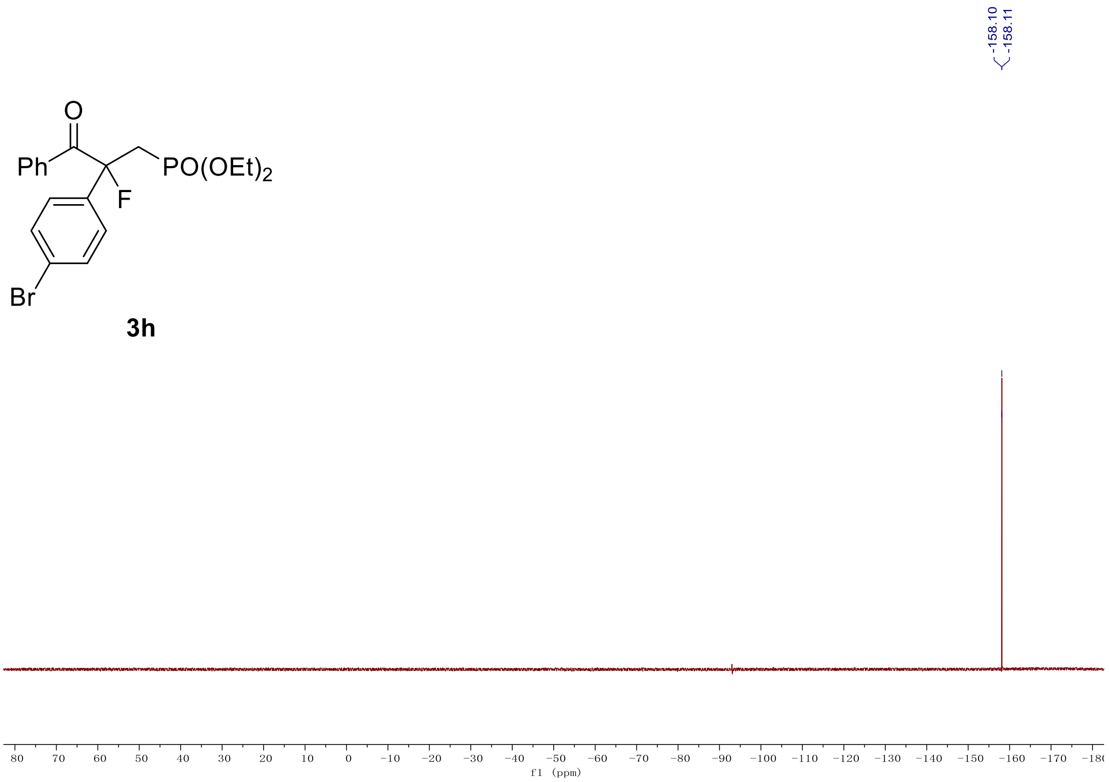

Supplementary Figure 81. ^{19}F NMR (471 MHz, $CDCl_3$) spectrum for compound **3h**

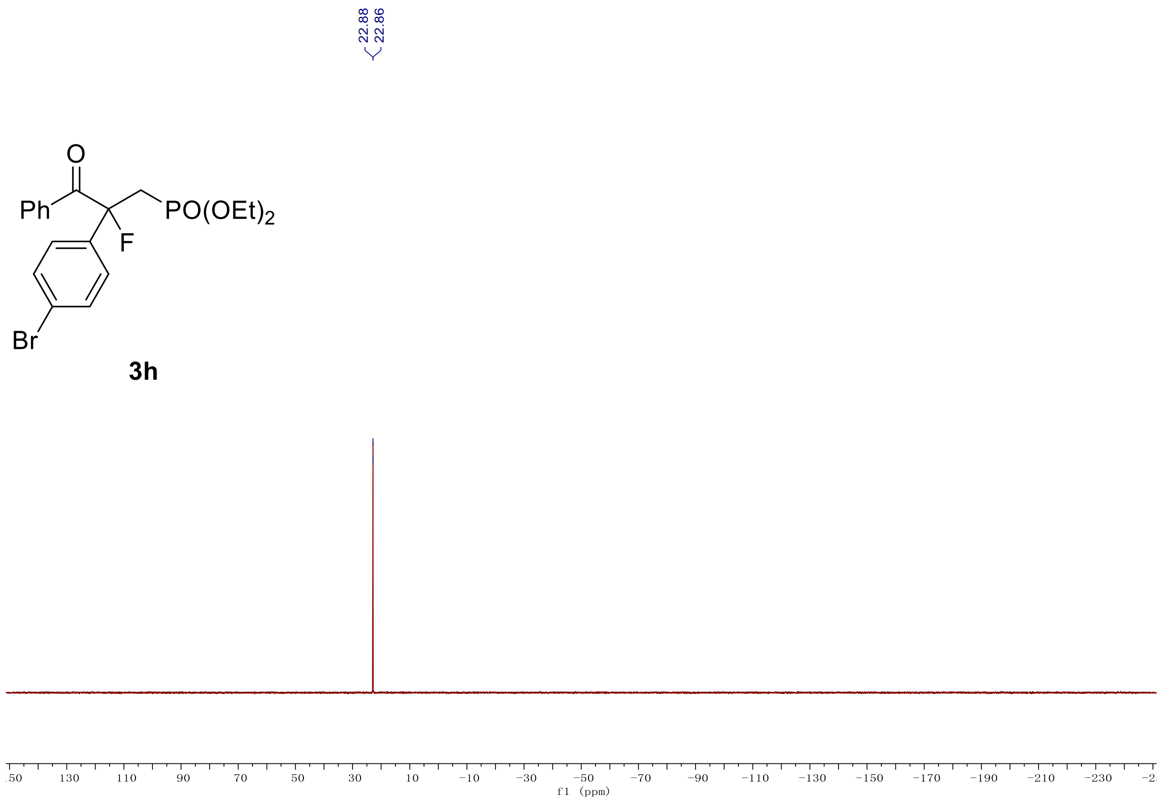

Supplementary Figure 82. ^{31}P NMR (202 MHz, CDCl3) spectrum for compound **3h**

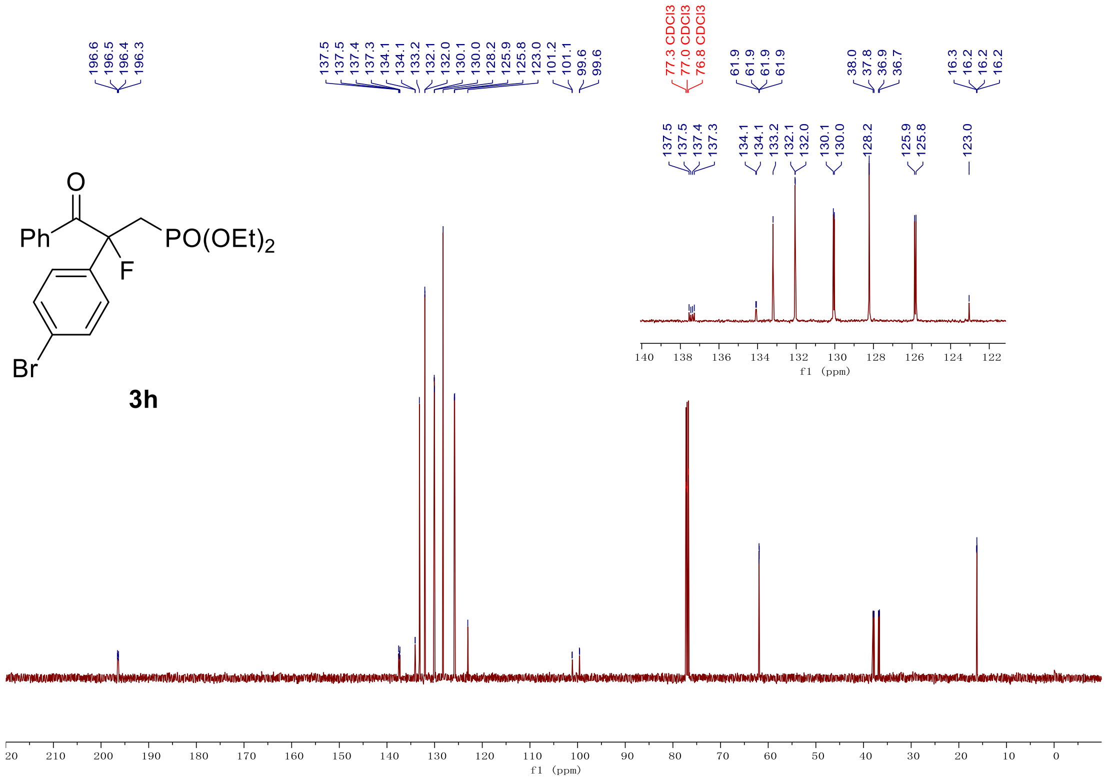

Supplementary Figure 83. ^{13}C NMR (125 MHz, CDCl3) spectrum for compound **3h**

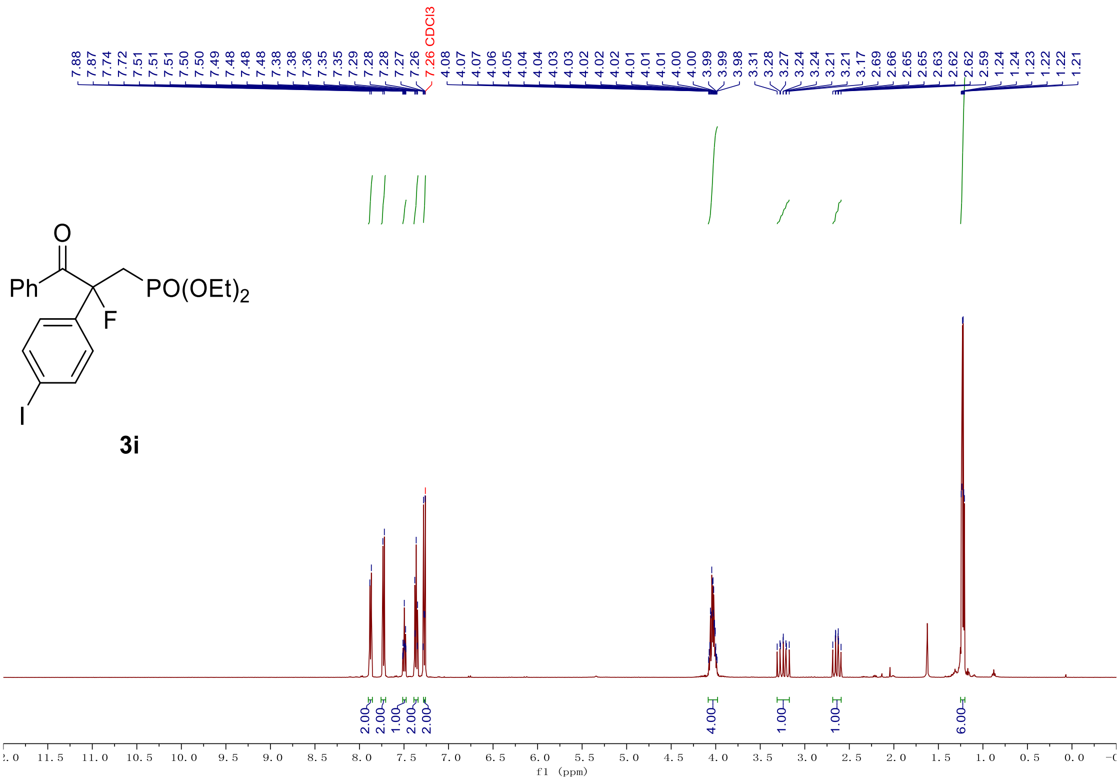

Supplementary Figure 84. ^{1}H NMR (500 MHz, $CDCl_3$) spectrum for compound **3i**

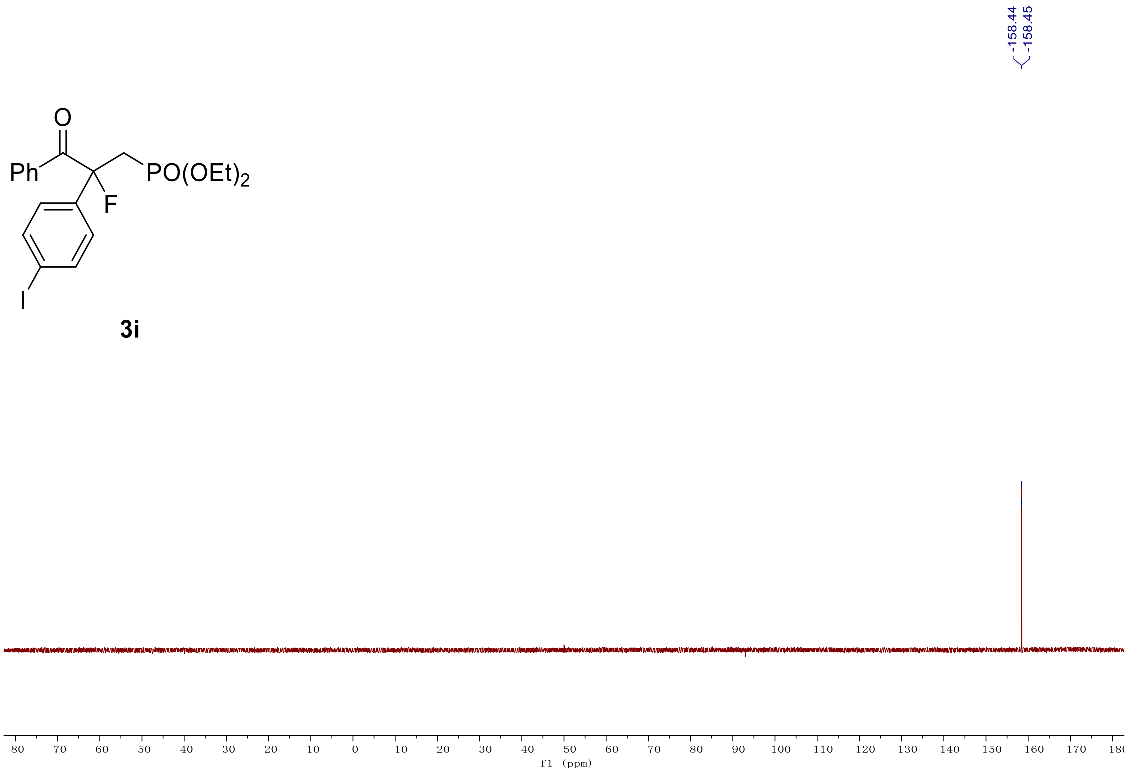

Supplementary Figure 85. ^{19}F NMR (471 MHz, $CDCl_3$) spectrum for compound **3i**

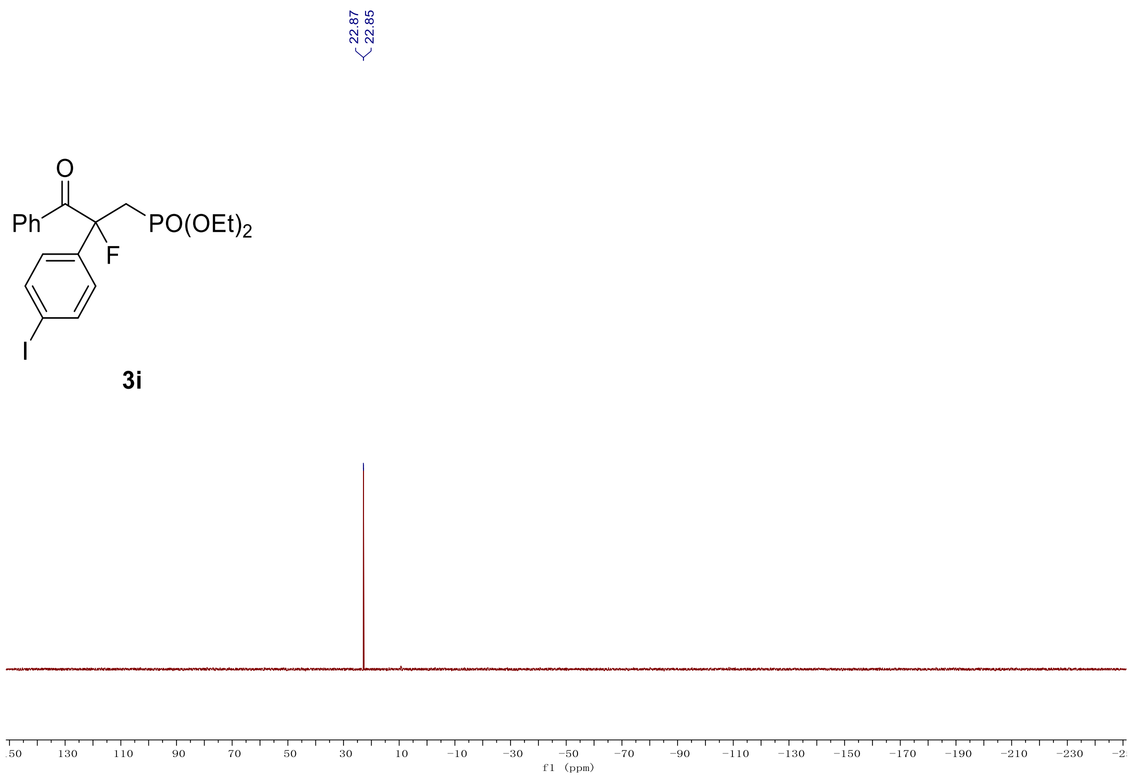

Supplementary Figure 86. ^{31}P NMR (202 MHz, $CDCl_3$) spectrum for compound **3i**

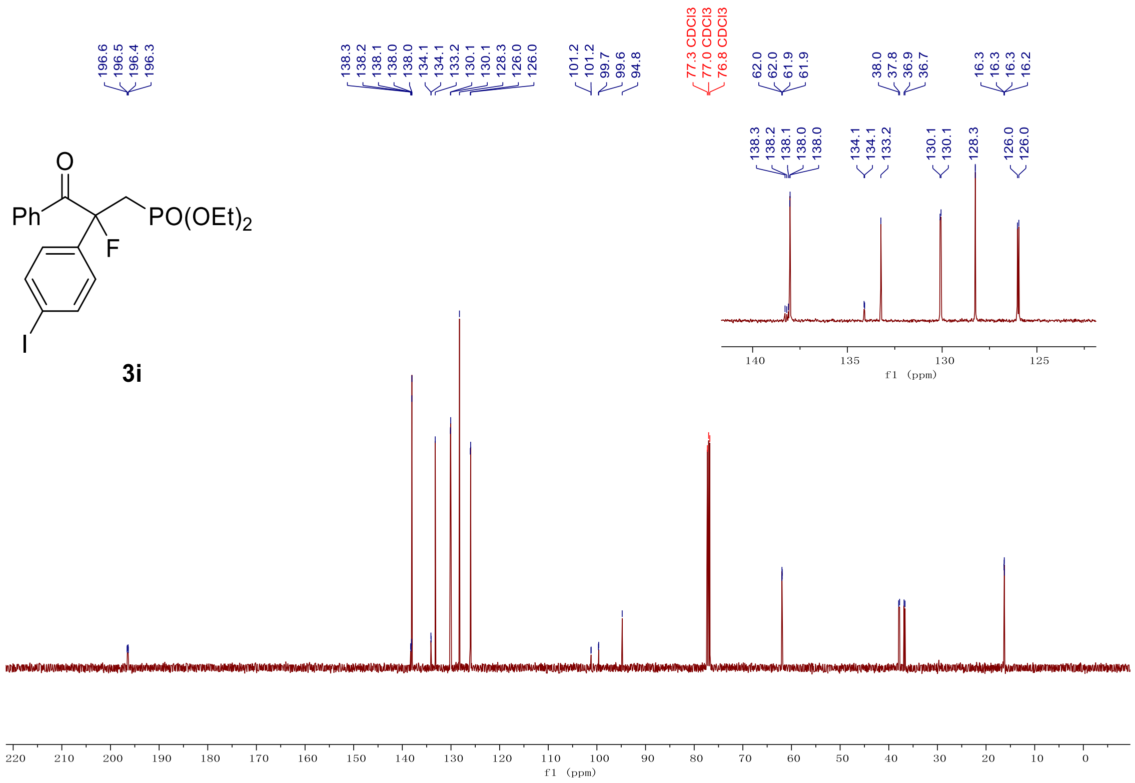

Supplementary Figure 87. ^{13}C NMR (125 MHz, $CDCl_3$) spectrum for compound **3i**

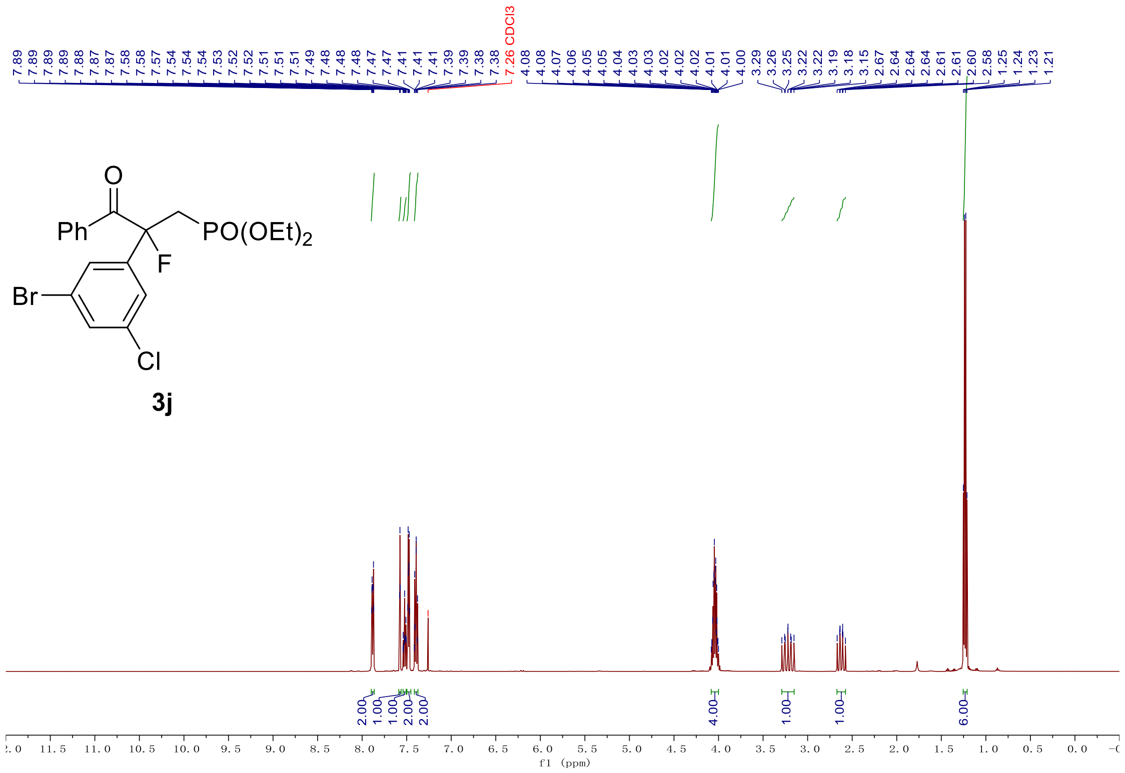

Supplementary Figure 88. ^{1}H NMR (500 MHz, $CDCl_3$) spectrum for compound **3j**

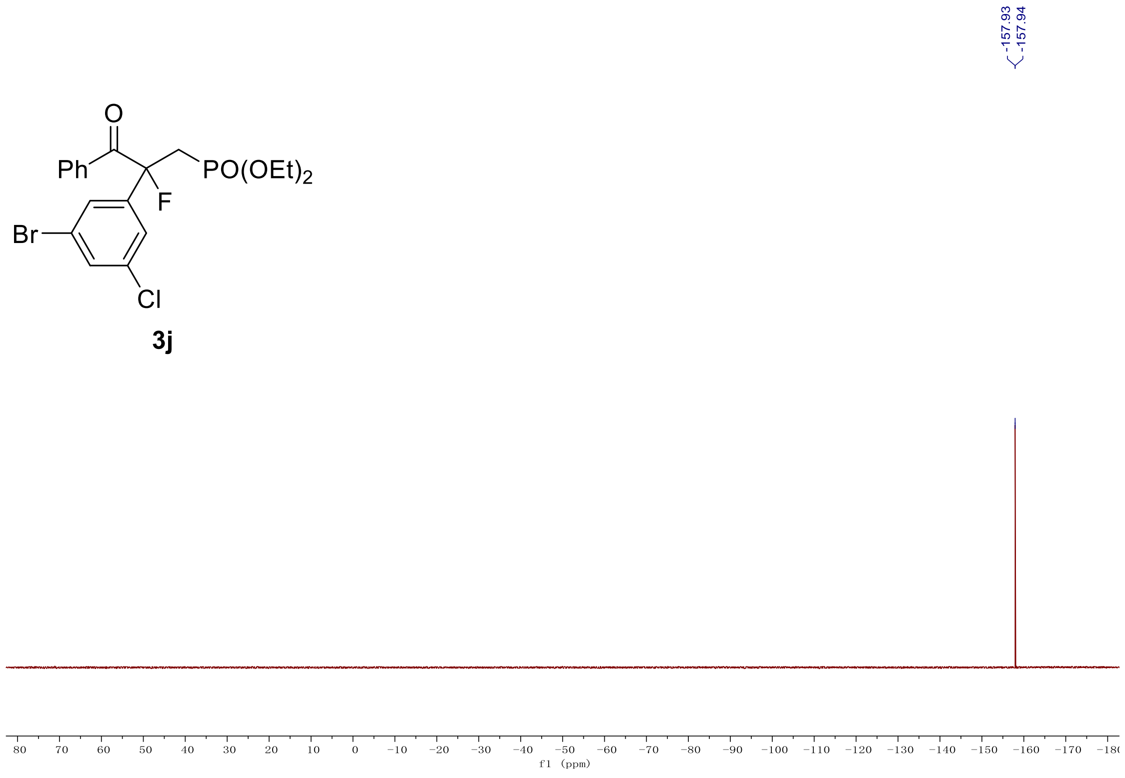

Supplementary Figure 89. ^{19}F NMR (471 MHz, $CDCl_3$) spectrum for compound **3j**

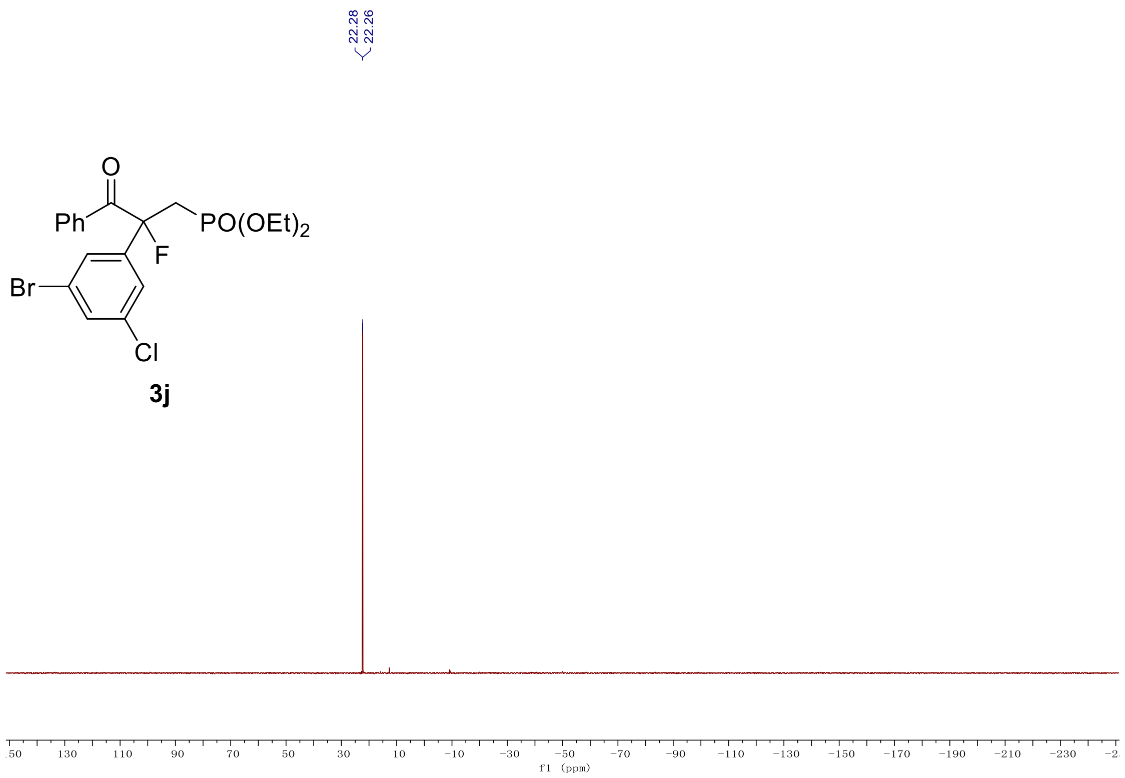

Supplementary Figure 90. ^{31}P NMR (202 MHz, CDCl$_3$) spectrum for compound **3j**

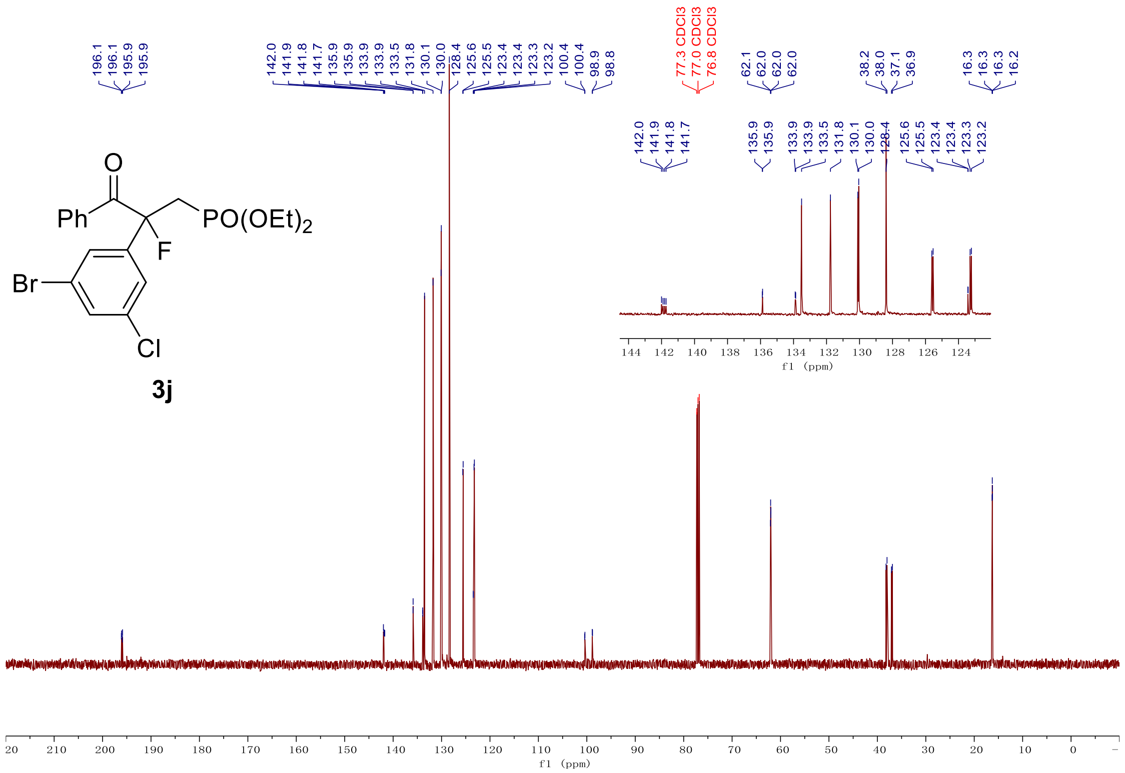

Supplementary Figure 91. ^{13}C NMR (125 MHz, CDCl$_3$) spectrum for compound **3j**

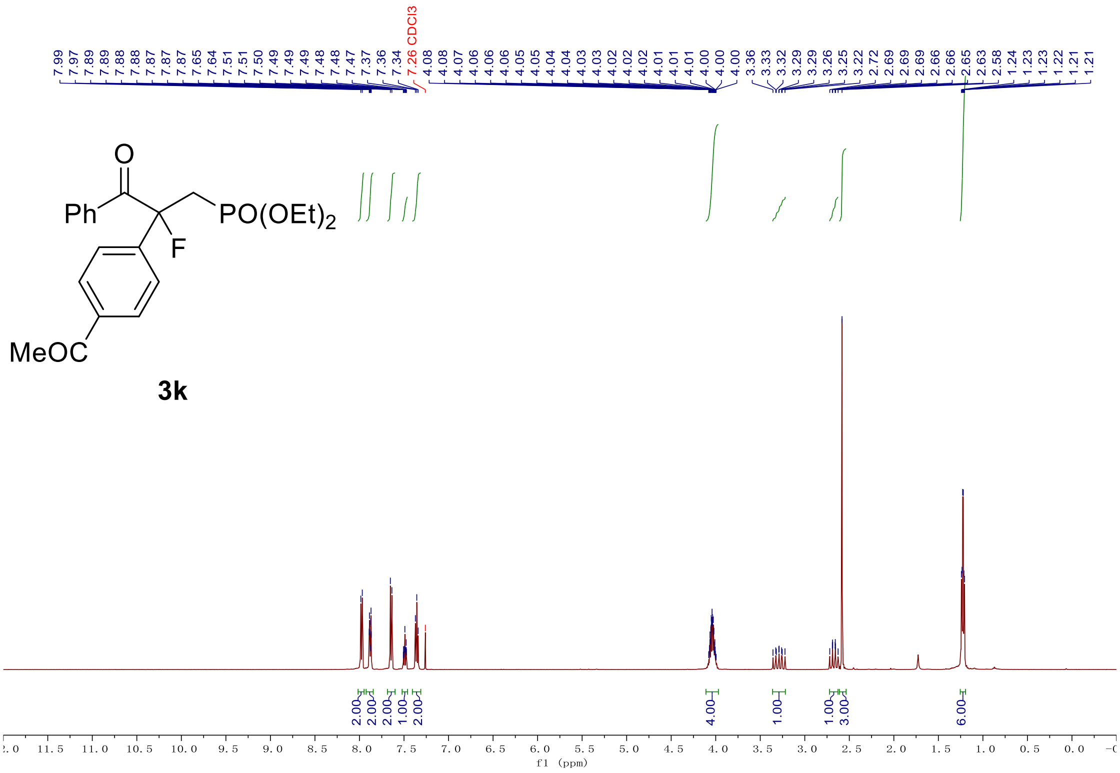

Supplementary Figure 92. ^{1}H NMR (500 MHz, $CDCl_3$) spectrum for compound **3k**

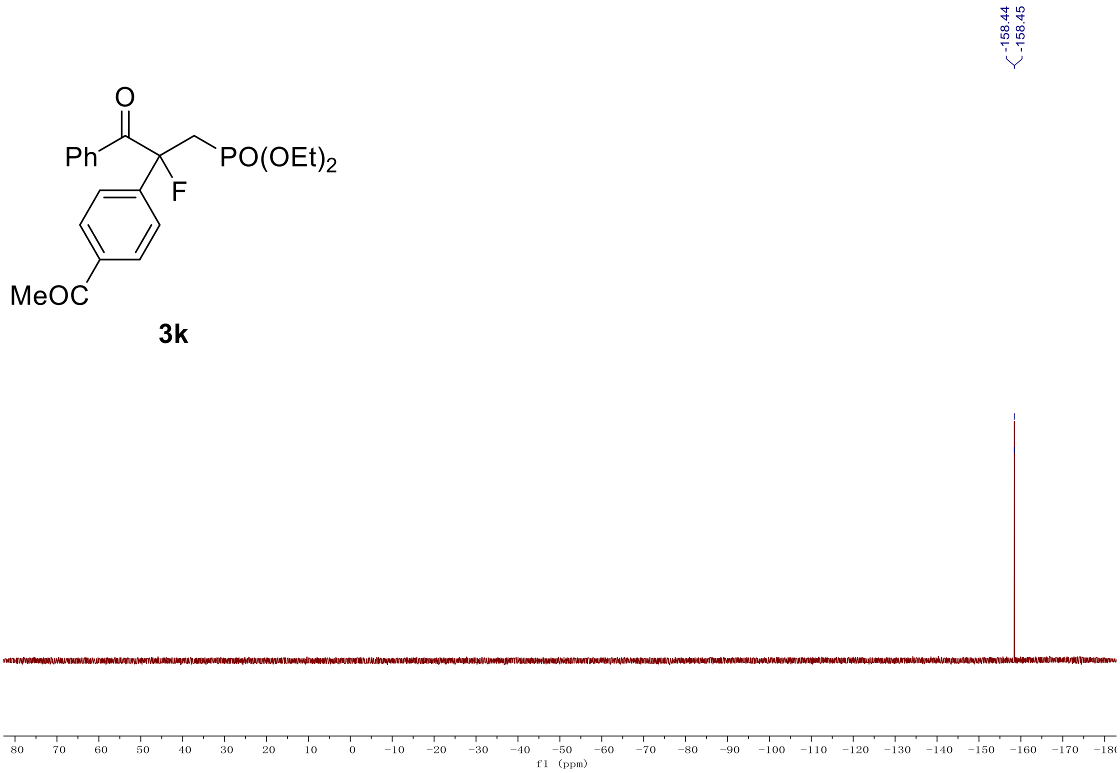

Supplementary Figure 93. ^{19}F NMR (125 MHz, $CDCl_3$) spectrum for compound **3k**

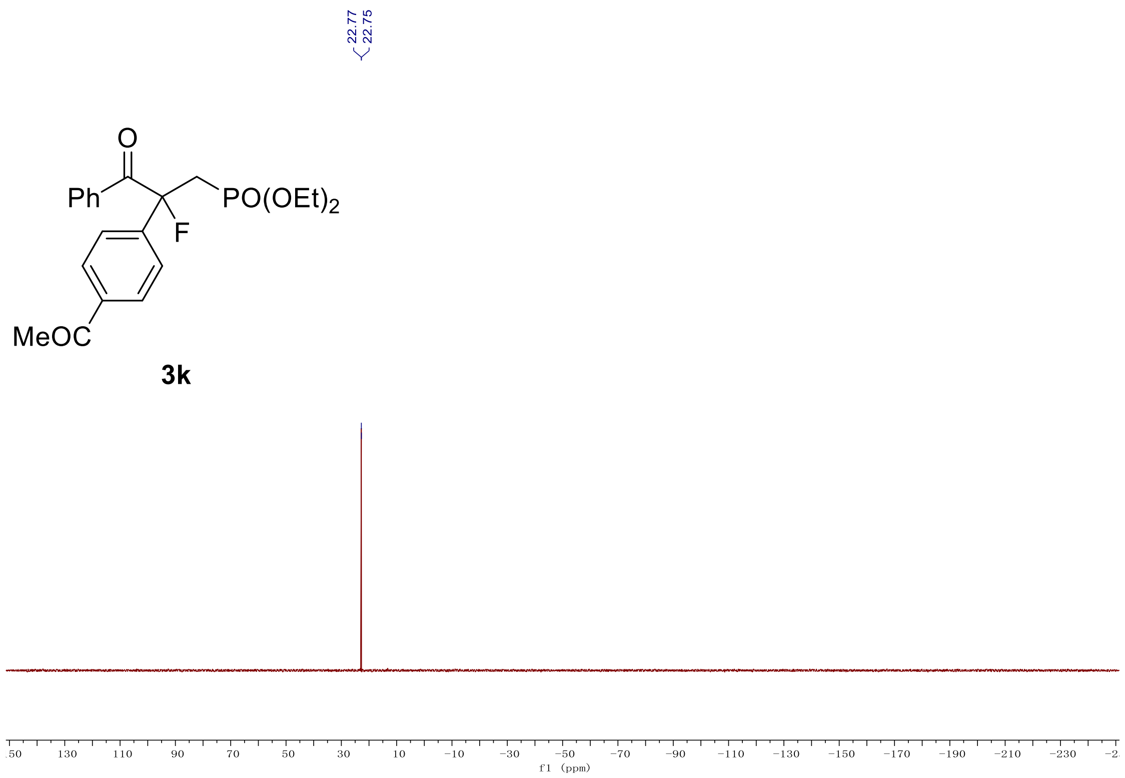

Supplementary Figure 94. ^{31}P NMR (202 MHz, $CDCl_3$) spectrum for compound **3k**

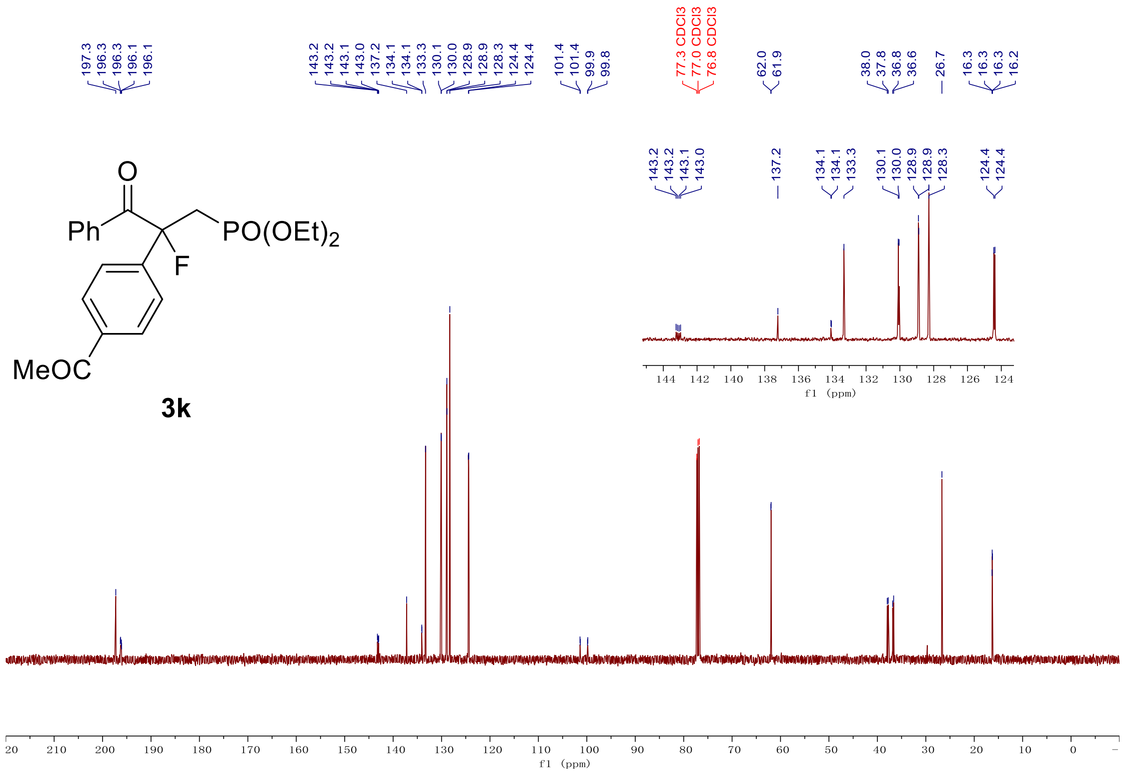

Supplementary Figure 95. ^{13}C NMR (125 MHz, $CDCl_3$) spectrum for compound **3k**

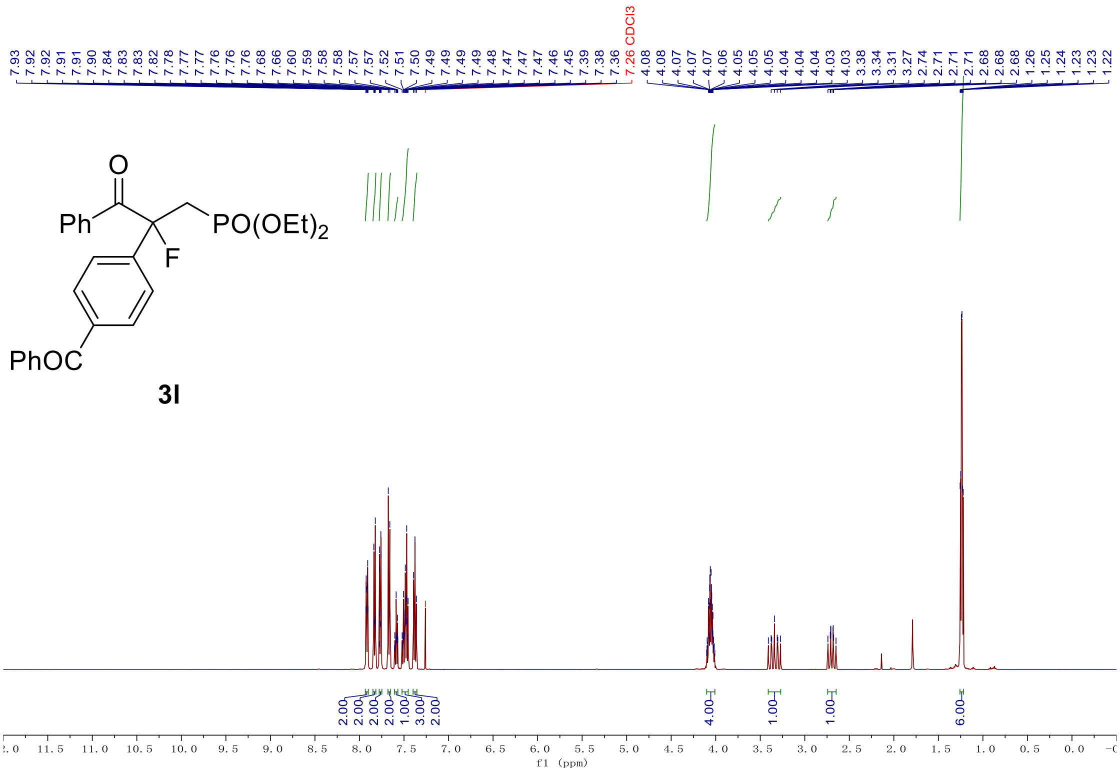

Supplementary Figure 96. ^{1}H NMR (500 MHz, CDCl$_3$) spectrum for compound **3l**

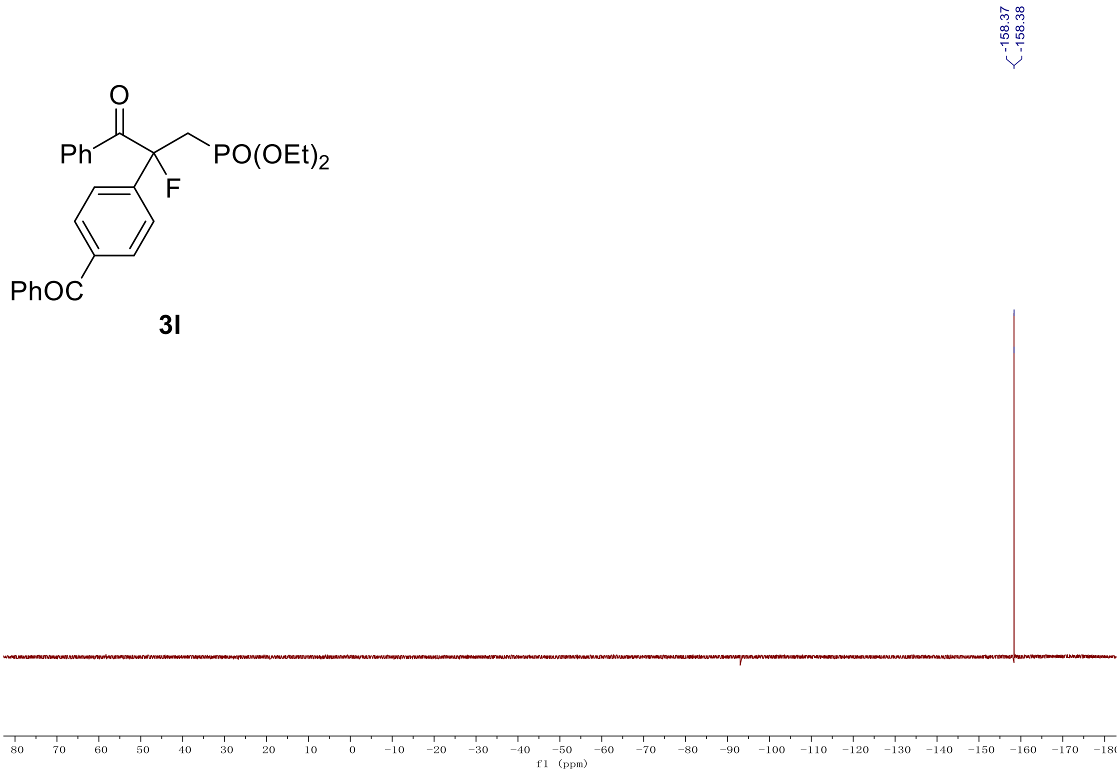

Supplementary Figure 97. ^{19}F NMR (471 MHz, CDCl$_3$) spectrum for compound **3l**

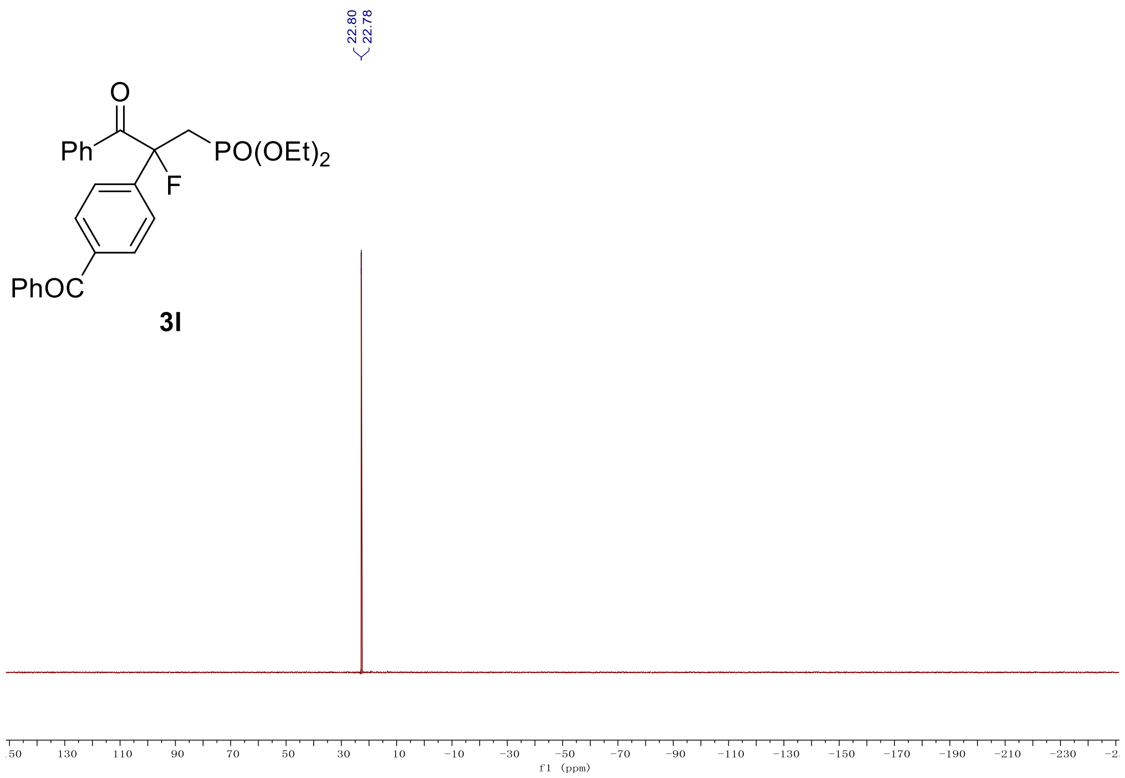

Supplementary Figure 98. ^{31}P NMR (202 MHz, $CDCl_3$) spectrum for compound **3l**

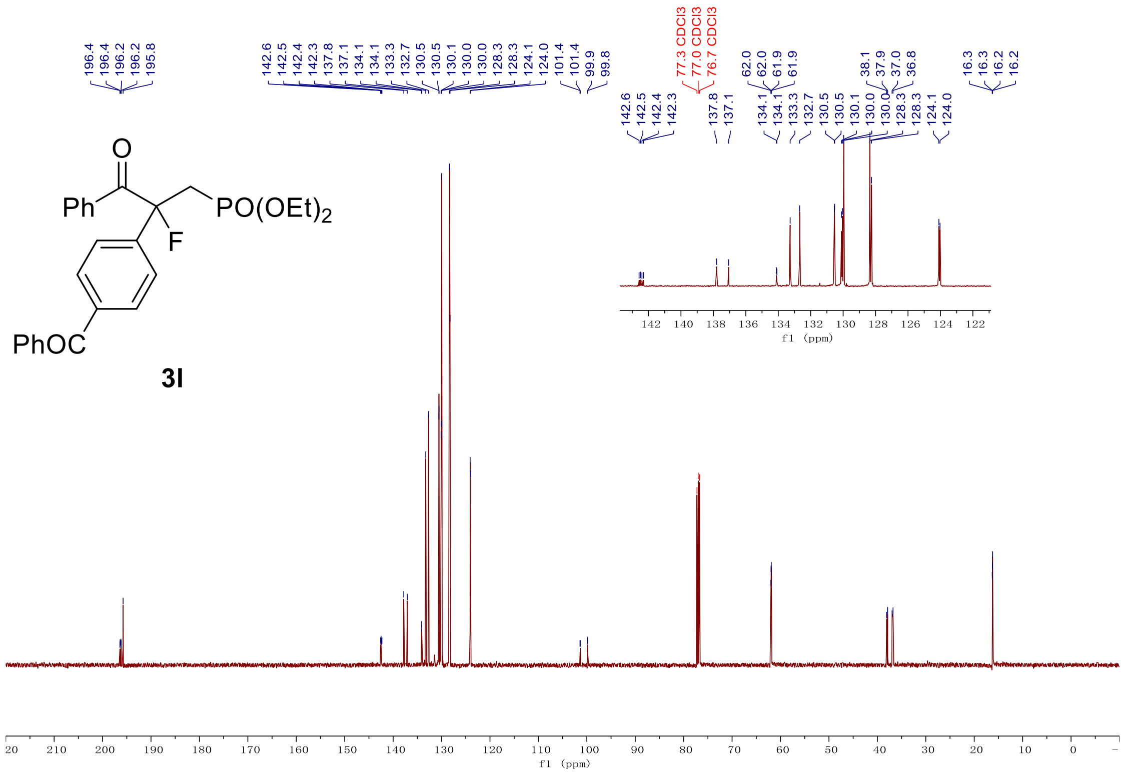

Supplementary Figure 99. ^{13}C NMR (125 MHz, $CDCl_3$) spectrum for compound **3l**

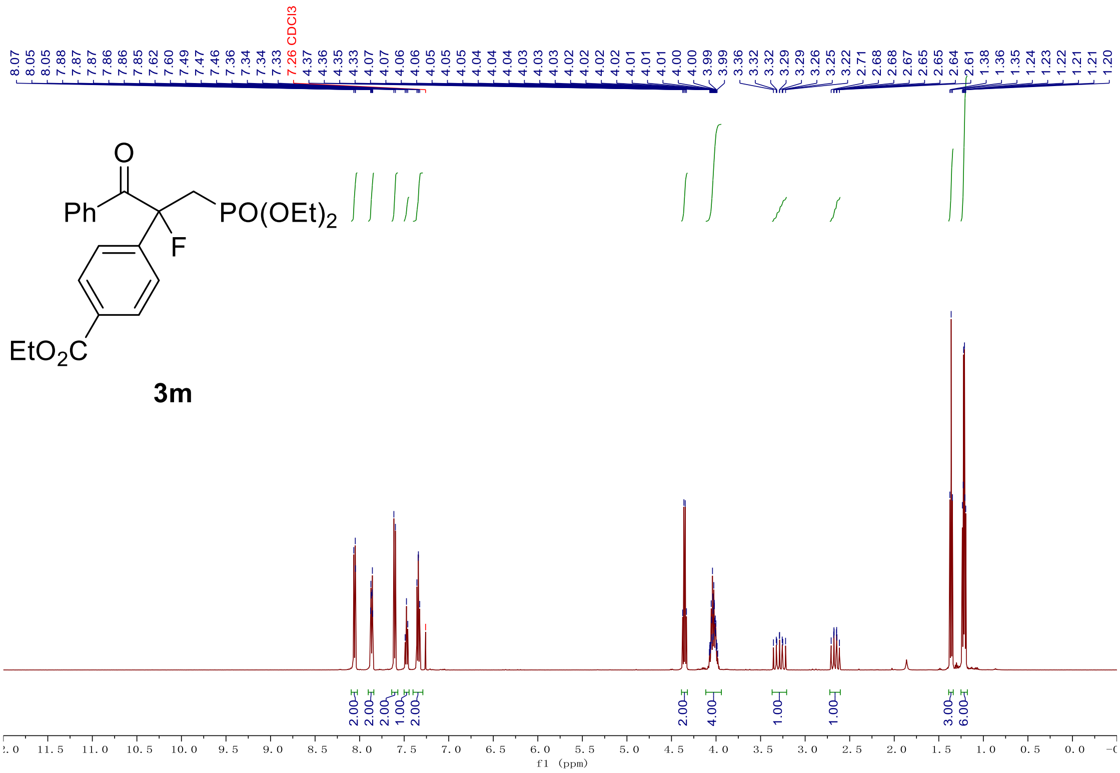

Supplementary Figure 100. ^{1}H NMR (500 MHz, $CDCl_3$) spectrum for compound **3m**

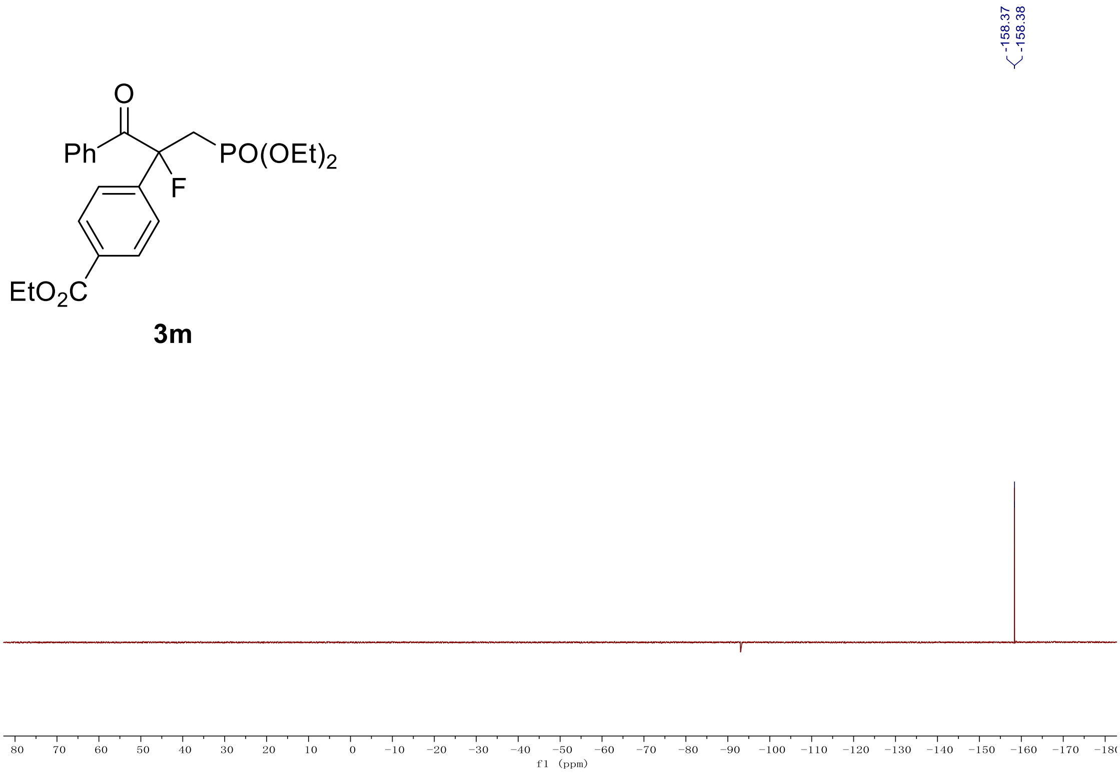

Supplementary Figure 101. ^{19}F NMR (471 MHz, $CDCl_3$) spectrum for compound **3m**

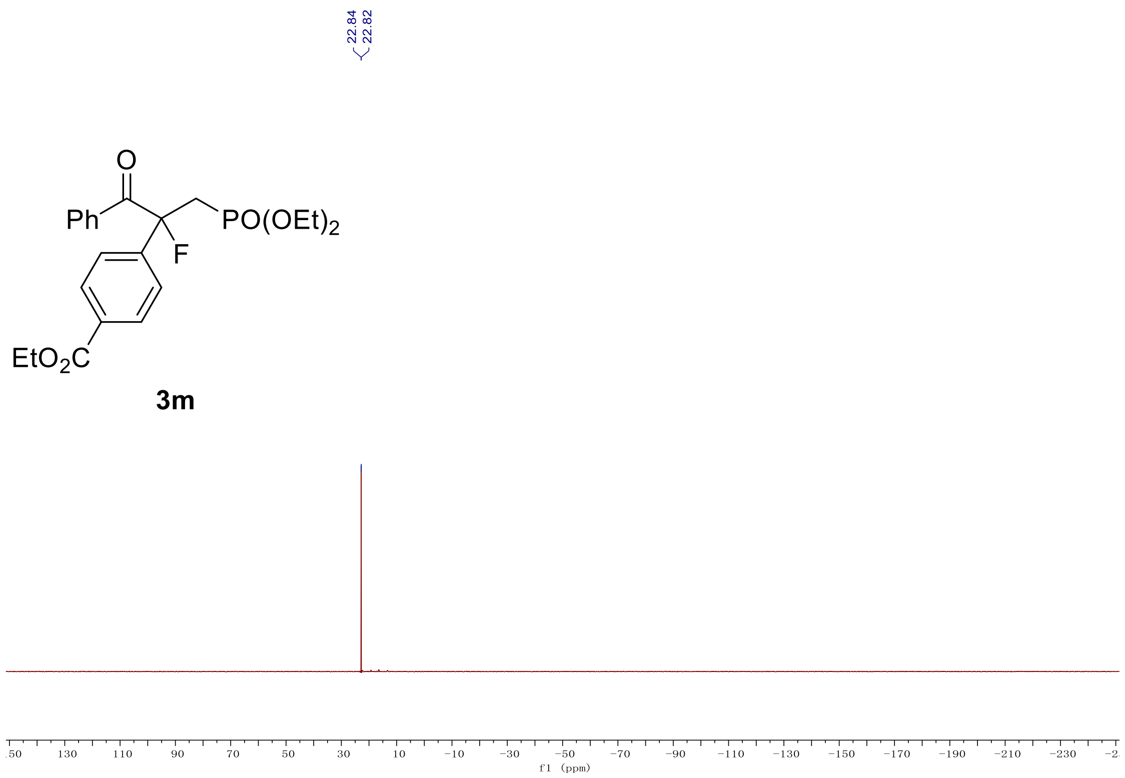

Supplementary Figure 102. ^{31}P NMR (202 MHz, $CDCl_3$) spectrum for compound **3m**

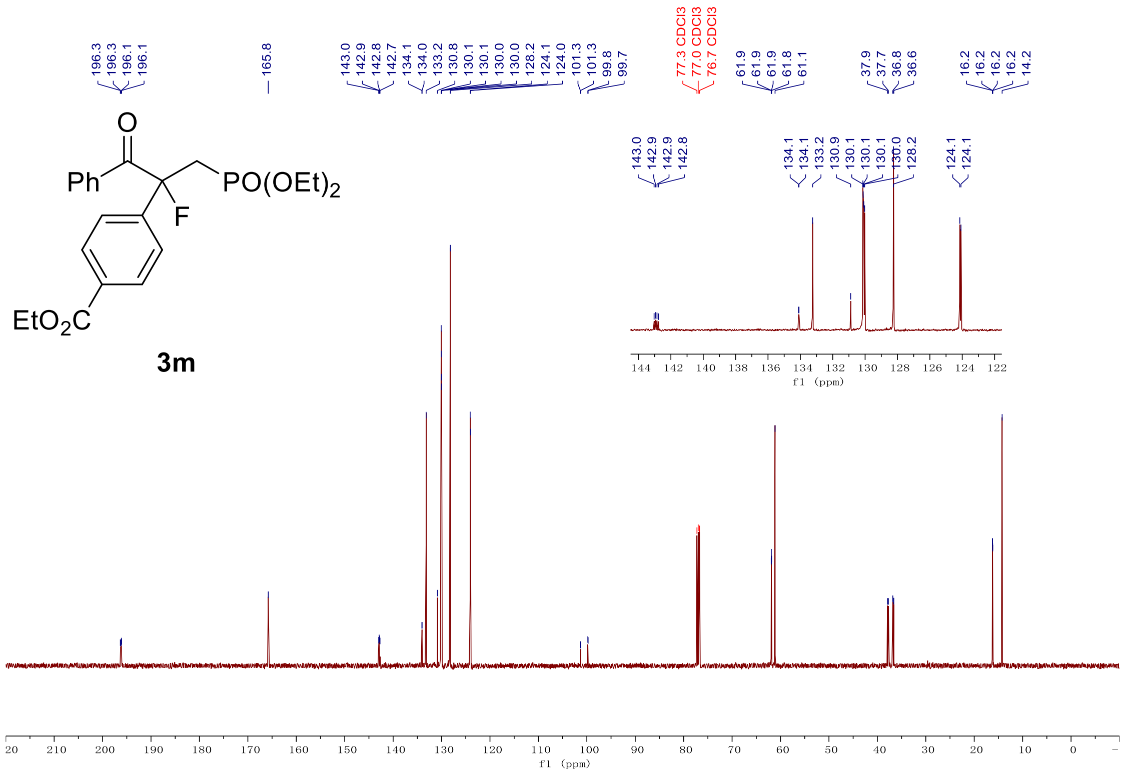

Supplementary Figure 103. ^{13}C NMR (125 MHz, $CDCl_3$) spectrum for compound **3m**

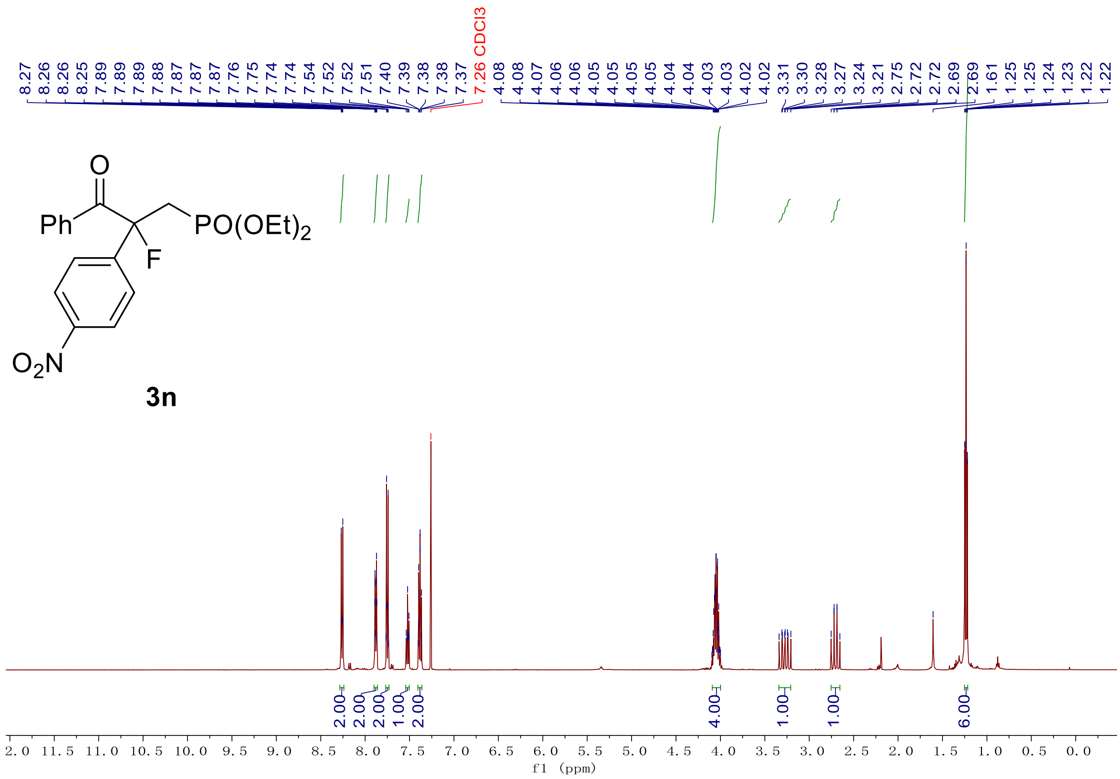

Supplementary Figure 104. ^{1}H NMR (500 MHz, $CDCl_3$) spectrum for compound **3n**

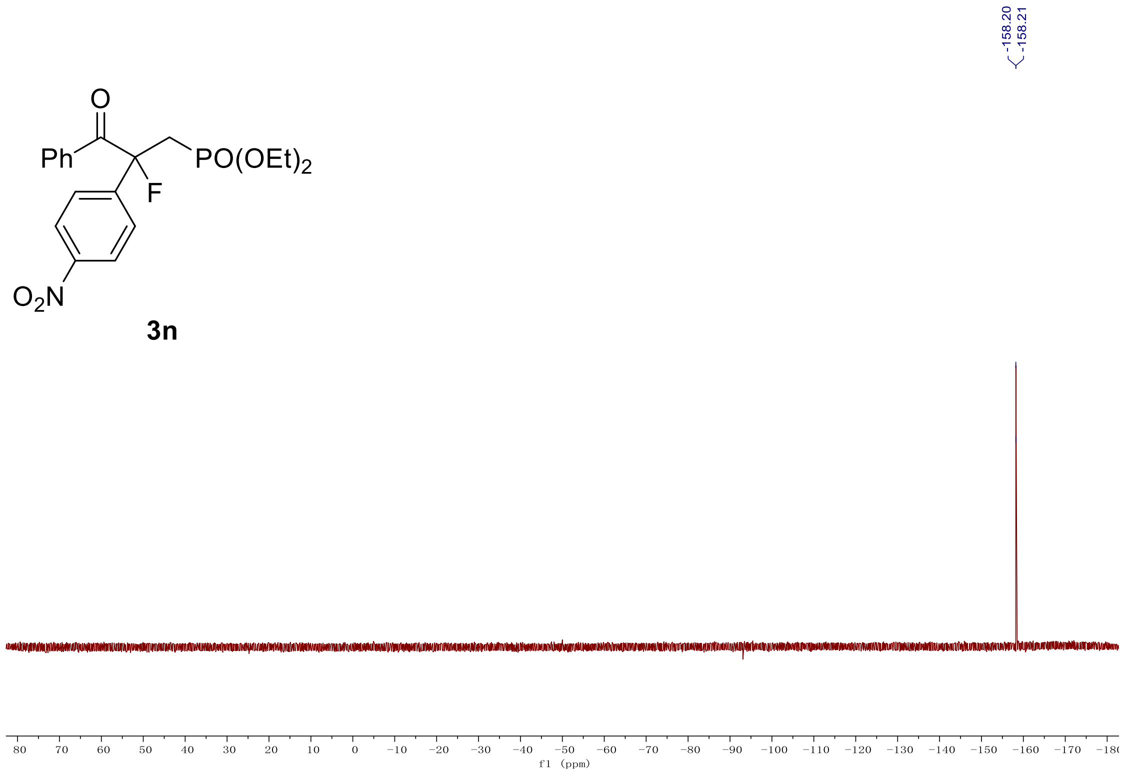

Supplementary Figure 105. ^{19}F NMR (471 MHz, $CDCl_3$) spectrum for compound **3n**

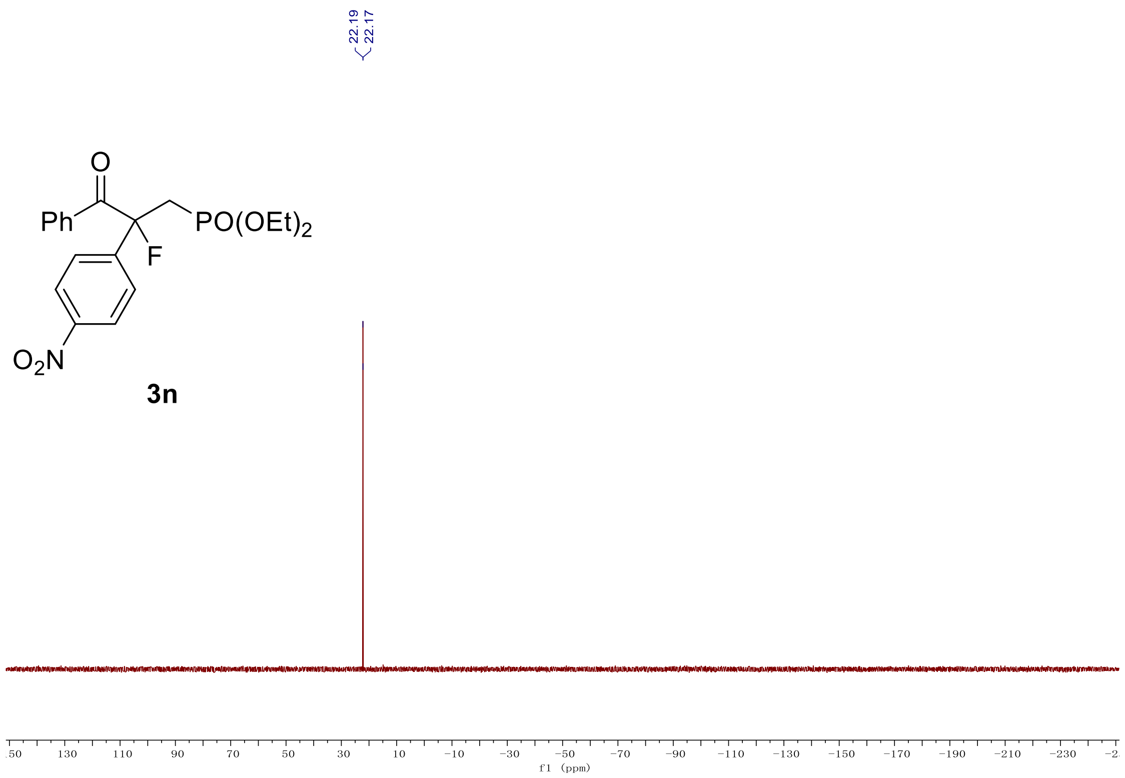

Supplementary Figure 106. ^{31}P NMR (202 MHz, $CDCl_3$) spectrum for compound **3n**

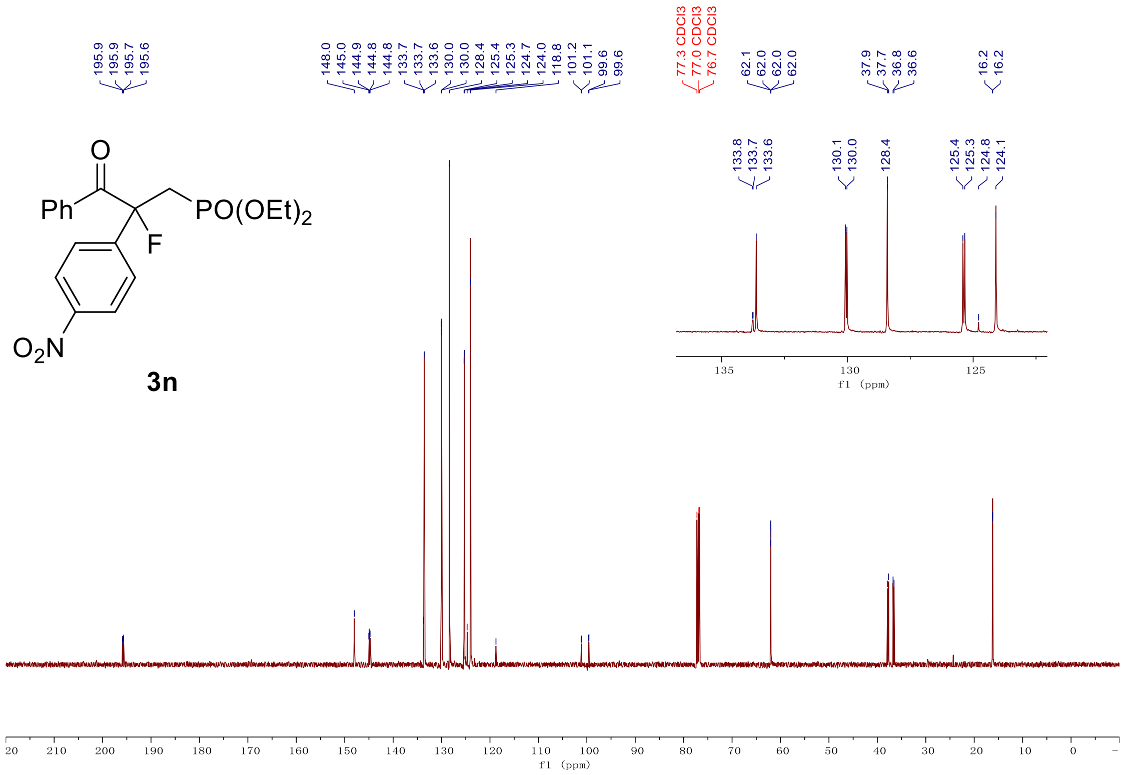

Supplementary Figure 107. ^{13}C NMR (125 MHz, $CDCl_3$) spectrum for compound **3n**

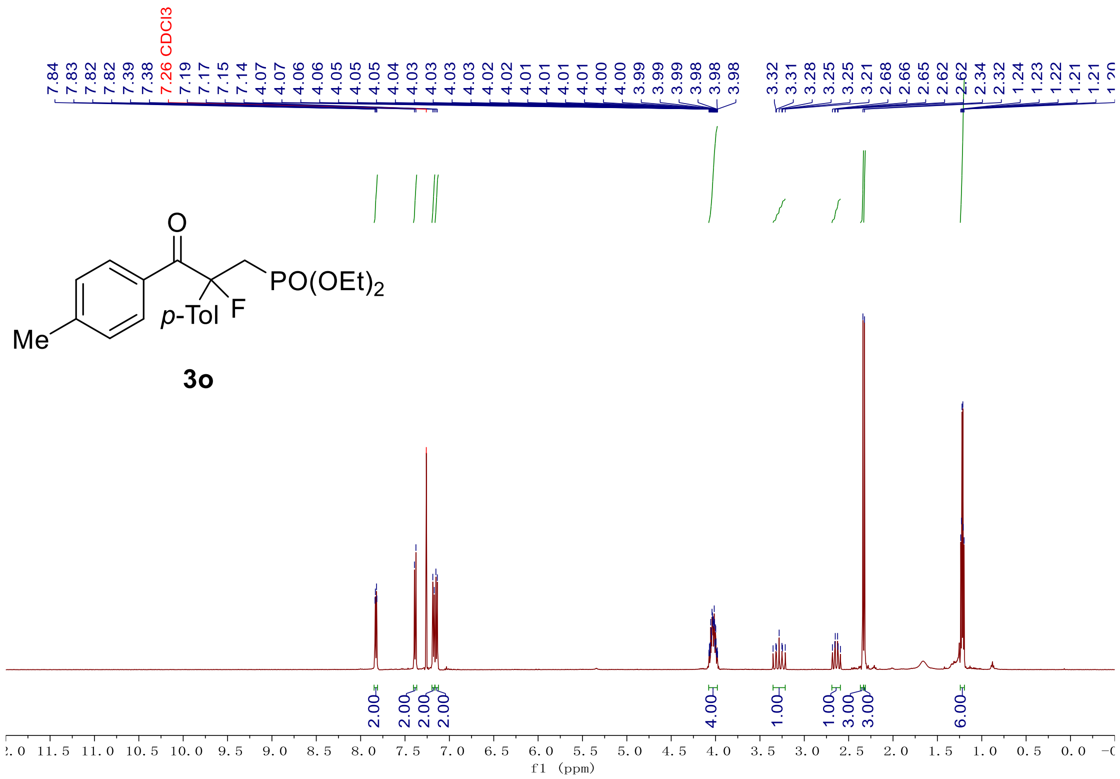

Supplementary Figure 108. ^{1}H NMR (500 MHz, $CDCl_3$) spectrum for compound **3o**

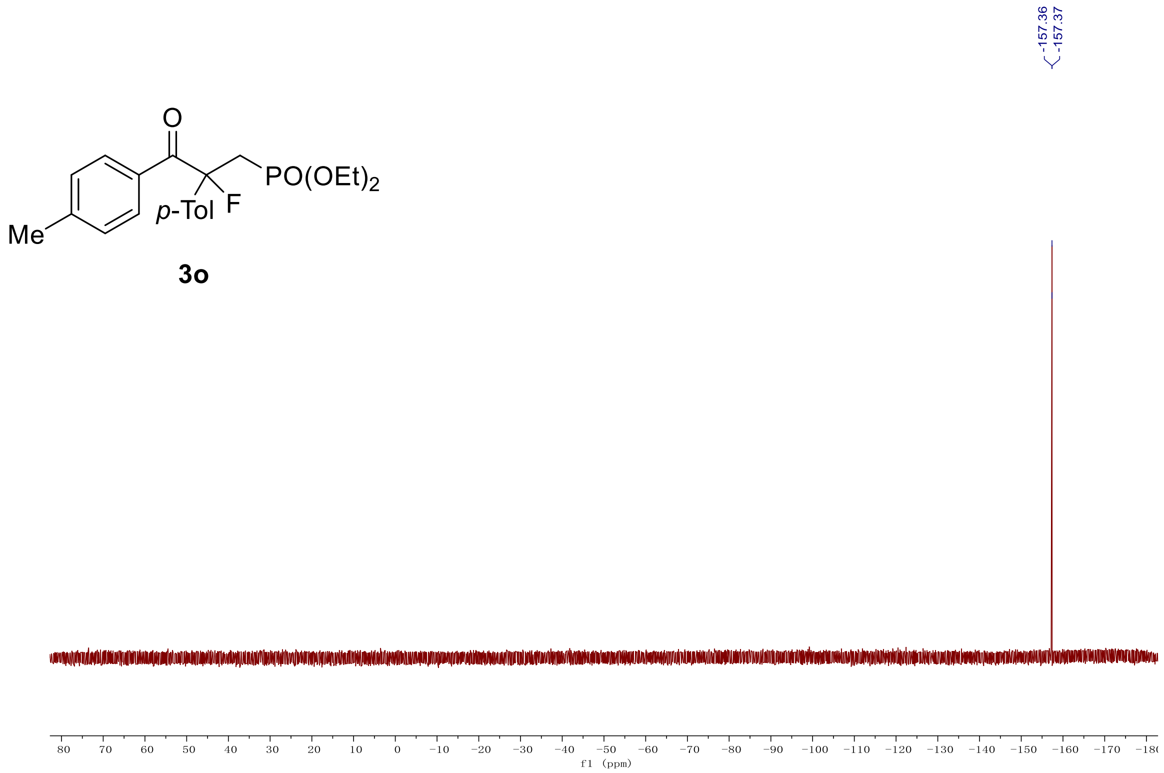

Supplementary Figure 109. ^{19}F NMR (471 MHz, $CDCl_3$) spectrum for compound **3o**

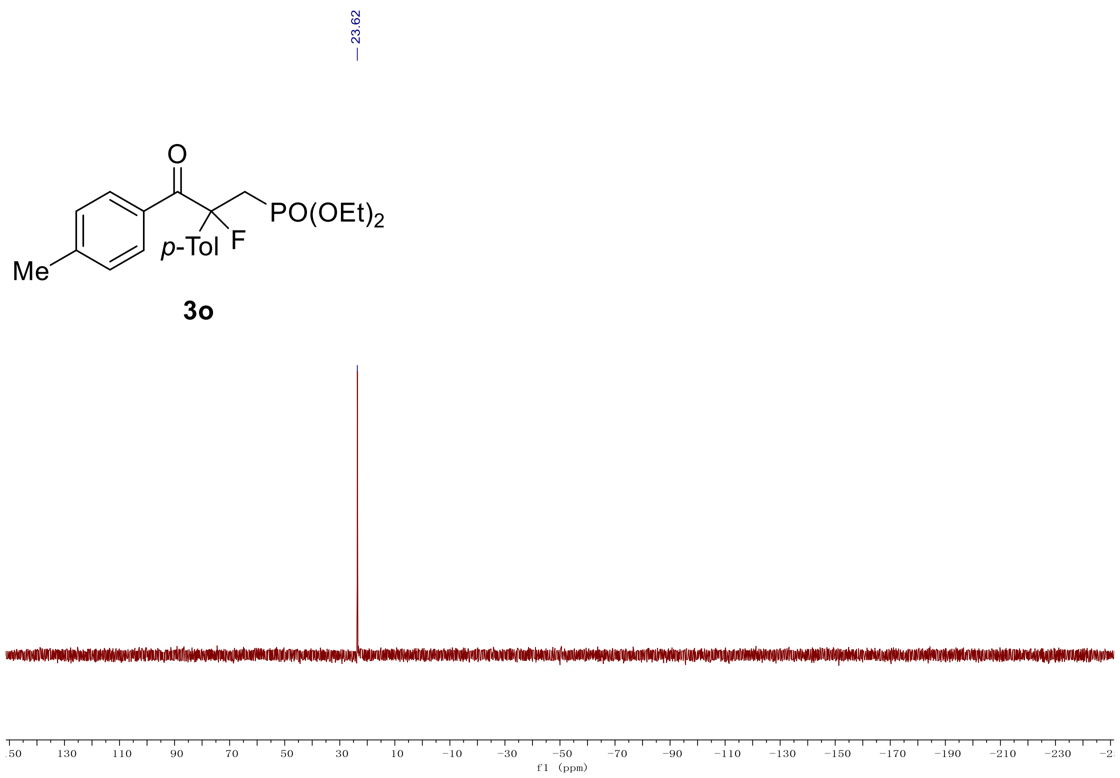

Supplementary Figure 110. ^{31}P NMR (202 MHz, $CDCl_3$) spectrum for compound **3o**

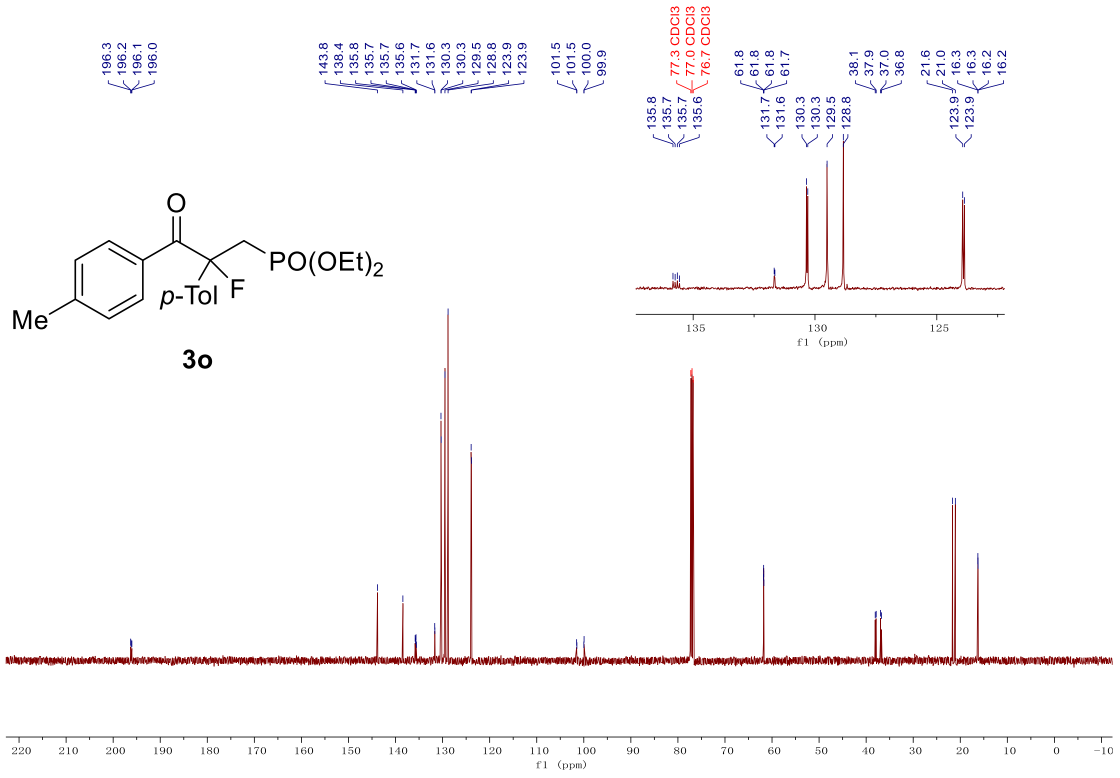

Supplementary Figure 111. ^{13}C NMR (125 MHz, $CDCl_3$) spectrum for compound **3o**

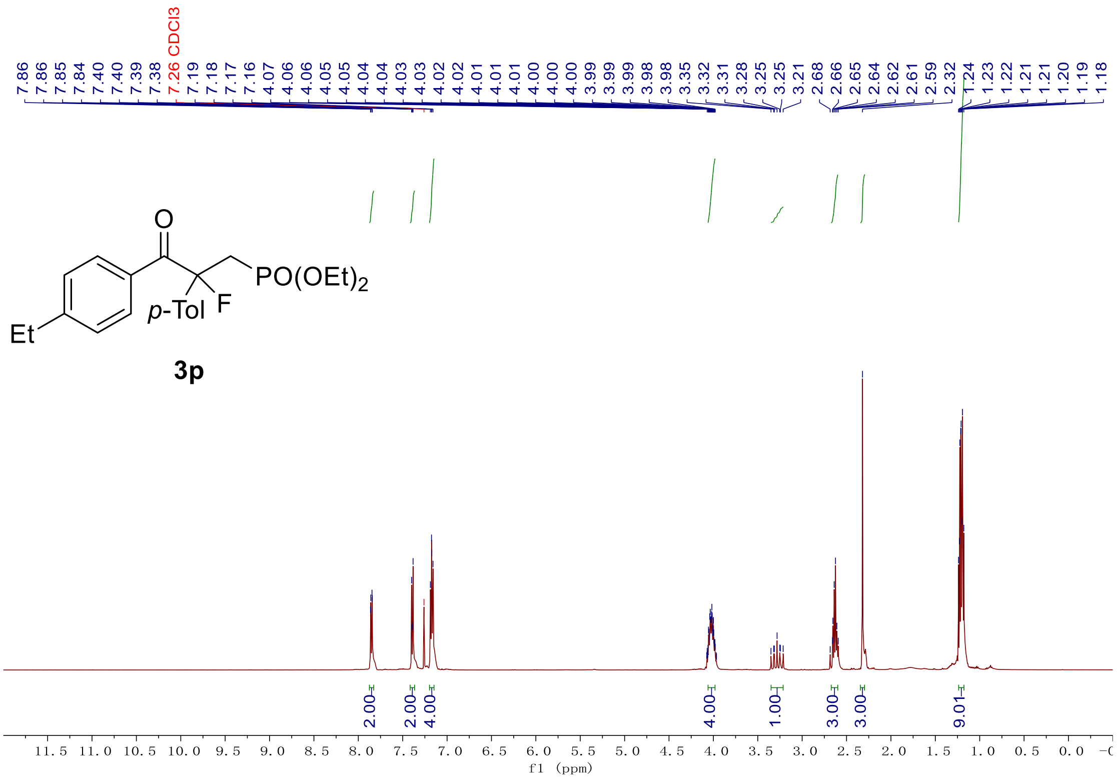

Supplementary Figure 112. ^{1}H NMR (500 MHz, $CDCl_3$) spectrum for compound **3p**

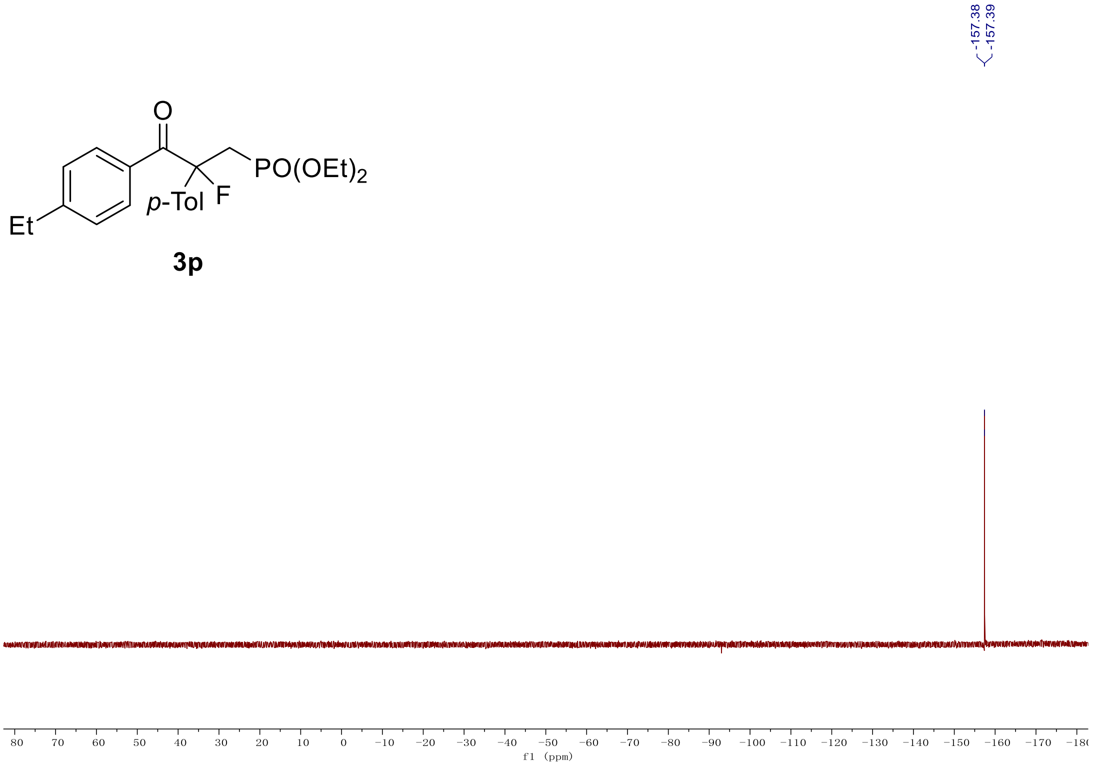

Supplementary Figure 113. ^{19}F NMR (471 MHz, $CDCl_3$) spectrum for compound **3p**

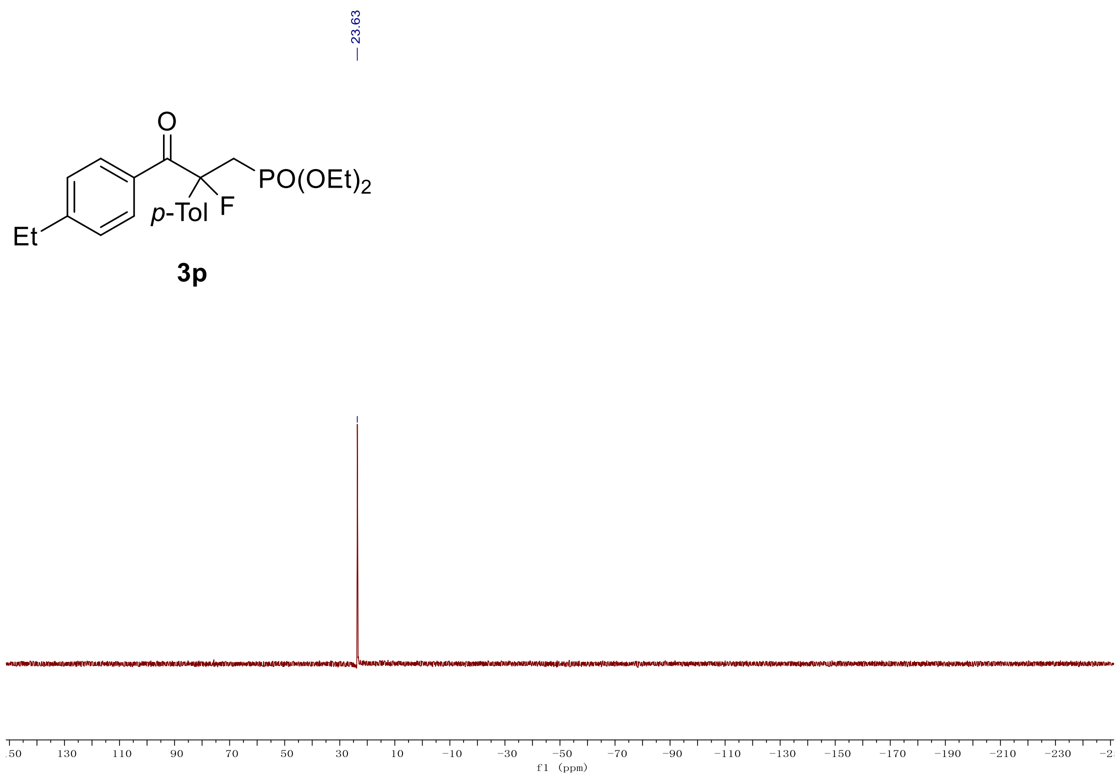

Supplementary Figure 114. ^{31}P NMR (202 MHz, CDCl$_3$) spectrum for compound **3p**

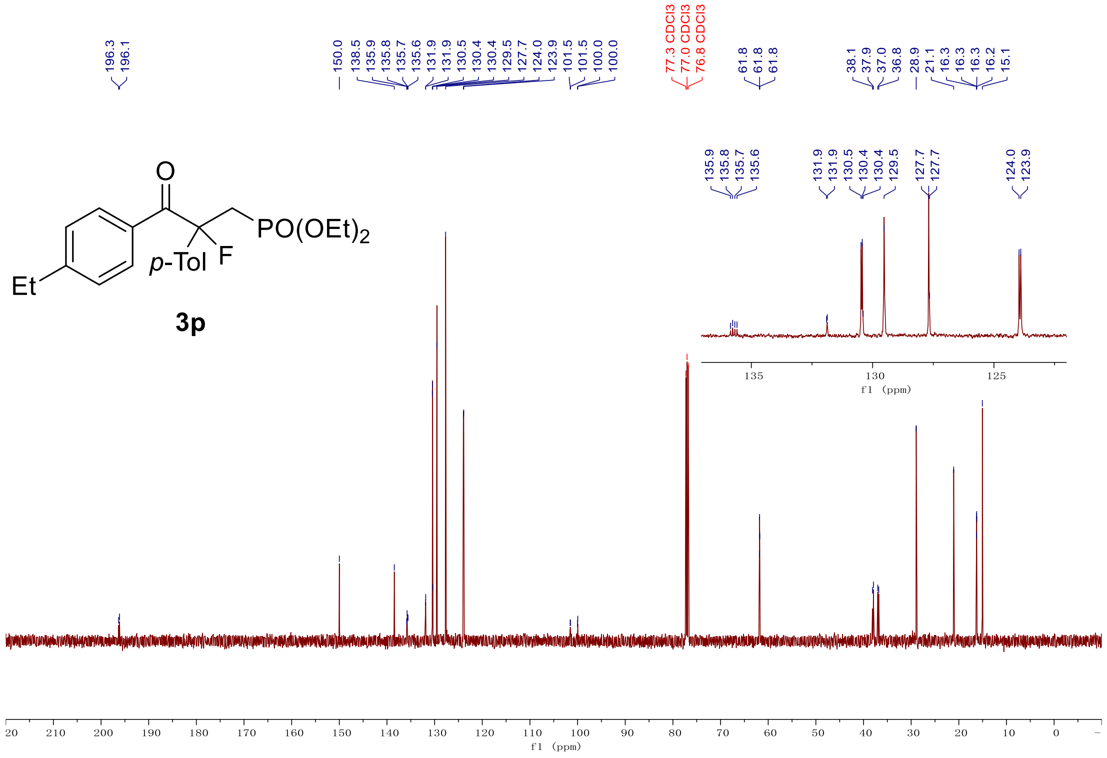

Supplementary Figure 115. ^{13}C NMR (125 MHz, CDCl$_3$) spectrum for compound **3p**

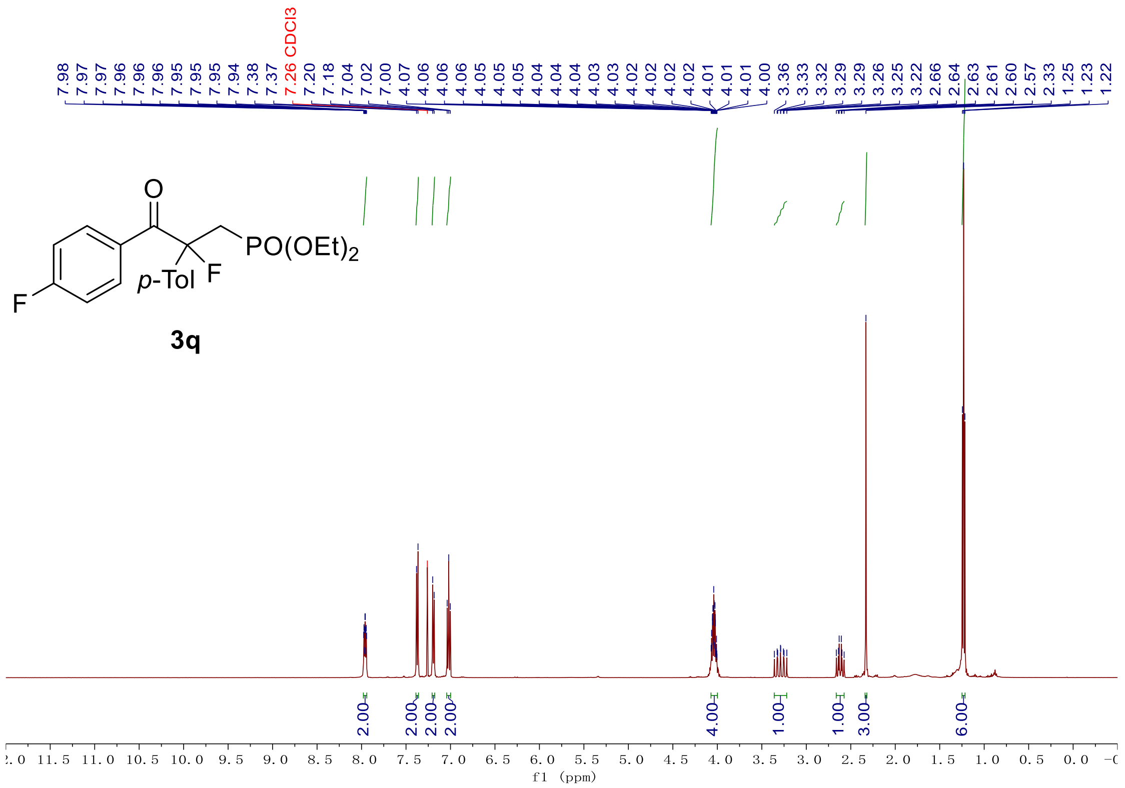

Supplementary Figure 116. ^{1}H NMR (500 MHz, CDCl$_3$) spectrum for compound **3q**

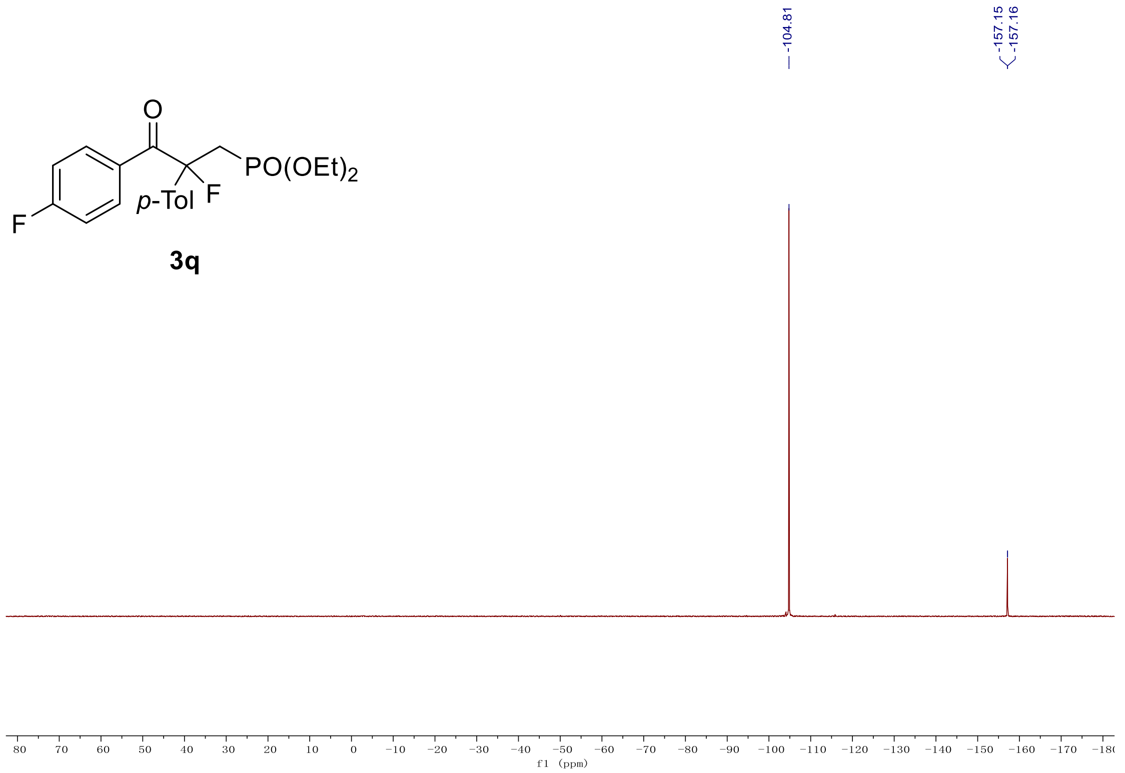

Supplementary Figure 117. ^{19}F NMR (471 MHz, CDCl$_3$) spectrum for compound **3q**

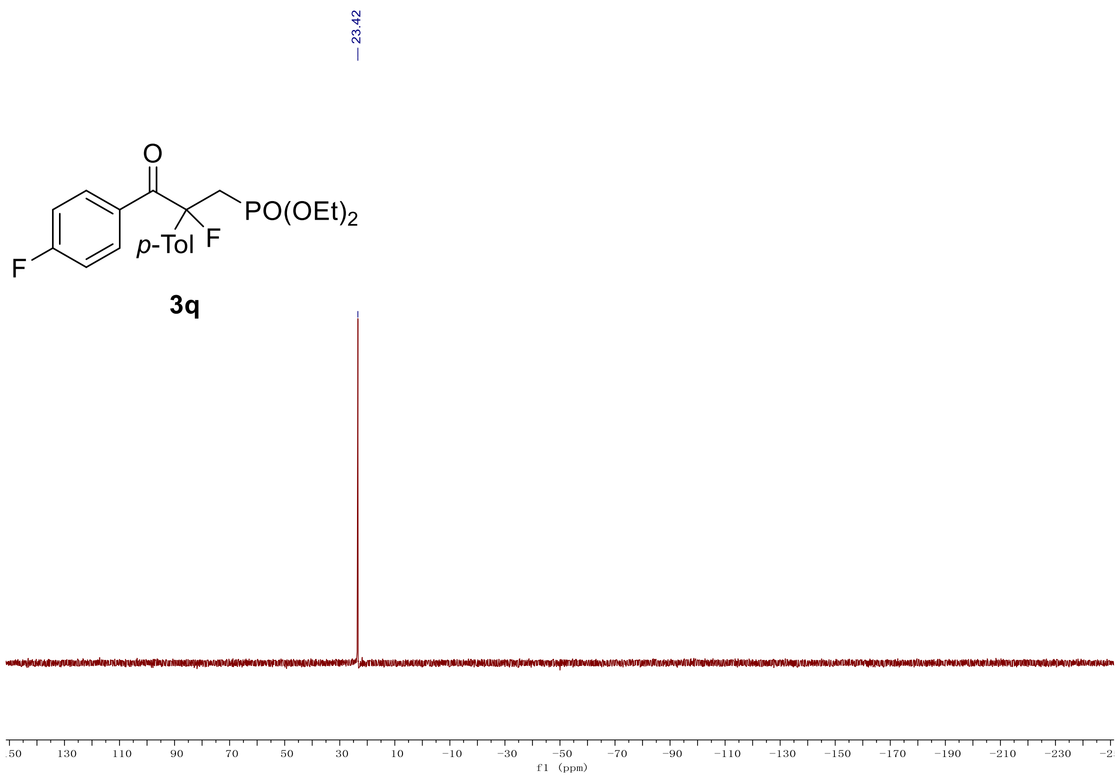

Supplementary Figure 118. ^{31}P NMR (202 MHz, CDCl$_3$) spectrum for compound **3q**

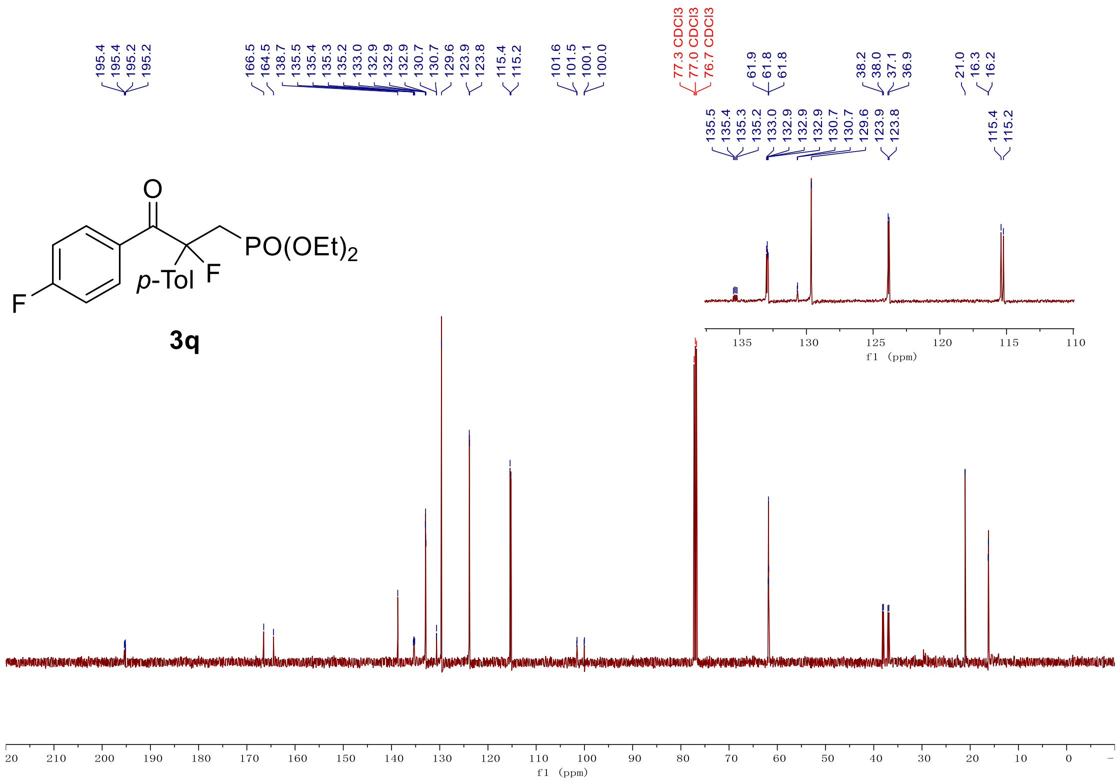

Supplementary Figure 119. ^{13}C NMR (125 MHz, CDCl$_3$) spectrum for compound **3q**

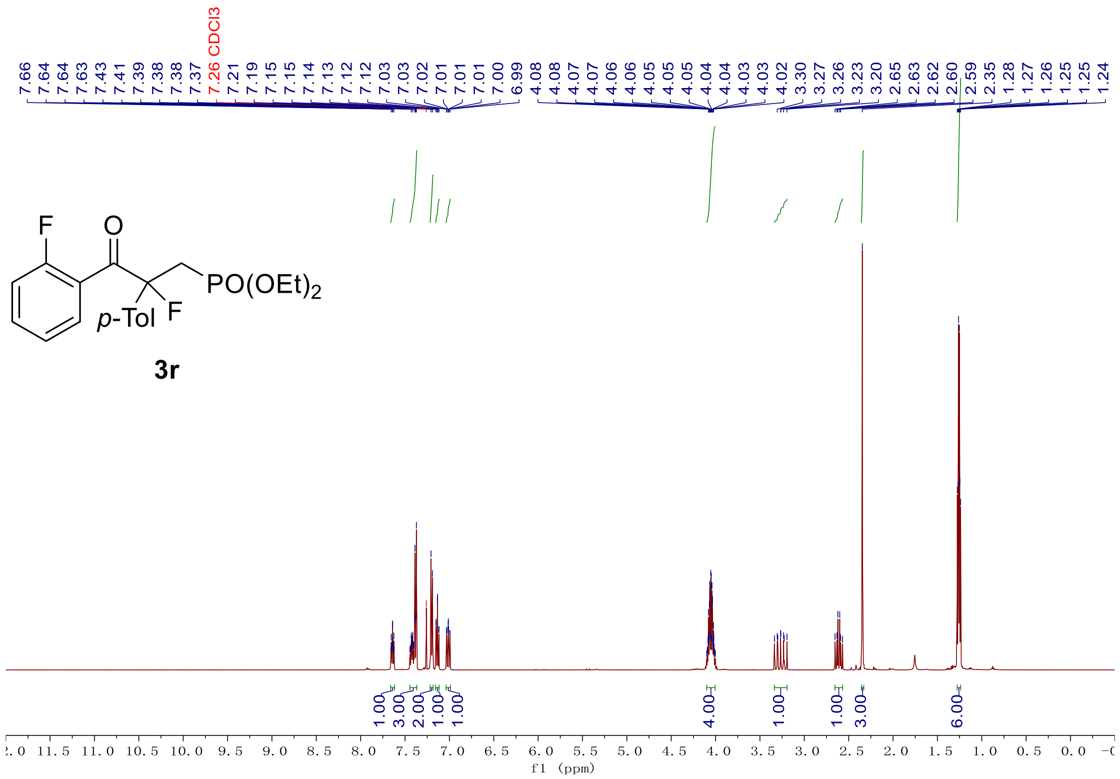

Supplementary Figure 120. ^{1}H NMR (500 MHz, $CDCl_3$) spectrum for compound **3r**

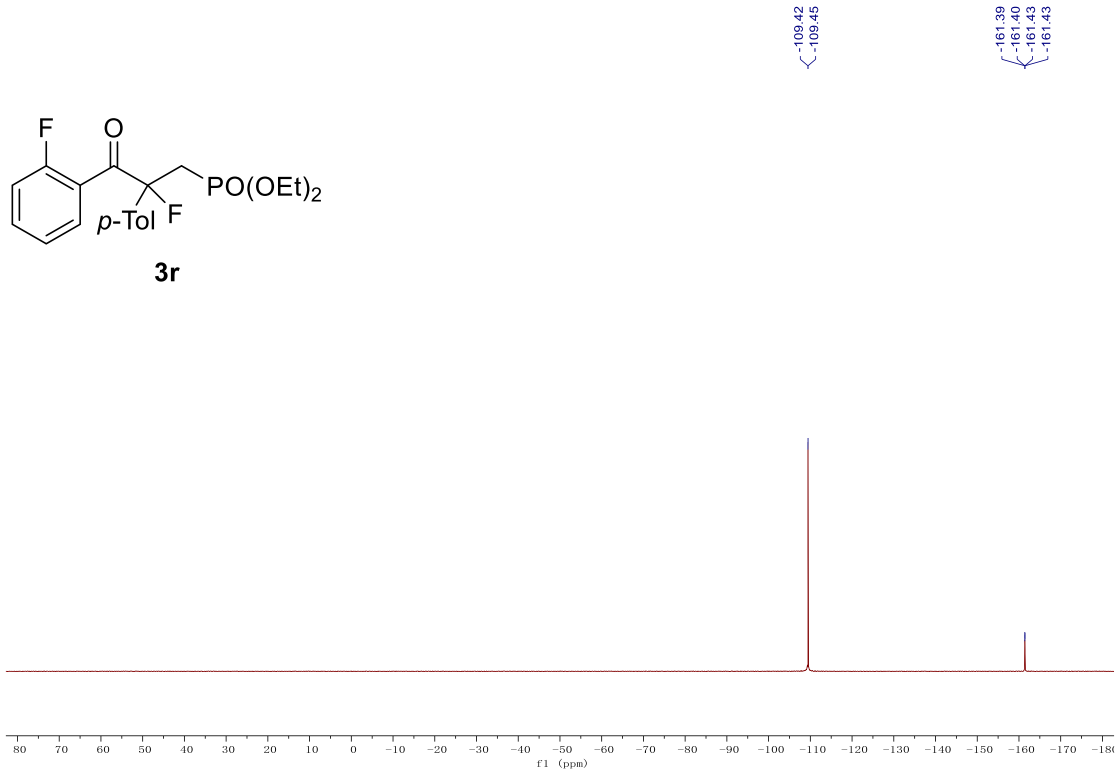

Supplementary Figure 121. ^{19}F NMR (471 MHz, $CDCl_3$) spectrum for compound **3r**

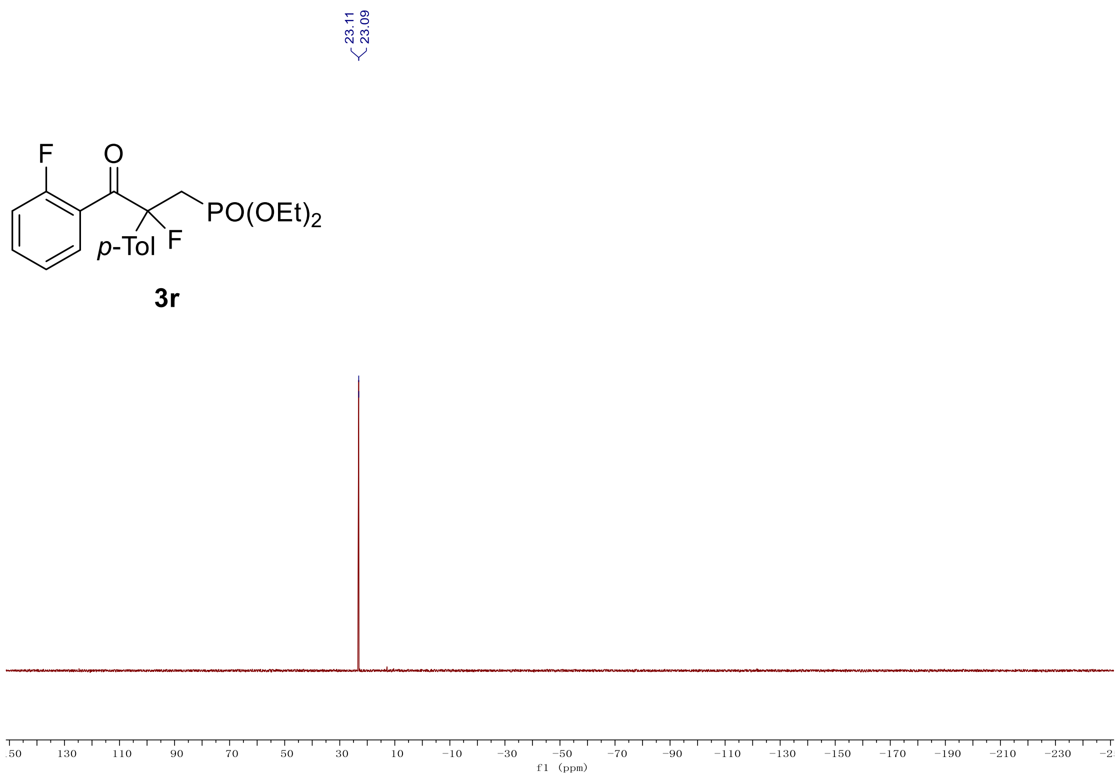

Supplementary Figure 122. ^{31}P NMR (202 MHz, $CDCl_3$) spectrum for compound **3r**

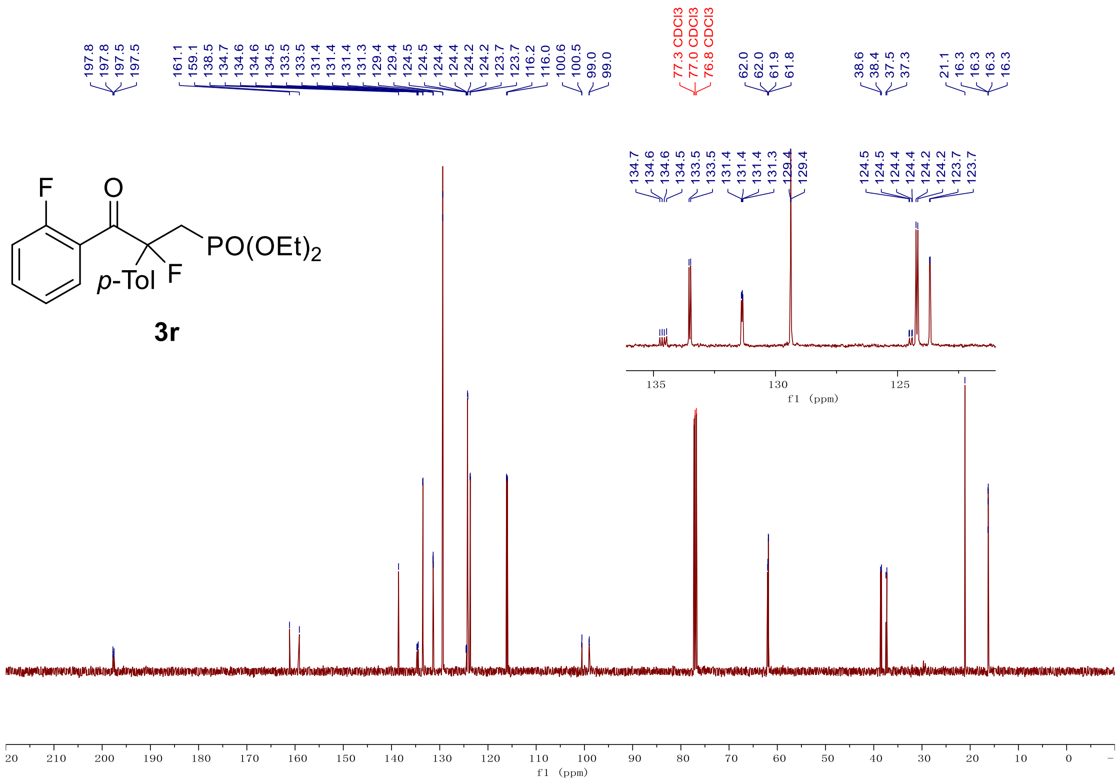

Supplementary Figure 123. ^{13}C NMR (125 MHz, $CDCl_3$) spectrum for compound **3r**

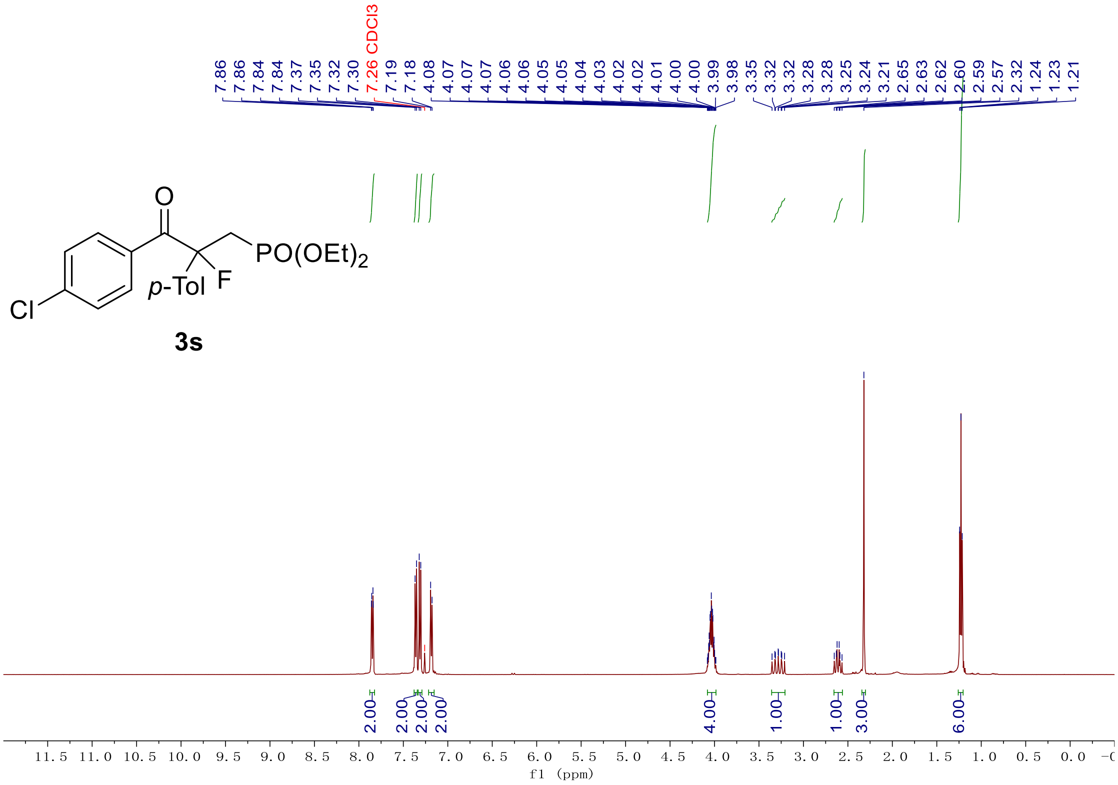

Supplementary Figure 124. ^{1}H NMR (500 MHz, $CDCl_3$) spectrum for compound **3s**

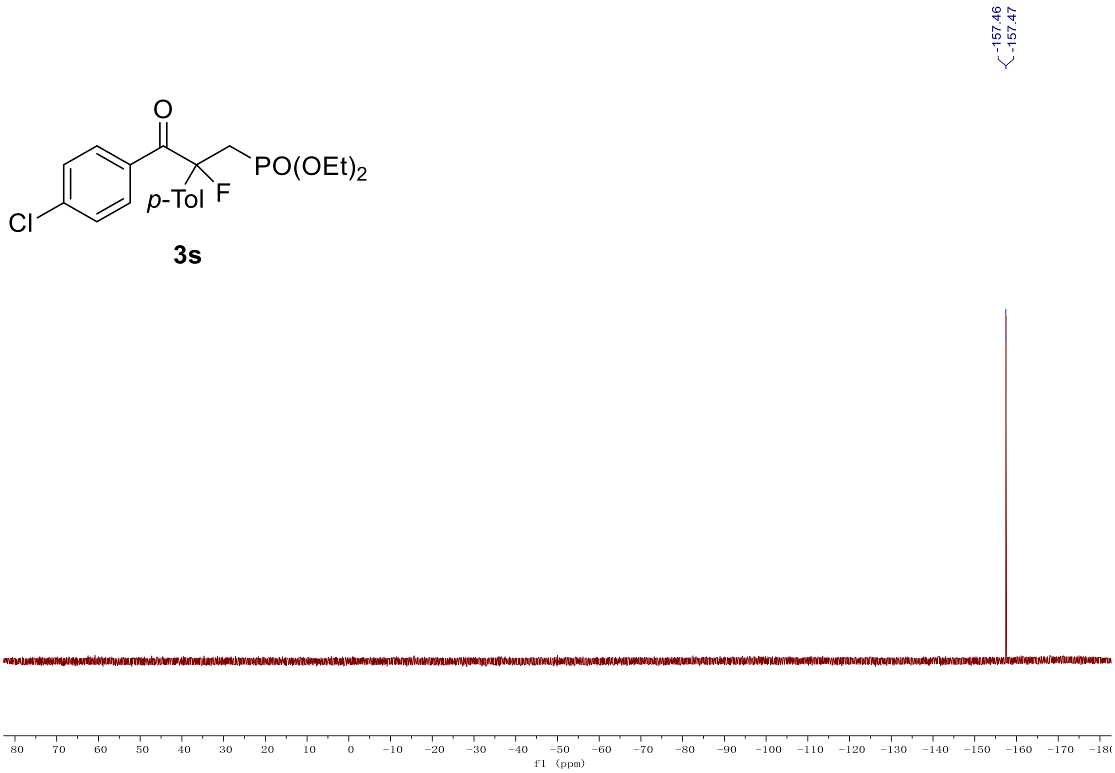

Supplementary Figure 125. ^{19}F NMR (471 MHz, $CDCl_3$) spectrum for compound **3s**

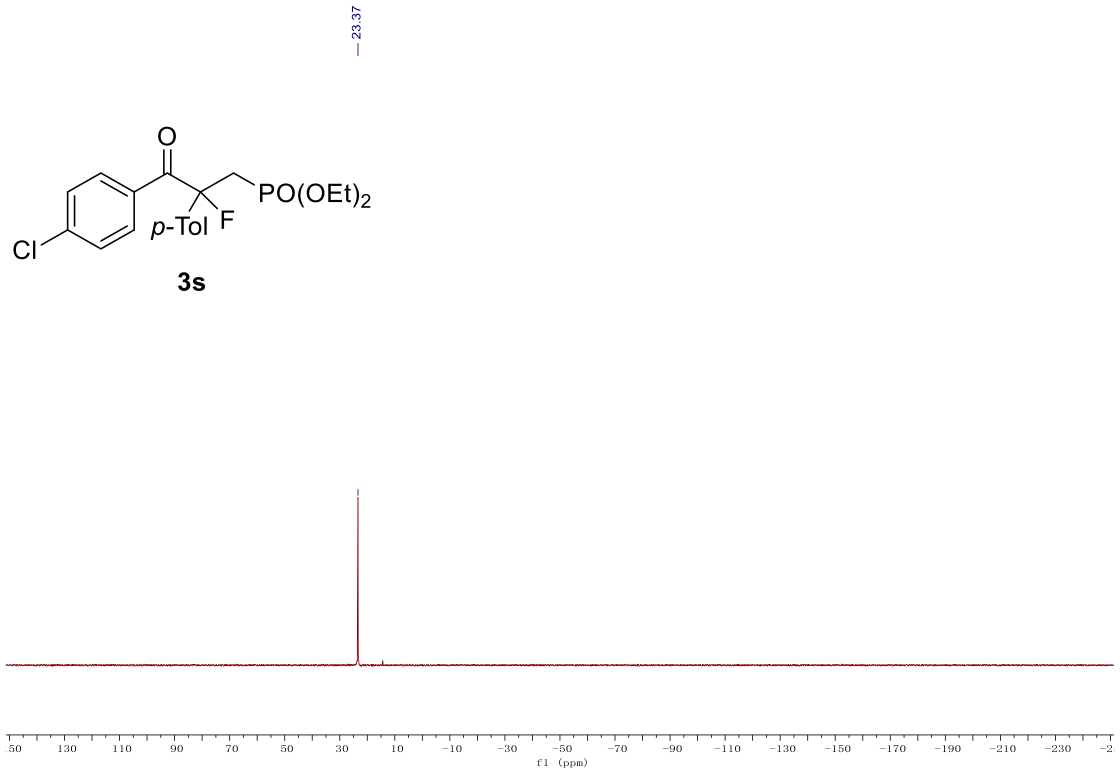

Supplementary Figure 126. ^{31}P NMR (202 MHz, $CDCl_3$) spectrum for compound **3s**

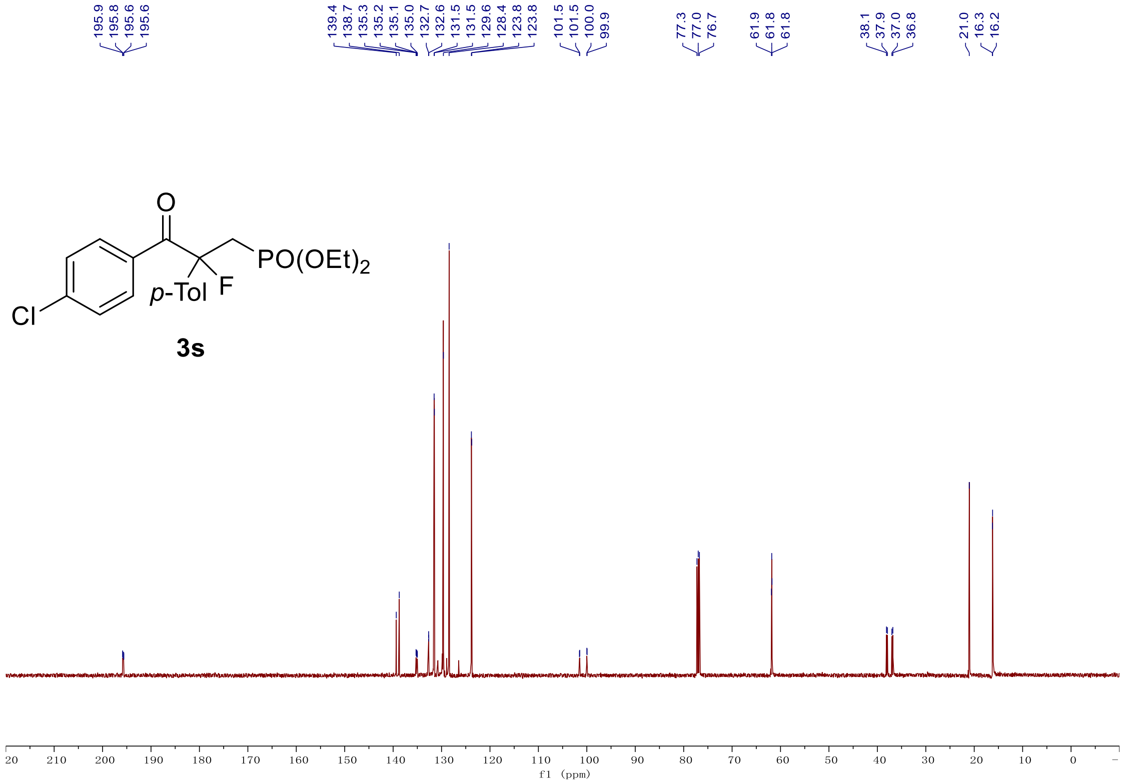

Supplementary Figure 127. ^{13}C NMR (125 MHz, $CDCl_3$) spectrum for compound **3s**

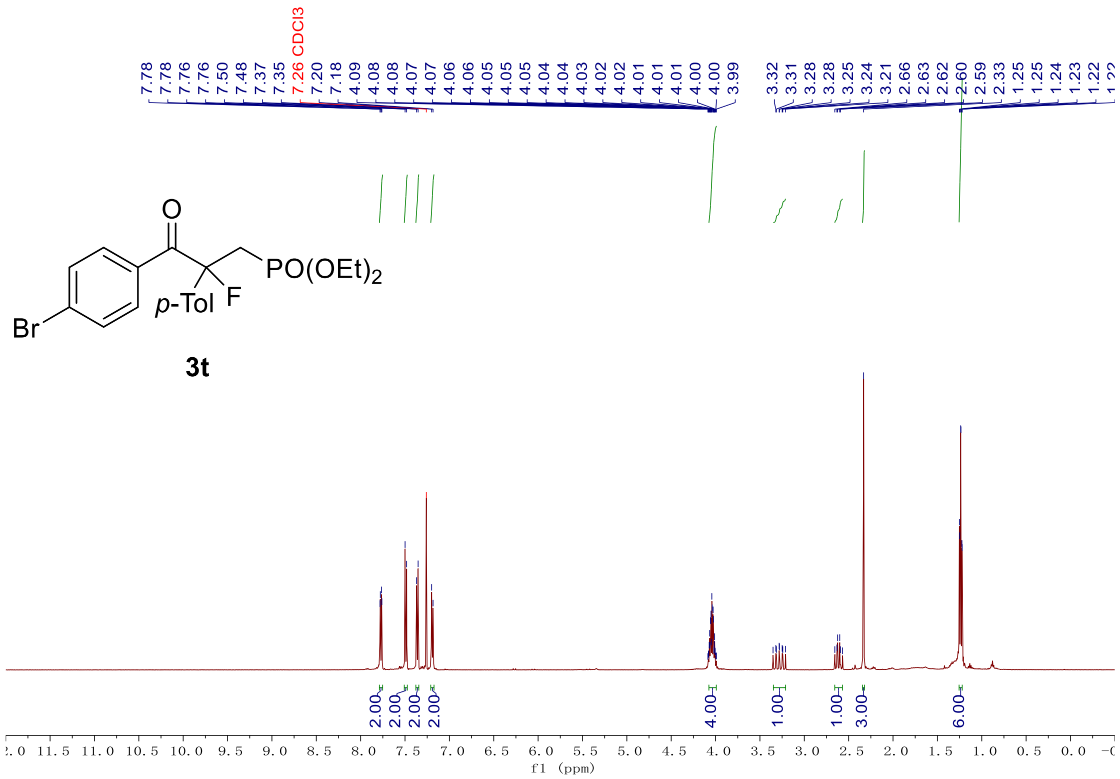

Supplementary Figure 128. ^{1}H NMR (500 MHz, $CDCl_3$) spectrum for compound **3t**

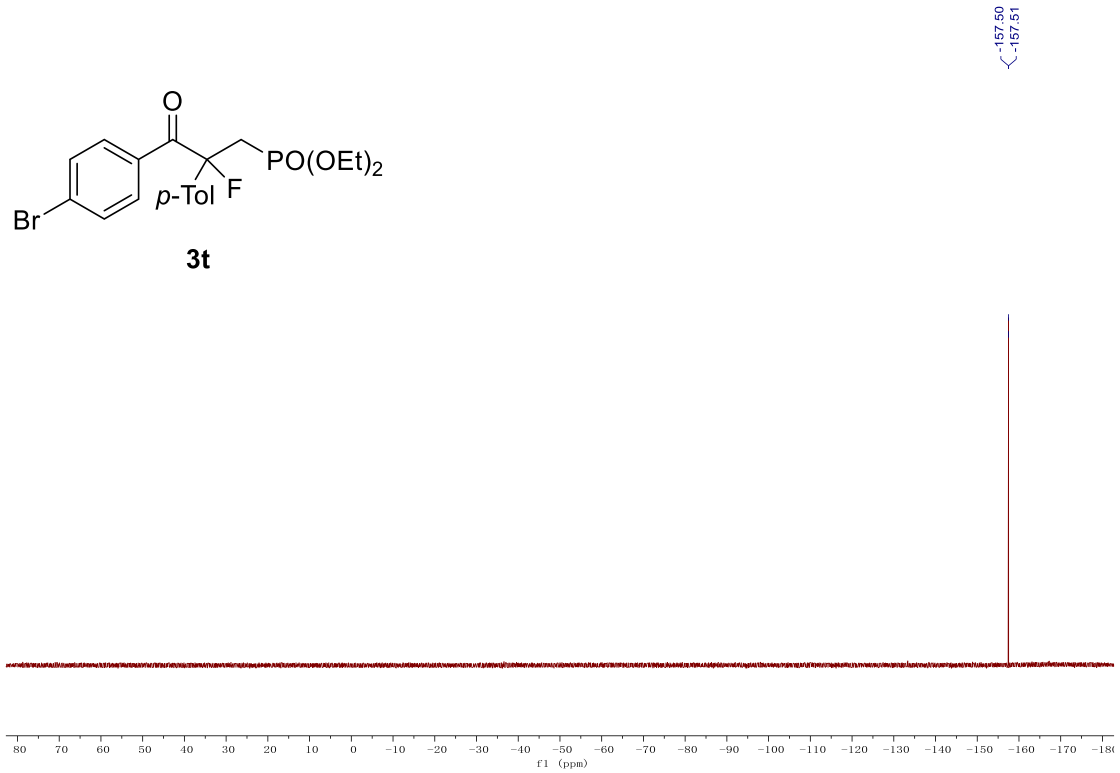

Supplementary Figure 129. ^{19}F NMR (471 MHz, $CDCl_3$) spectrum for compound **3t**

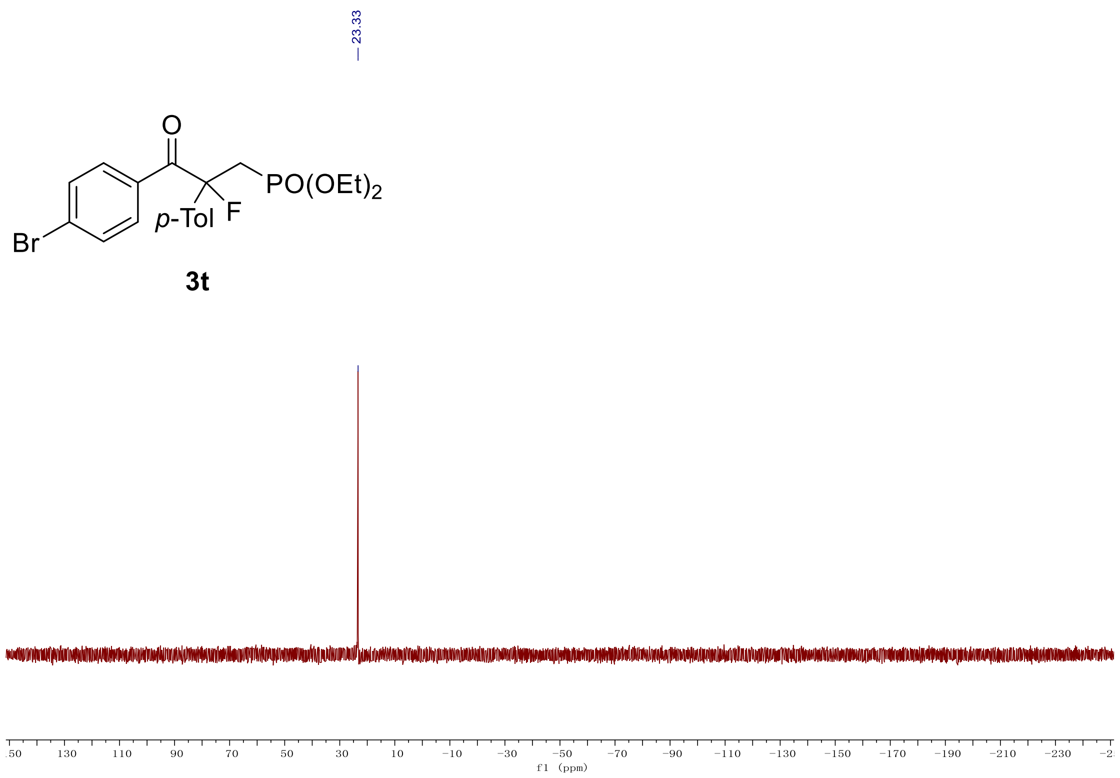

Supplementary Figure 130. ^{31}P NMR (202 MHz, CDCl3) spectrum for compound **3t**

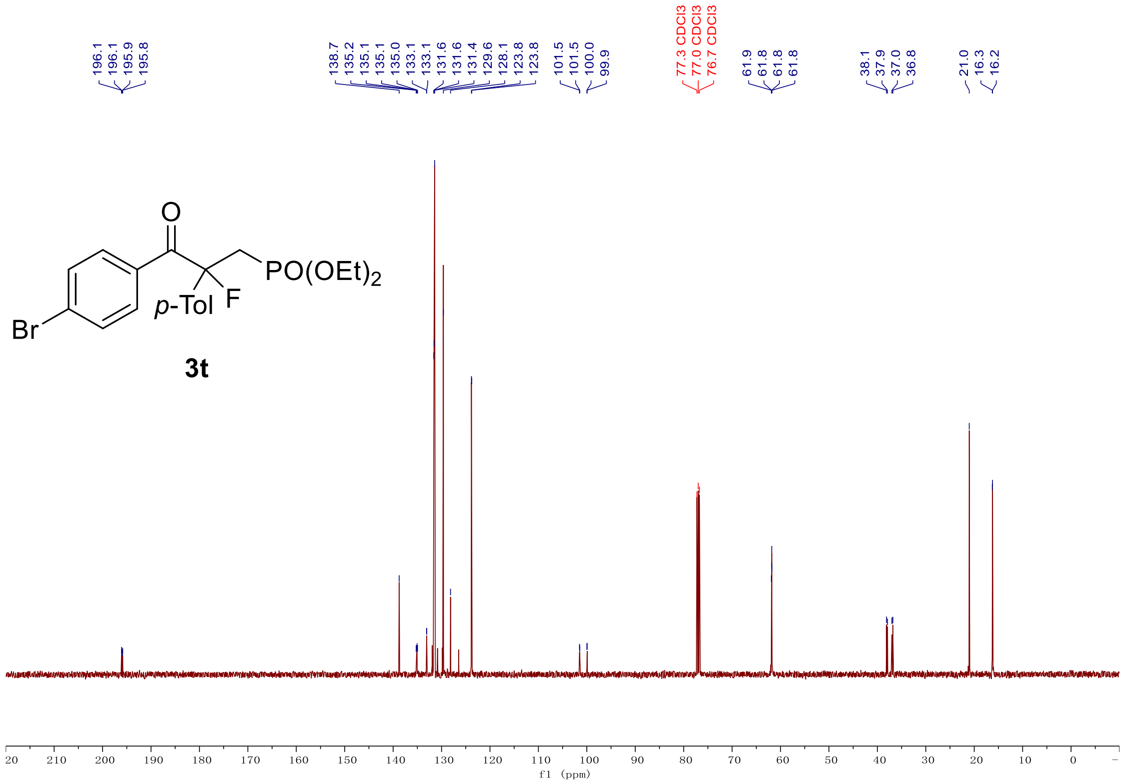

Supplementary Figure 131. ^{13}C NMR (125 MHz, CDCl3) spectrum for compound **3t**

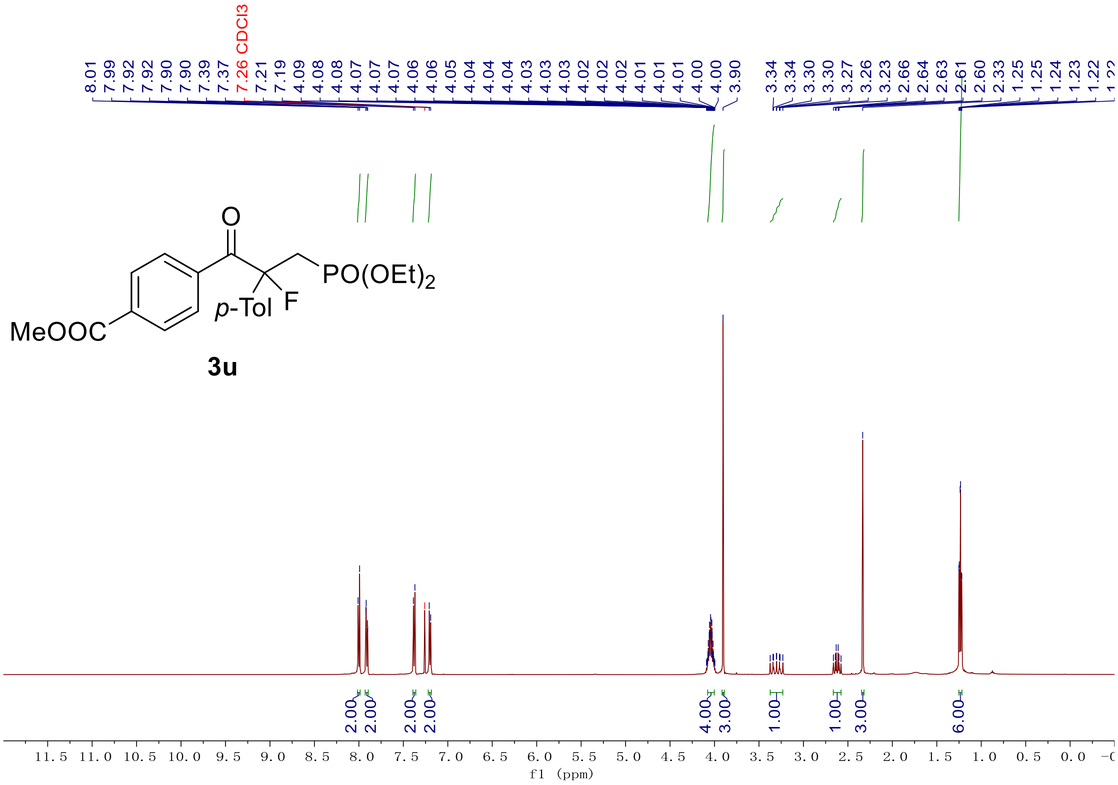

Supplementary Figure 132. ^{1}H NMR (500 MHz, $CDCl_3$) spectrum for compound **3u**

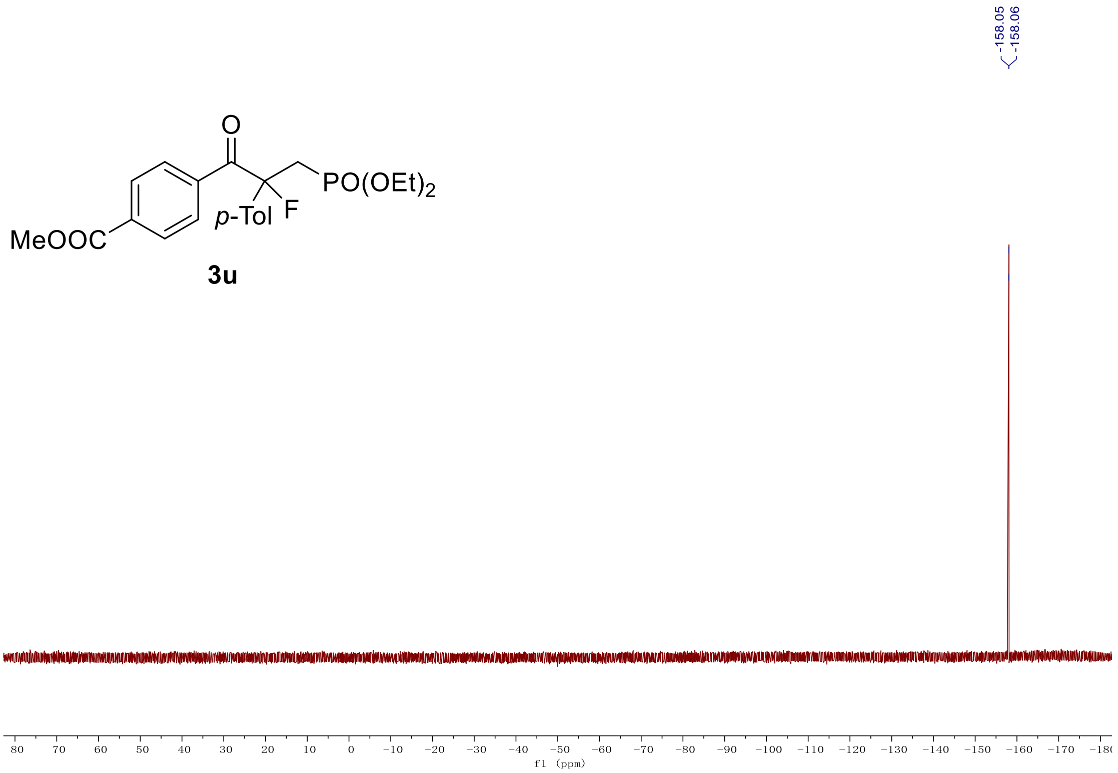

Supplementary Figure 133. ^{19}F NMR (471 MHz, $CDCl_3$) spectrum for compound **3u**

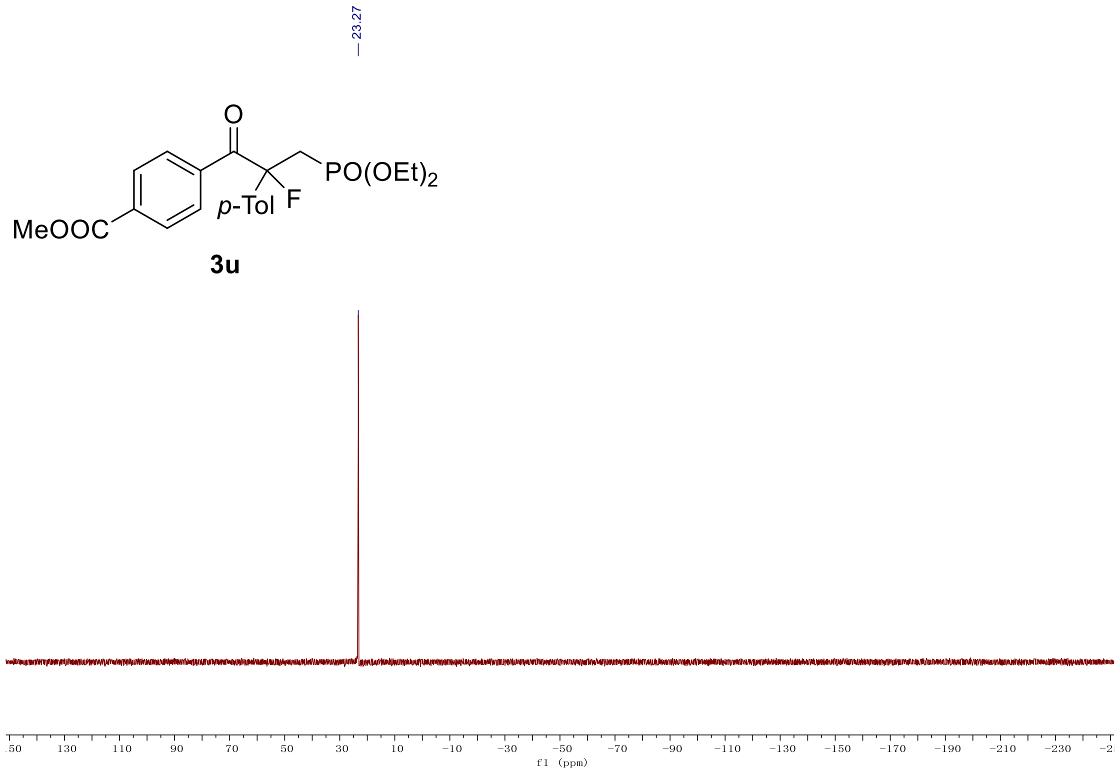

Supplementary Figure 134. ^{31}P NMR (202 MHz, $CDCl_3$) spectrum for compound **3u**

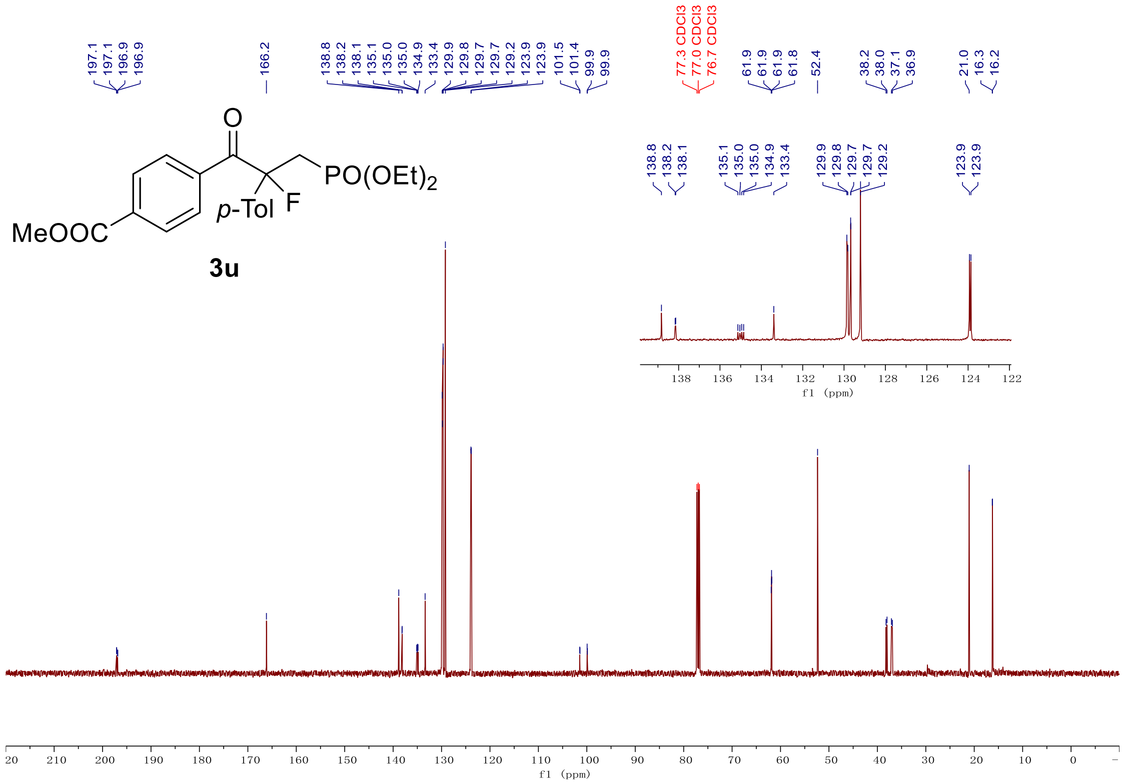

Supplementary Figure 135. ^{13}C NMR (125 MHz, $CDCl_3$) spectrum for compound **3u**

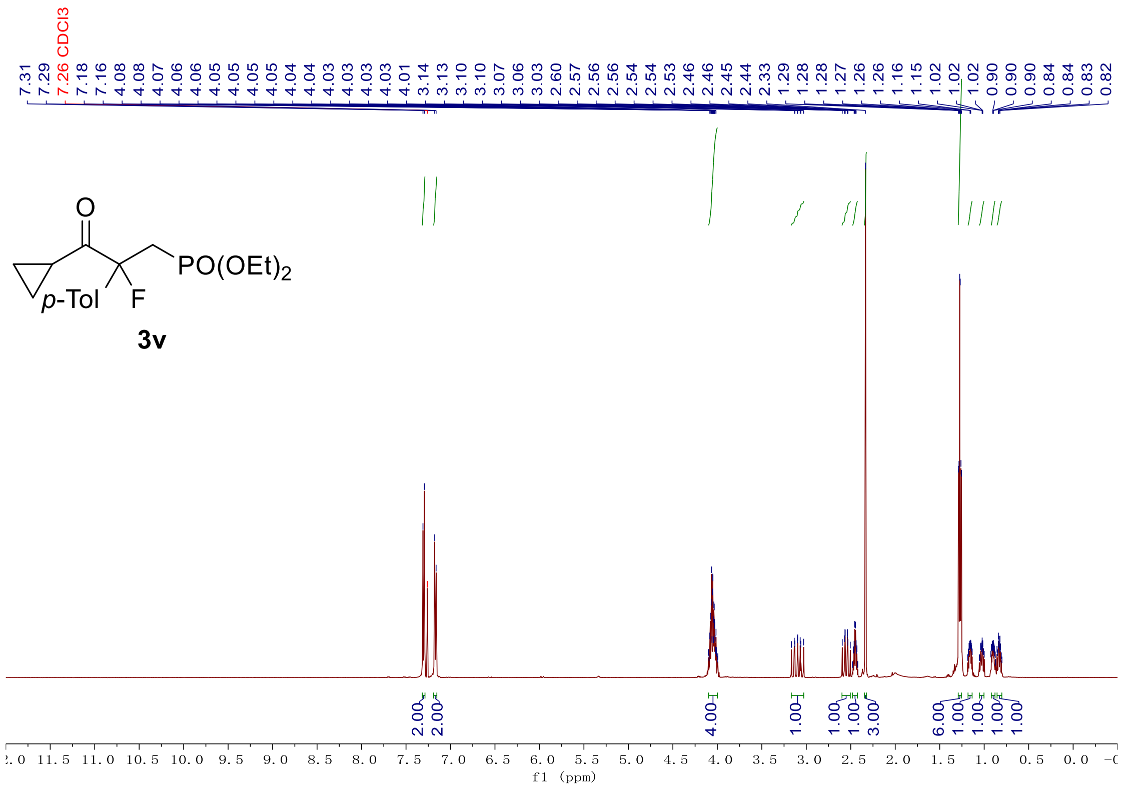

Supplementary Figure 136. ^{1}H NMR (500 MHz, $CDCl_3$) spectrum for compound **3v**

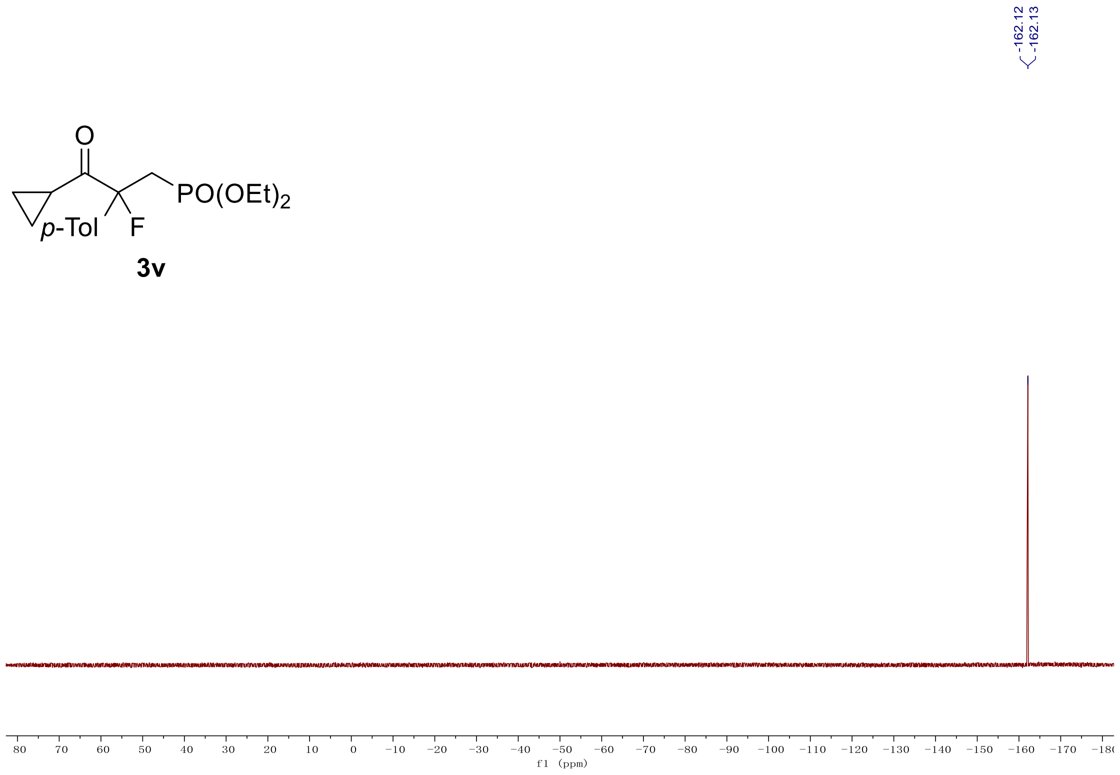

Supplementary Figure 137. ^{19}F NMR (471 MHz, $CDCl_3$) spectrum for compound **3v**

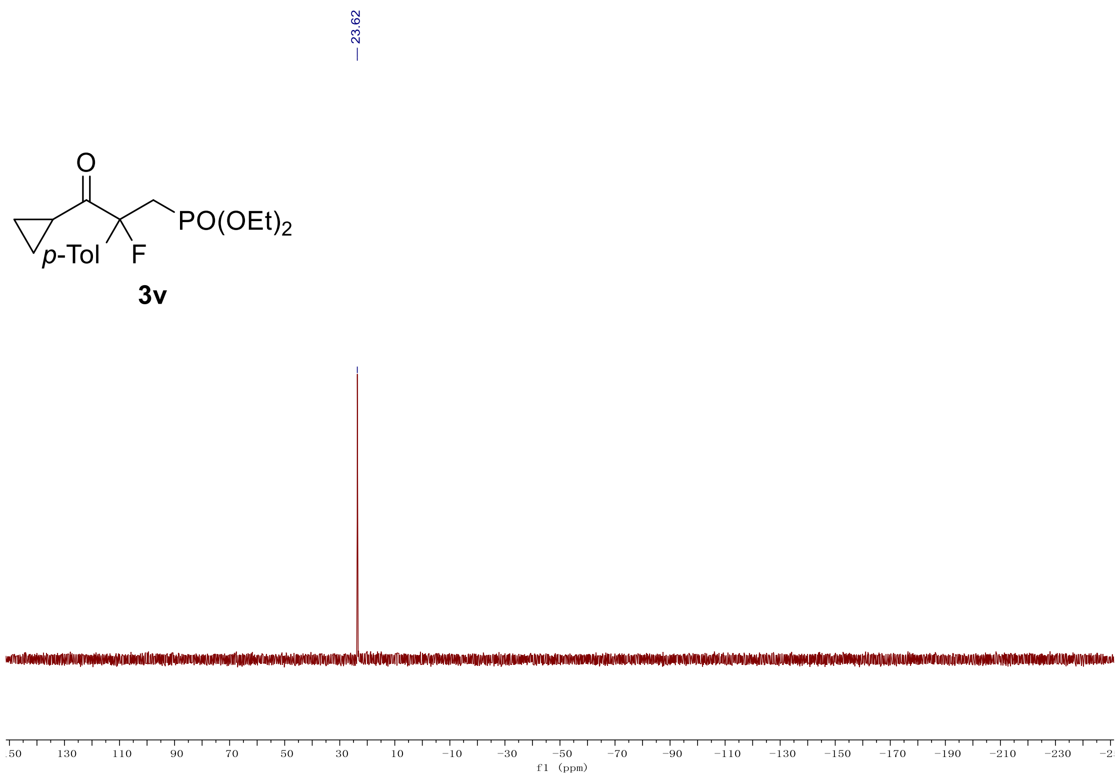

Supplementary Figure 138. ^{31}P NMR (202 MHz, CDCl3) spectrum for compound **3v**

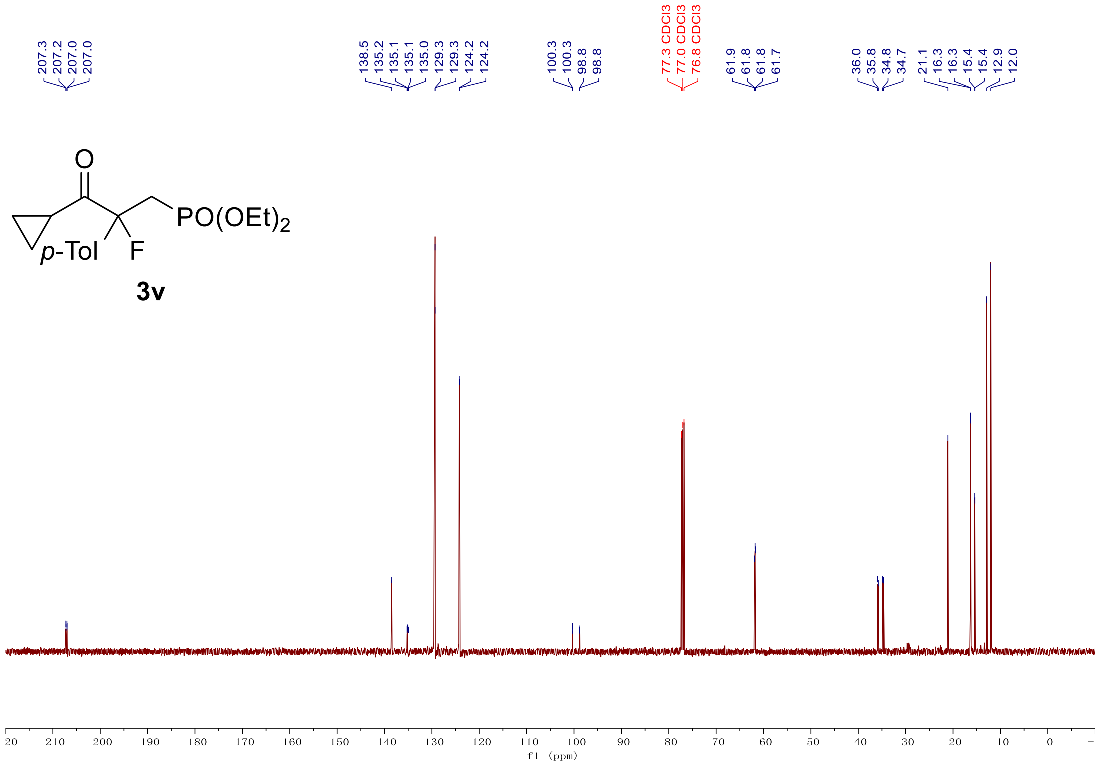

Supplementary Figure 139. ^{13}C NMR (125 MHz, CDCl3) spectrum for compound **3v**

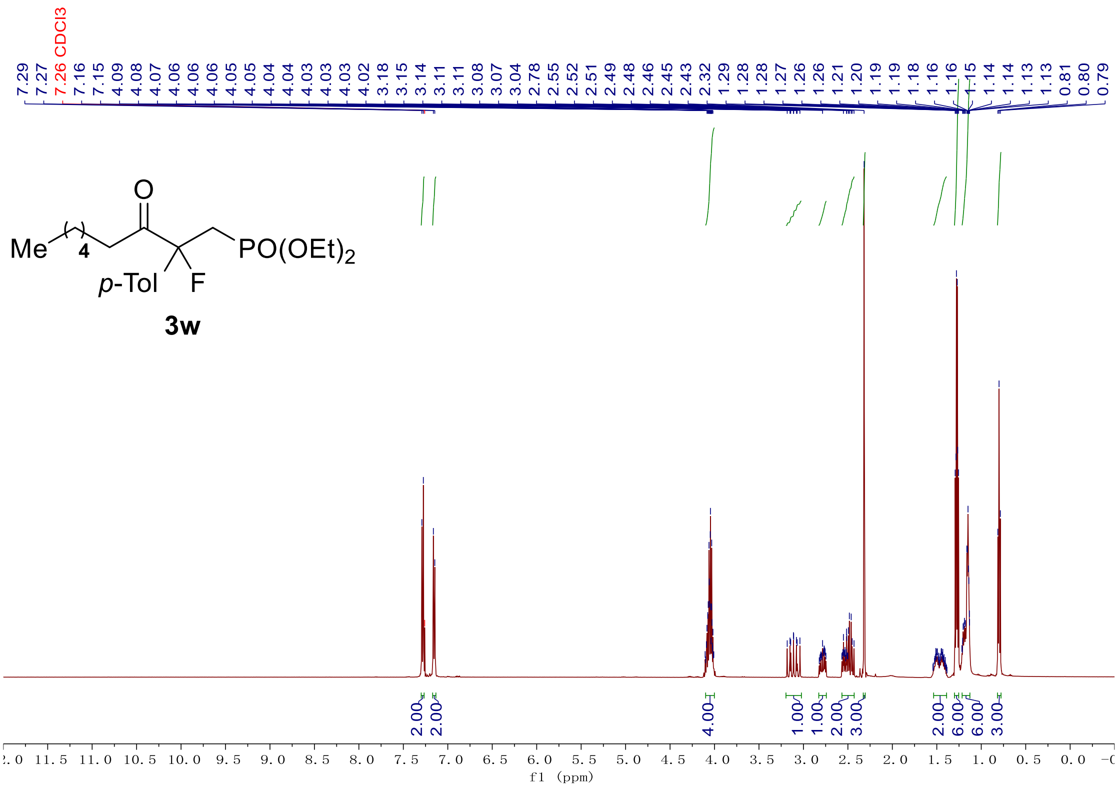

Supplementary Figure 140. ^{1}H NMR (500 MHz, $CDCl_3$) spectrum for compound **3w**

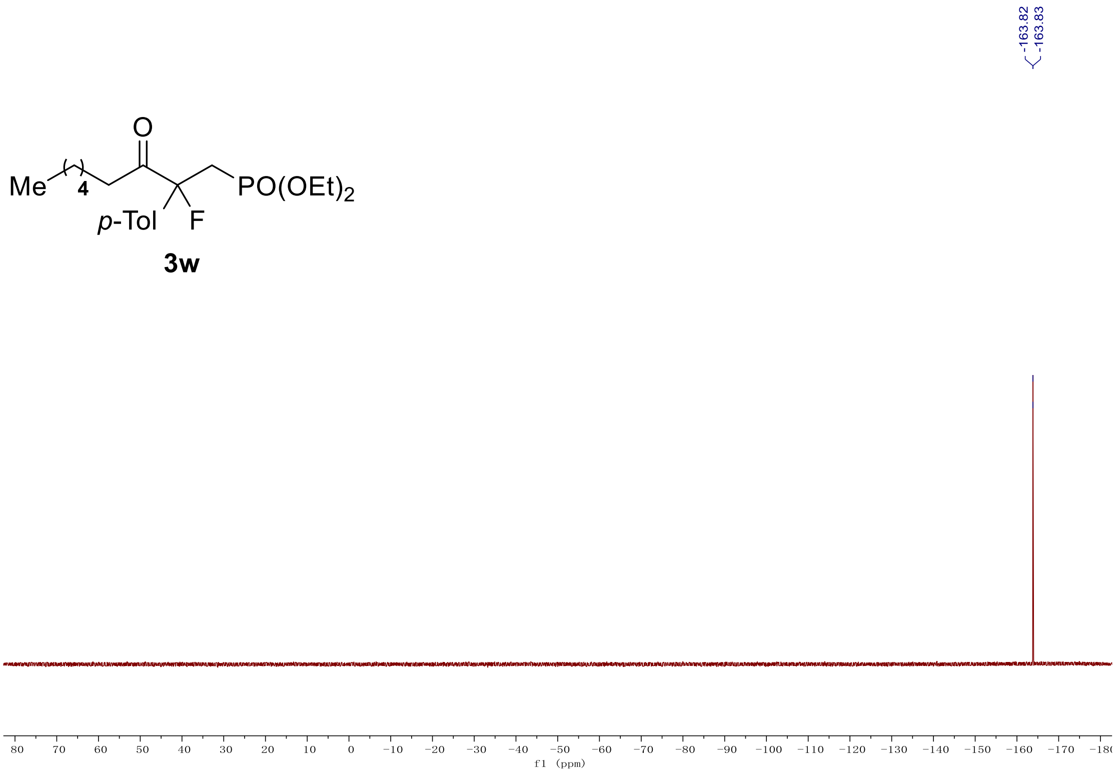

Supplementary Figure 141. ^{19}F NMR (471 MHz, $CDCl_3$) spectrum for compound **3w**

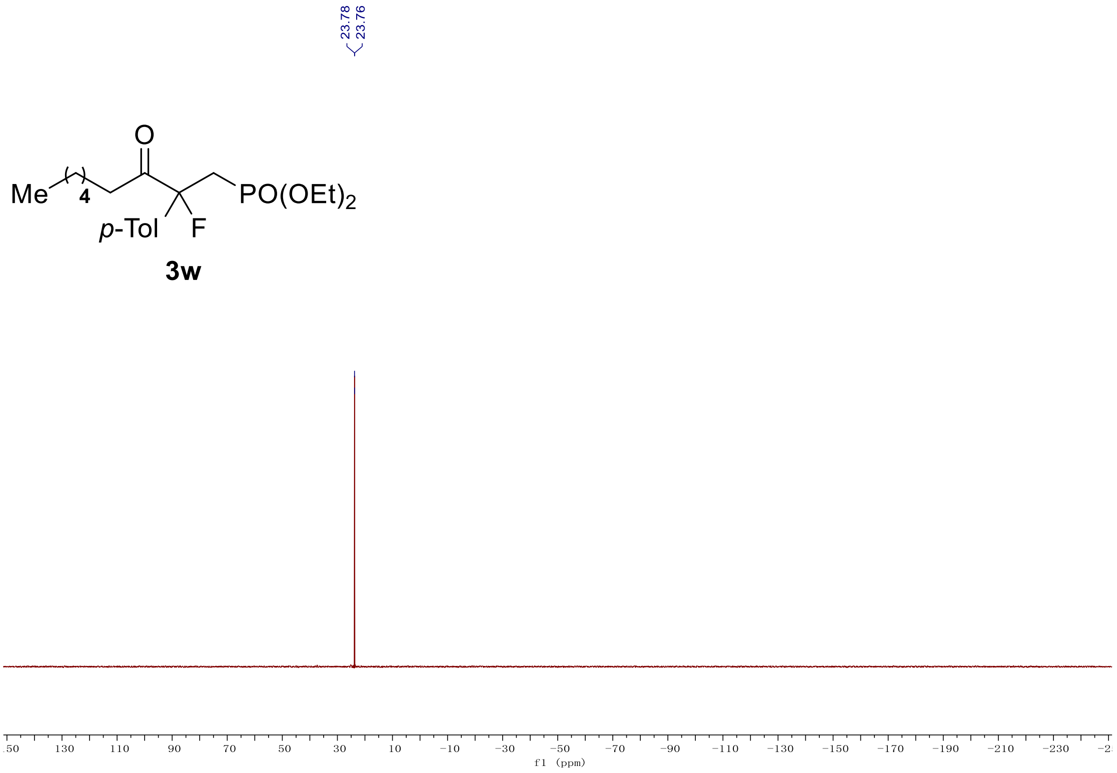

Supplementary Figure 142. ^{31}P NMR (202 MHz, $CDCl_3$) spectrum for compound **3w**

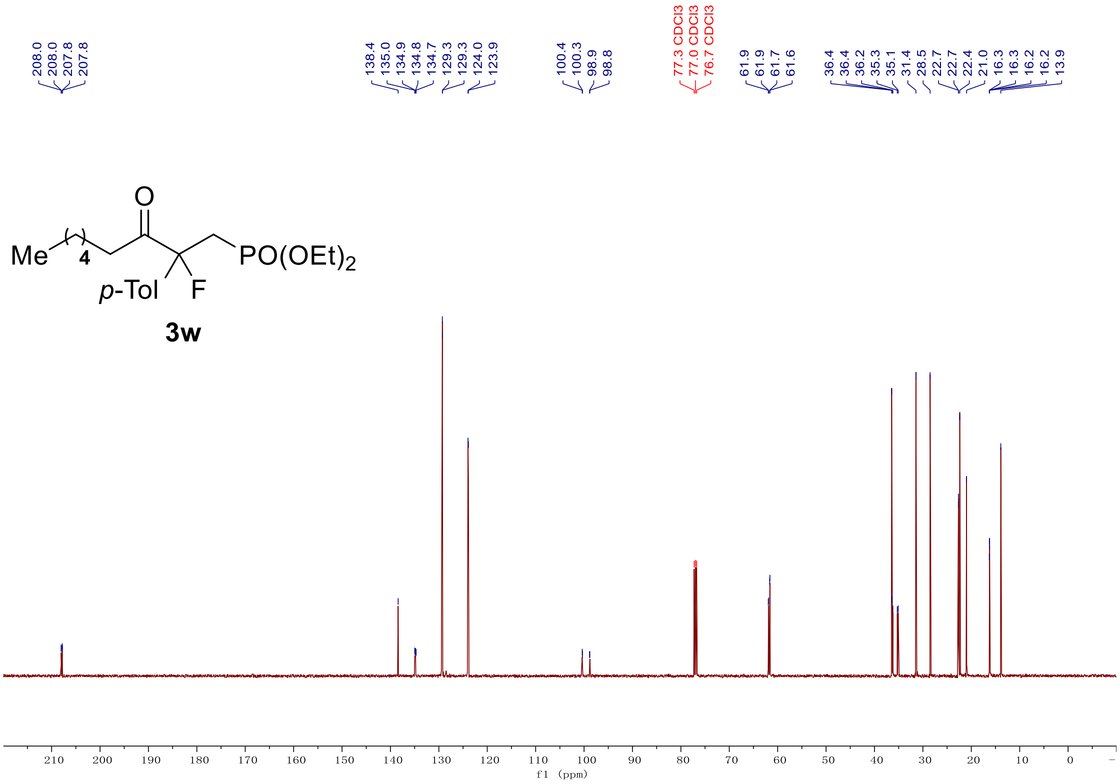

Supplementary Figure 143. ^{13}C NMR (125 MHz, $CDCl_3$) spectrum for compound **3w**

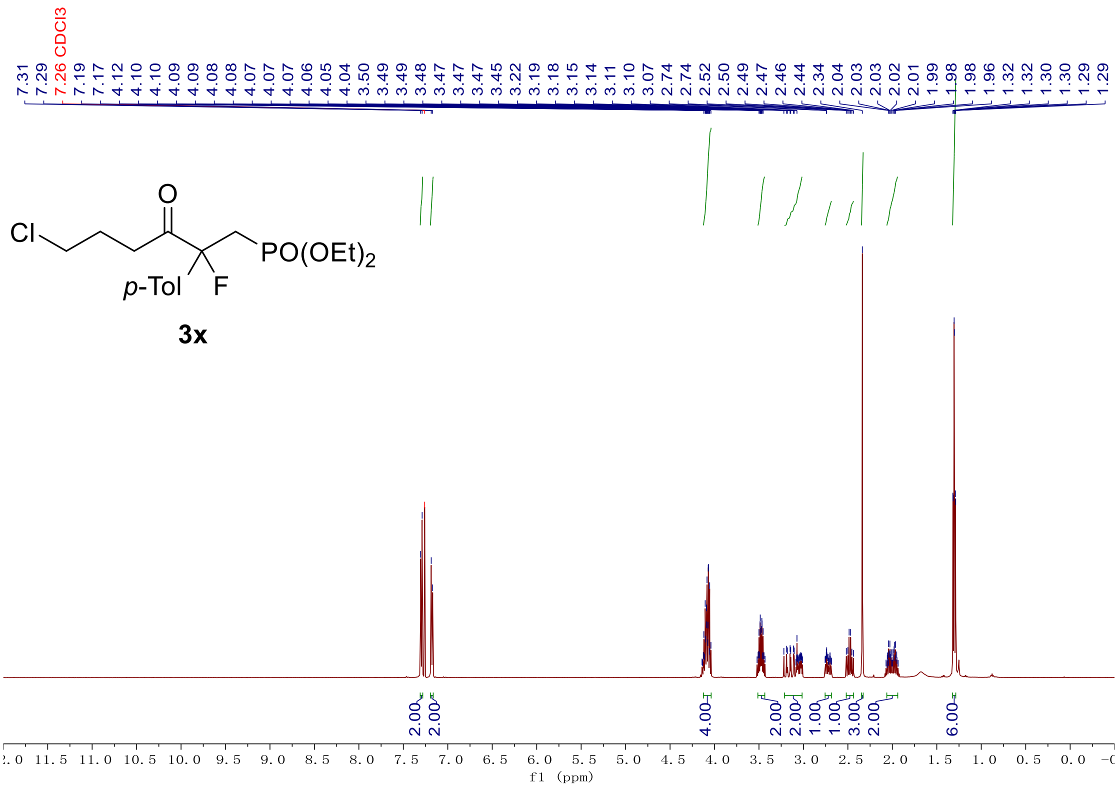

Supplementary Figure 144. ^{1}H NMR (500 MHz, $CDCl_3$) spectrum for compound **3x**

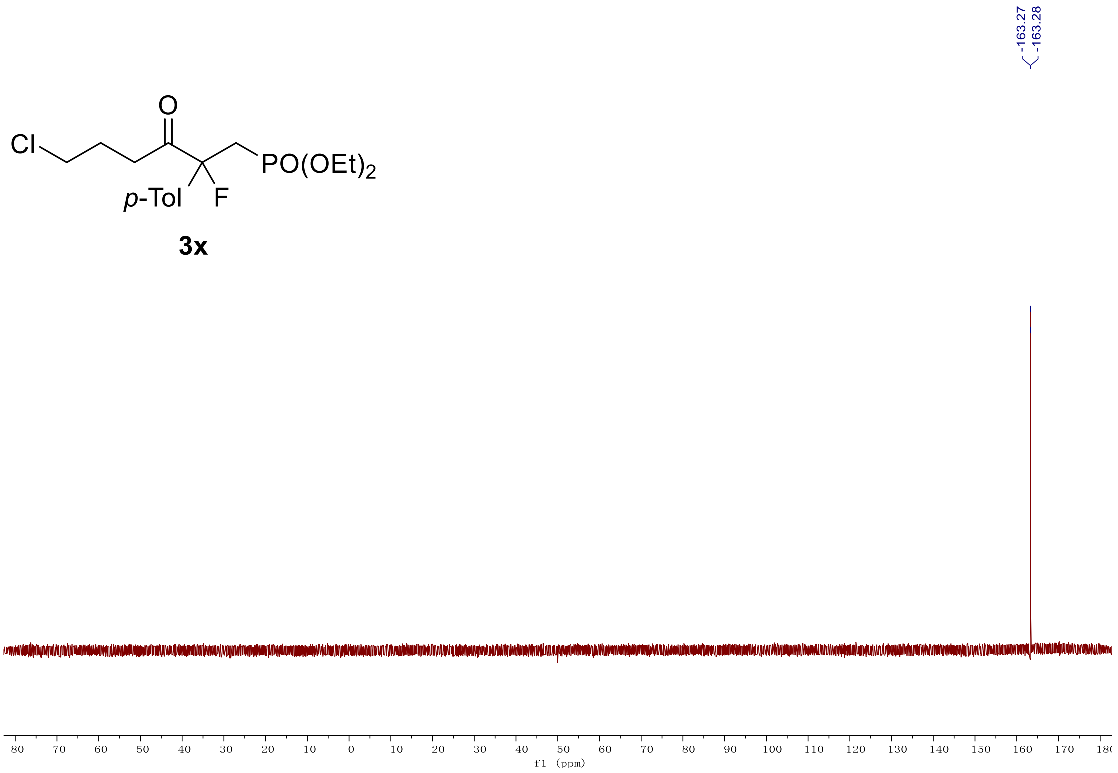

Supplementary Figure 145. ^{19}F NMR (471 MHz, $CDCl_3$) spectrum for compound **3x**

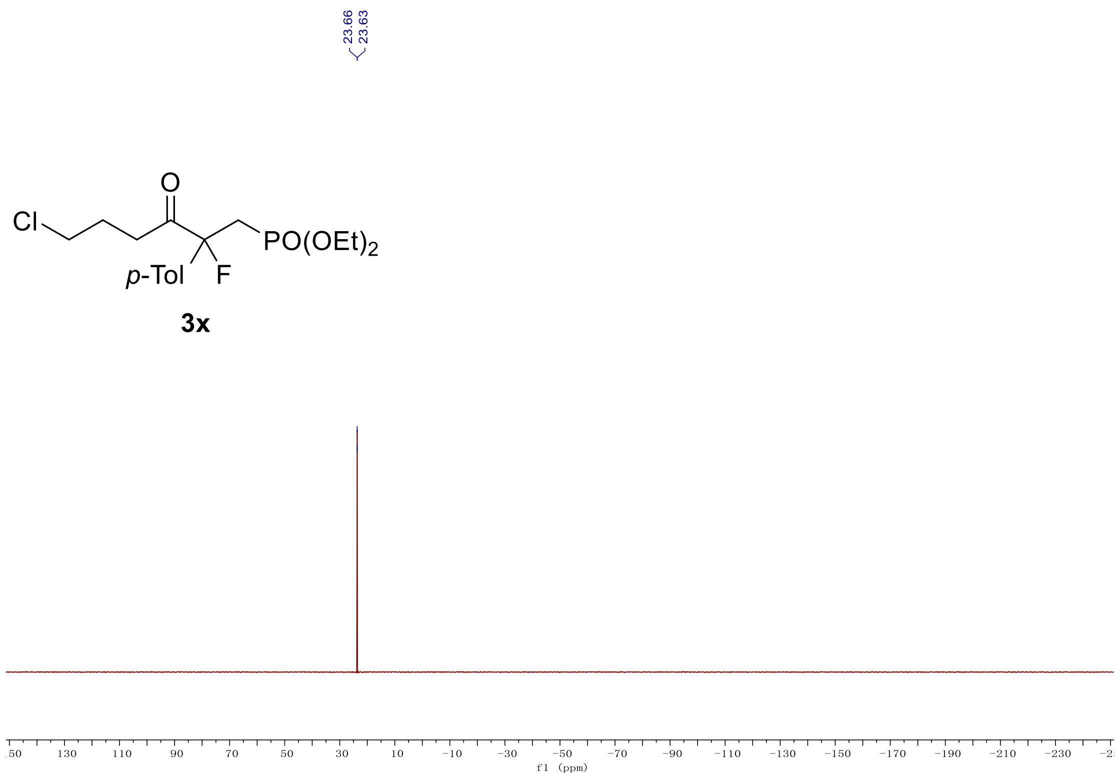

Supplementary Figure 146. ^{31}P NMR (202 MHz, CDCl₃) spectrum for compound **3x**

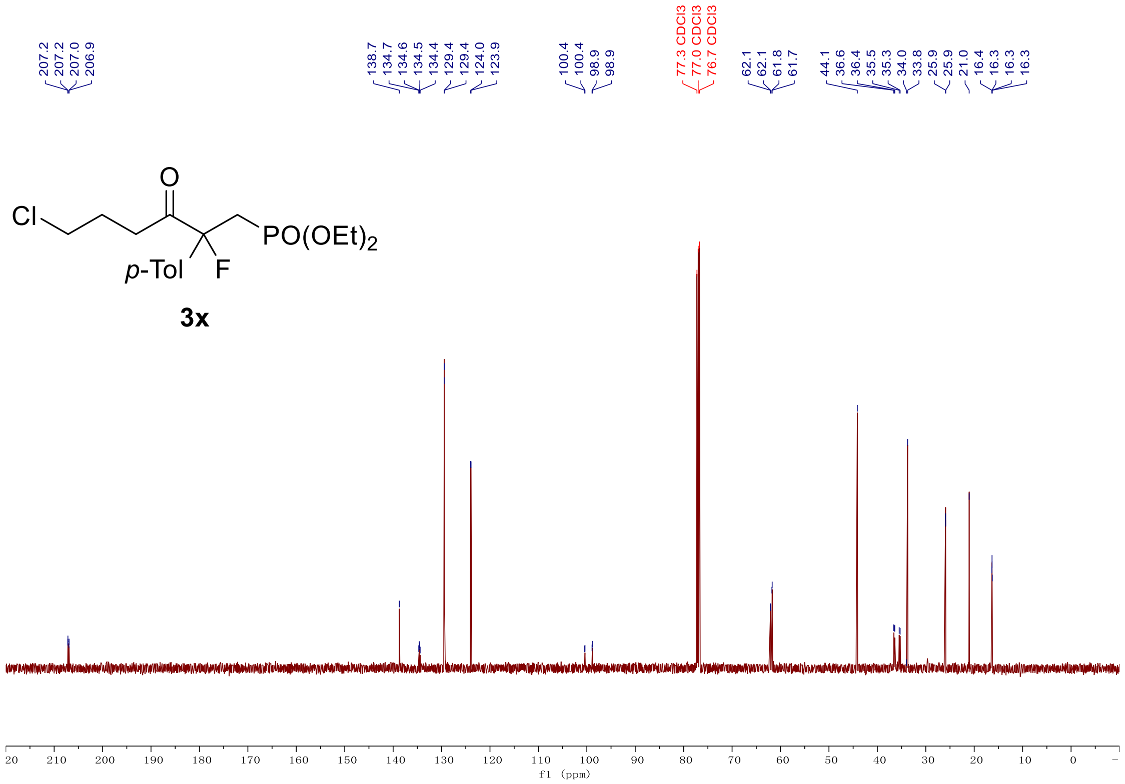

Supplementary Figure 147. ^{13}C NMR (125 MHz, CDCl₃) spectrum for compound **3x**

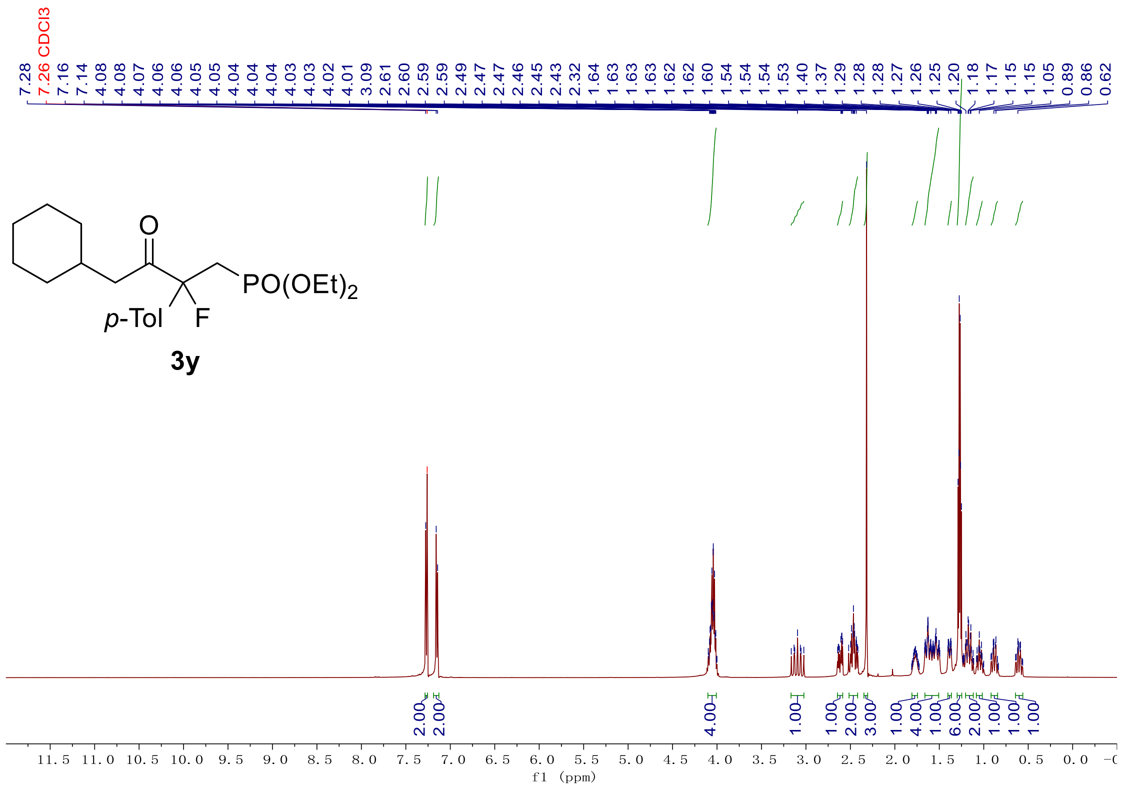

Supplementary Figure 148. ^{1}H NMR (500 MHz, $CDCl_3$) spectrum for compound **3y**

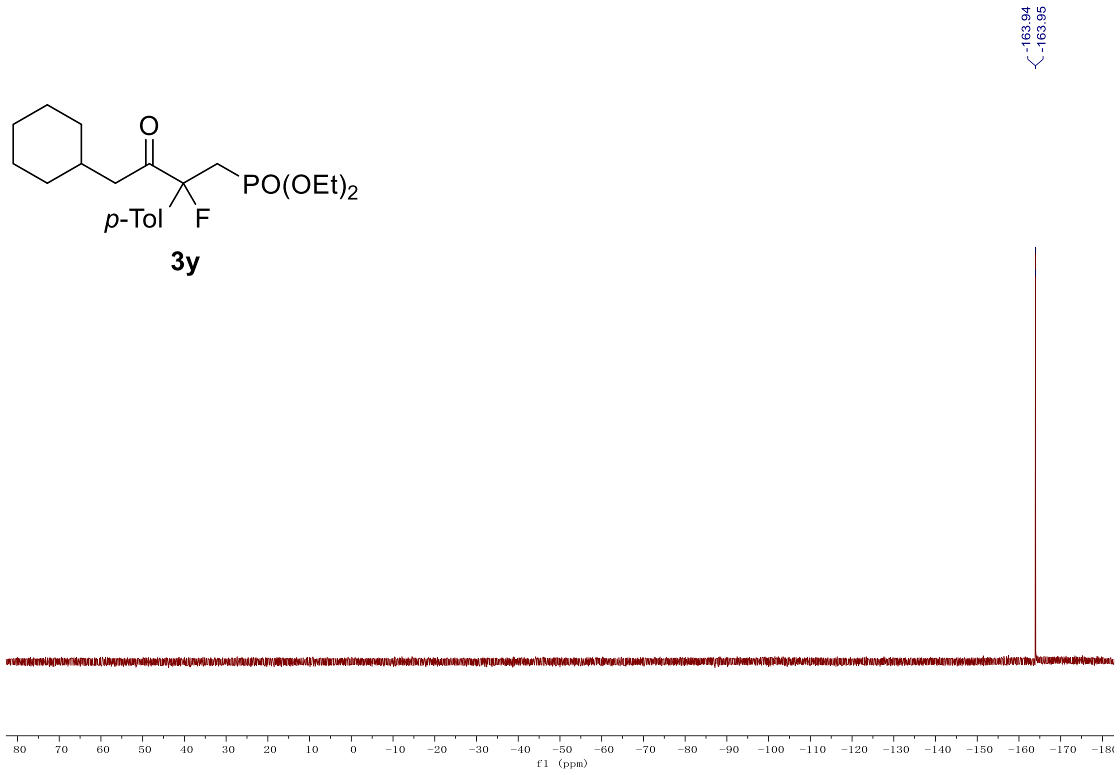

Supplementary Figure 149. ^{19}F NMR (471 MHz, $CDCl_3$) spectrum for compound **3y**

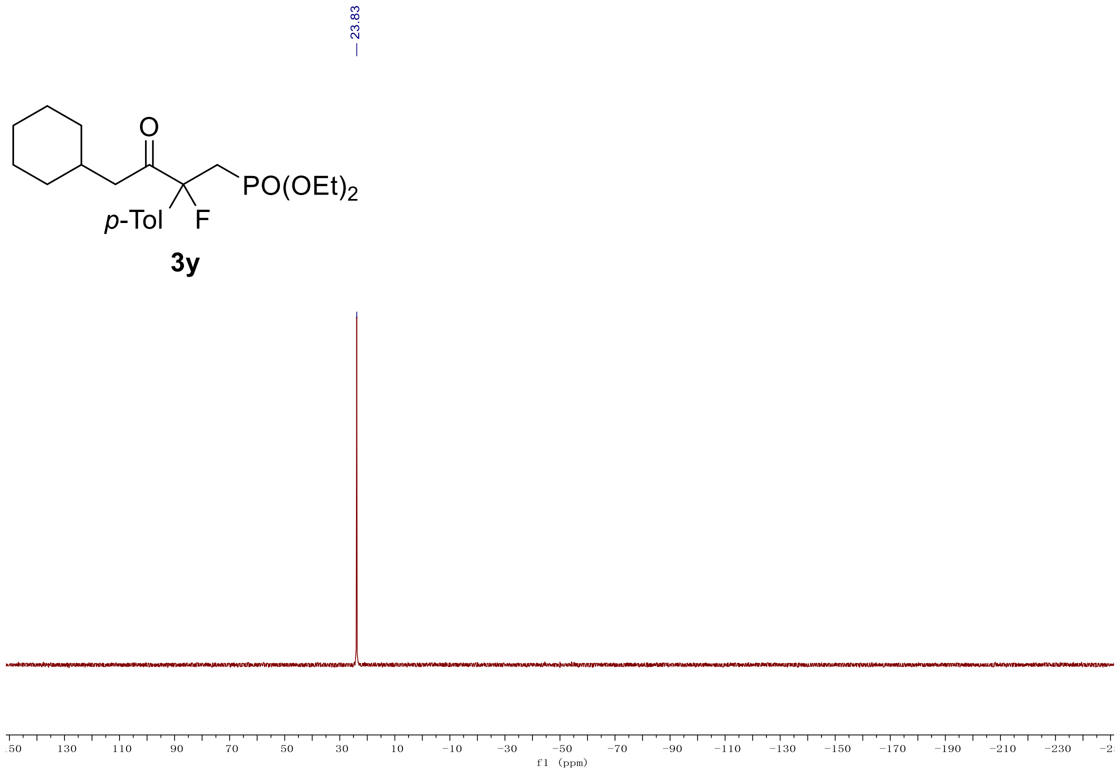

Supplementary Figure 150. ^{31}P NMR (202 MHz, $CDCl_3$) spectrum for compound **3y**

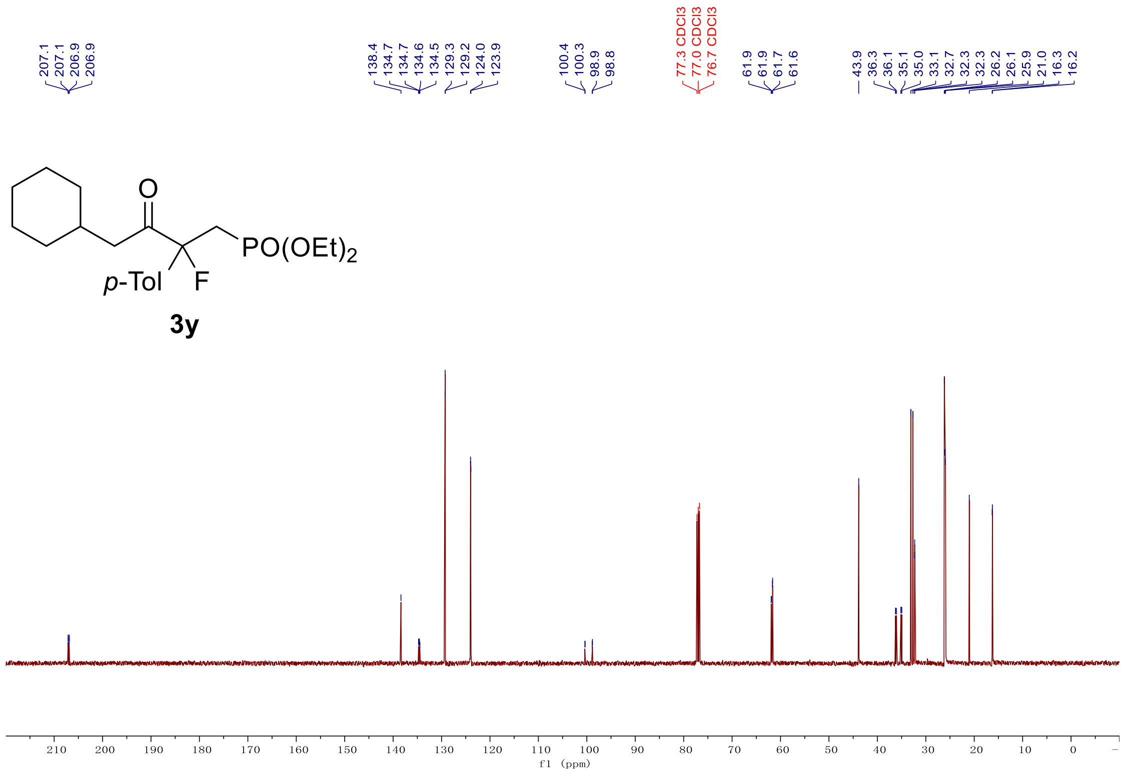

Supplementary Figure 151. ^{13}C NMR (125 MHz, $CDCl_3$) spectrum for compound **3y**

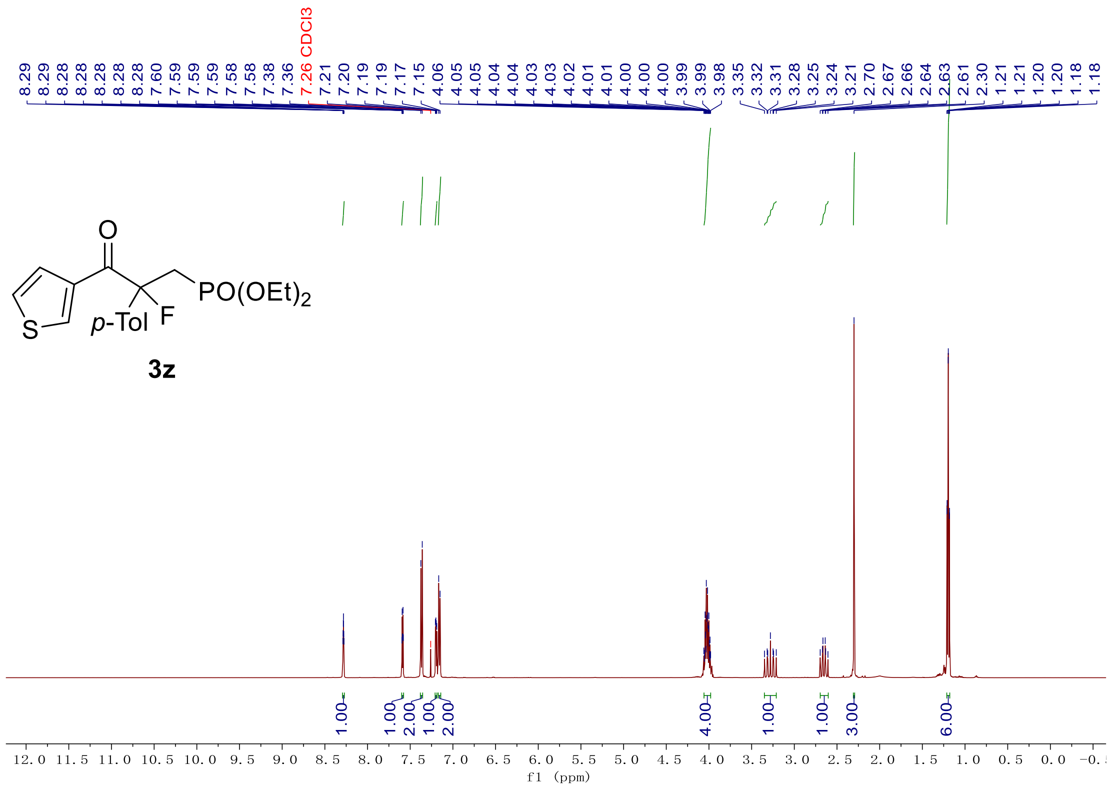

Supplementary Figure 152. ^{1}H NMR (500 MHz, $CDCl_3$) spectrum for compound **3z**

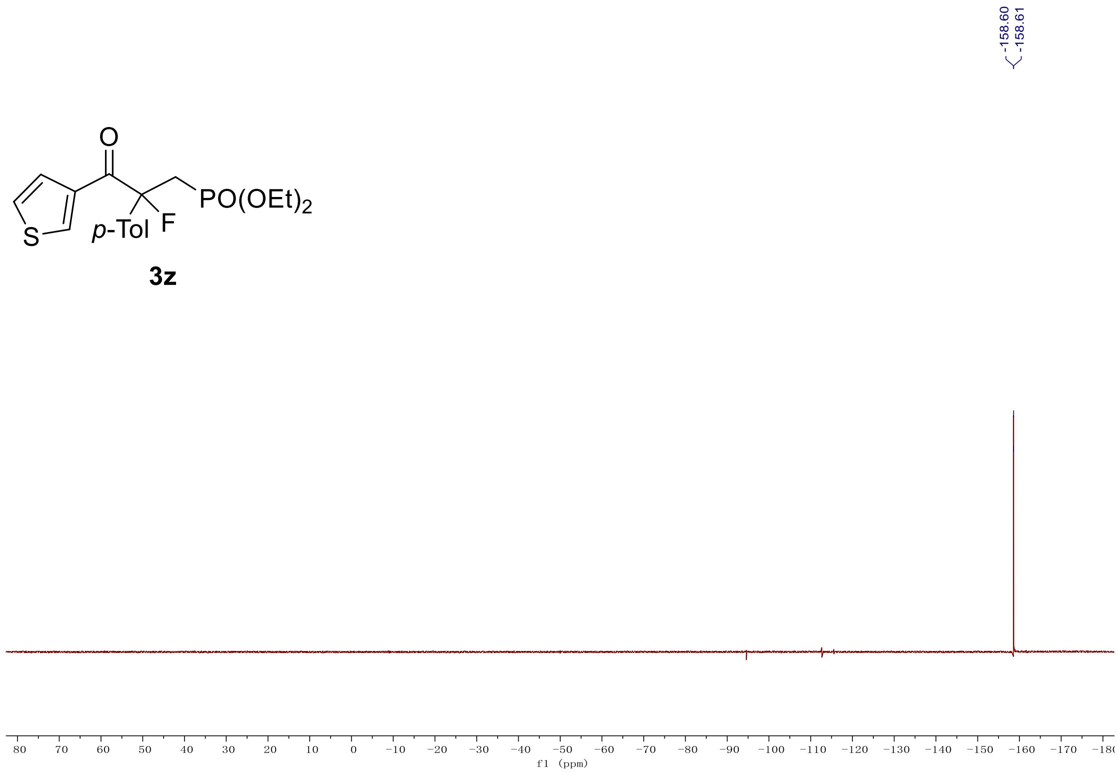

Supplementary Figure 153. ^{19}F NMR (471 MHz, $CDCl_3$) spectrum for compound **3z**

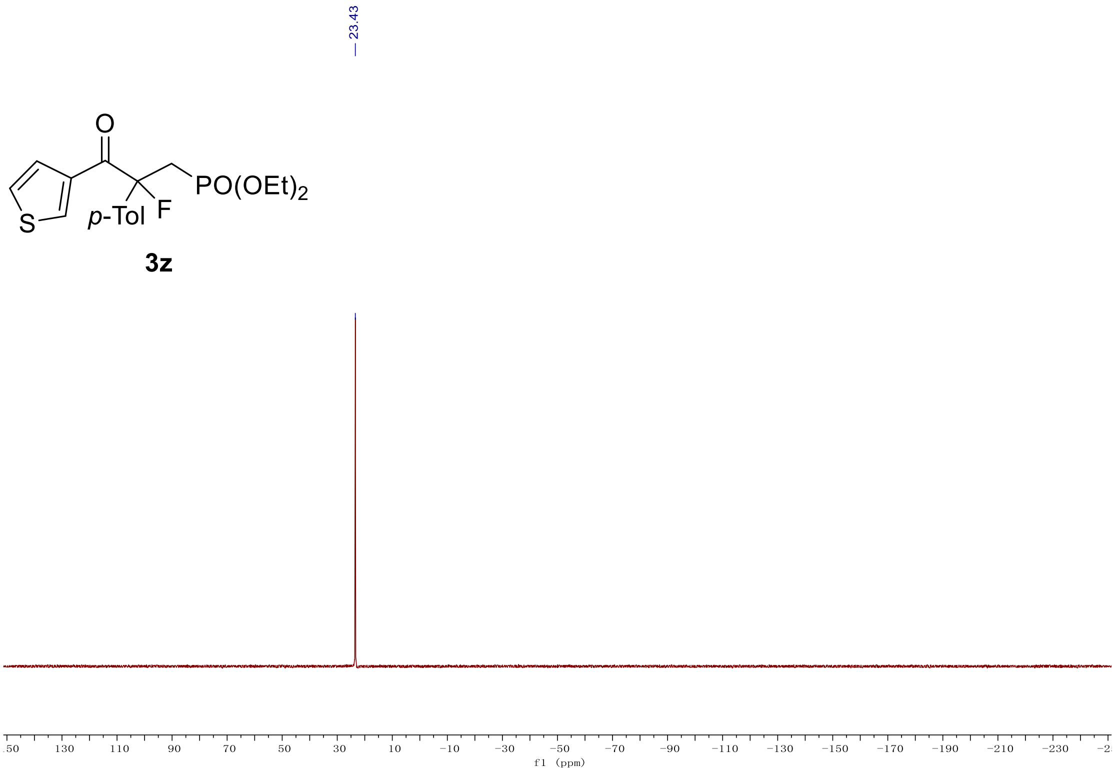

Supplementary Figure 154. ^{31}P NMR (202 MHz, CDCl₃) spectrum for compound **3z**

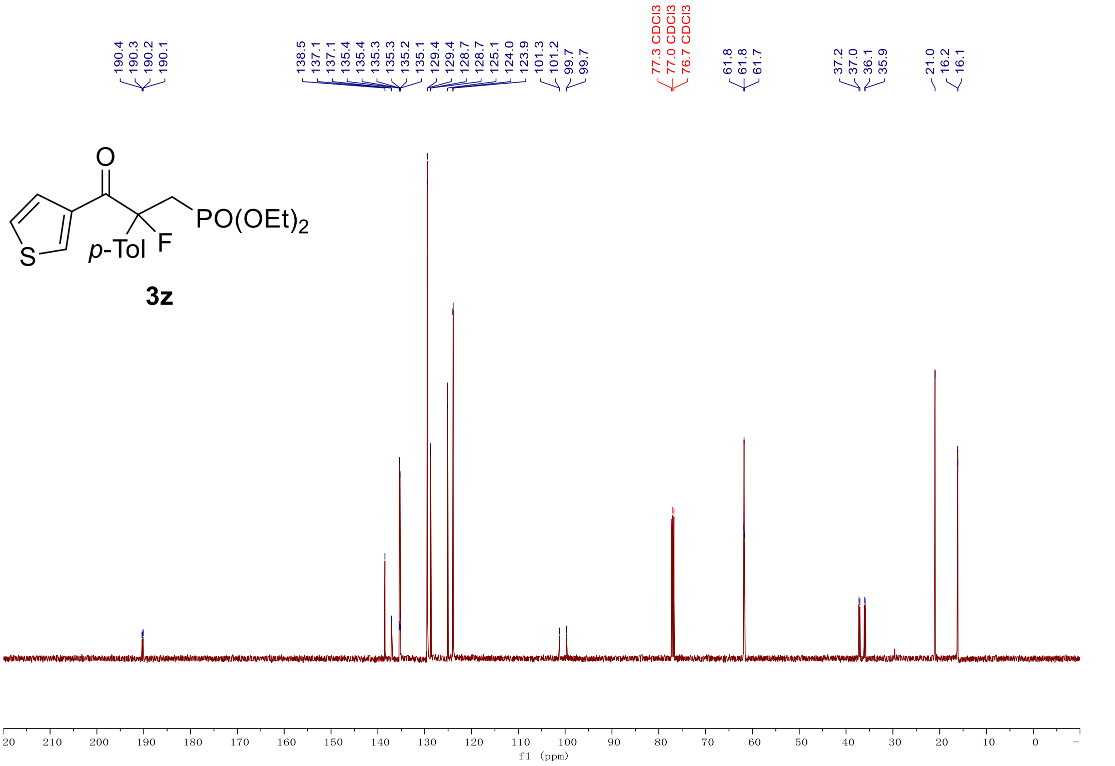

Supplementary Figure 155. ^{13}C NMR (125 MHz, CDCl₃) spectrum for compound **3z**

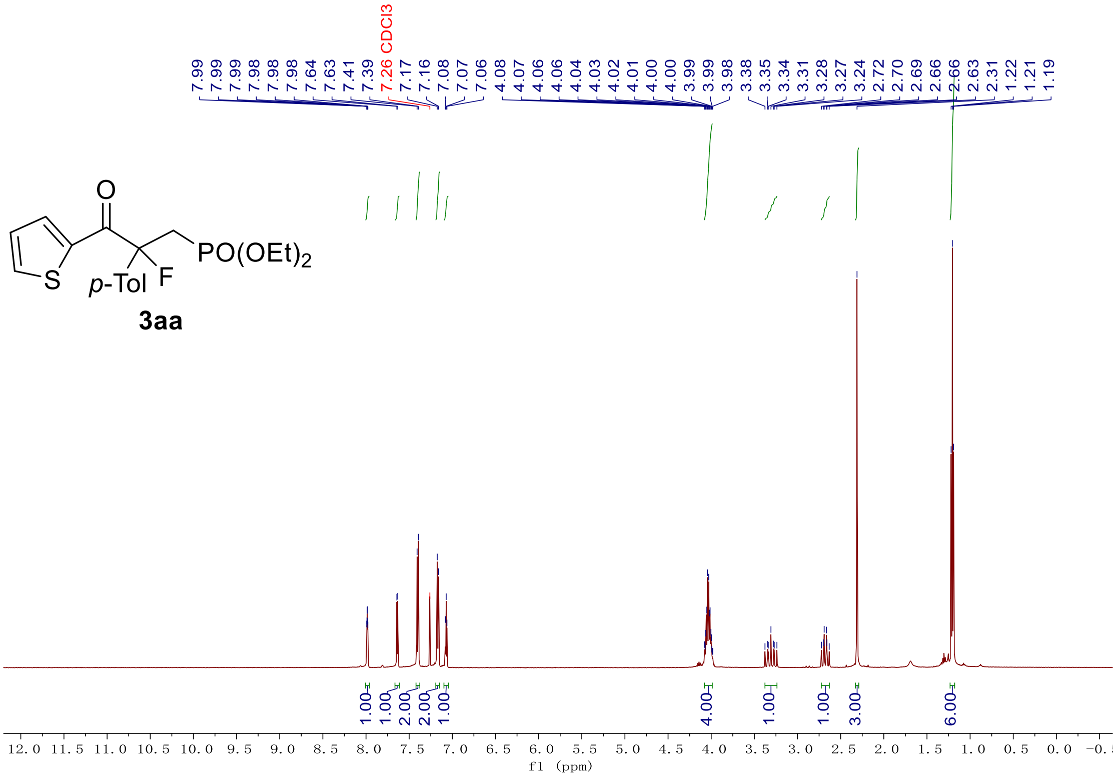

Supplementary Figure 156. ^{1}H NMR (500 MHz, $CDCl_3$) spectrum for compound **3aa**

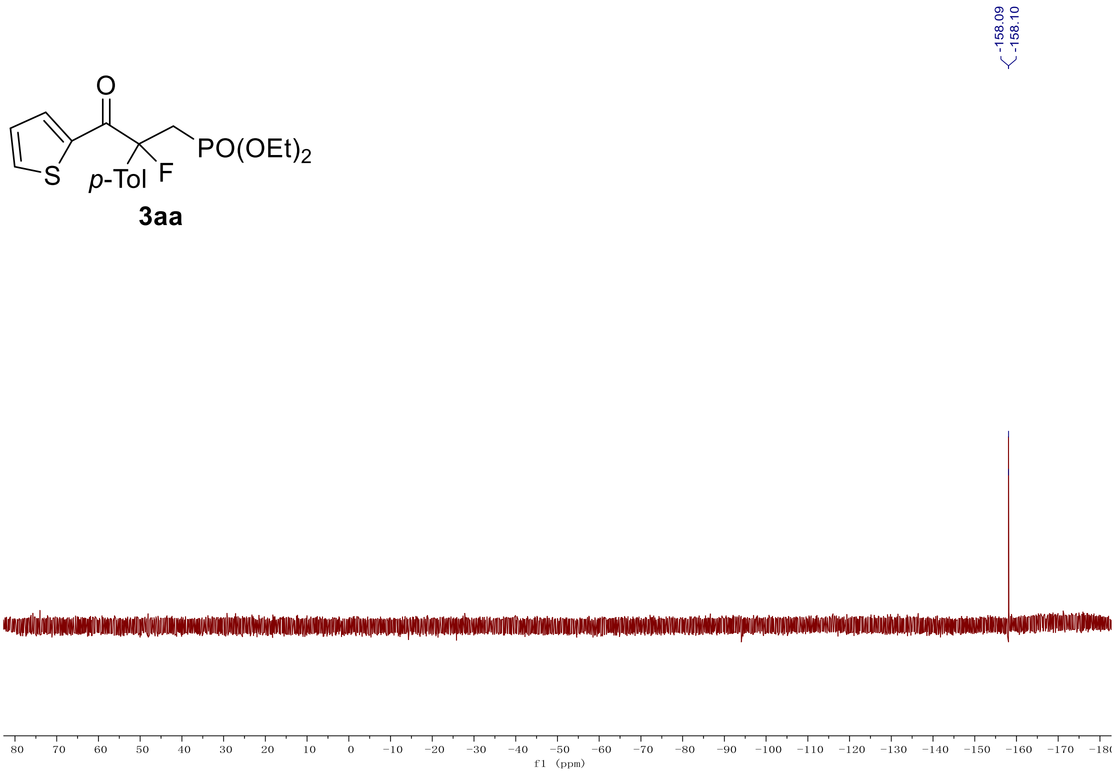

Supplementary Figure 157. ^{19}F NMR (471 MHz, $CDCl_3$) spectrum for compound **3aa**

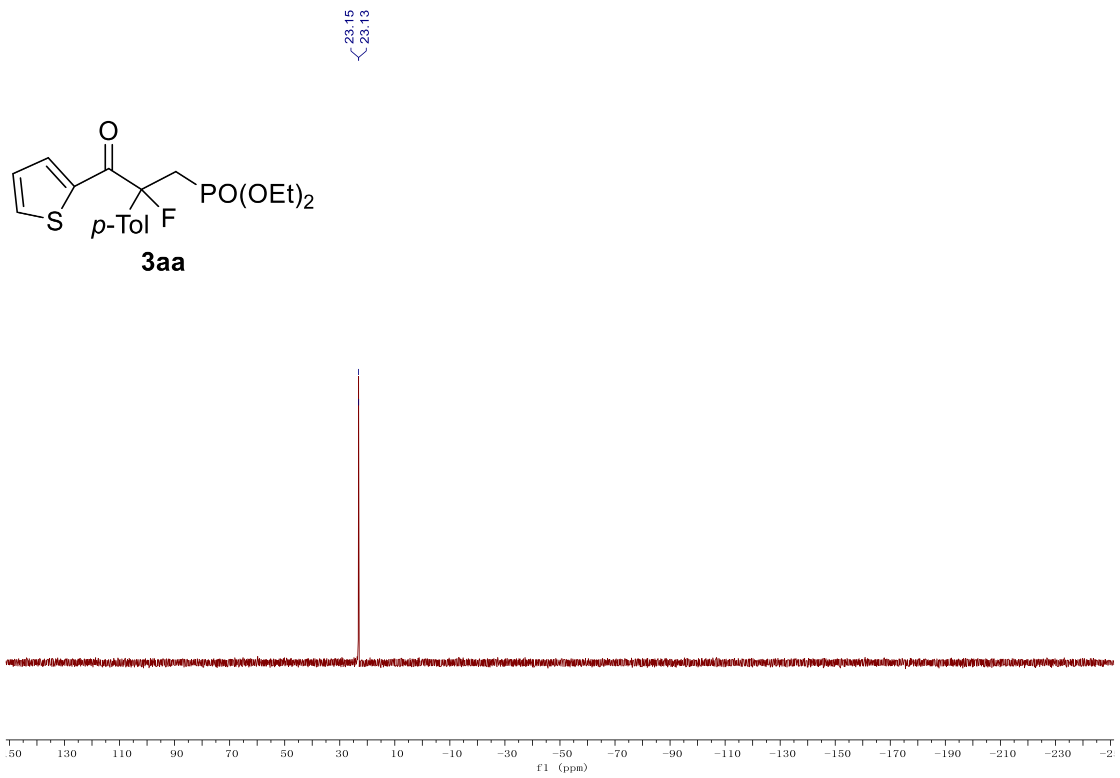

Supplementary Figure 158. ^{31}P NMR (202 MHz, $CDCl_3$) spectrum for compound **3aa**

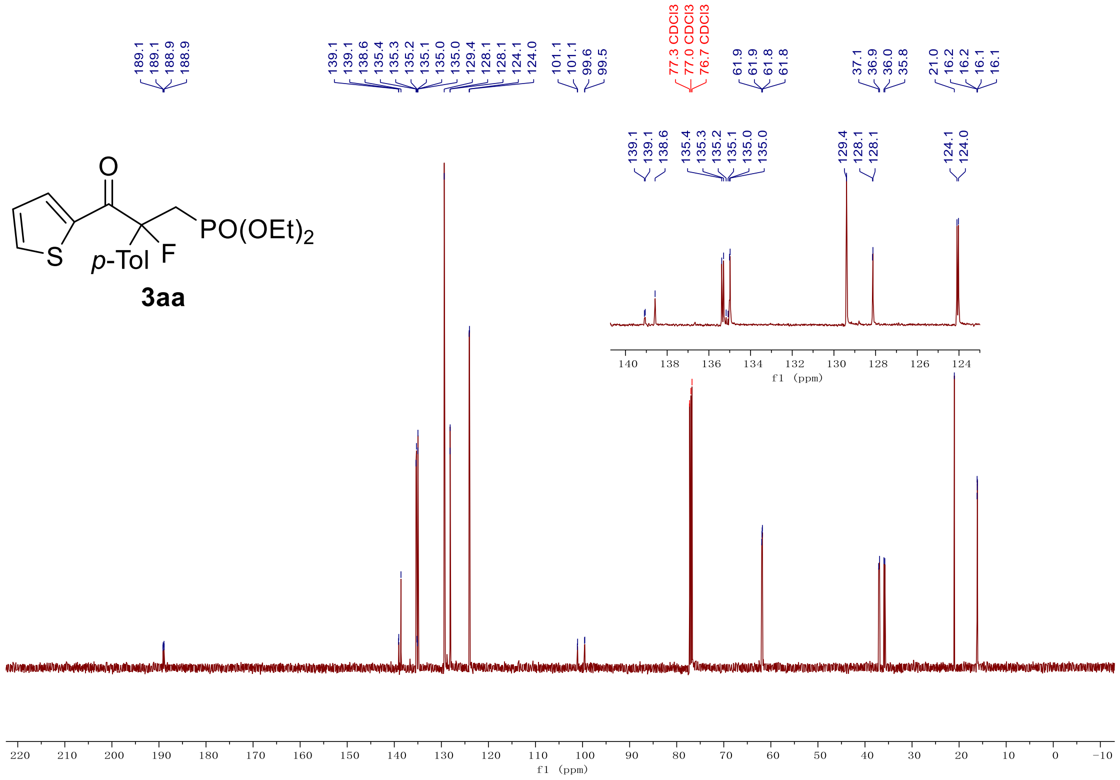

Supplementary Figure 159. ^{13}C NMR (125 MHz, $CDCl_3$) spectrum for compound **3aa**

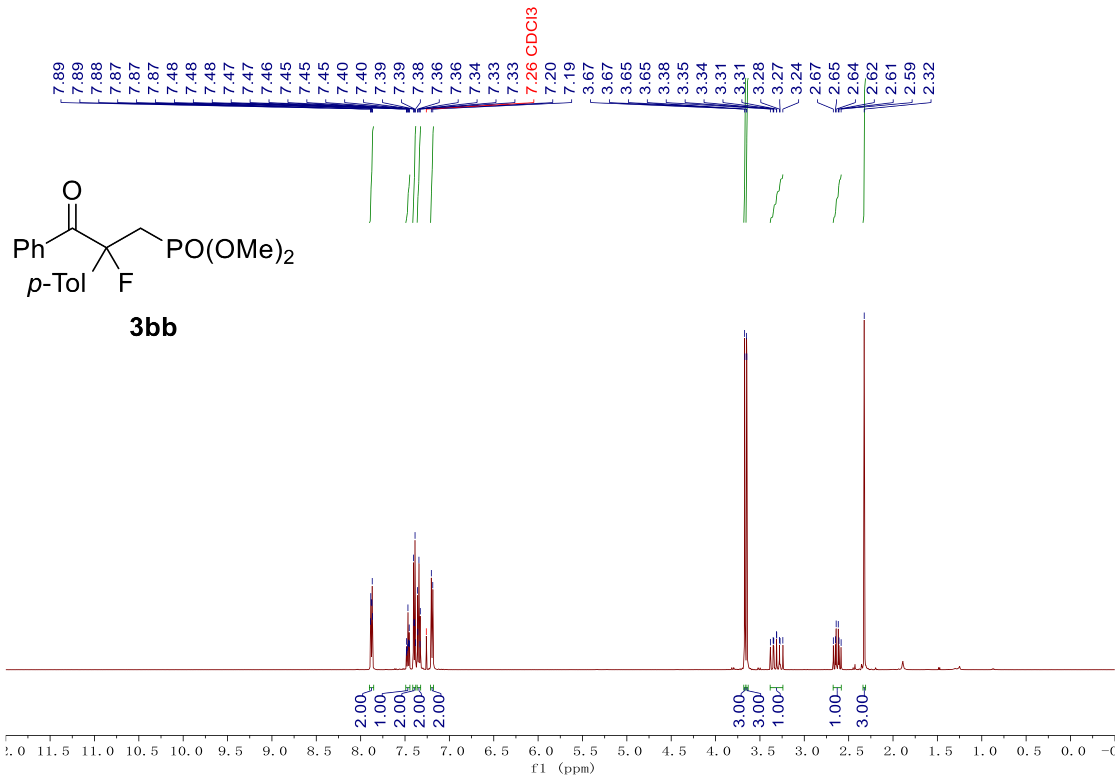

Supplementary Figure 160. ^{1}H NMR (500 MHz, $CDCl_3$) spectrum for compound **3bb**

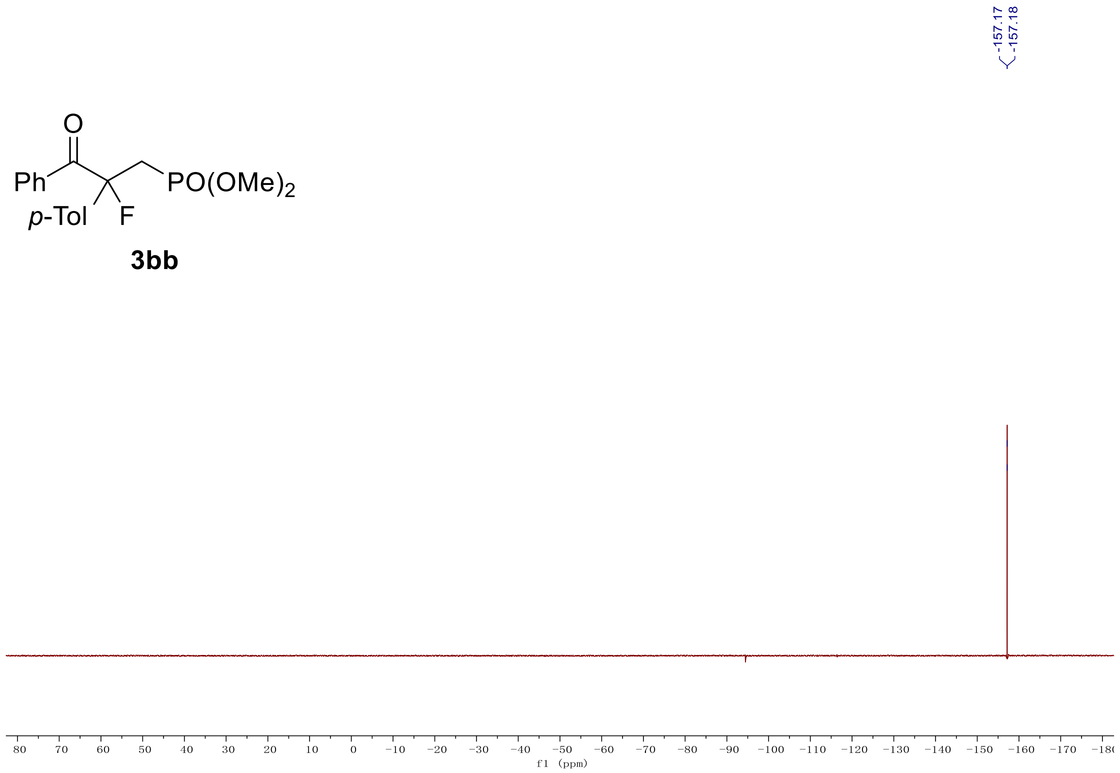

Supplementary Figure 161. ^{19}F NMR (471 MHz, $CDCl_3$) spectrum for compound **3bb**

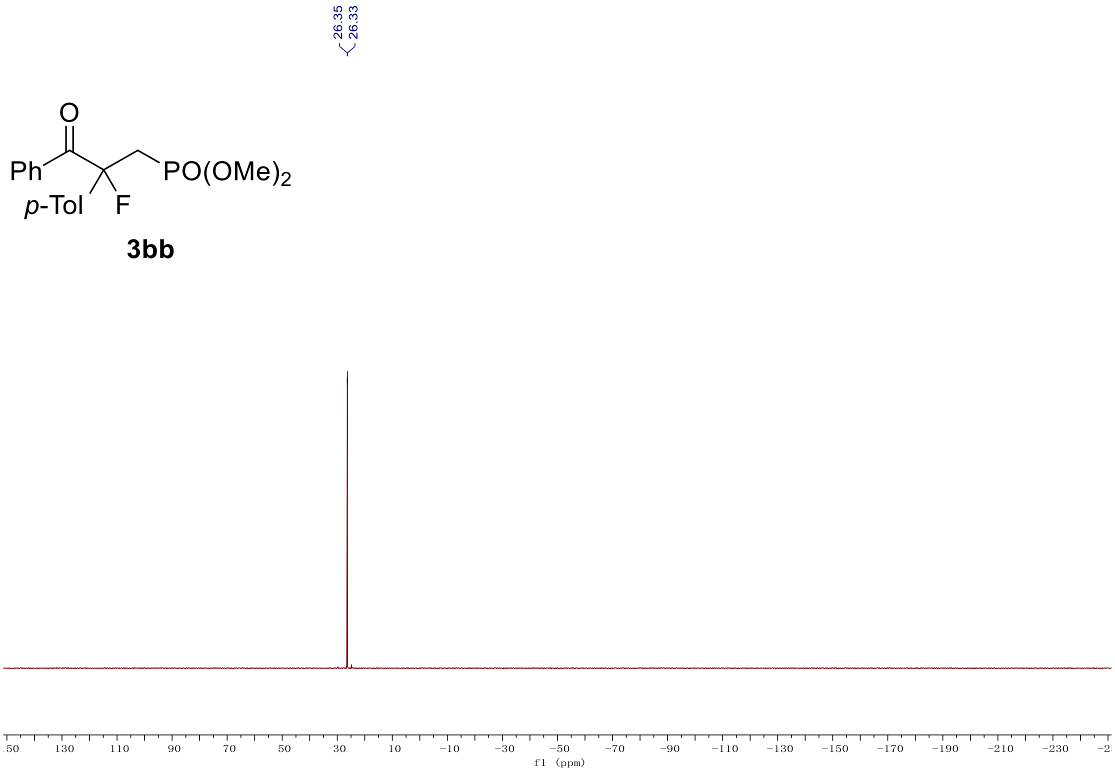

Supplementary Figure 162. ^{31}P NMR (202 MHz, CDCl$_3$) spectrum for compound **3bb**

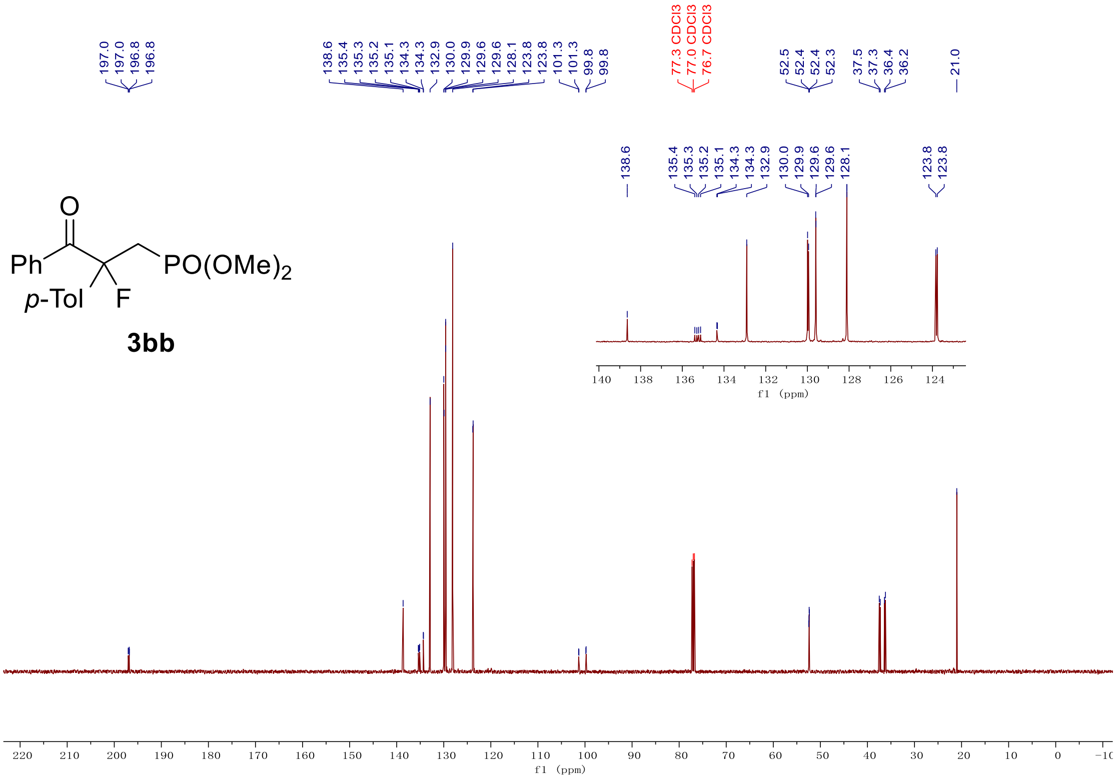

Supplementary Figure 163. ^{13}C NMR (125 MHz, CDCl$_3$) spectrum for compound **3bb**

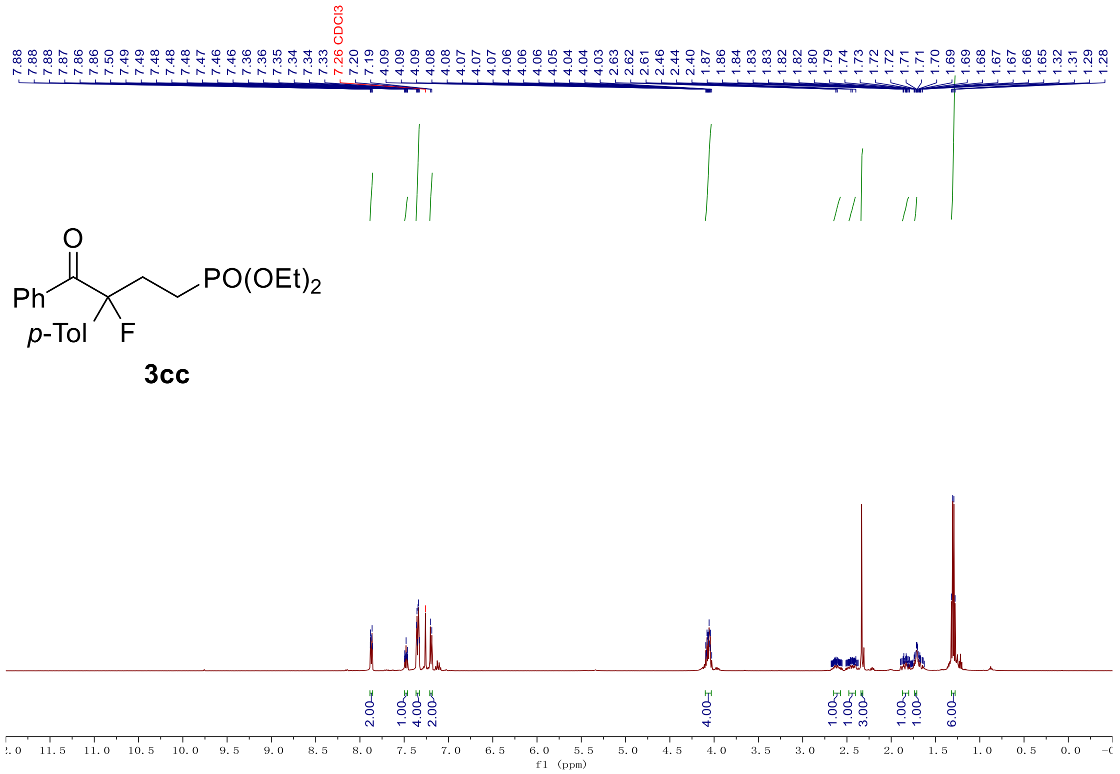

Supplementary Figure 164. ^{1}H NMR (500 MHz, $CDCl_3$) spectrum for compound **3cc**

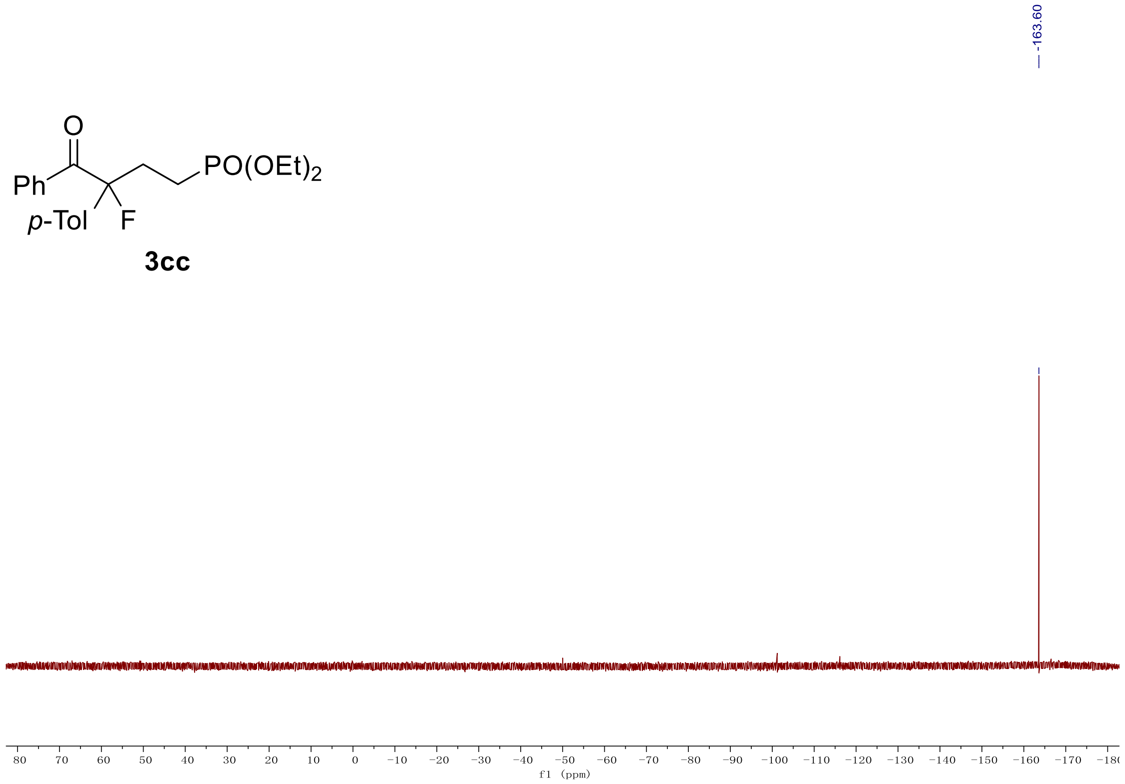

Supplementary Figure 165. ^{19}F NMR (471 MHz, $CDCl_3$) spectrum for compound **3cc**

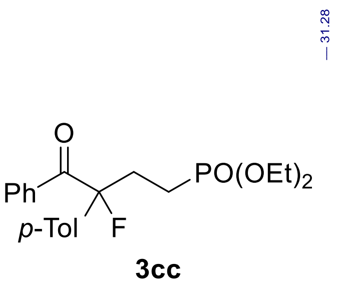

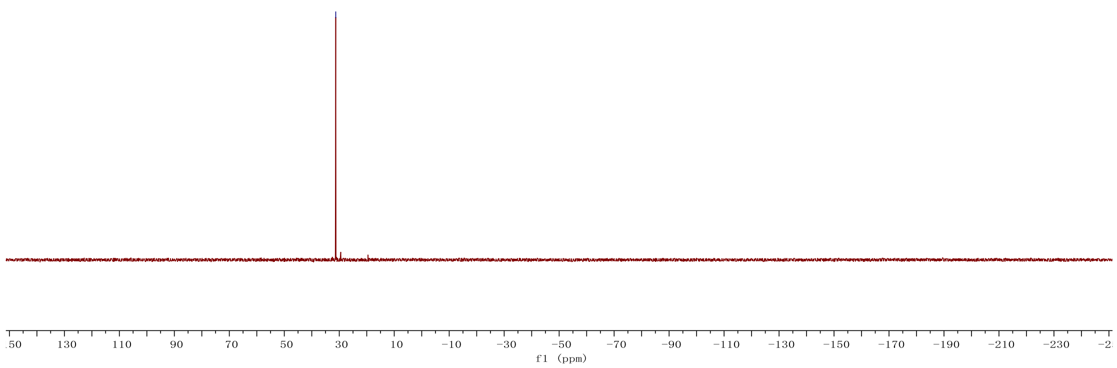

Supplementary Figure 166. ^{31}P NMR (202 MHz, $CDCl_3$) spectrum for compound **3cc**

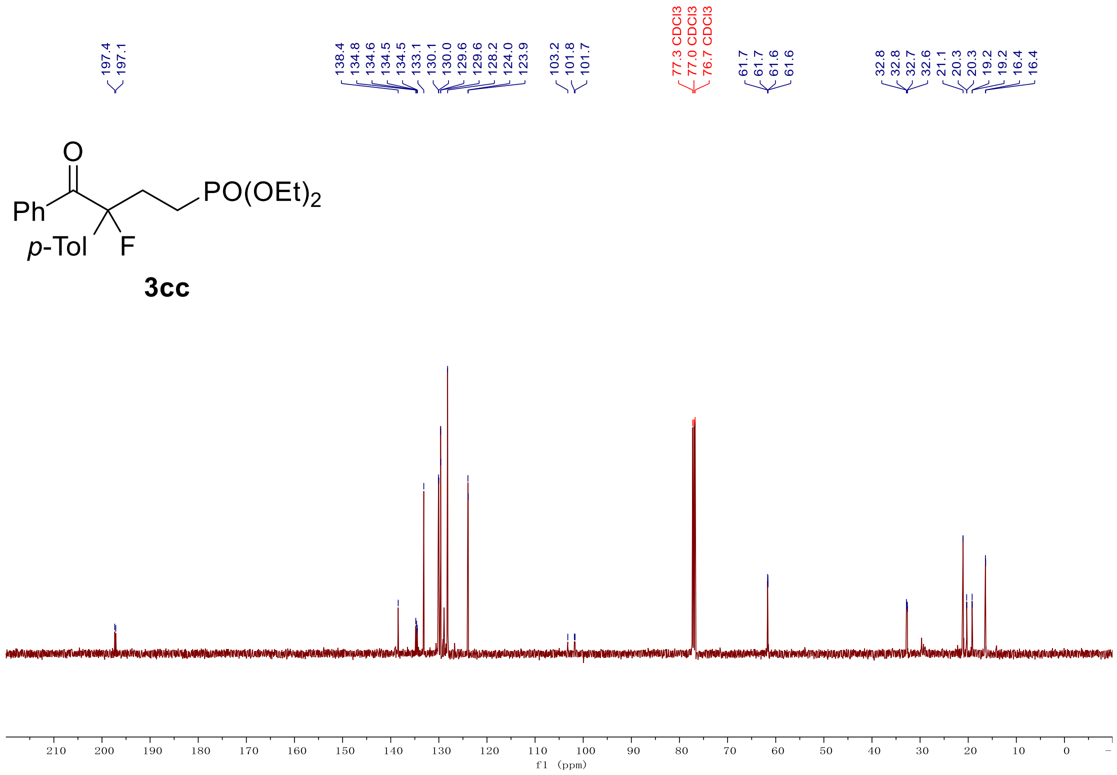

Supplementary Figure 167. ^{13}C NMR (125 MHz, $CDCl_3$) spectrum for compound **3cc**

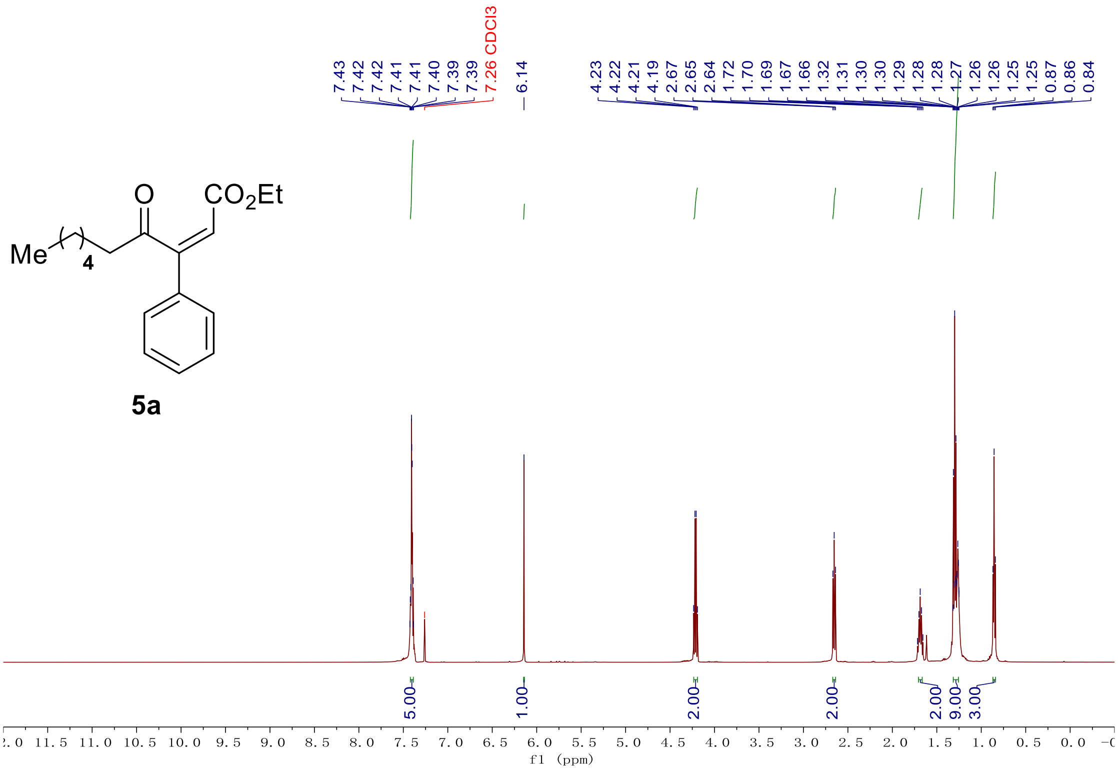

Supplementary Figure 168. ^{1}H NMR (500 MHz, $CDCl_3$) spectrum for compound **5a**

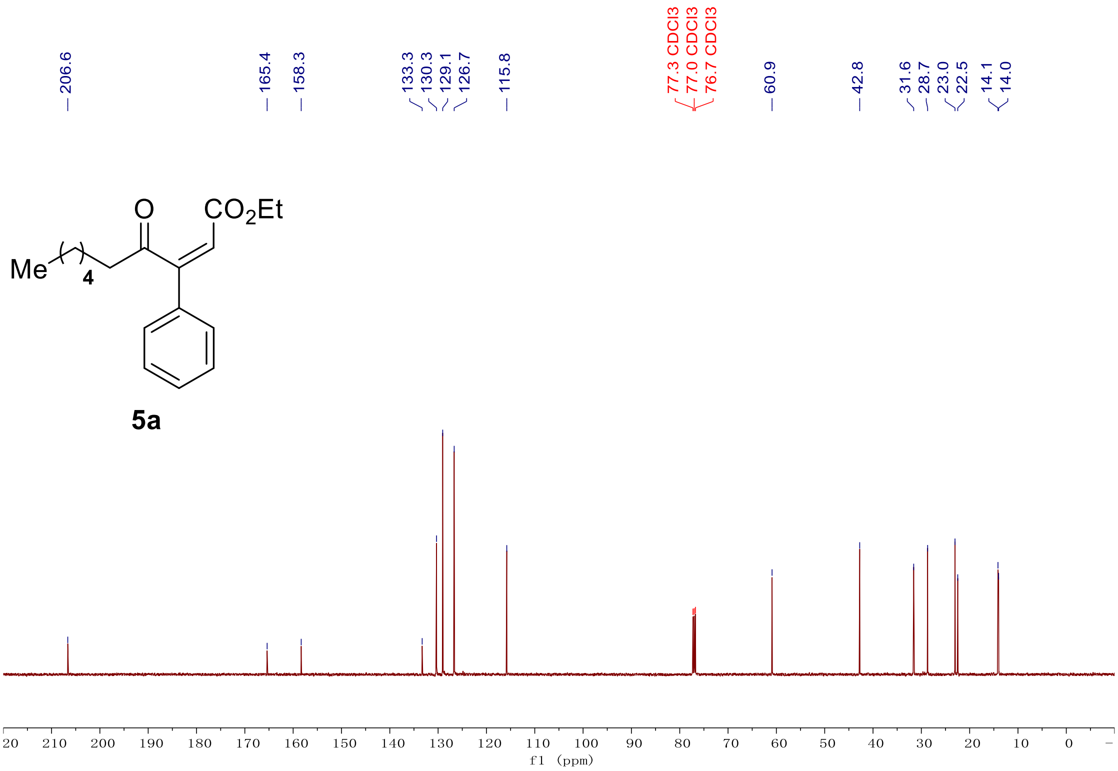

Supplementary Figure 169. ^{13}C NMR (125 MHz, $CDCl_3$) spectrum for compound **5a**

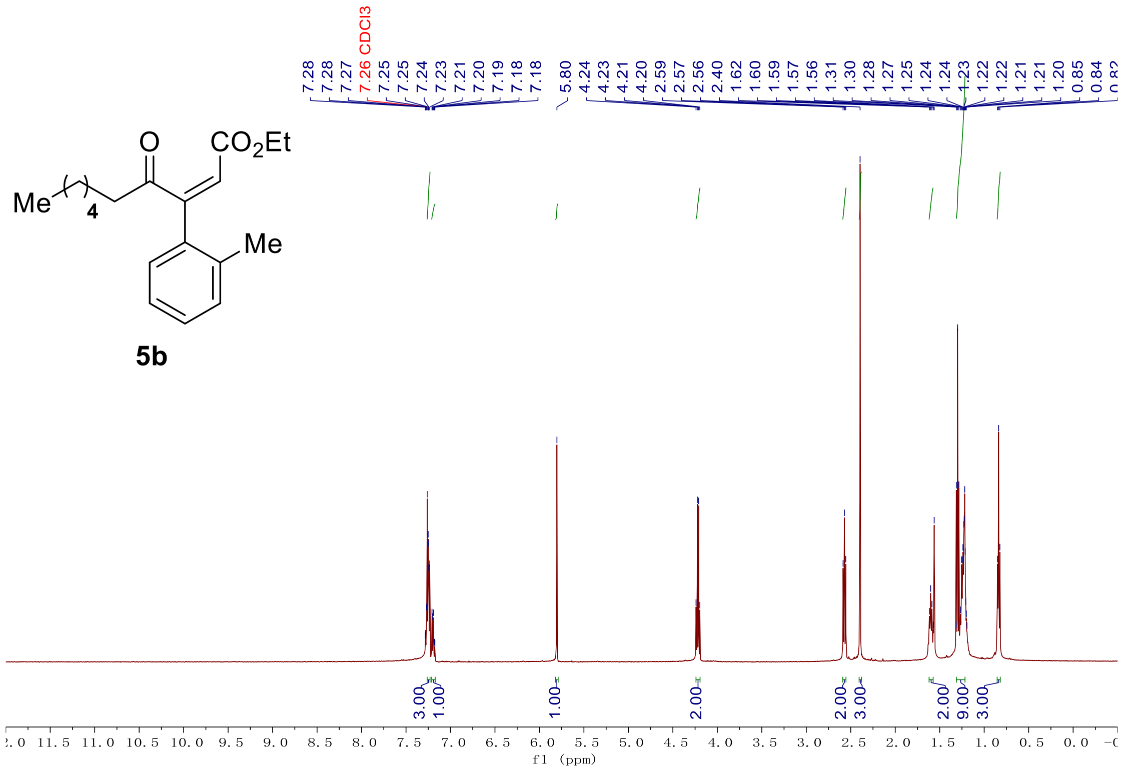

Supplementary Figure 170. ^{1}H NMR (500 MHz, $CDCl_3$) spectrum for compound **5b**

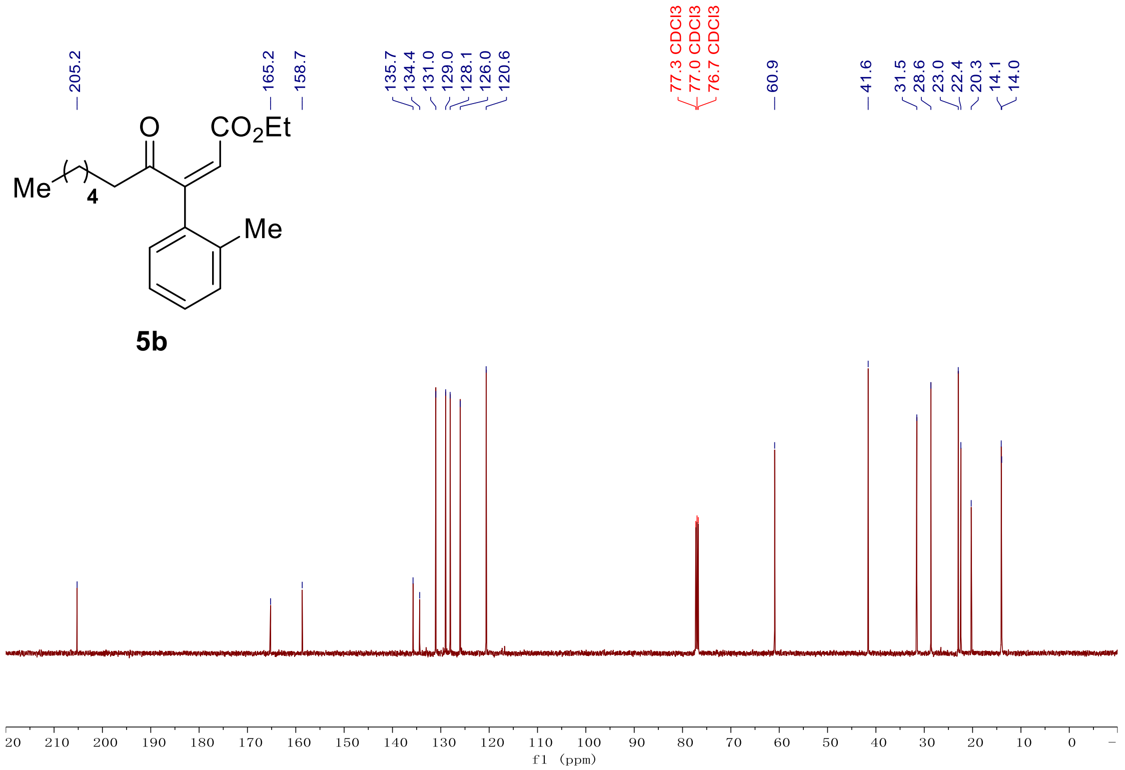

Supplementary Figure 171. ^{13}C NMR (125 MHz, $CDCl_3$) spectrum for compound **5b**

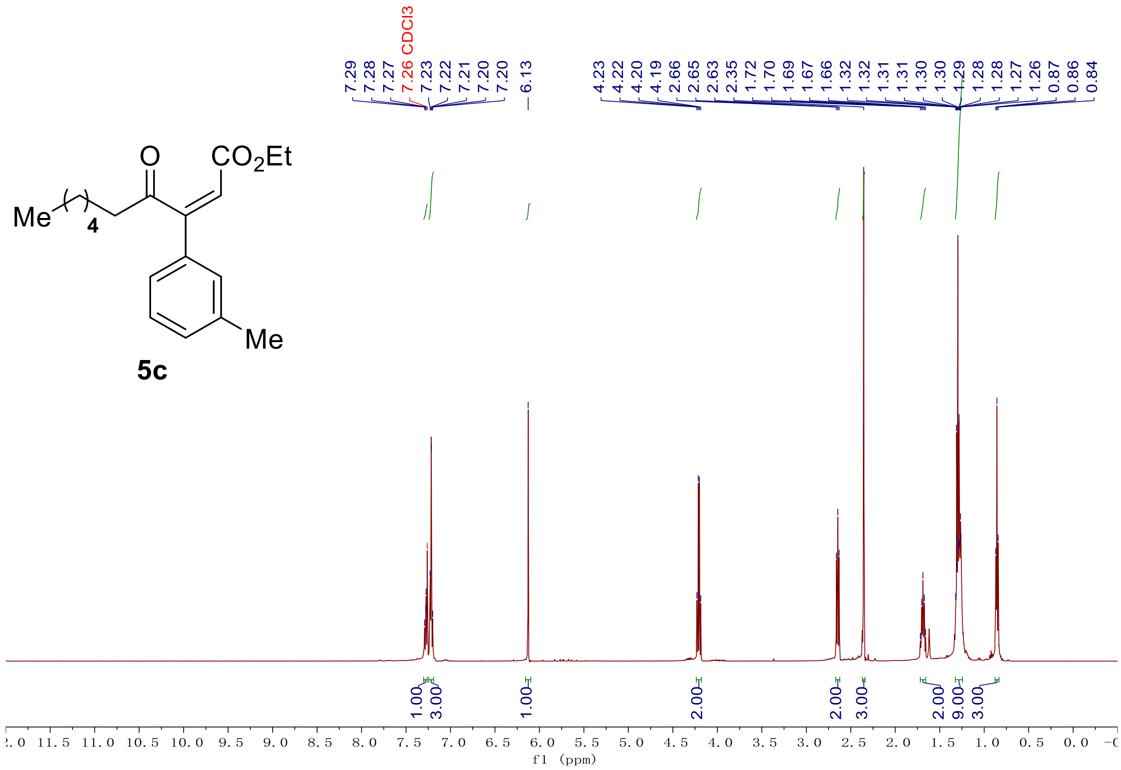

Supplementary Figure 172. ^{1}H NMR (500 MHz, $CDCl_3$) spectrum for compound **5c**

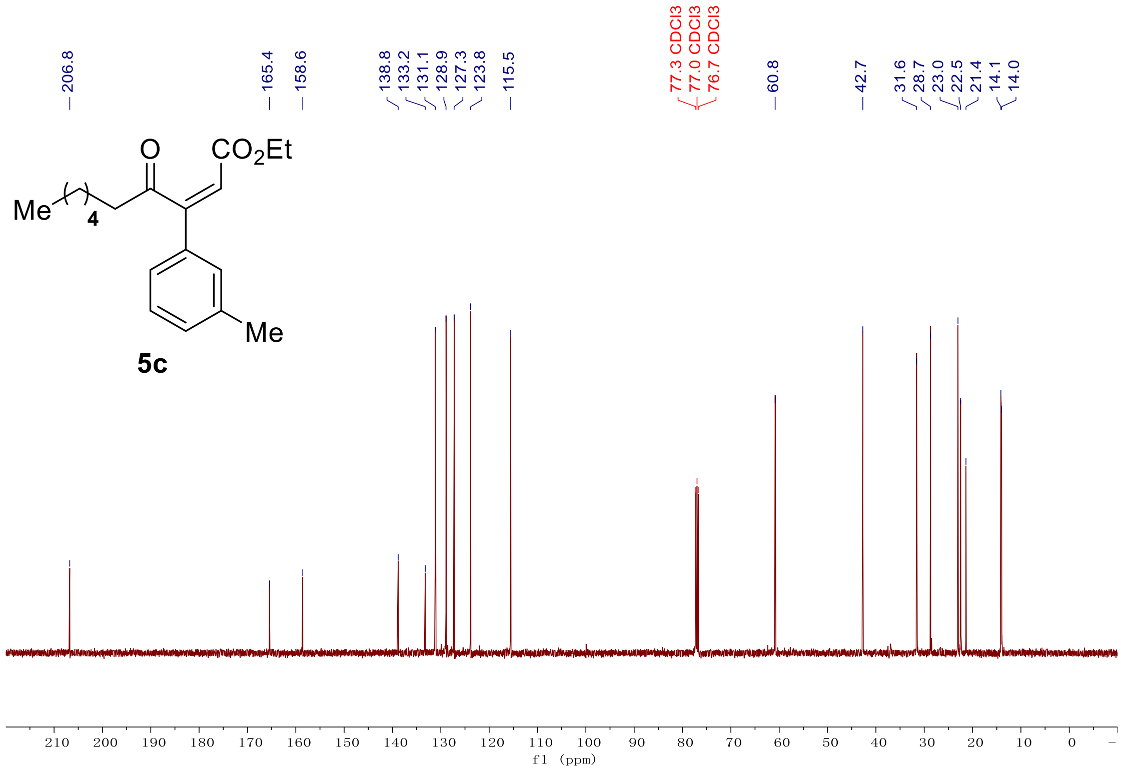

Supplementary Figure 173. ^{13}C NMR (125 MHz, $CDCl_3$) spectrum for compound **5c**

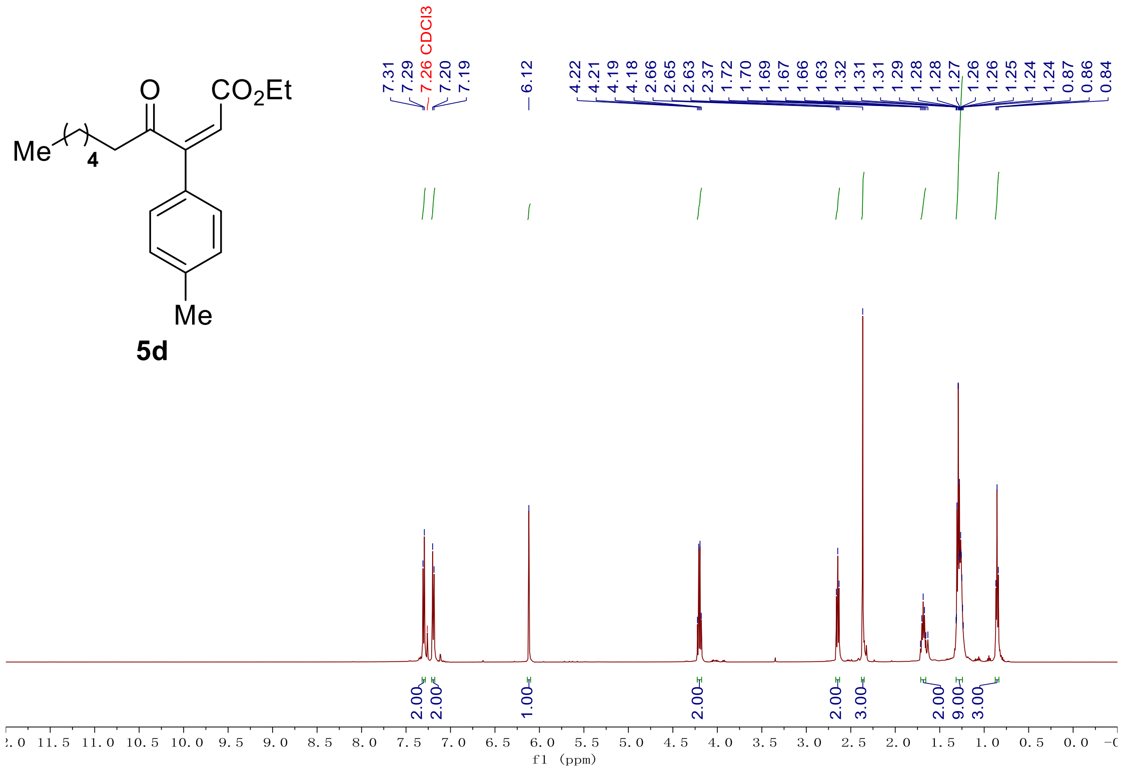

Supplementary Figure 174. ^{1}H NMR (500 MHz, $CDCl_3$) spectrum for compound **5d**

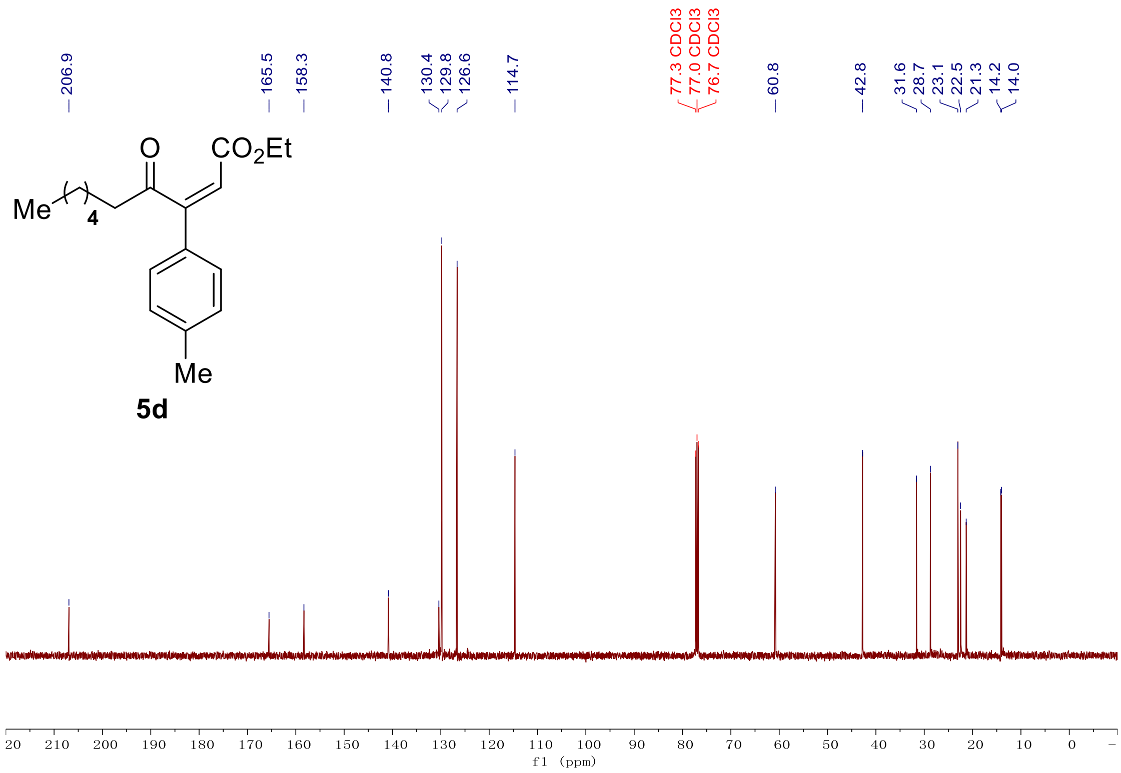

Supplementary Figure 175. ^{13}C NMR (125 MHz, $CDCl_3$) spectrum for compound **5d**

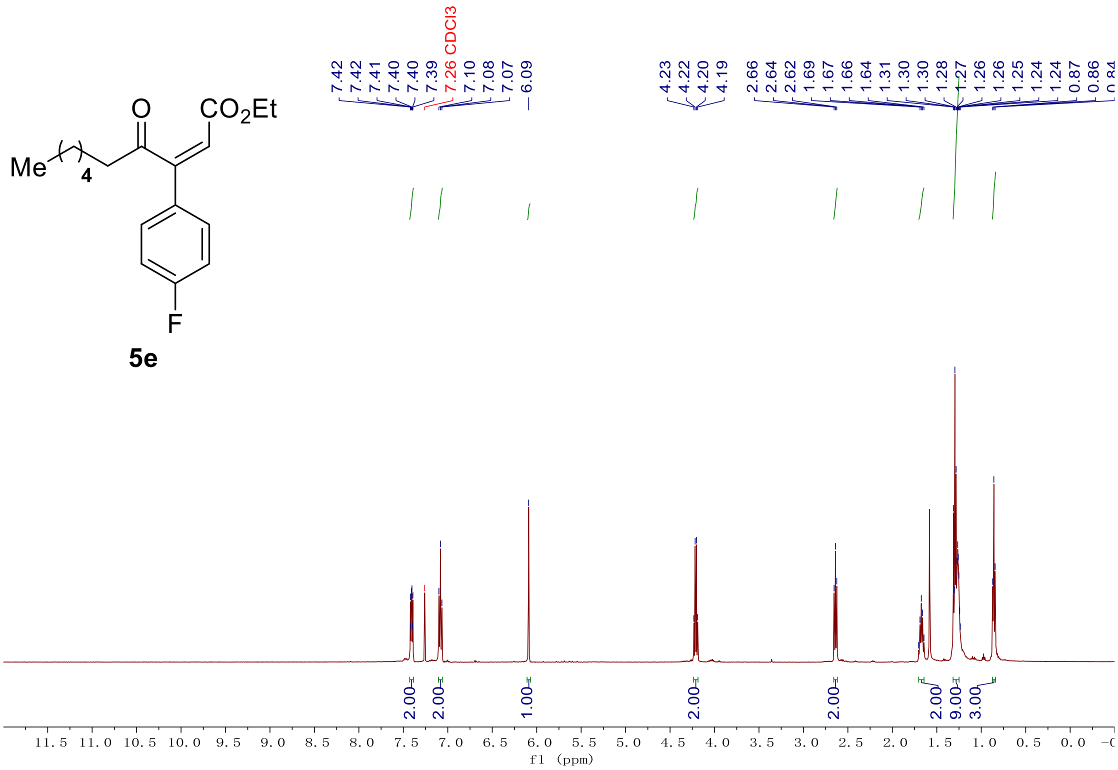

Supplementary Figure 176. ^{1}H NMR (500 MHz, $CDCl_3$) spectrum for compound **5e**

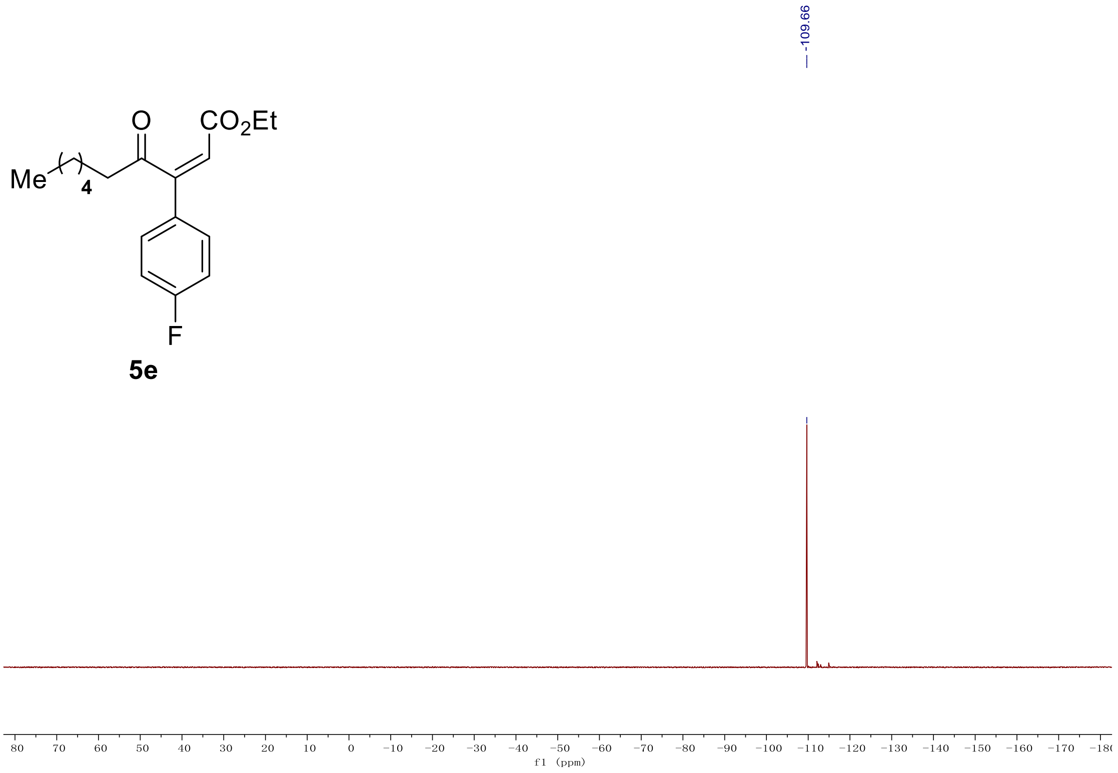

Supplementary Figure 177. ^{19}F NMR (471 MHz, $CDCl_3$) spectrum for compound **5e**

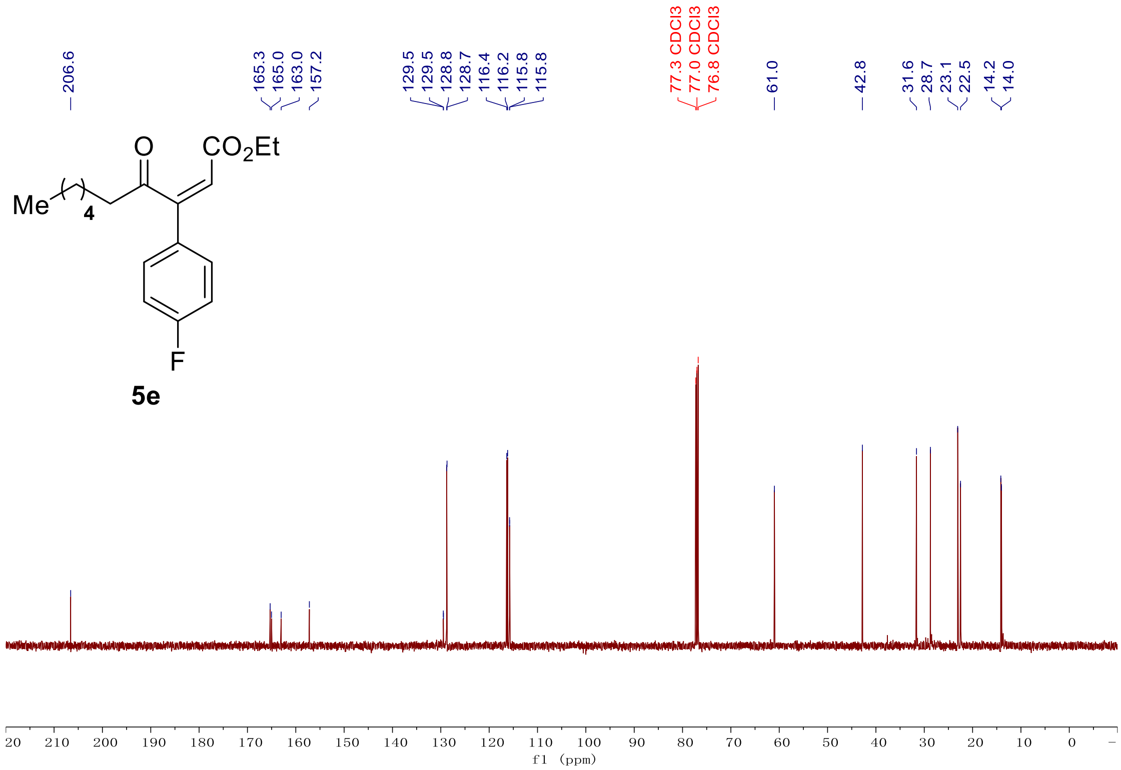

Supplementary Figure 178. ^{13}C NMR (125 MHz, $CDCl_3$) spectrum for compound **5e**

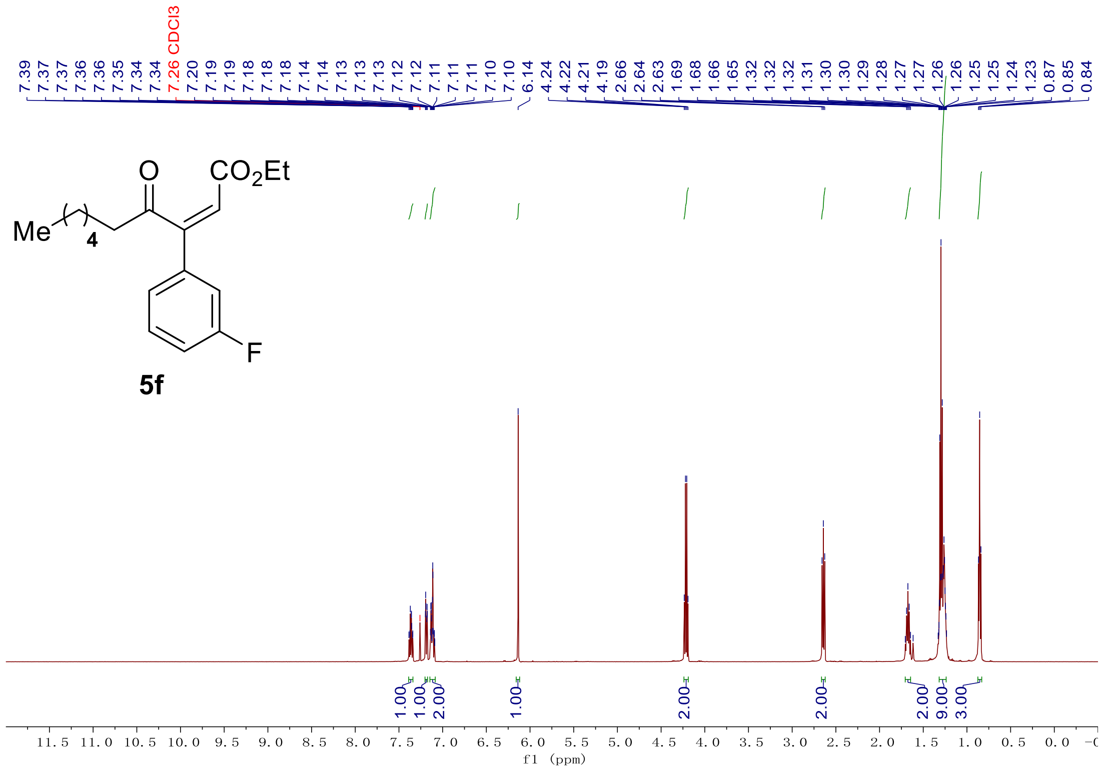

Supplementary Figure 179. ^{1}H NMR (500 MHz, $CDCl_3$) spectrum for compound **5f**

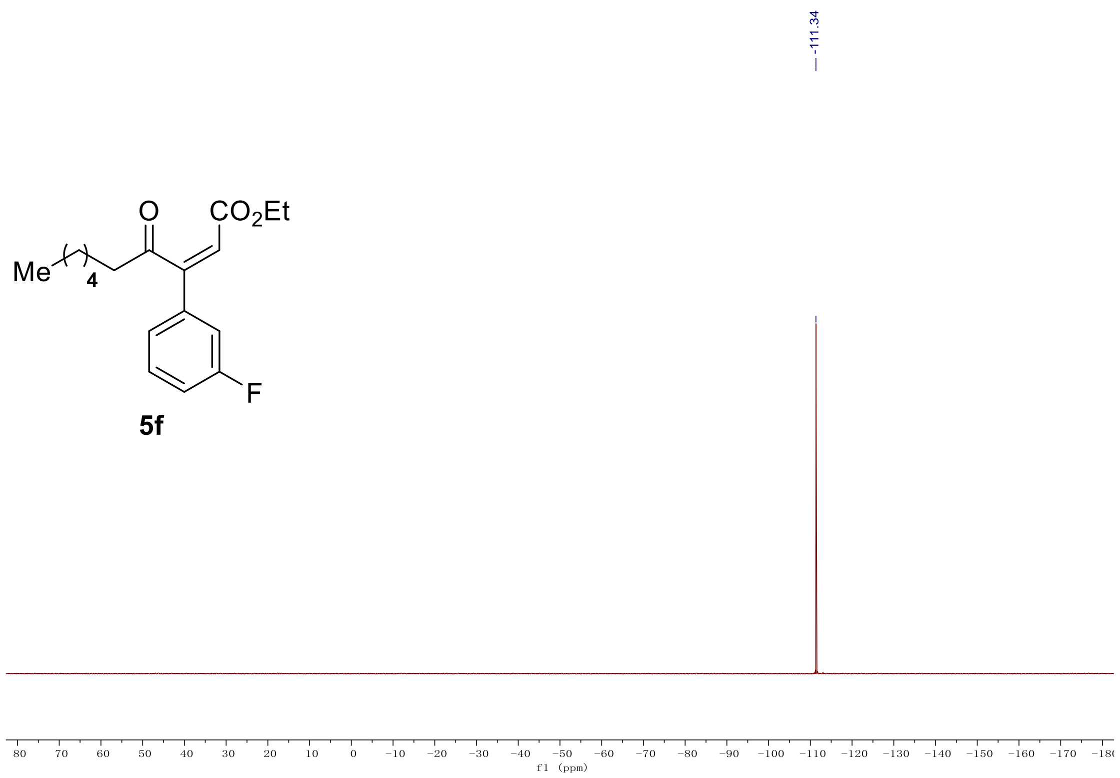

Supplementary Figure 180. ^{19}F NMR (471 MHz, $CDCl_3$) spectrum for compound **5f**

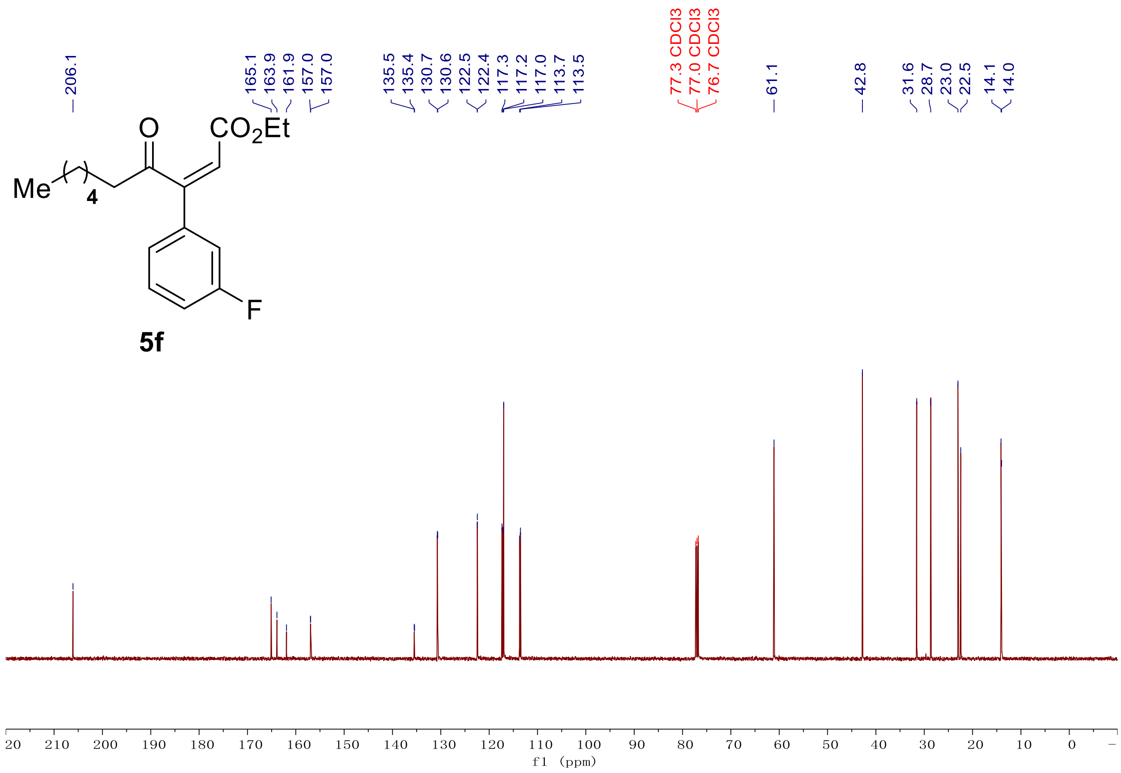

Supplementary Figure 181. ^{13}C NMR (125 MHz, $CDCl_3$) spectrum for compound **5f**

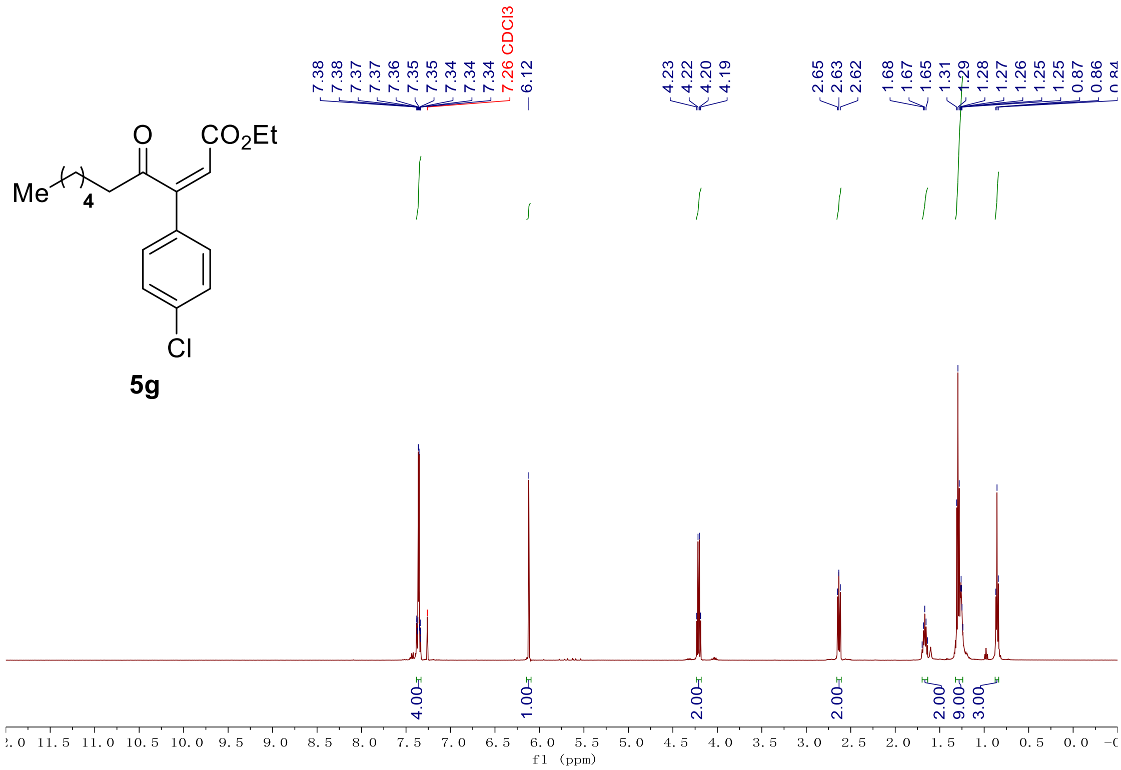

Supplementary Figure 182. ^{1}H NMR (500 MHz, $CDCl_3$) spectrum for compound **5g**

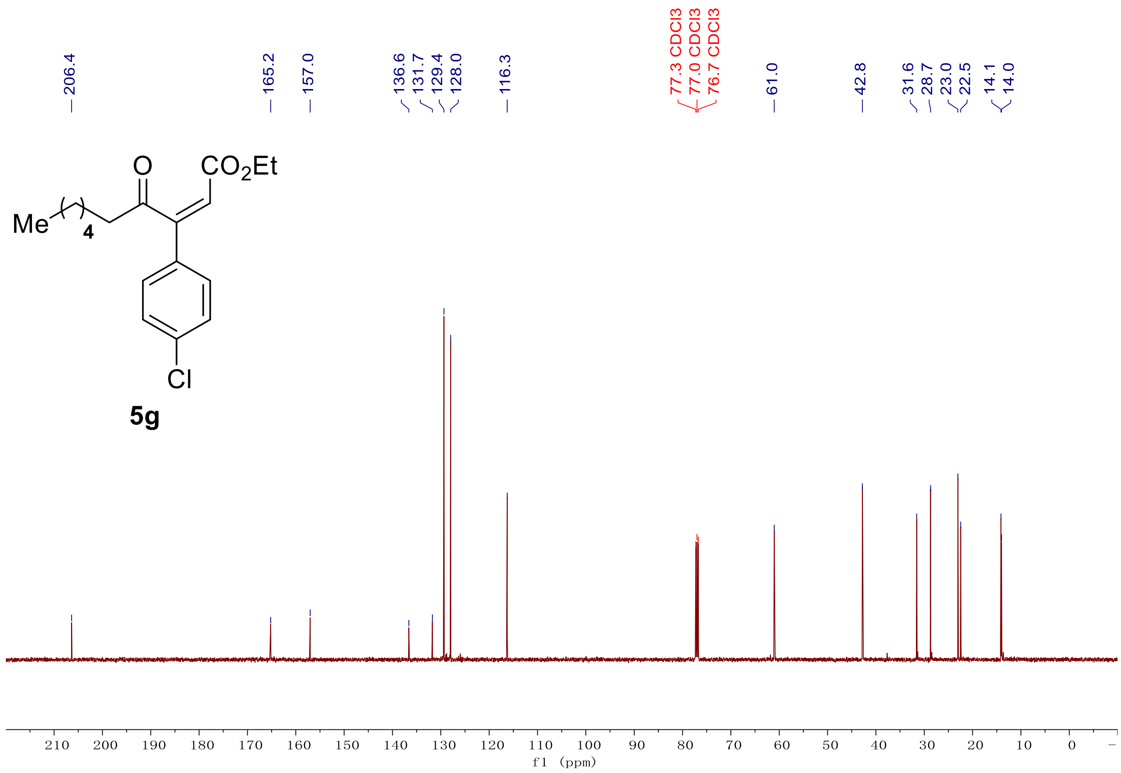

Supplementary Figure 183. ^{13}C NMR (125 MHz, $CDCl_3$) spectrum for compound **5g**

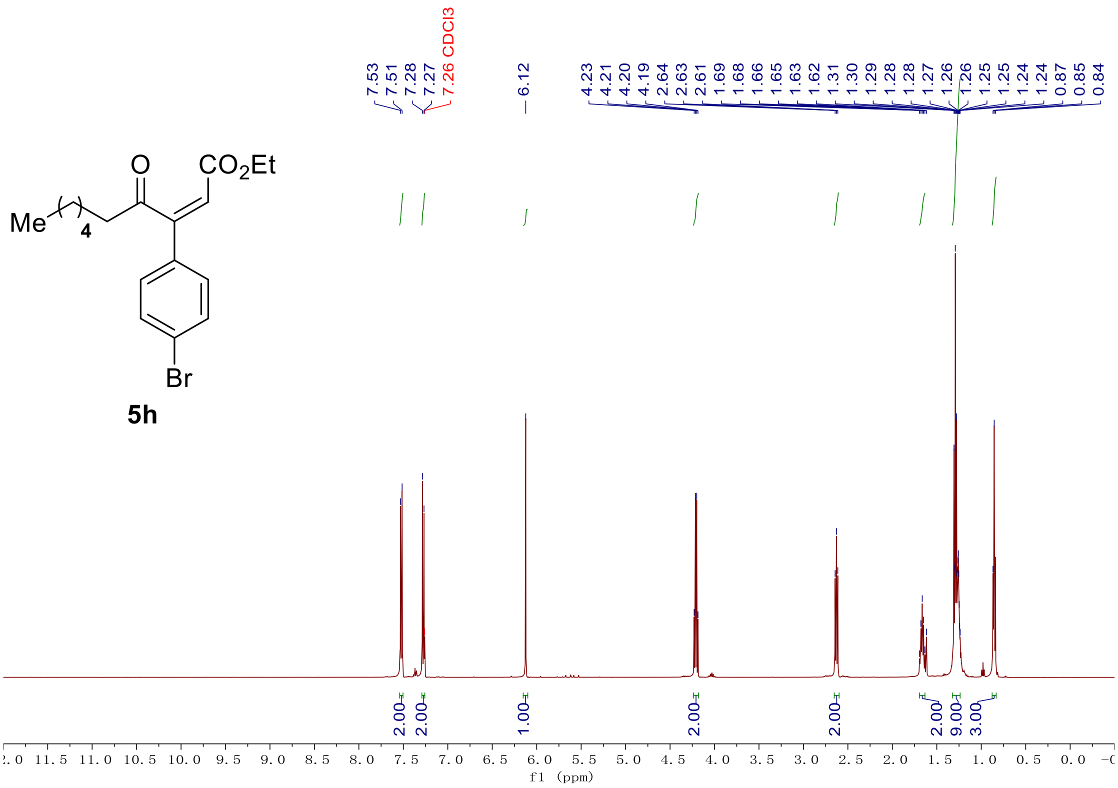

Supplementary Figure 184. ^{1}H NMR (500 MHz, $CDCl_3$) spectrum for compound **5h**

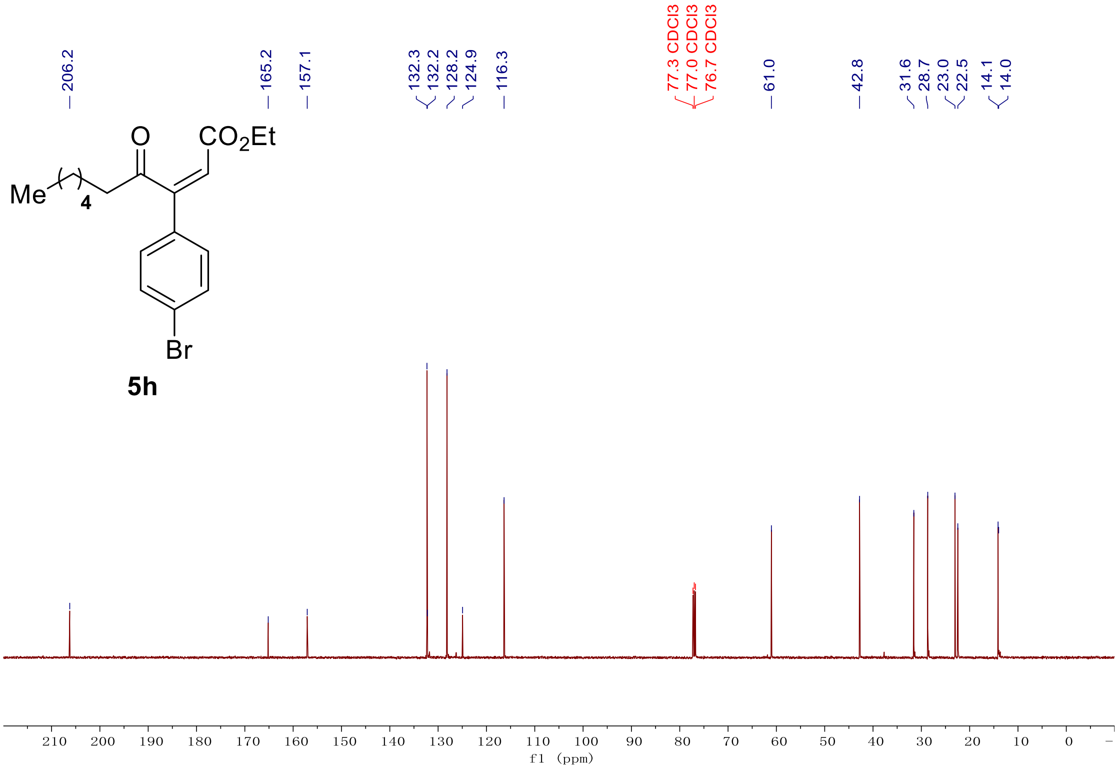

Supplementary Figure 185. ^{13}C NMR (125 MHz, $CDCl_3$) spectrum for compound **5h**

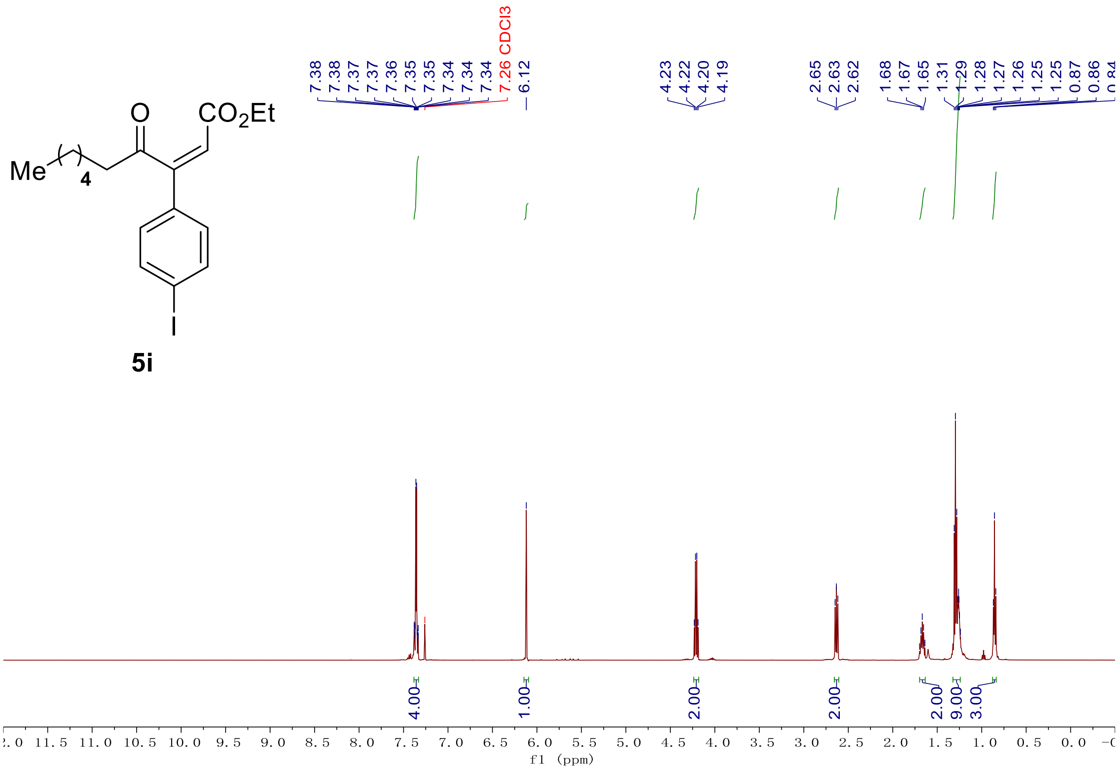

Supplementary Figure 186. ^{1}H NMR (500 MHz, $CDCl_3$) spectrum for compound **5i**

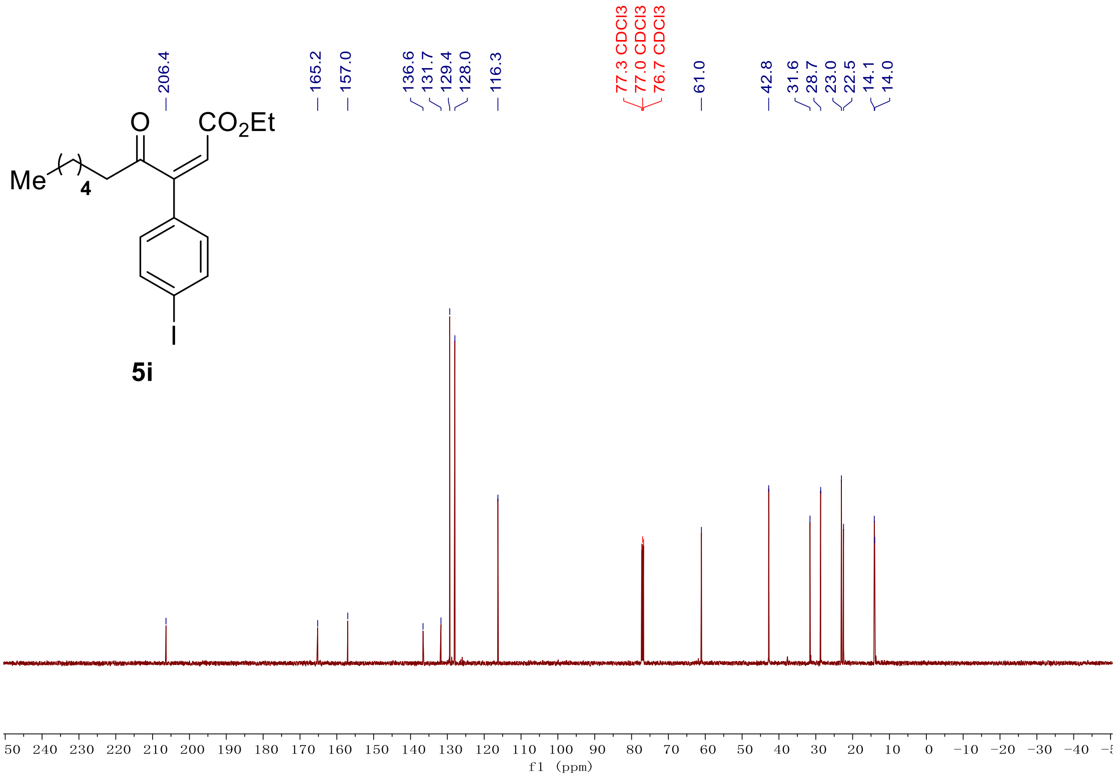

Supplementary Figure 187. ^{13}C NMR (125 MHz, $CDCl_3$) spectrum for compound **5i**

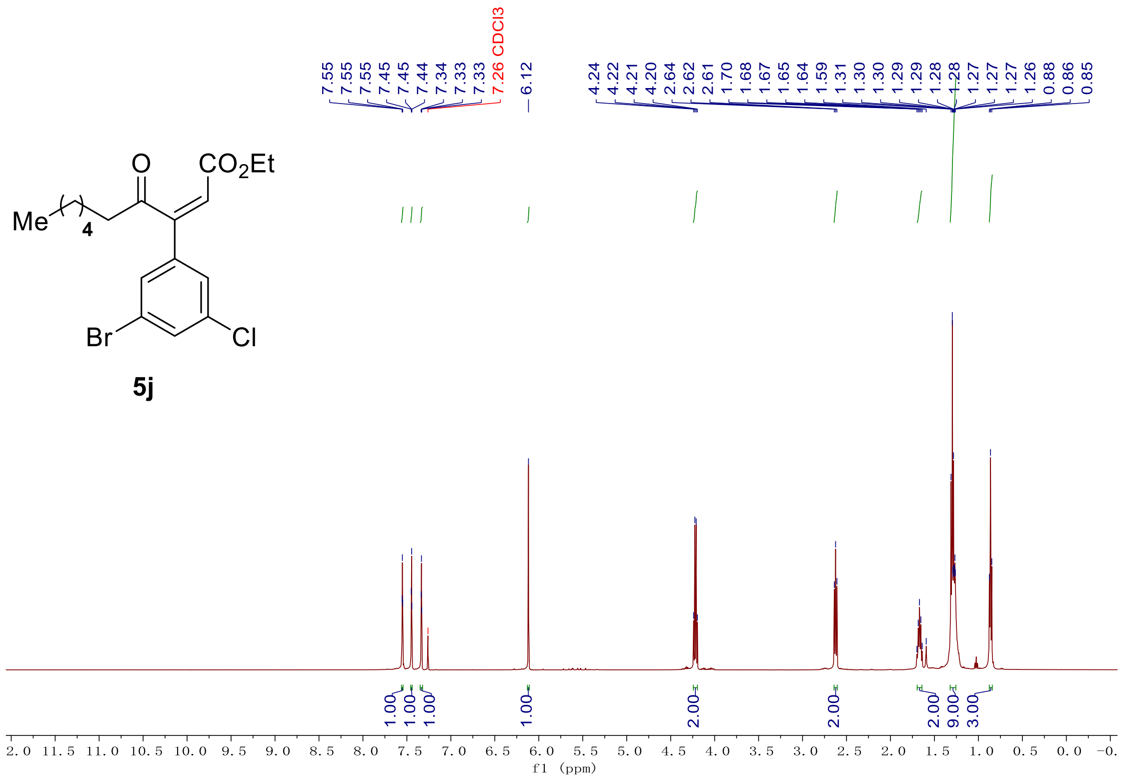

Supplementary Figure 188. ^{1}H NMR (500 MHz, $CDCl_3$) spectrum for compound **5j**

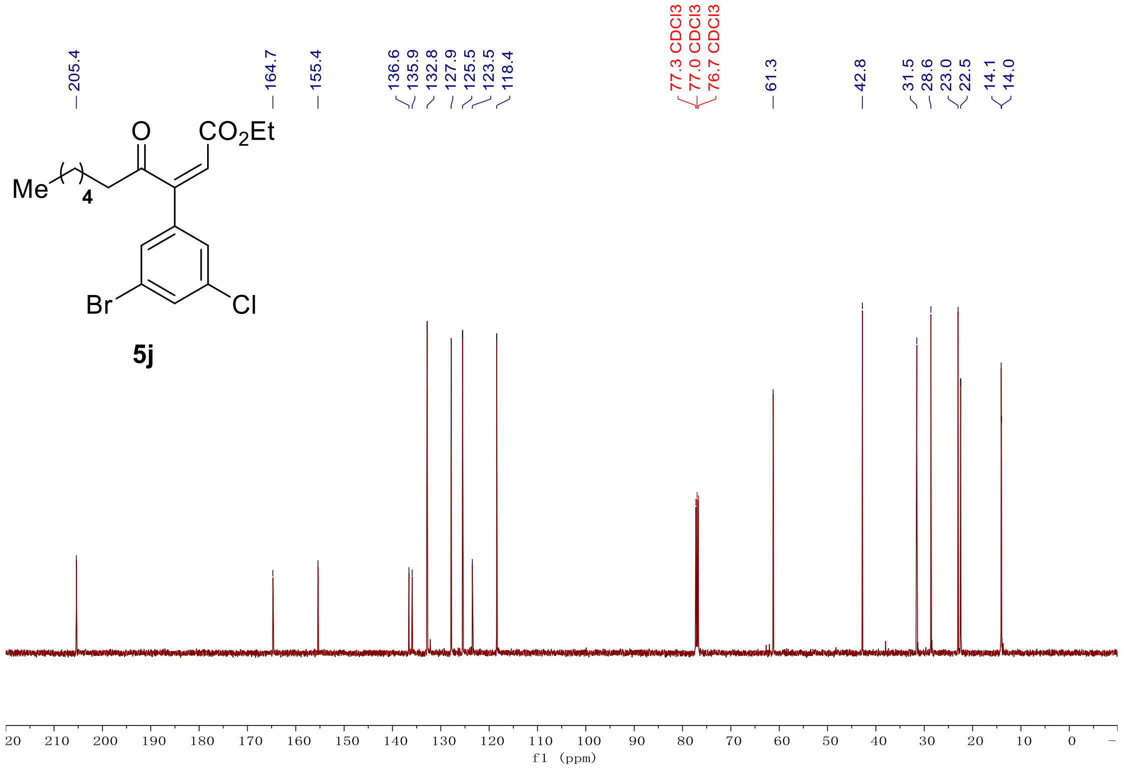

Supplementary Figure 189. ^{13}C NMR (125 MHz, $CDCl_3$) spectrum for compound **5j**

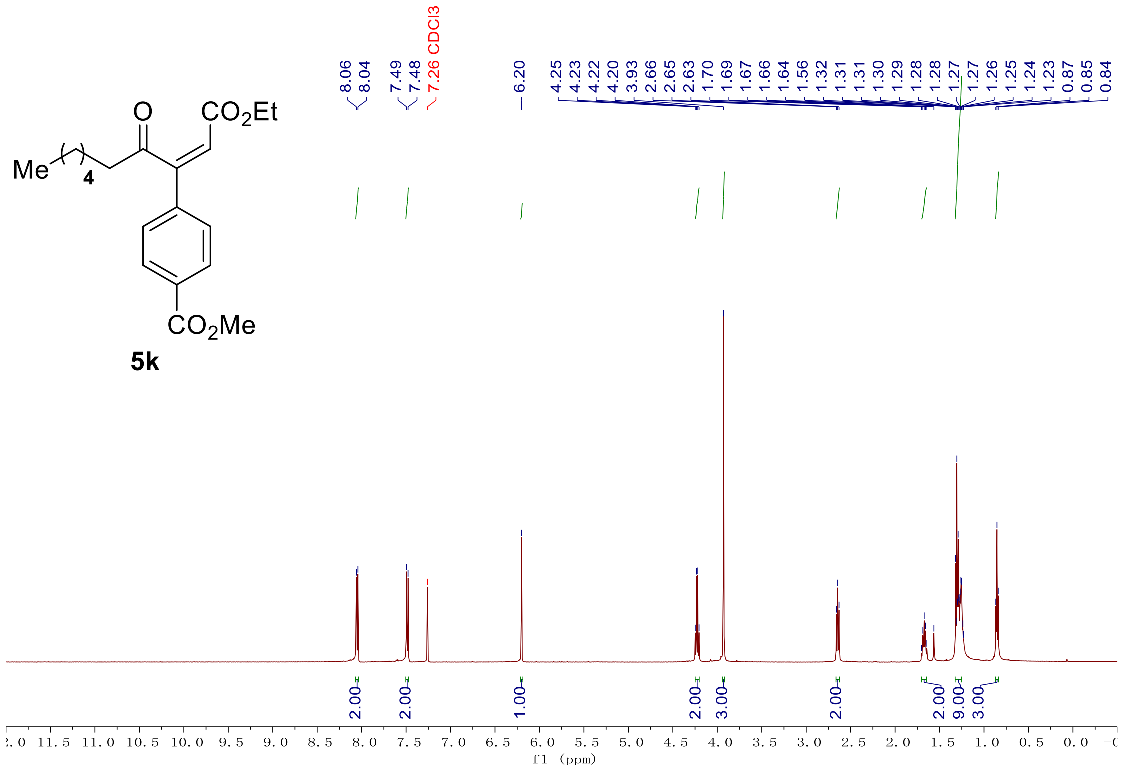

Supplementary Figure 190. ^{1}H NMR (500 MHz, $CDCl_3$) spectrum for compound **5k**

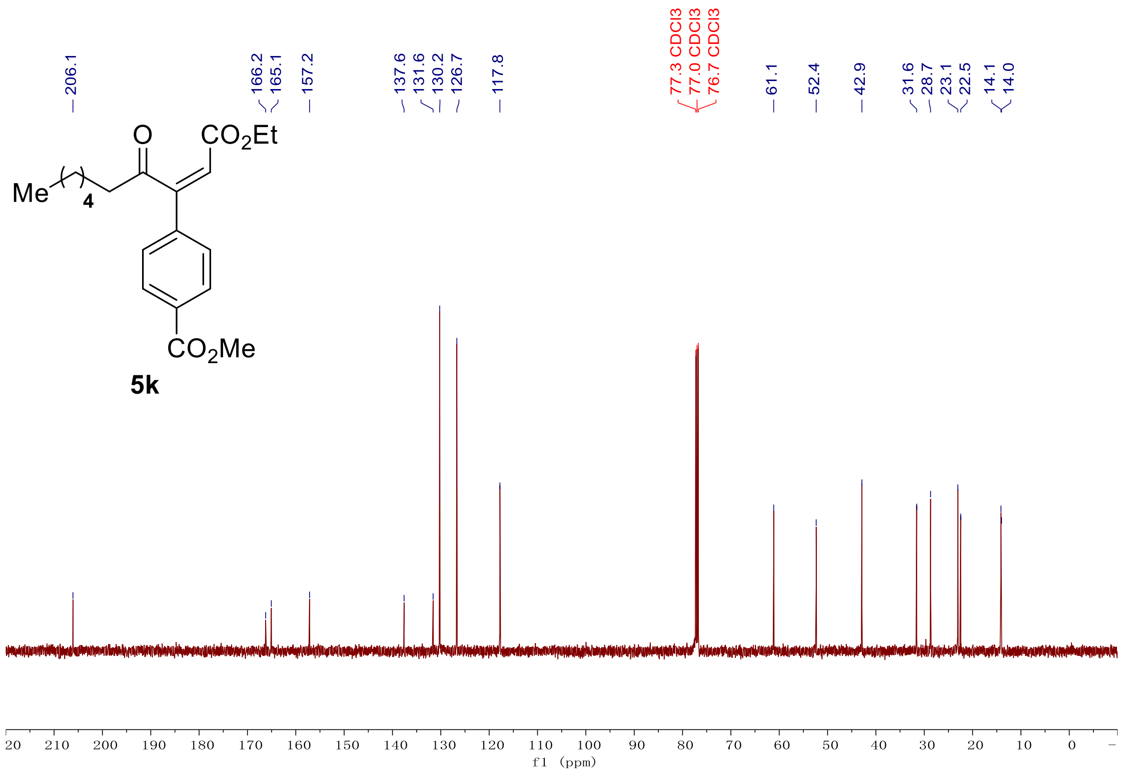

Supplementary Figure 191. ^{13}C NMR (125 MHz, $CDCl_3$) spectrum for compound **5k**

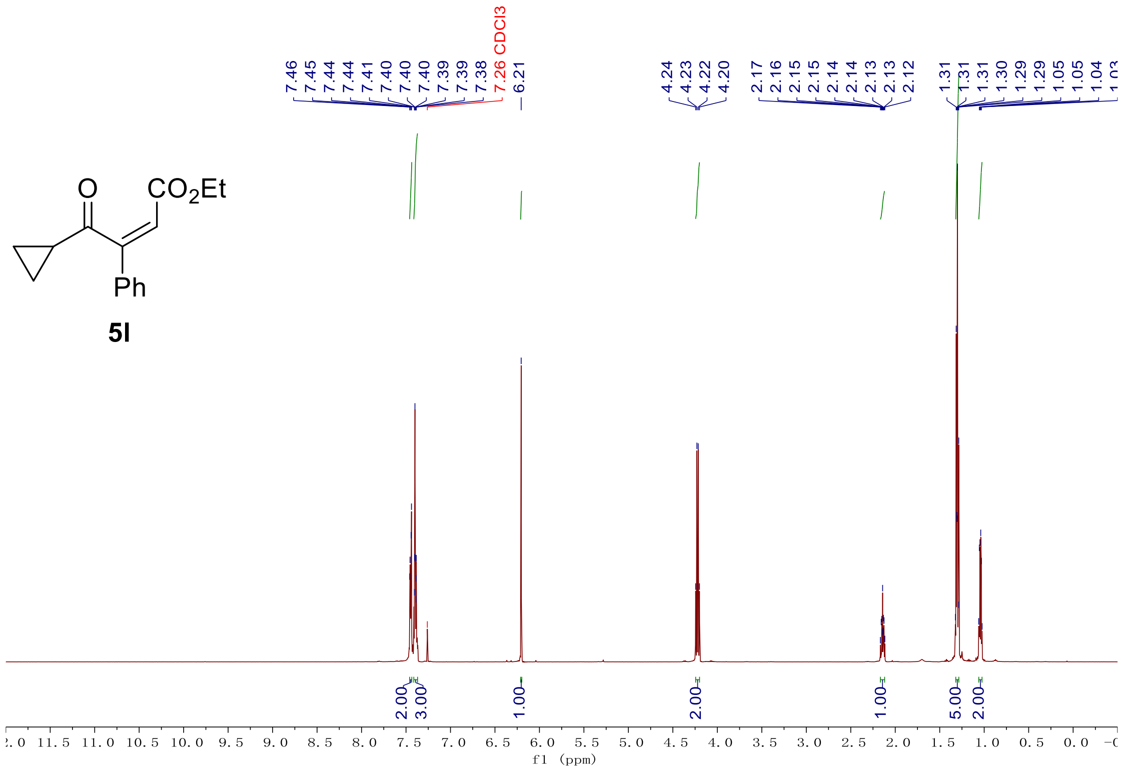

Supplementary Figure 192. ^{1}H NMR (500 MHz, CDCl3) spectrum for compound **5l**

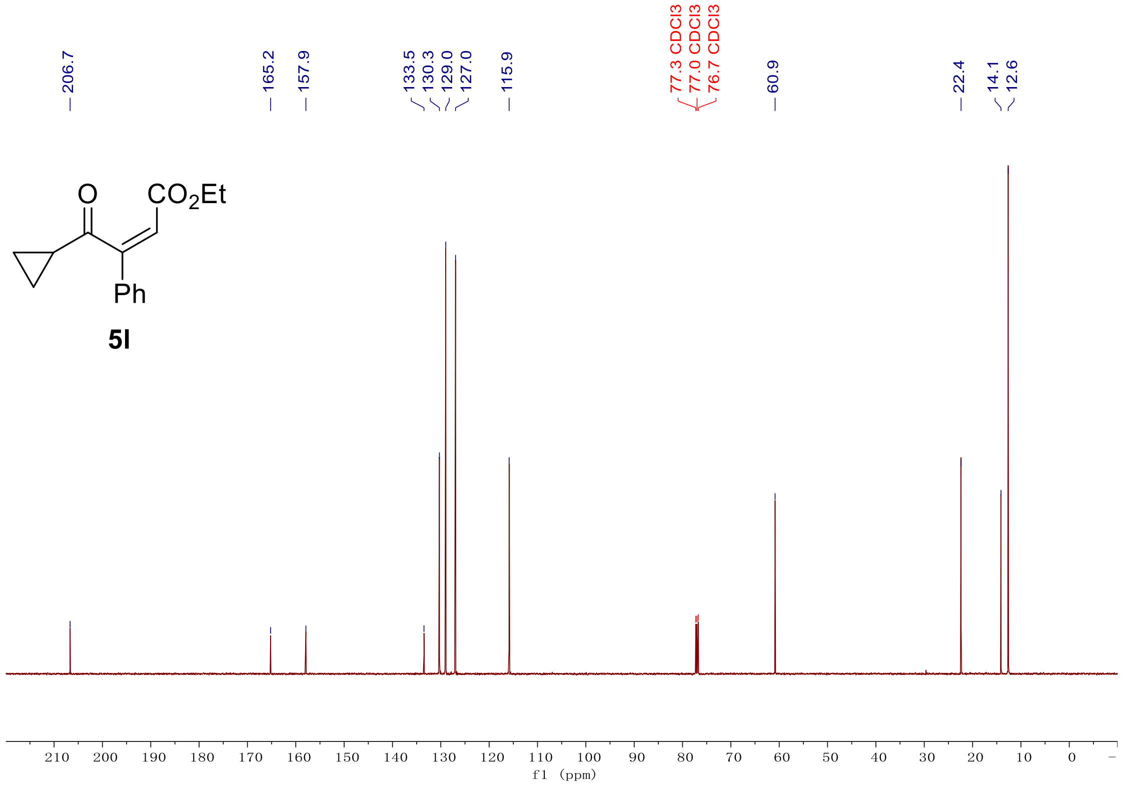

Supplementary Figure 193. ^{13}C NMR (125 MHz, CDCl3) spectrum for compound **5l**

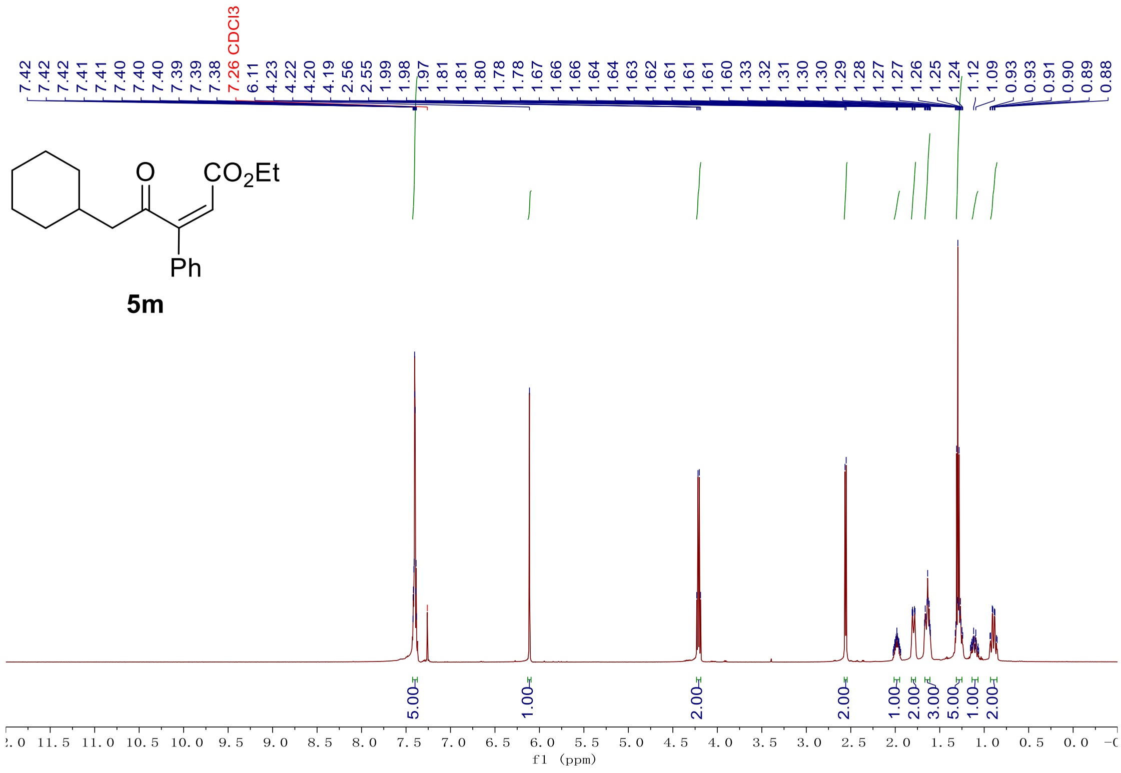

Supplementary Figure 194. ^{1}H NMR (500 MHz, $CDCl_3$) spectrum for compound **5m**

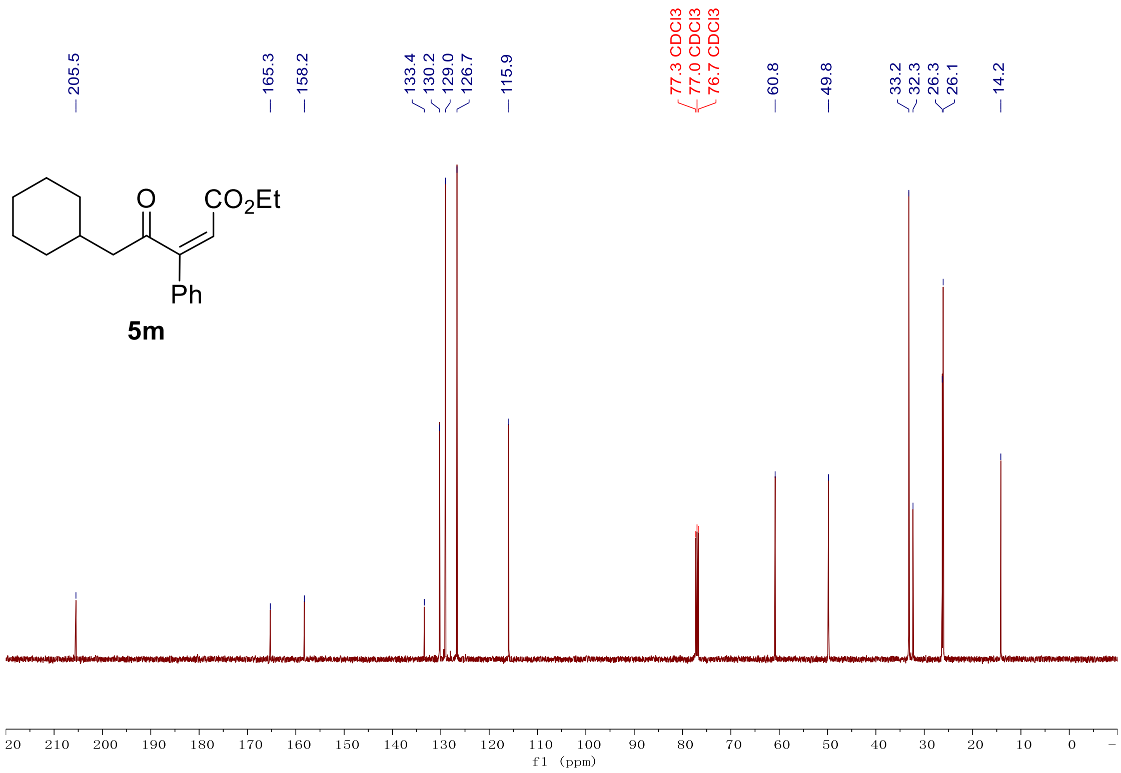

Supplementary Figure 195. ^{13}C NMR (125 MHz, $CDCl_3$) spectrum for compound **5m**

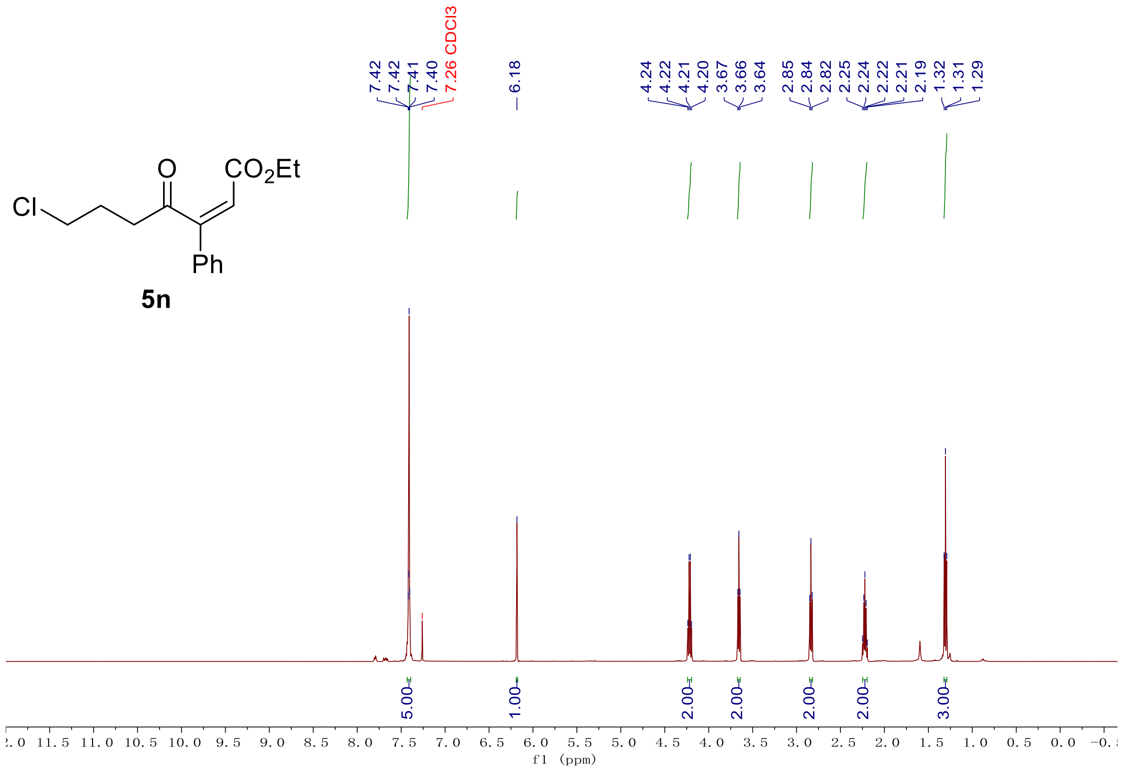

Supplementary Figure 196. ^{1}H NMR (500 MHz, $CDCl_3$) spectrum for compound **5n**

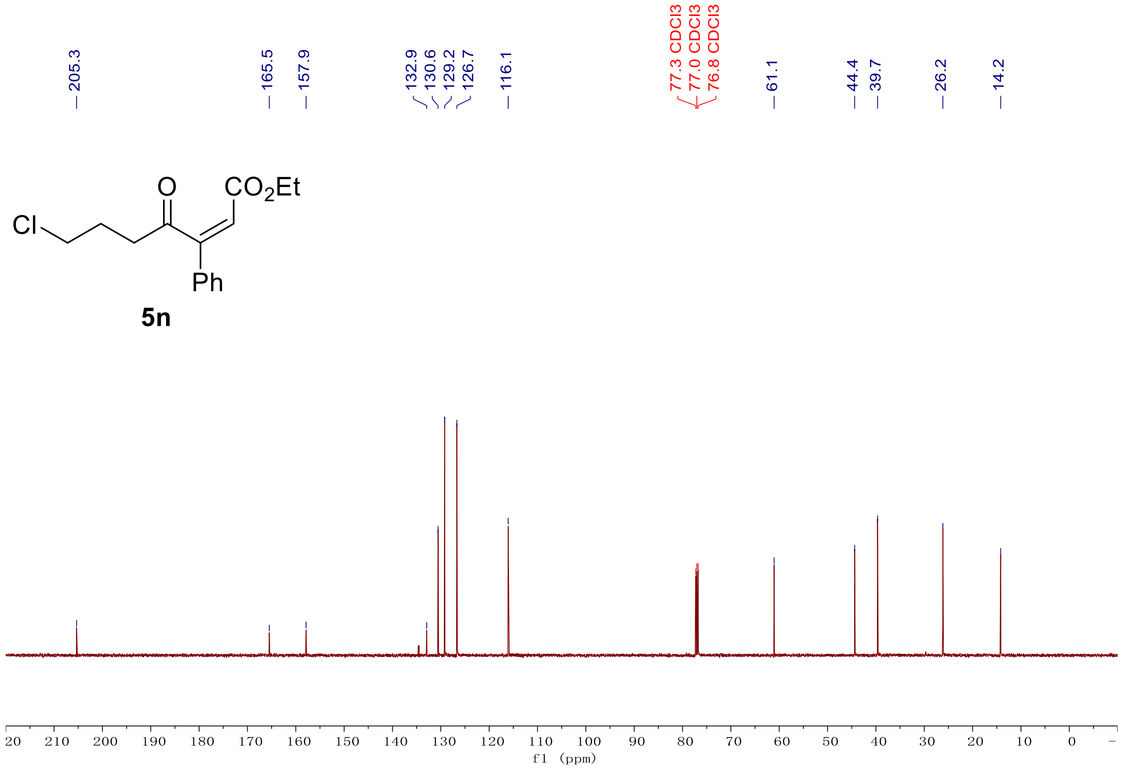

Supplementary Figure 197. ^{13}C NMR (125 MHz, $CDCl_3$) spectrum for compound **5n**

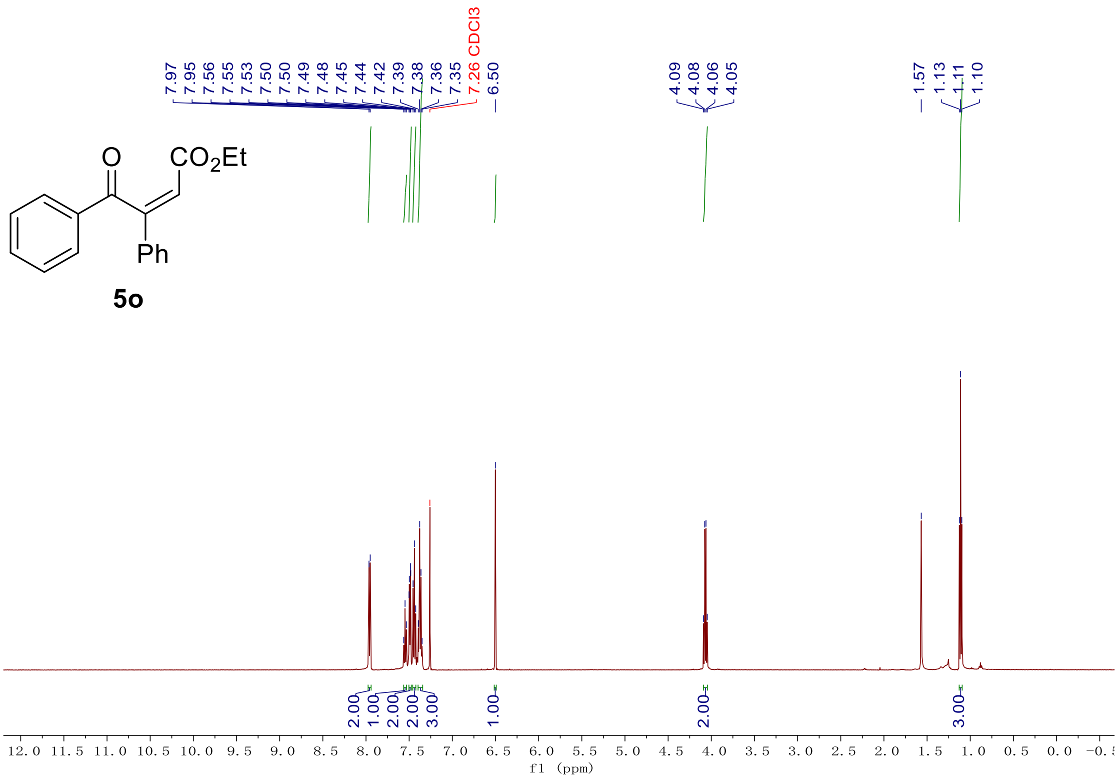

Supplementary Figure 198. ^{1}H NMR (500 MHz, $CDCl_3$) spectrum for compound **5o**

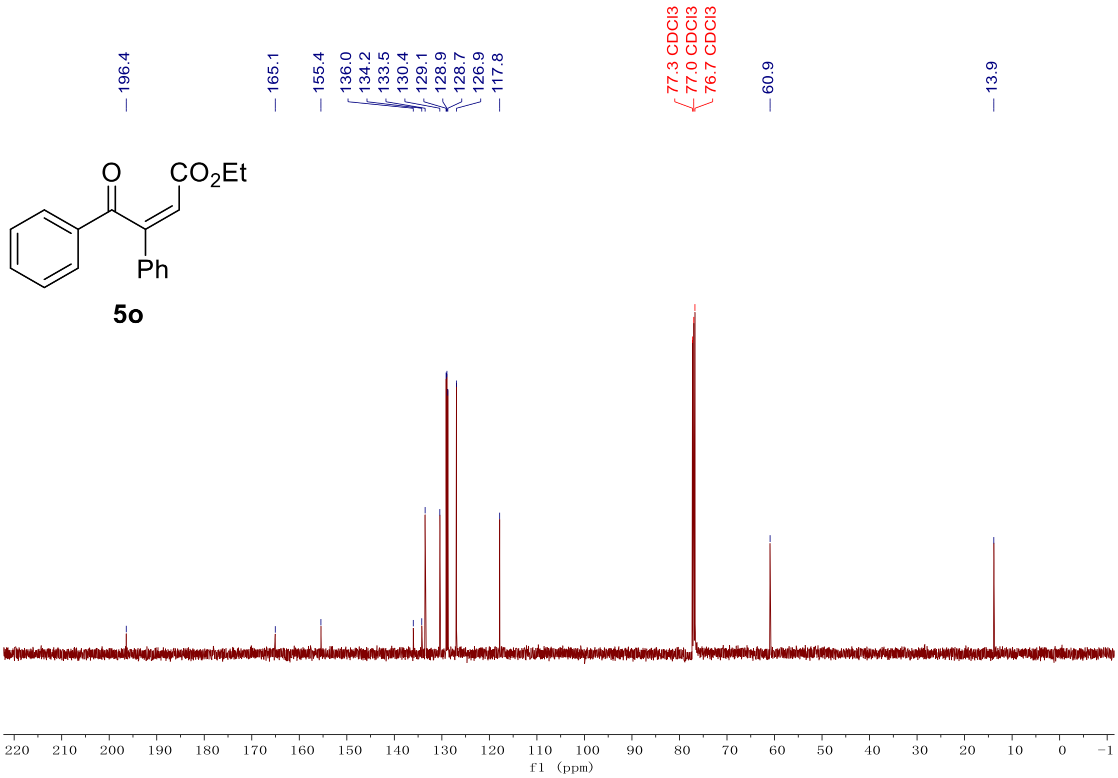

Supplementary Figure 199. ^{13}C NMR (125 MHz, $CDCl_3$) spectrum for compound **5o**

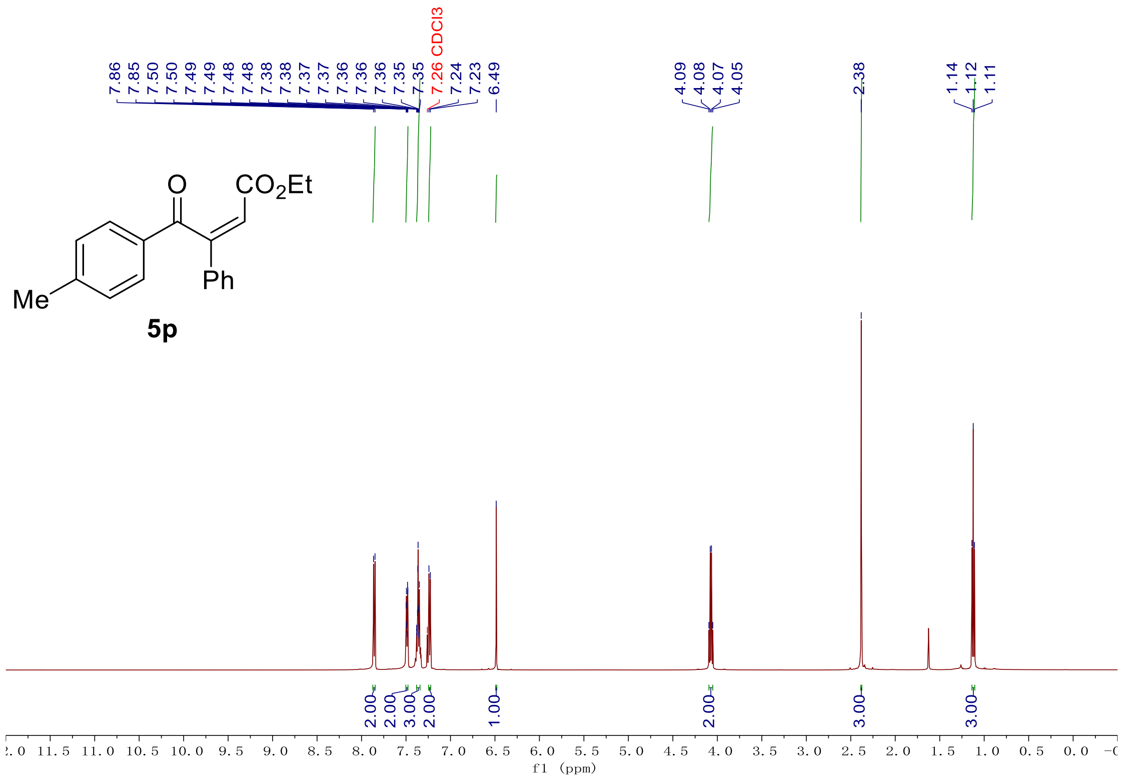

Supplementary Figure 200. ^{1}H NMR (500 MHz, $CDCl_3$) spectrum for compound **5p**

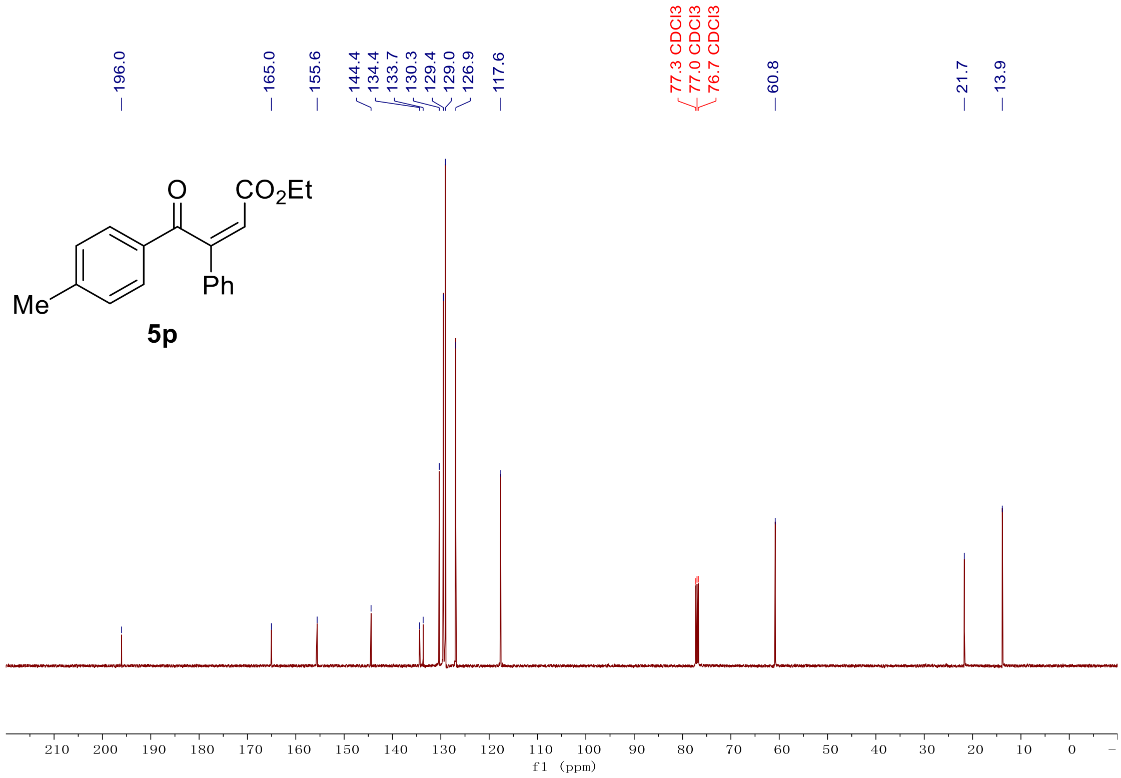

Supplementary Figure 201. ^{13}C NMR (125 MHz, $CDCl_3$) spectrum for compound **5p**

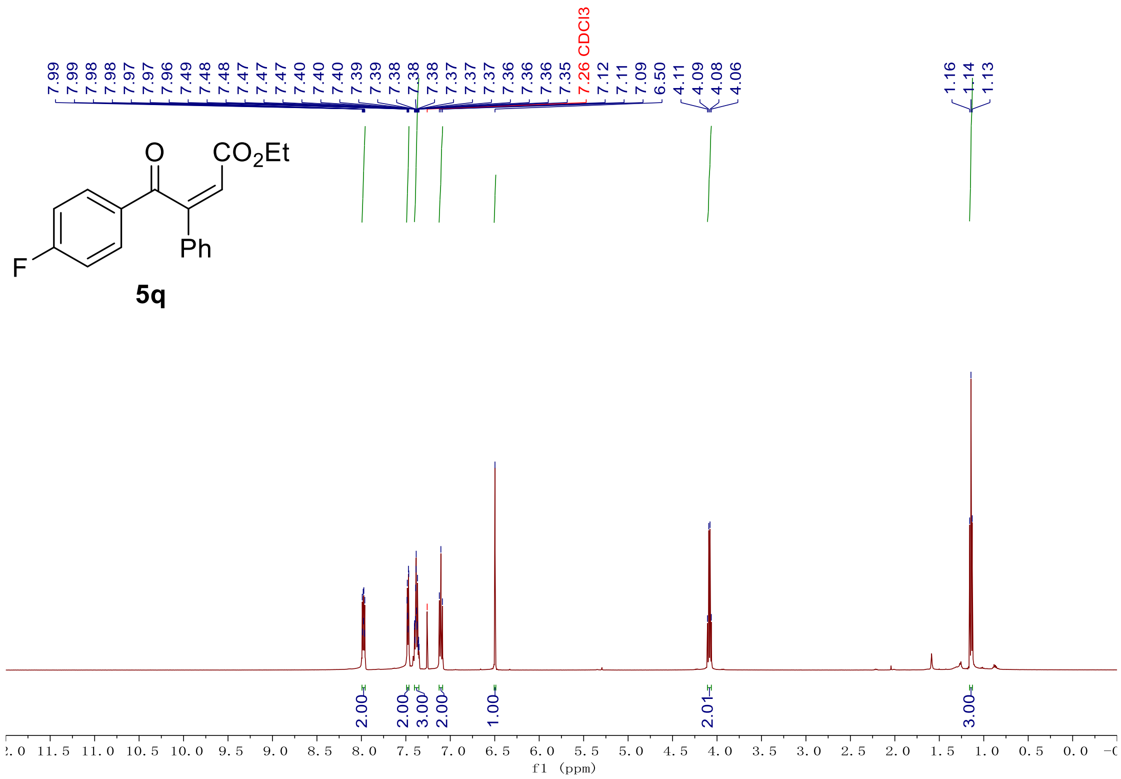

Supplementary Figure 202. ^{1}H NMR (500 MHz, $CDCl_3$) spectrum for compound **5q**

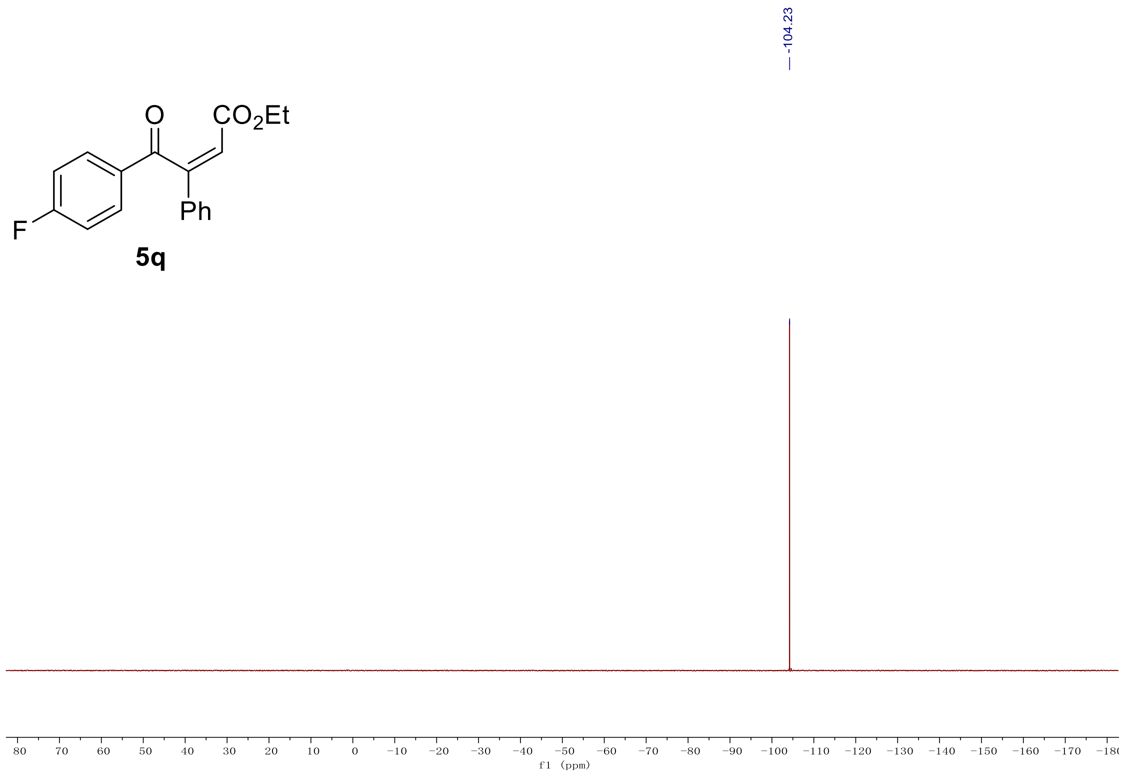

Supplementary Figure 203. ^{19}F NMR (471 MHz, $CDCl_3$) spectrum for compound **5q**

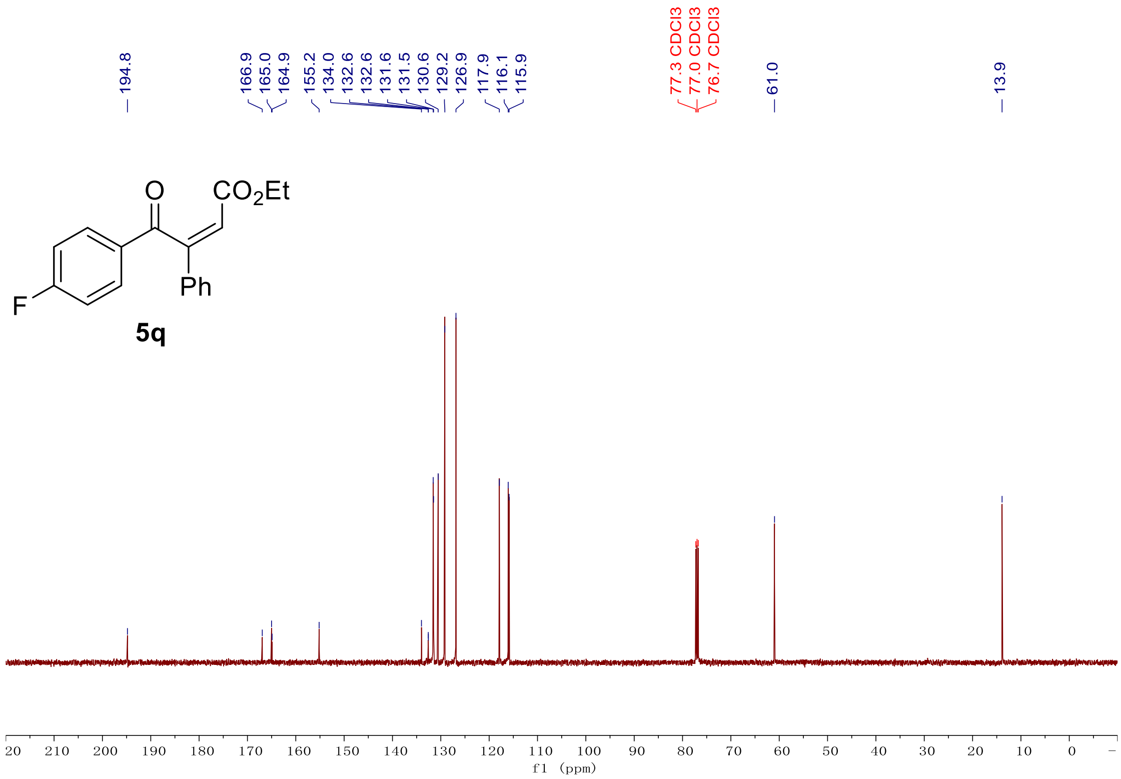

Supplementary Figure 204. ^{13}C NMR (125 MHz, CDCl$_3$) spectrum for compound **5q**

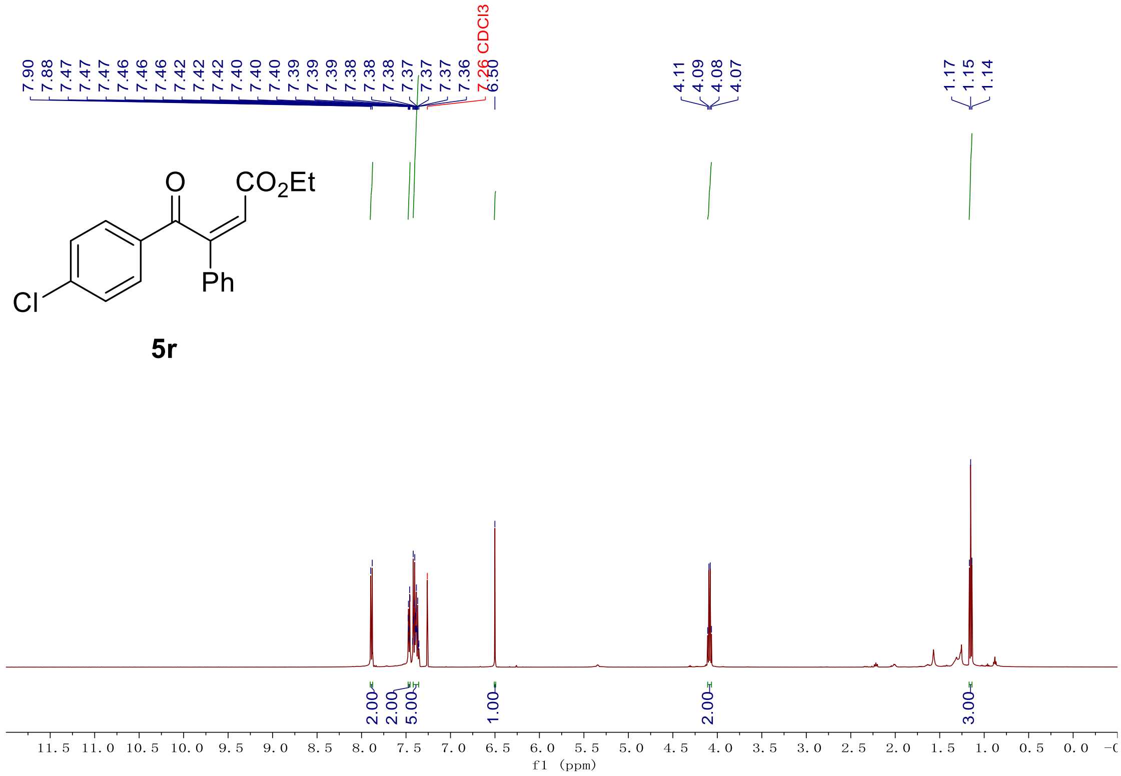

Supplementary Figure 205. ^{1}H NMR (500 MHz, CDCl$_3$) spectrum for compound **5r**

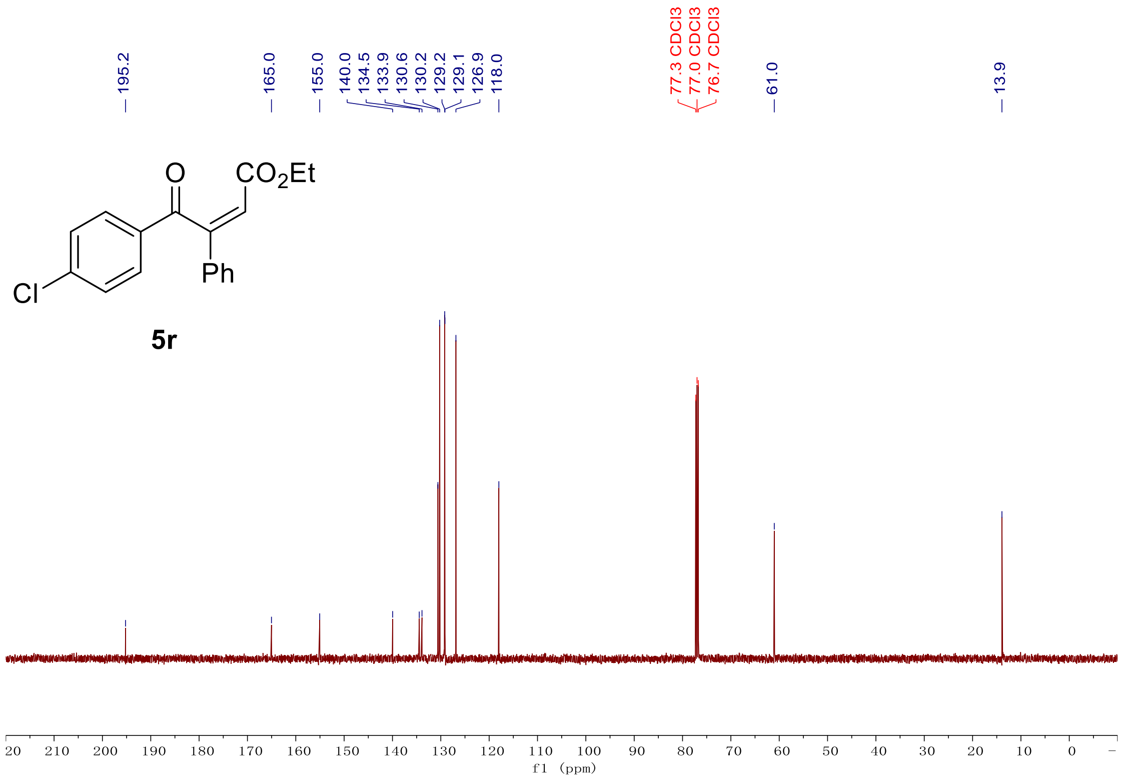

Supplementary Figure 206. ^{13}C NMR (125 MHz, CDCl3) spectrum for compound **5r**

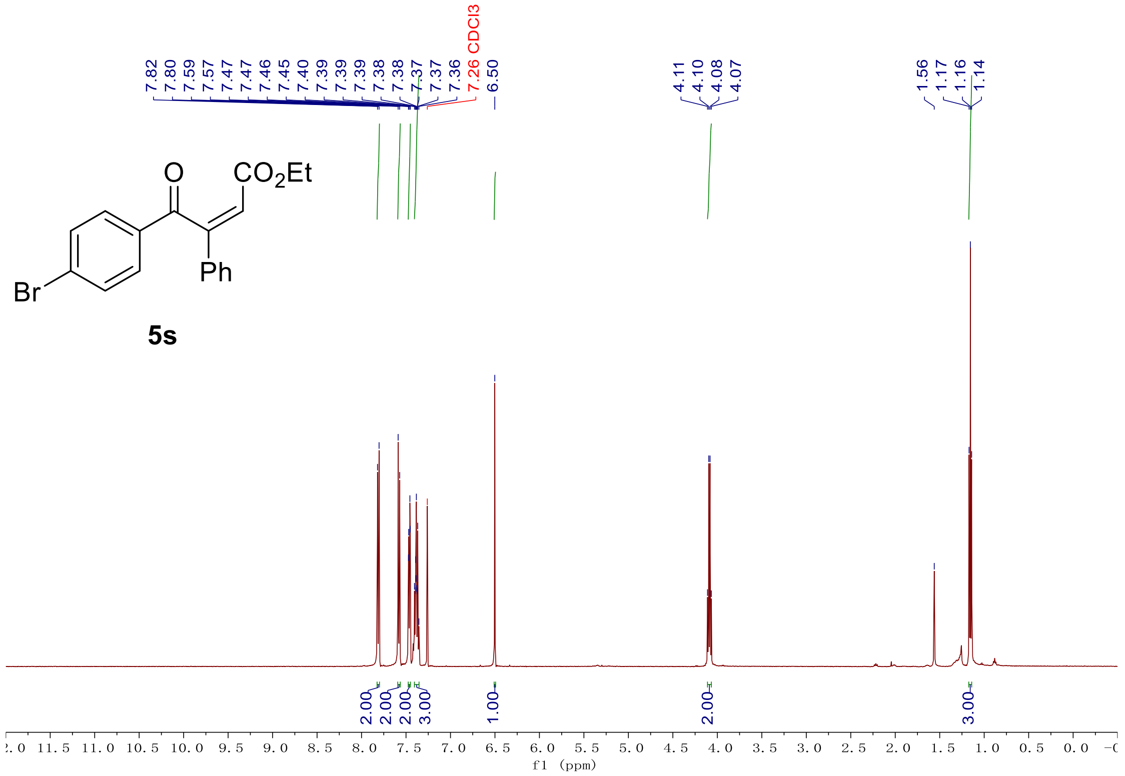

Supplementary Figure 207. ^{1}H NMR (500 MHz, CDCl3) spectrum for compound **5s**

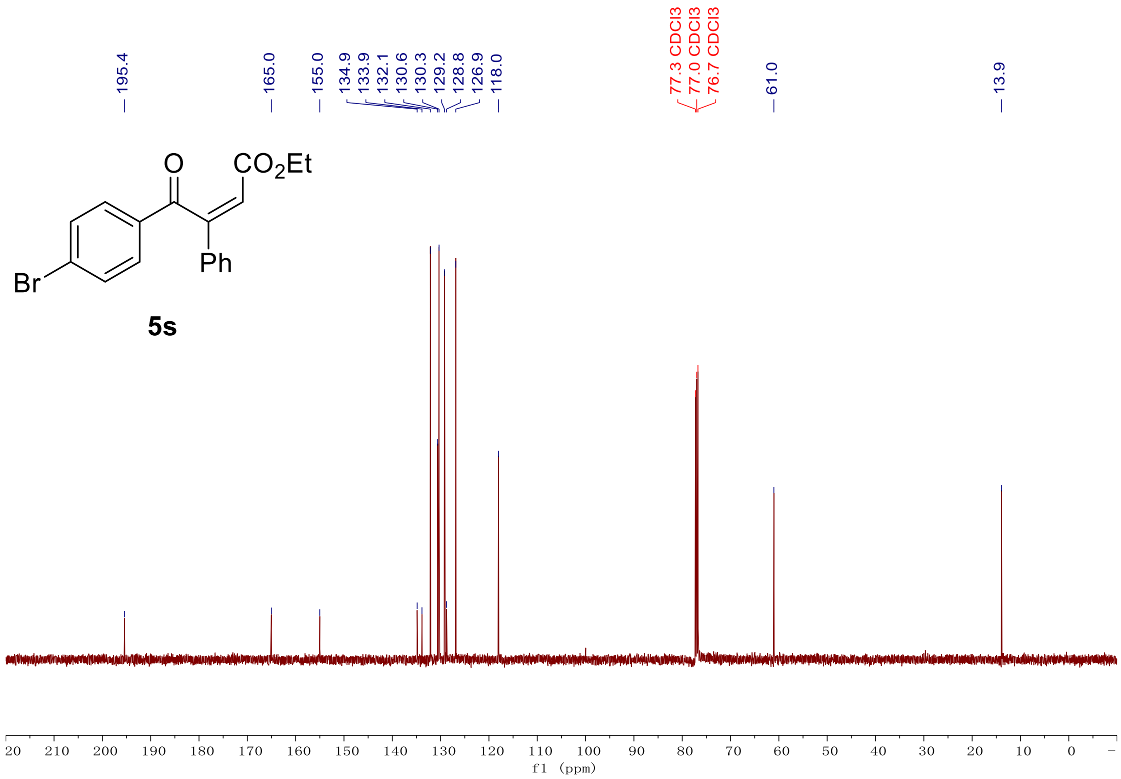

Supplementary Figure 208. ^{13}C NMR (125 MHz, CDCl$_3$) spectrum for compound **5s**

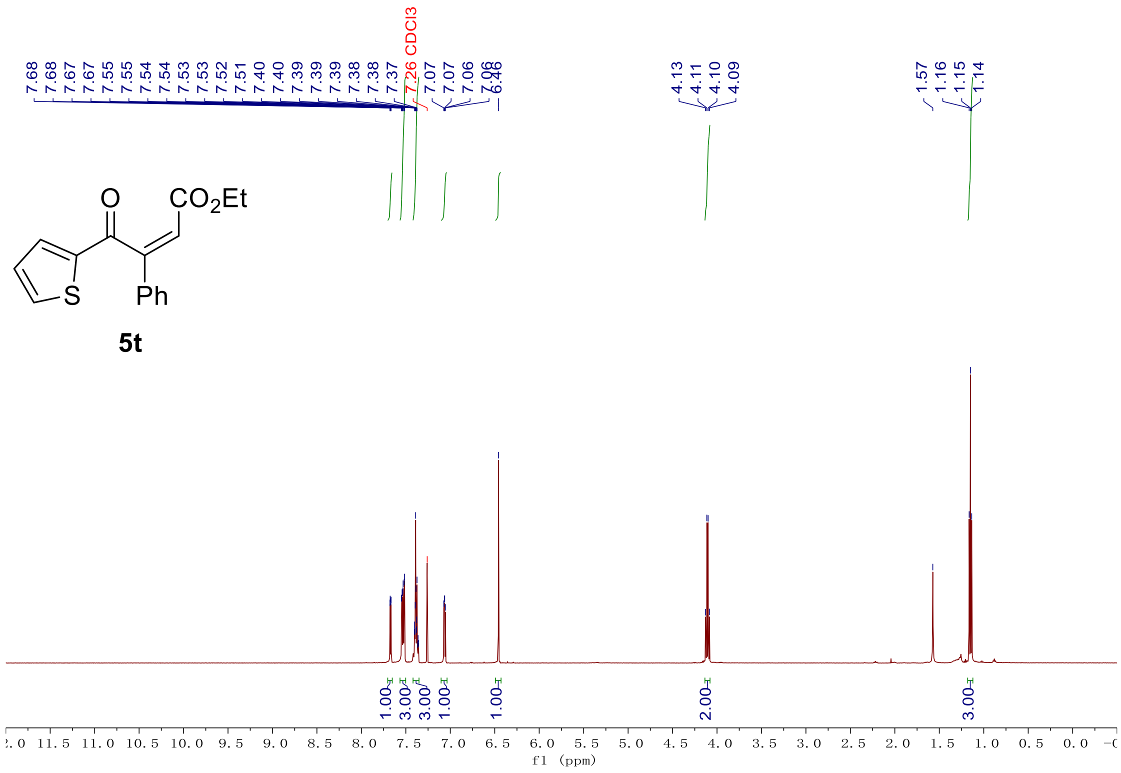

Supplementary Figure 209. ^{1}H NMR (500 MHz, CDCl$_3$) spectrum for compound **5t**

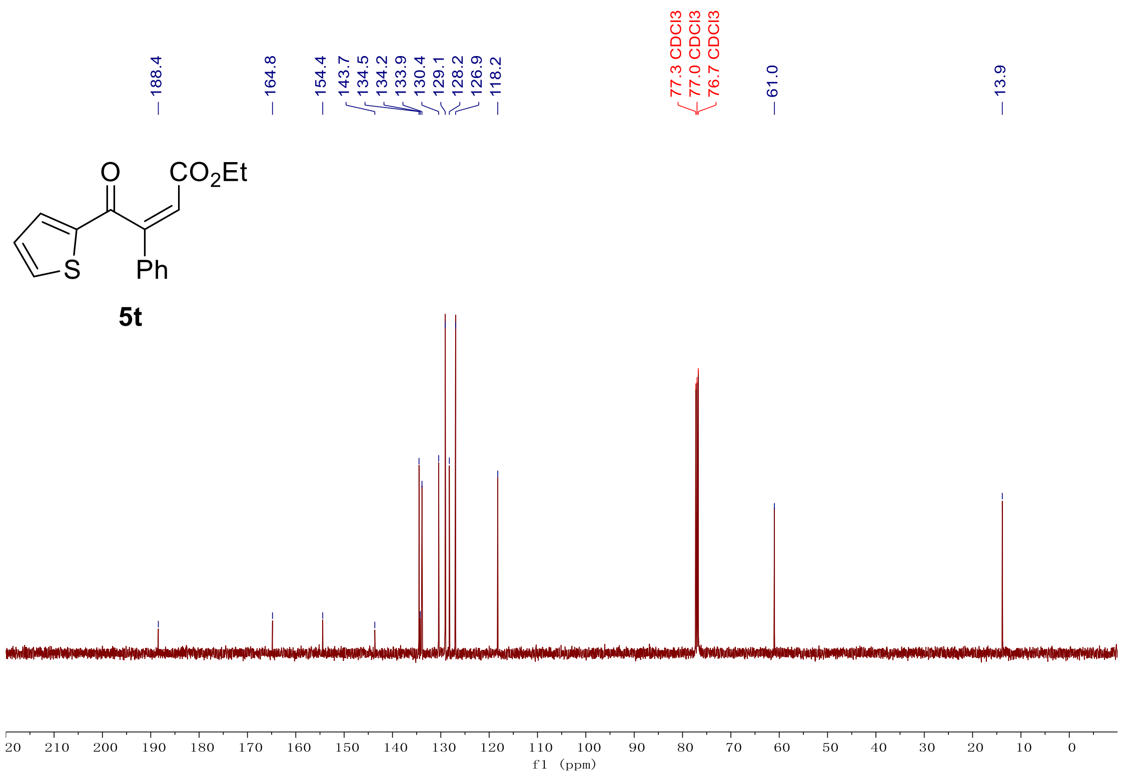

Supplementary Figure 210. ^{13}C NMR (125 MHz, $CDCl_3$) spectrum for compound **5t**

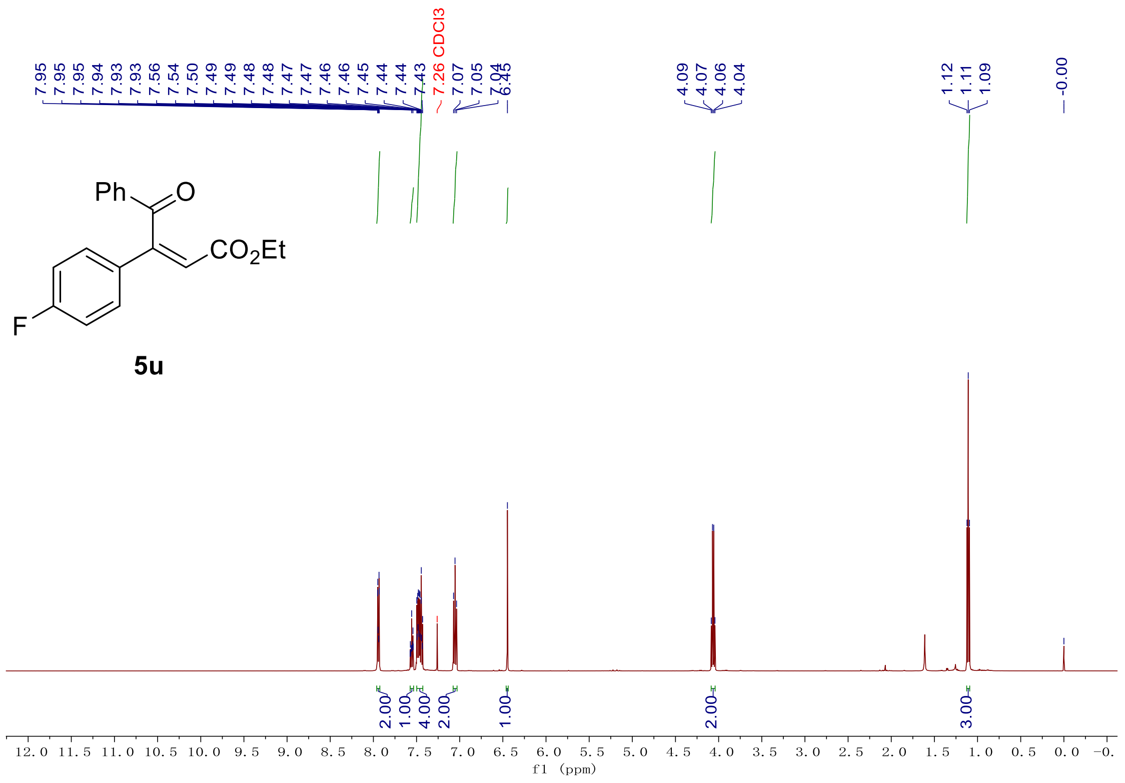

Supplementary Figure 211. ^{1}H NMR (500 MHz, $CDCl_3$) spectrum for compound **5u**

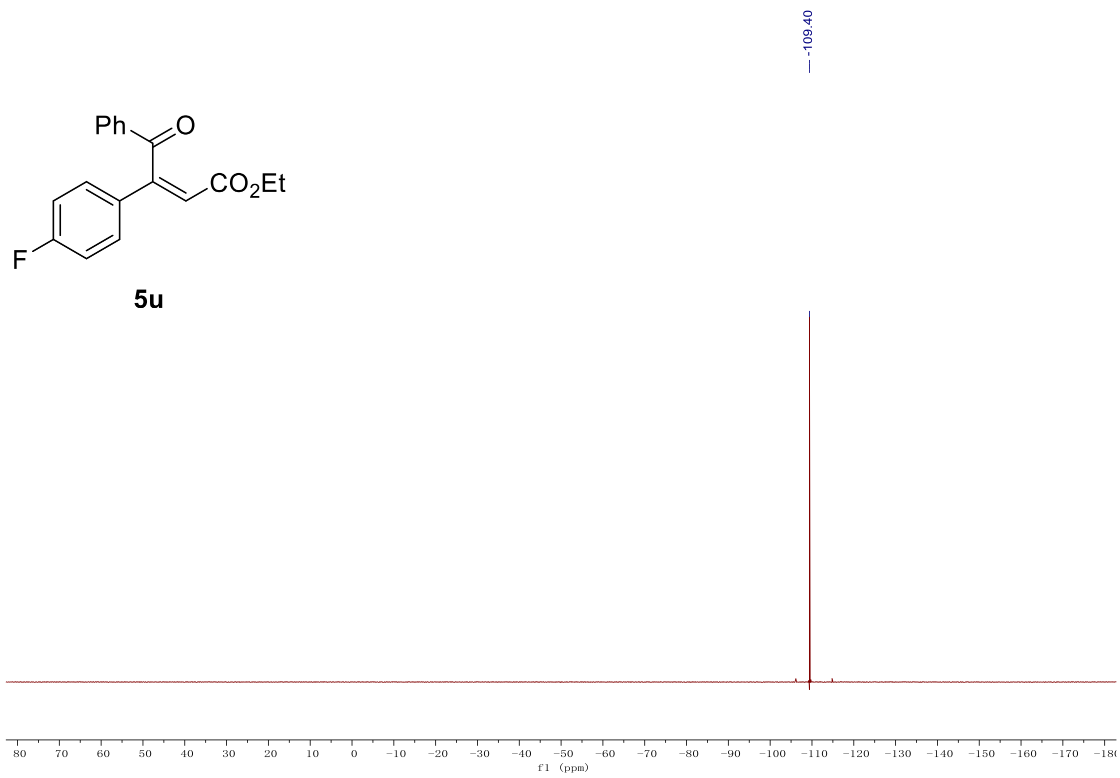

Supplementary Figure 212. ^{19}F NMR (471 MHz, CDCl3) spectrum for compound **5u**

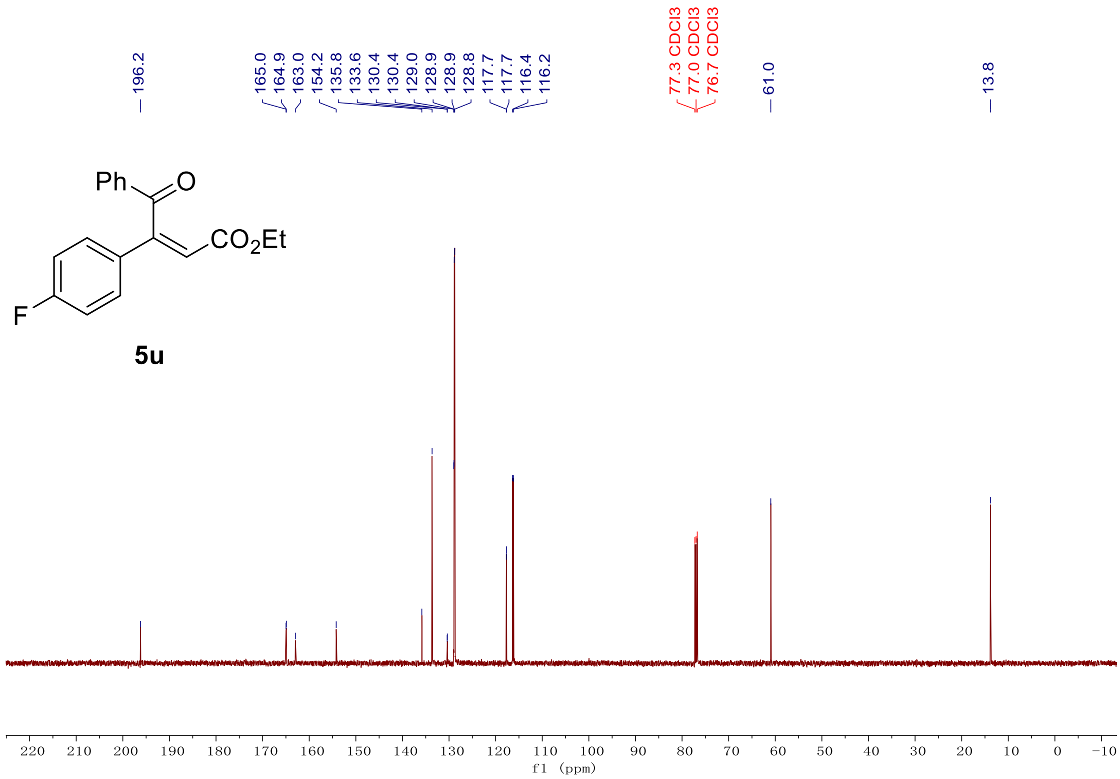

Supplementary Figure 213. ^{13}C NMR (125 MHz, CDCl3) spectrum for compound **5u**

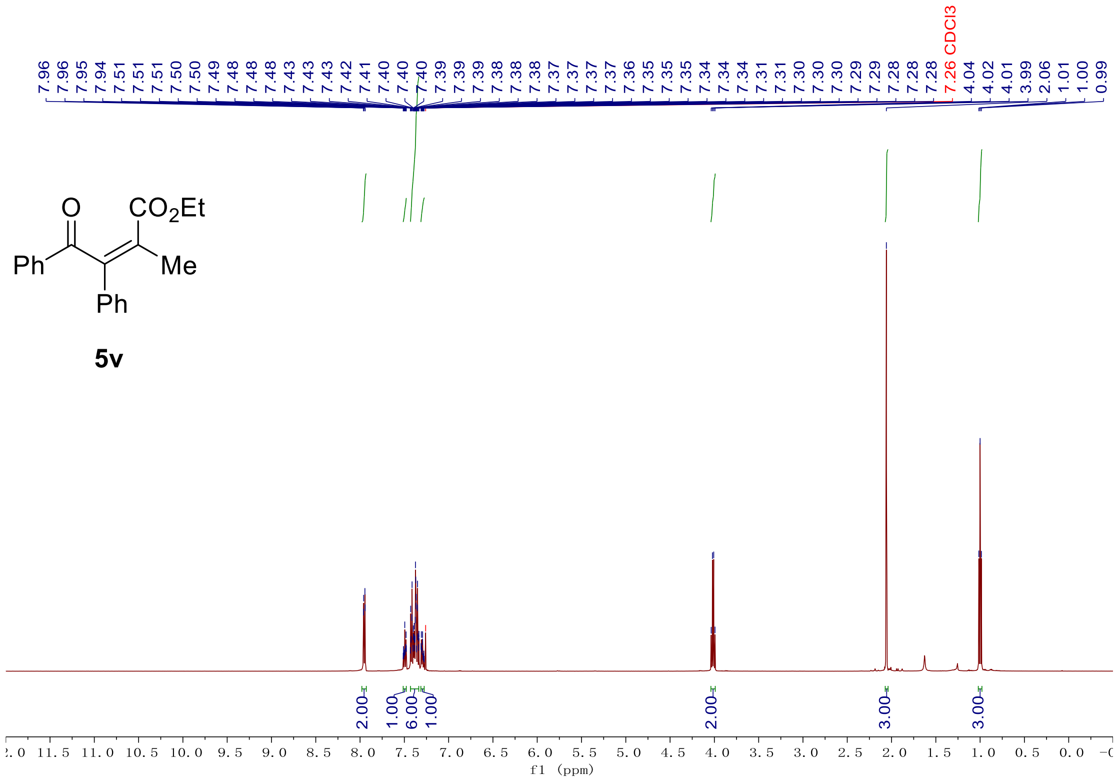

Supplementary Figure 214. ^{1}H NMR (500 MHz, $CDCl_3$) spectrum for compound **5v**

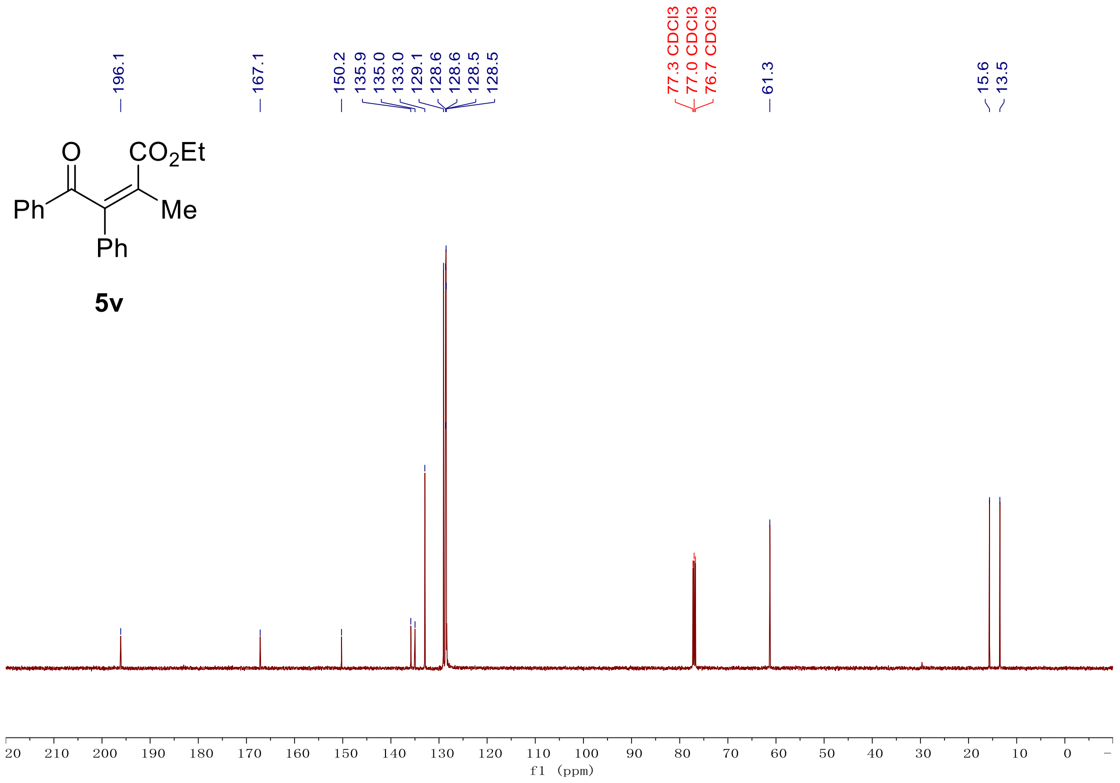

Supplementary Figure 215. ^{13}C NMR (125 MHz, $CDCl_3$) spectrum for compound **5v**

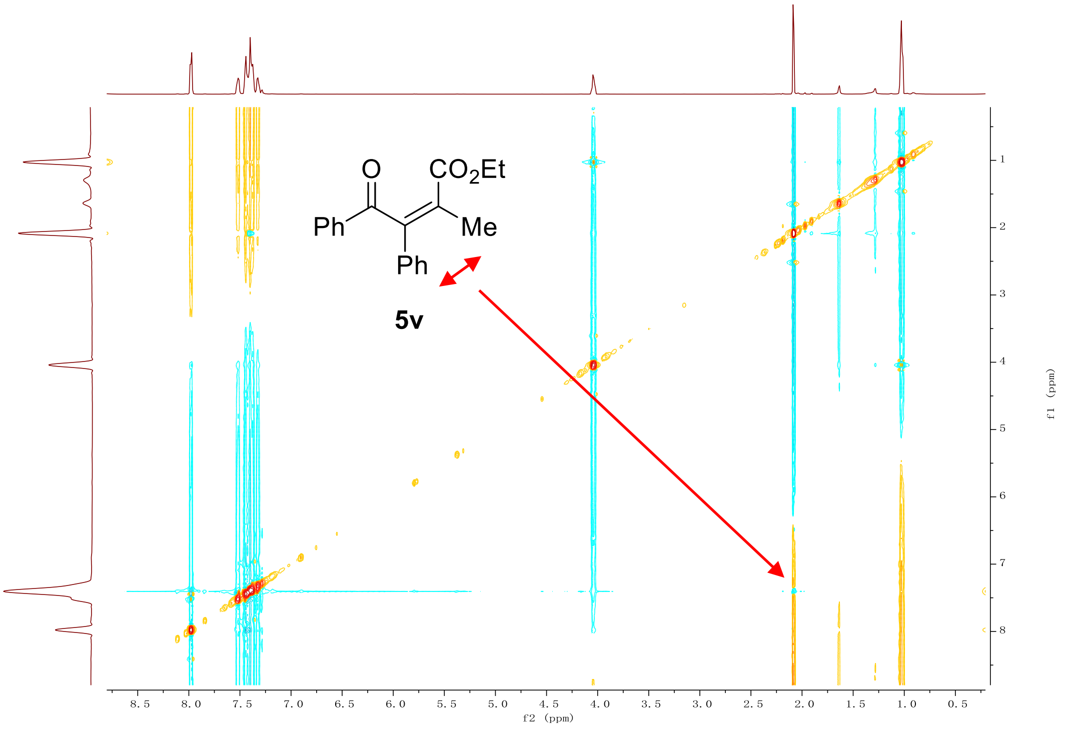

Supplementary Figure 216. ^1H-^1H, NOESY NMR (600 MHz, $CDCl_3$) spectrum for compound **5v**

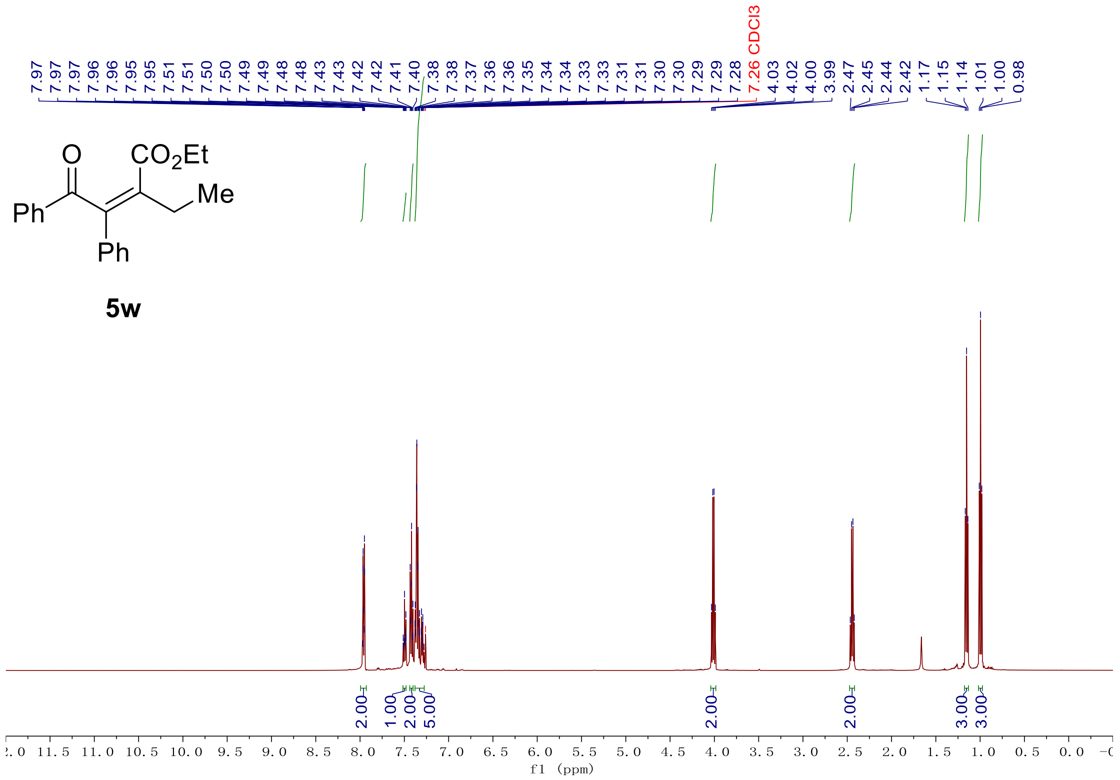

Supplementary Figure 217. ^1H NMR (500 MHz, $CDCl_3$) spectrum for compound **5w**

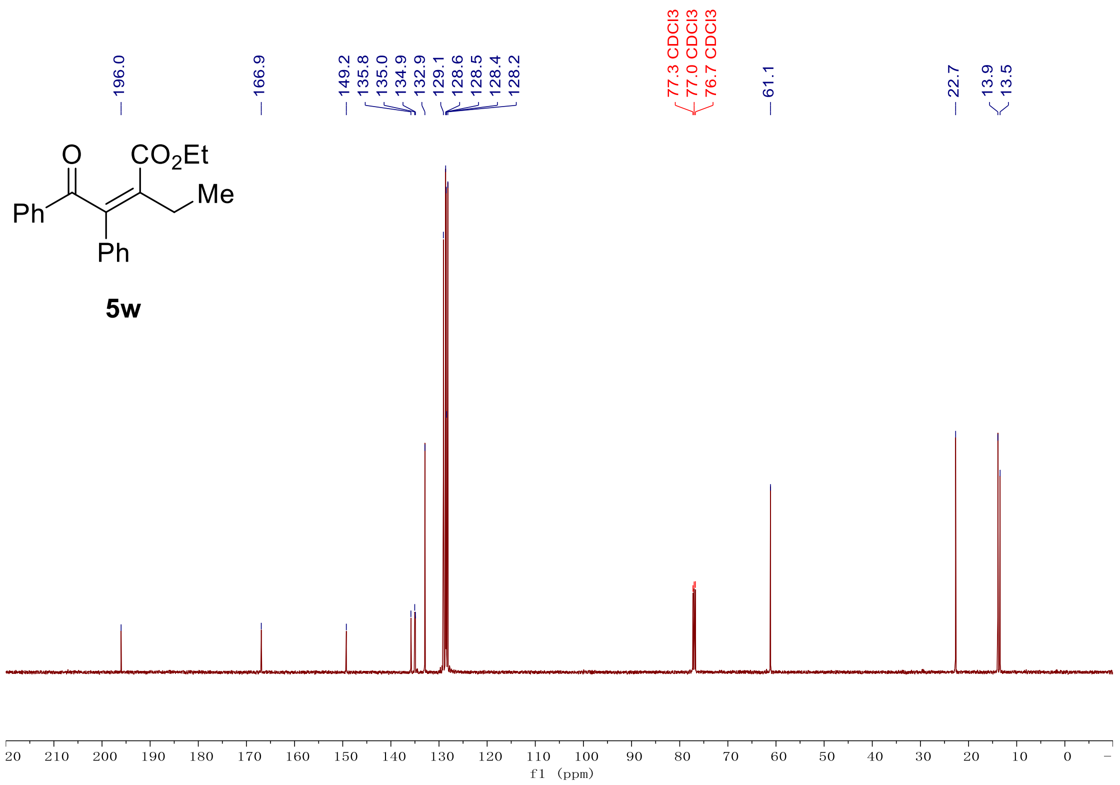

Supplementary Figure 218. ^{13}C NMR (125 MHz, CDCl3) spectrum for compound **5w**

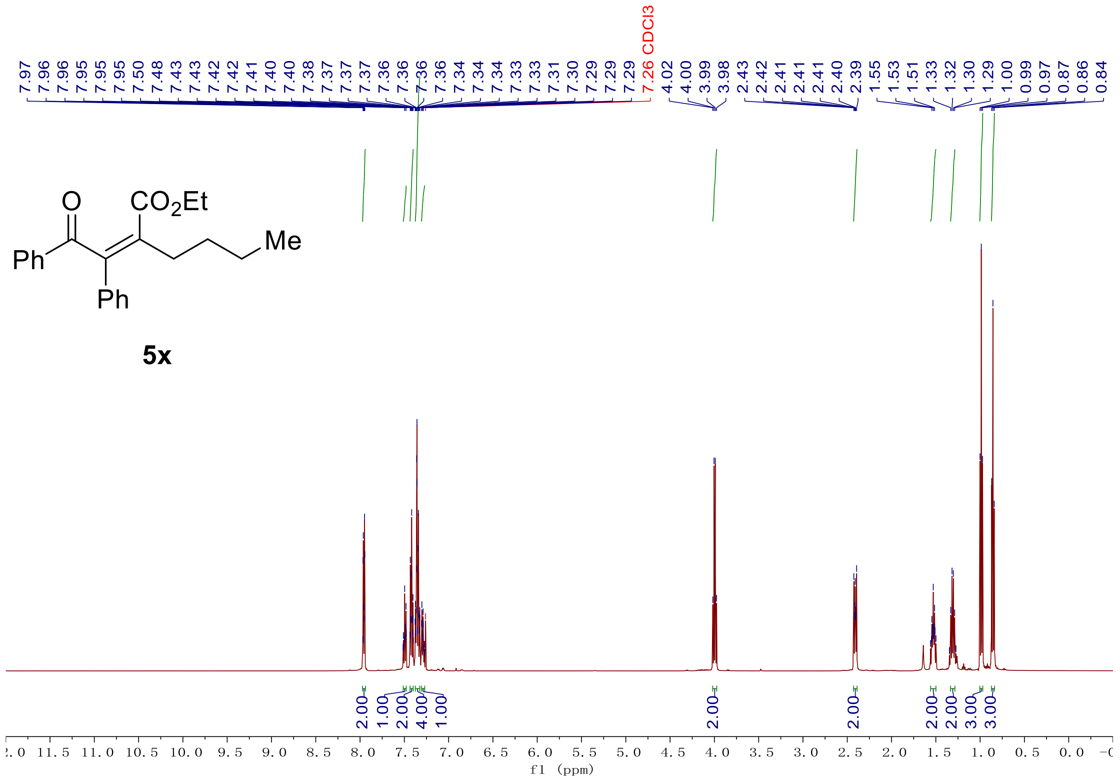

Supplementary Figure 219. ^{1}H NMR (500 MHz, CDCl3) spectrum for compound **5x**

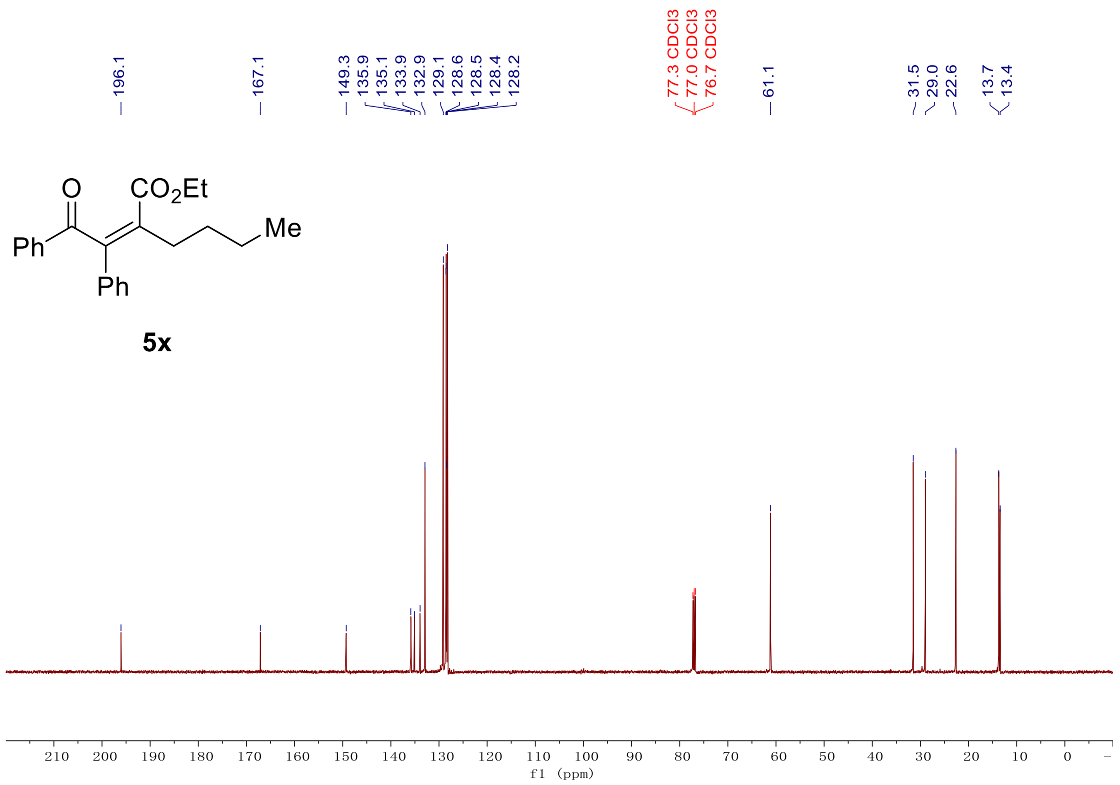

Supplementary Figure 220. ^{13}C NMR (125 MHz, $CDCl_3$) spectrum for compound **5x**

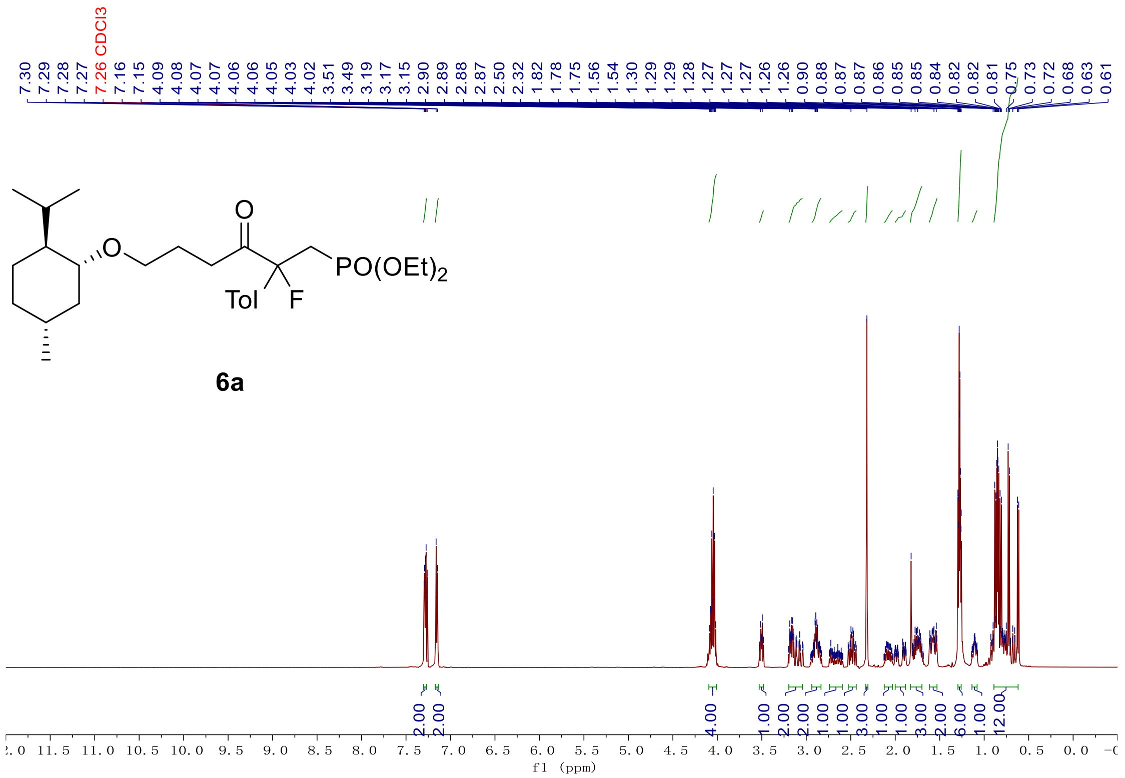

Supplementary Figure 221. ^{1}H NMR (500 MHz, $CDCl_3$) spectrum for compound **6a**

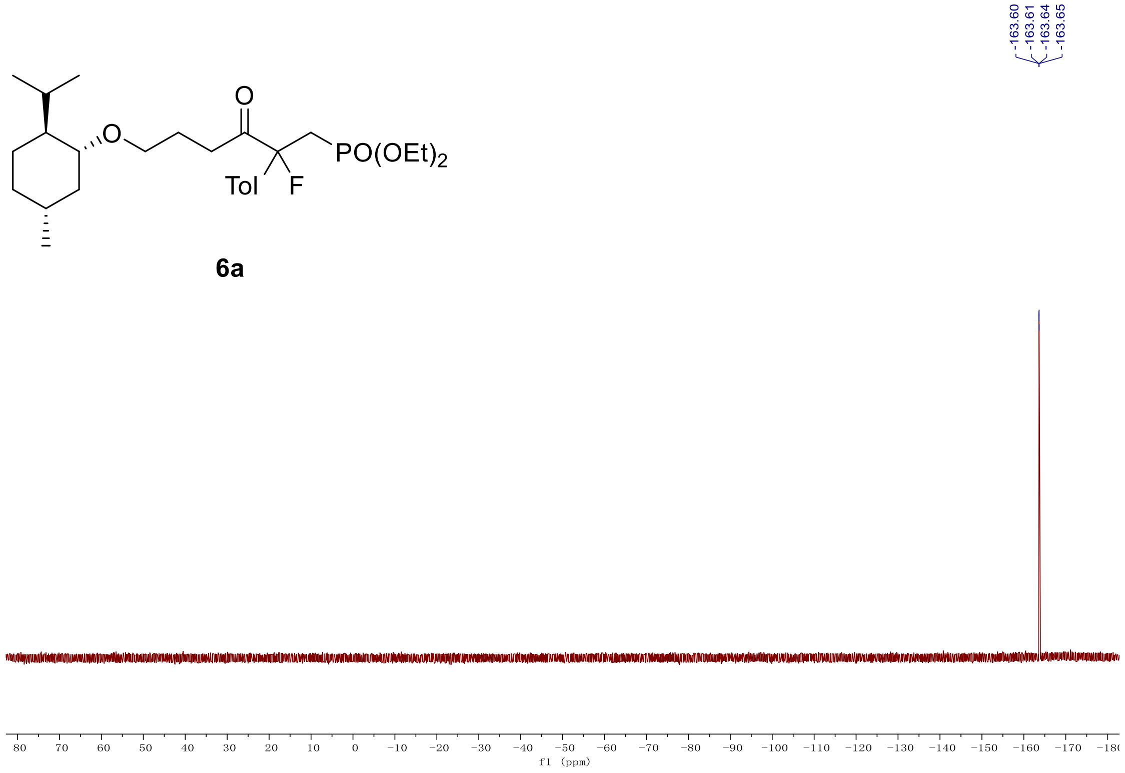

Supplementary Figure 222. ^{19}F NMR (471 MHz, $CDCl_3$) spectrum for compound **6a**

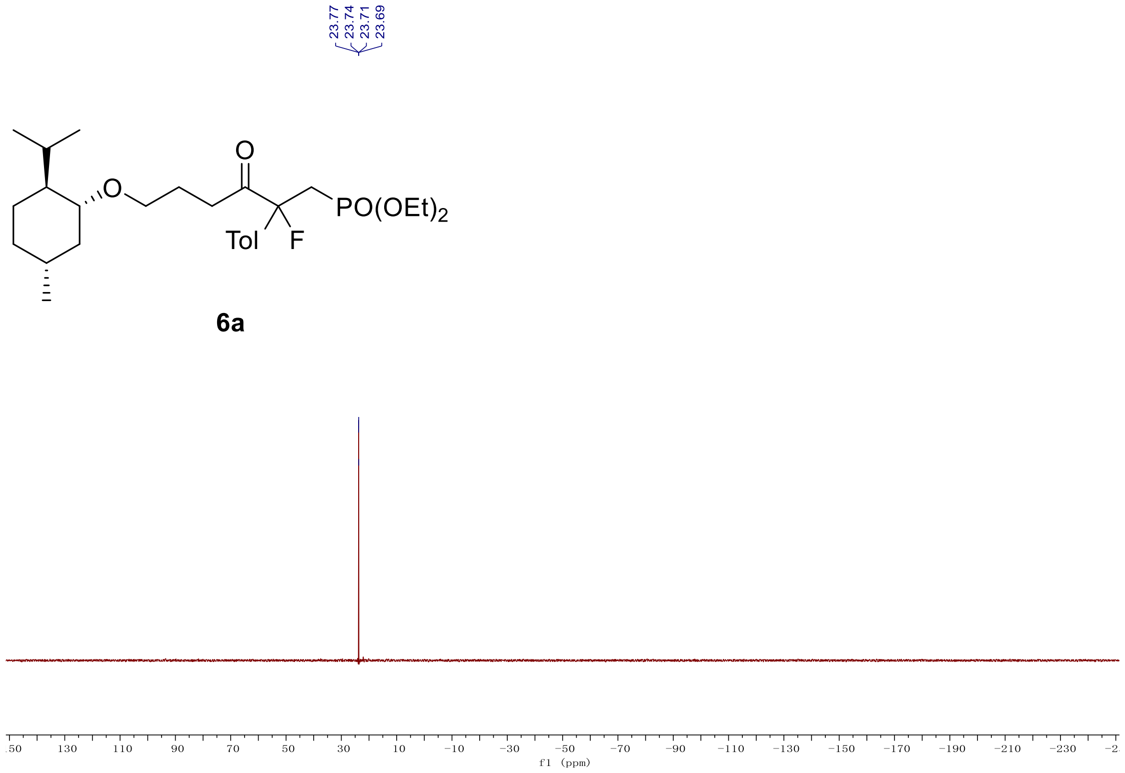

Supplementary Figure 223. ^{31}P NMR (202 MHz, $CDCl_3$) spectrum for compound **6a**

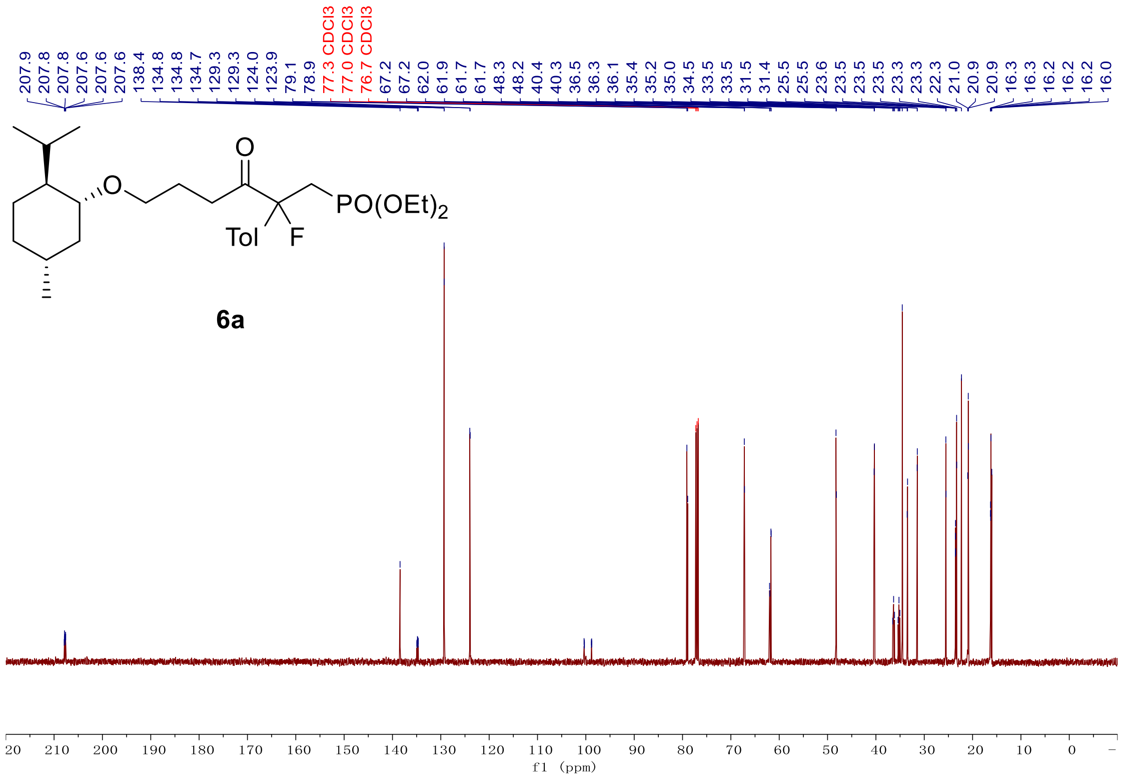

Supplementary Figure 224. ^{13}C NMR (125 MHz, $CDCl_3$) spectrum for compound **6a**

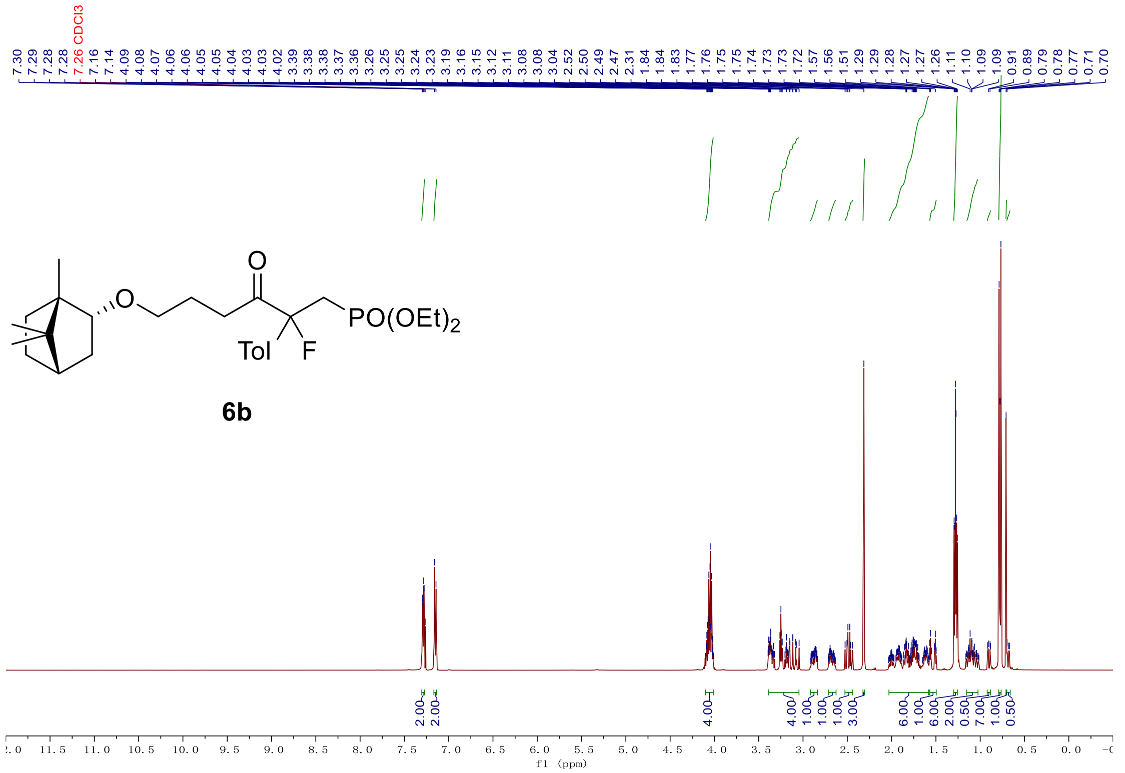

Supplementary Figure 225. ^{1}H NMR (500 MHz, $CDCl_3$) spectrum for compound **6b**

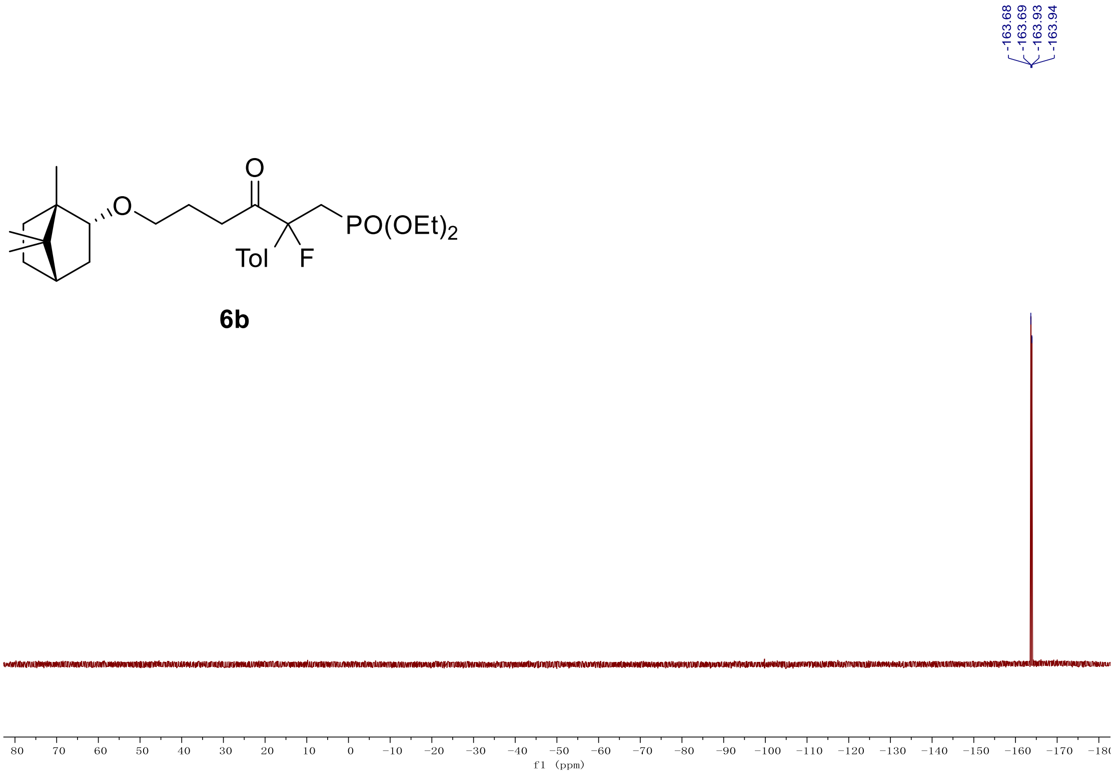

Supplementary Figure 226. ^{19}F NMR (471 MHz, $CDCl_3$) spectrum for compound **6b**

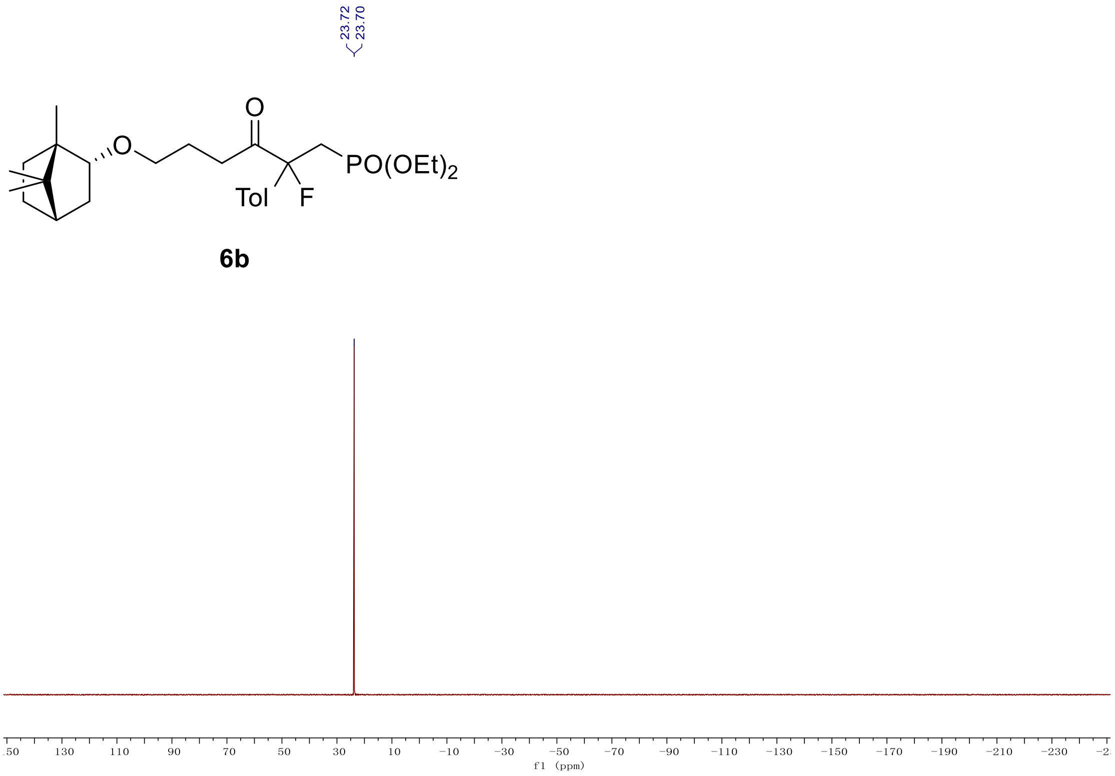

Supplementary Figure 227. ^{31}P NMR (202 MHz, $CDCl_3$) spectrum for compound **6b**

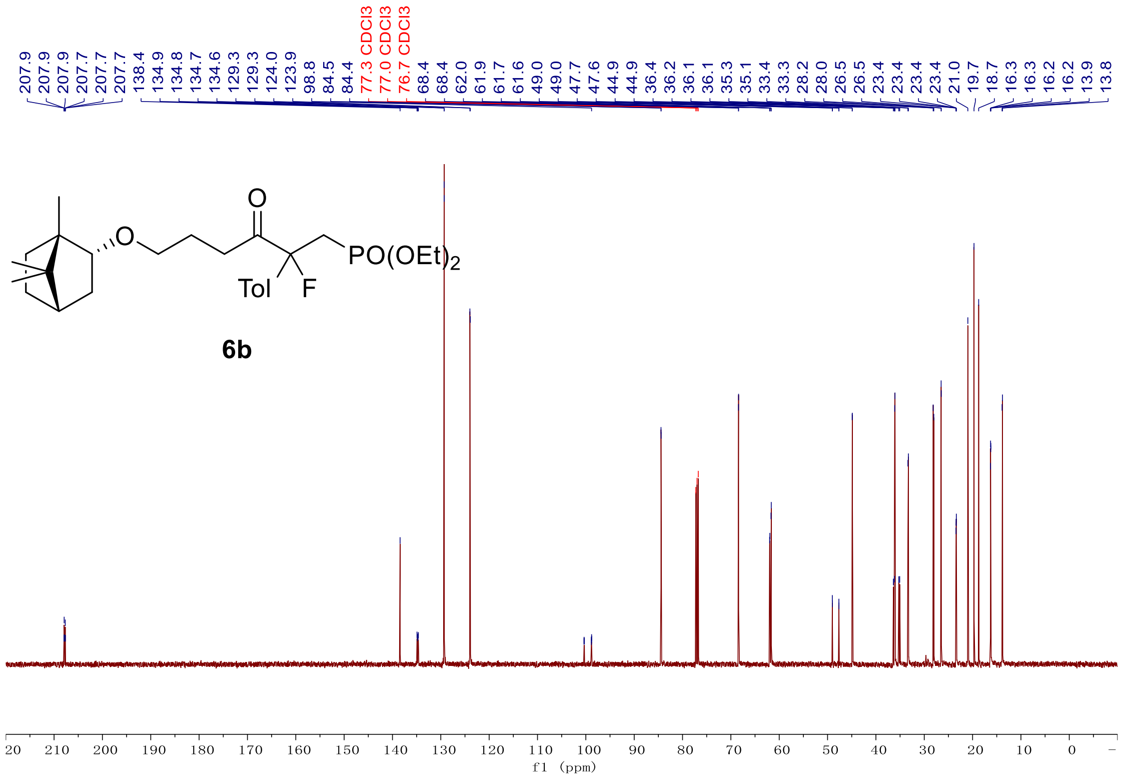

Supplementary Figure 228. ^{13}C NMR (125 MHz, $CDCl_3$) spectrum for compound **6b**

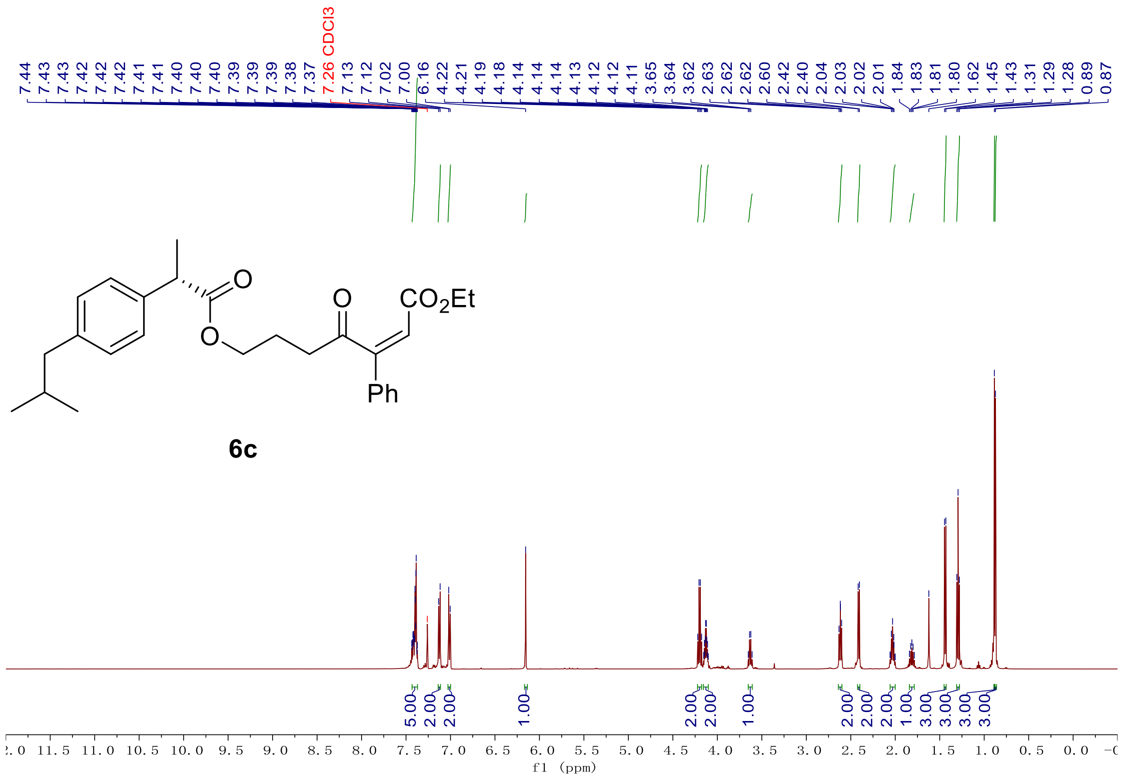

Supplementary Figure 229. ^{1}H NMR (500 MHz, $CDCl_3$) spectrum for compound **6c**

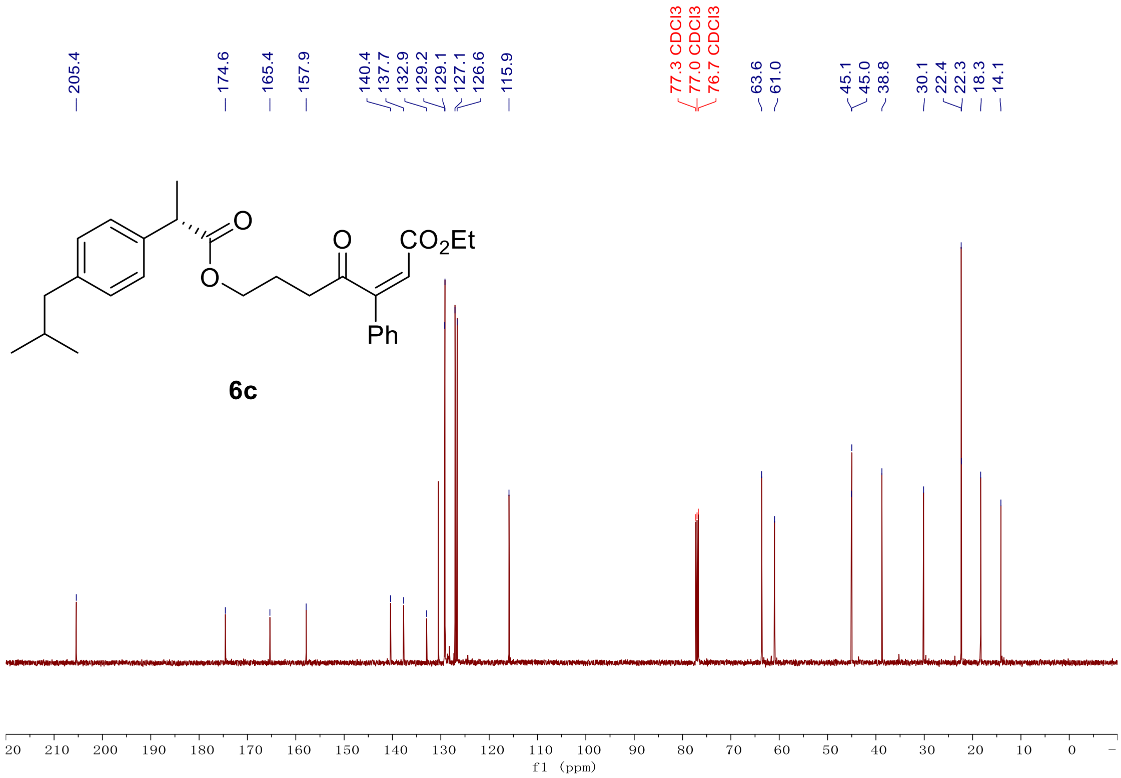

Supplementary Figure 230. ^{13}C NMR (125 MHz, $CDCl_3$) spectrum for compound **6c**

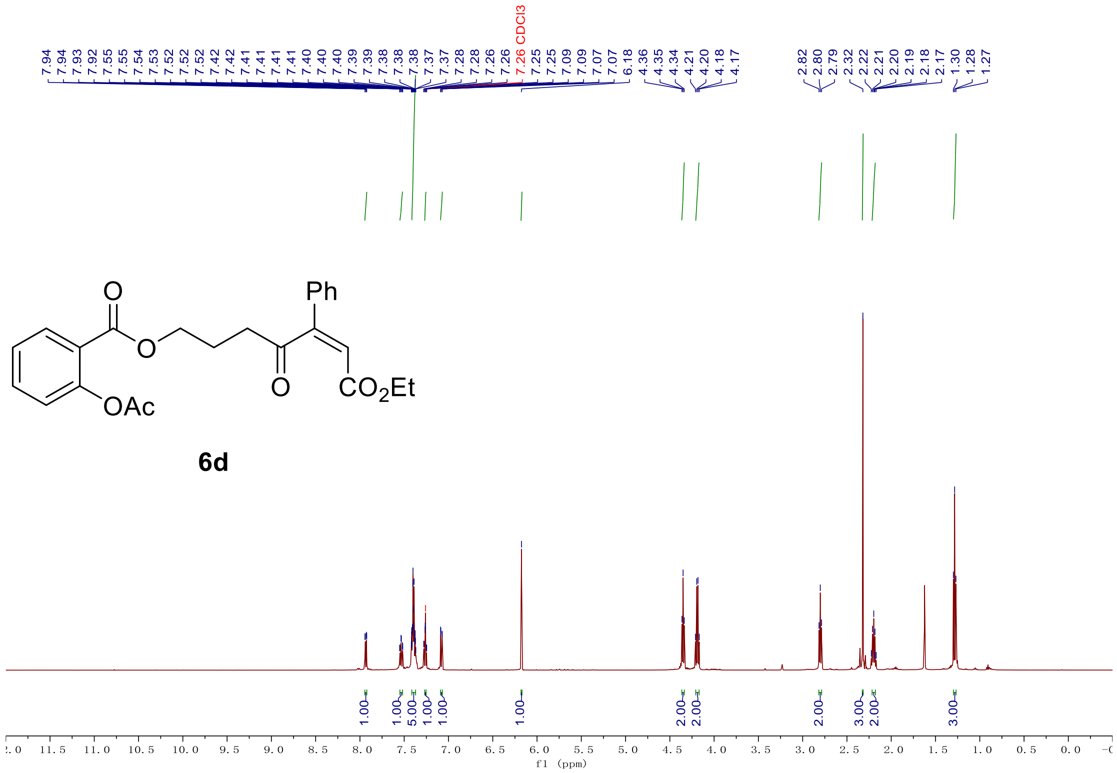

Supplementary Figure 231. ^{1}H NMR (500 MHz, $CDCl_3$) spectrum for compound **6d**

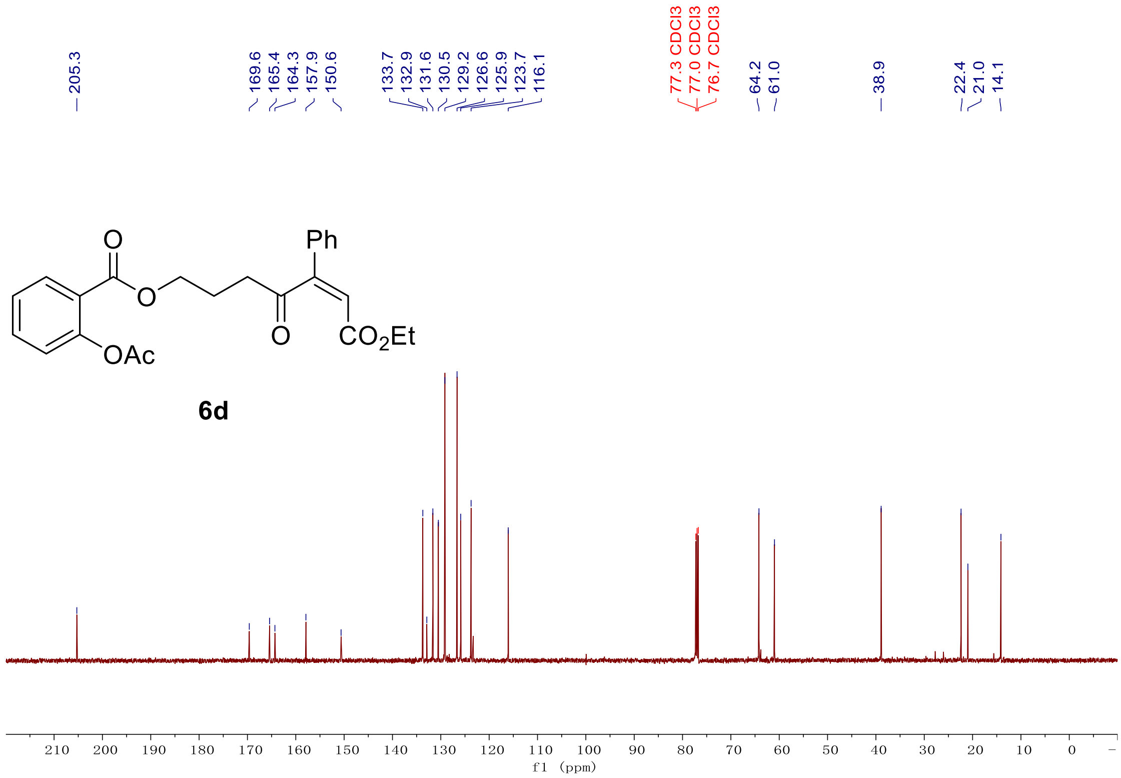

Supplementary Figure 232. ^{13}C NMR (125 MHz, $CDCl_3$) spectrum for compound **6d**

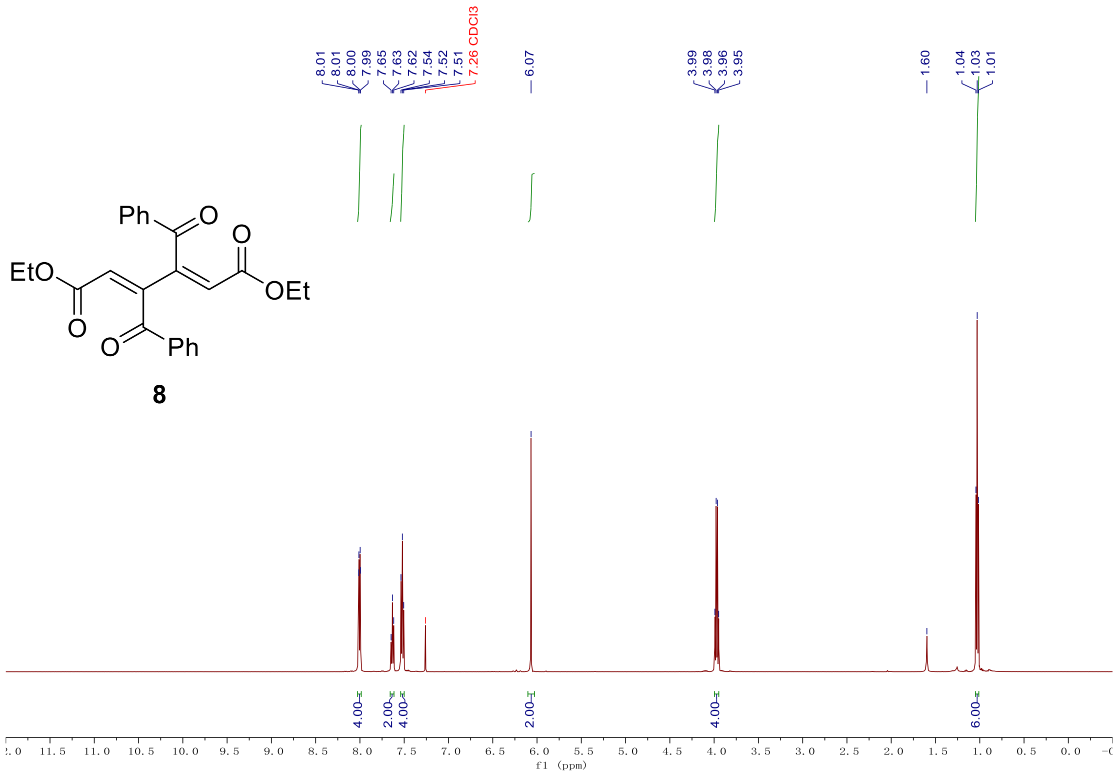

Supplementary Figure 233. ^{1}H NMR (500 MHz, $CDCl_3$) spectrum for compound **8**

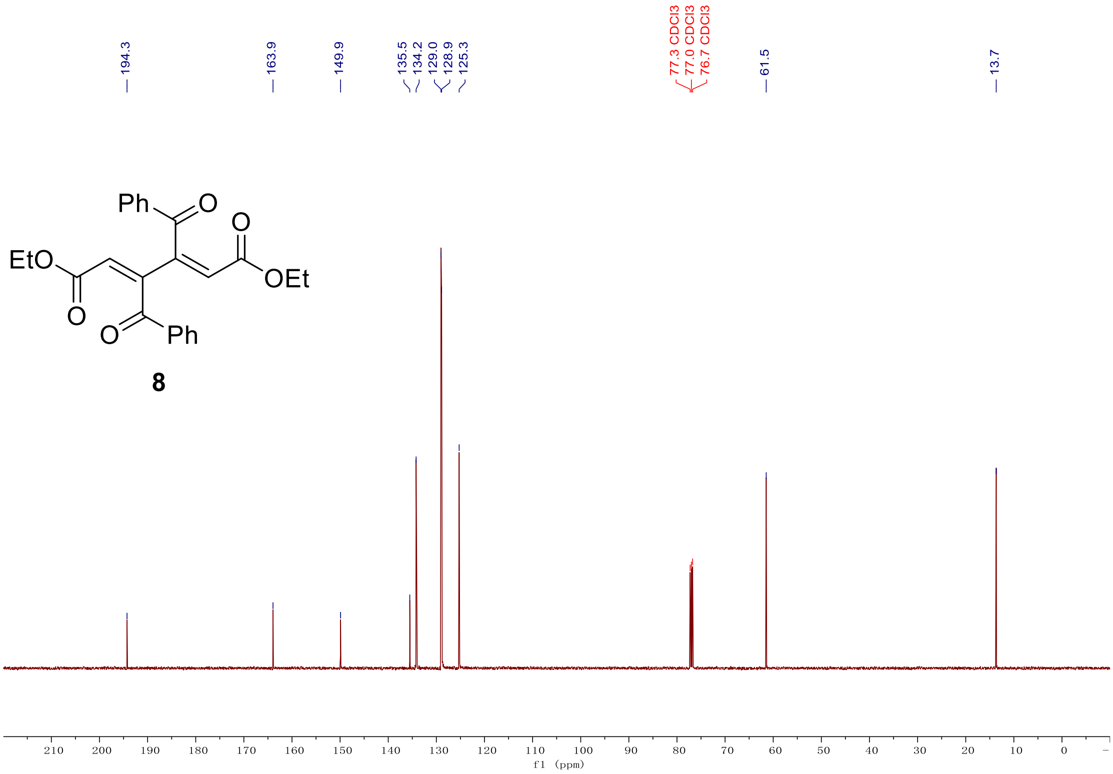

Supplementary Figure 234. ^{13}C NMR (125 MHz, $CDCl_3$) spectrum for compound **8**

9. References

[1] Liao, L.; Zhang, H.; Zhao, X. Selenium-π-Acid Catalyzed Oxidative Functionalization of Alkynes: Facile Access to Ynones and Multisubstituted Oxazoles. *ACS Catal.* **2018**, *8*, 6745−6750.

[2] Dong, X.; Jiang, W.; Hua, D.; Wang, X.; Xu, L.; Wu, X. Radicalmediated vicinal addition of alkoxysulfonyl/fluorosulfonyl and trifluoromethyl groups to aryl alkyl alkynes. *Chem. Sci.* **2021**, *12*, 11762−11768.

[3] Suarez, A.; Fu, G. C. A Straightforward and Mild Synthesis of Functionalized 3-Alkynoates. *Angew. Chem. Int. Ed.* **2004**, *43*, 3580−3582.
